# Cosmetics Europe compilation of historical serious eye damage/eye irritation in vivo data analysed by drivers of classification to support the selection of chemicals for development and evaluation of alternative methods/strategies: the Draize eye test Reference Database (DRD)

**DOI:** 10.1007/s00204-016-1679-x

**Published:** 2016-03-21

**Authors:** João Barroso, Uwe Pfannenbecker, Els Adriaens, Nathalie Alépée, Magalie Cluzel, Ann De Smedt, Jalila Hibatallah, Martina Klaric, Karsten R. Mewes, Marion Millet, Marie Templier, Pauline McNamee

**Affiliations:** 1 Cosmetics Europe - The Personal Care Association, Brussels, Belgium; 2 European Commission Joint Research Centre, Institute for Health and Consumer Protection, EURL ECVAM/Systems Toxicology Unit, Ispra, VA Italy; 3 Front End Innovation - Toxicology, Beiersdorf AG, Hamburg, Germany; 4 Adriaens Consulting BVBA, Aalter, Belgium; 5 Department of Predictive Biological Methods and Models, L’Oréal Research and Innovation, Aulnay Sous Bois, France; 6 Product Safety Department, LVMH Recherche - LVMH Parfums et Cosmétiques, St. Jean de Braye Cedex, France; 7 Preclinical Development and Safety, Janssen Research and Development, Beerse, Belgium; 8 Toxicology Department, Chanel Parfums Beauté, Neuilly sur Seine, France; 9 Biological and Clinical Research, Henkel AG and Co. KGaA, Düsseldorf, Germany; 10 In Vitro Metabolism and Alternative Methods, Pierre Fabre, Castres, France; 11 Central Product Safety, The Procter and Gamble Company, Egham, Surrey, UK

**Keywords:** UN GHS/EU CLP, Drivers of classification, Eye irritation/serious damage to the eye, Draize eye test Reference Database, Validation of alternative methods, Chemicals selection

## Abstract

A thorough understanding of which of the effects assessed in the in vivo Draize eye test are responsible for driving UN GHS/EU CLP classification is critical for an adequate selection of chemicals to be used in the development and/or evaluation of alternative methods/strategies and for properly assessing their predictive capacity and limitations. For this reason, Cosmetics Europe has compiled a database of Draize data (Draize eye test Reference Database, DRD) from external lists that were created to support past validation activities. This database contains 681 independent in vivo studies on 634 individual chemicals representing a wide range of chemical classes. A description of all the ocular effects observed in vivo, i.e. degree of severity and persistence of corneal opacity (CO), iritis, and/or conjunctiva effects, was added for each individual study in the database, and the studies were categorised according to their UN GHS/EU CLP classification and the main effect driving the classification. An evaluation of the various in vivo drivers of classification compiled in the database was performed to establish which of these are most important from a regulatory point of view. These analyses established that the most important drivers for Cat 1 Classification are (1) CO mean ≥ 3 (days 1–3) (severity) and (2) CO persistence on day 21 in the absence of severity, and those for Cat 2 classification are (3) CO mean ≥ 1 and (4) conjunctival redness mean ≥ 2. Moreover, it is shown that all classifiable effects (including persistence and CO = 4) should be present in ≥60 % of the animals to drive a classification. As a consequence, our analyses suggest the need for a critical revision of the UN GHS/EU CLP decision criteria for the Cat 1 classification of chemicals. Finally, a number of key criteria are identified that should be taken into consideration when selecting reference chemicals for the development, evaluation and/or validation of alternative methods and/or strategies for serious eye damage/eye irritation testing. Most important, the DRD is an invaluable tool for any future activity involving the selection of reference chemicals.

**Electronic supplementary material:**

The online version of this article (doi:10.1007/s00204-016-1679-x) contains supplementary material, which is available to authorized users.

## Introduction

Several prospective and retrospective validation studies/activities for in vitro test methods in the area of serious eye damage/eye irritation have taken place over the last 20 years. These activities have led to the adoption of several methods by the Organisation for Economic Co-operation and Development (OECD) as partial replacement alternatives to the regulatory in vivo Draize rabbit eye test, i.e. OECD Test Guideline (TG) 405 (OECD 2012a). Currently, four test methods are accepted by the OECD to classify chemicals as inducing serious eye damage according to the United Nations Globally Harmonized System of Classification and Labelling of Chemicals (UN GHS) (UN 2013) and the European Union Regulation on Classification, Labelling and Packaging of chemicals (EU CLP) implementing UN GHS in EU (EC 2008) (UN GHS/EU CLP Category 1, herein after referred to as Cat 1). These are two organotypic assays, the Bovine Corneal Opacity and Permeability (BCOP) test method (OECD TG 437) and the Isolated Chicken Eye (ICE) test method (OECD TG 438) (OECD 2013a, b), and two cell-based assays, the Fluorescein Leakage (FL) test method (OECD TG 460) (OECD 2012b) and more recently the short-time exposure (STE) test method (Takahashi et al. 2008, 2009; Sakaguchi et al. 2011) validated by the Japanese Center for the Validation of Alternative Methods (JaCVAM) (Kojima et al. 2013) and adopted by the OECD in 2015 (OECD TG 491) (OECD 2015a). The BCOP, ICE and STE are also accepted by the OECD for identifying chemicals not requiring classification for serious eye damage/eye irritation (UN GHS/EU CLP No Category; herein after referred to as No Cat) (OECD 2013a, b, 2015a). Furthermore, a new TG (OECD TG 492) was also adopted by the OECD in 2015 for identifying chemicals not requiring classification for serious eye damage/eye irritation (No Cat) (OECD 2015b). TG 492 describes the Reconstructed human Cornea-like Epithelium (RhCE) test method EpiOcular™ Eye Irritation Test (EIT) that was validated in a prospective study coordinated by EURL ECVAM/Cosmetics Europe (Freeman et al. 2010). Finally, the Cytosensor Microphysiometer (CM) test method (Hartung et al. 2010) has been endorsed as scientifically valid for limited applicability domains (EURL ECVAM 2008b; ESAC 2009; ICCVAM 2010) and is currently in the process of review by the OECD for the identification of chemicals inducing serious eye damage (Cat 1) as well as chemicals not requiring classification for serious eye damage/eye irritation (No Cat).

Partial replacement of the in vivo Draize rabbit eye test has been achieved through application of the regulatory accepted in vitro test methods that are mentioned above. Several analyses have been conducted to understand the limitations of alternative methods for predicting in vivo serious eye damage/eye irritation (Scott et al. 2010) and to explain why full replacement has not yet been reached (Bruner et al. 1998; York and Steiling 1998; Balls et al. 1999; Adriaens et al. 2014). These reviews identified key causes that are briefly discussed below. For instance, in validation studies/activities performed during the 1990s (Balls et al. 1999) the results of the in vitro methods were often correlated with the modified maximum average score (MMAS) of the Draize eye test. The MMAS is a sum score of weighted individual tissue scores which combines effects of the test chemical on the cornea, iris and conjunctiva into one score occurring at 24 h or later after instillation without taking into account the reversibility of the effects. In contrast, more recent retrospective validation activities (ICCVAM 2007, 2010; EURL ECVAM 2008a, b) have already used the UN GHS/EU CLP classification for serious eye damage/eye irritation (UN 2013; EC 2008). In addition, the Eye Irritation Reference Chemicals data bank published by the European Centre for Toxicology and Ecotoxicology of Chemicals (Bagley et al. 1992; ECETOC 1992) was many times the main, if not the only, database used for selecting test chemicals for validation studies. At that time, this database contained only 55 chemicals (72 in vivo studies in total), and it was therefore not a comprehensive representation of chemical classes. Since this version of the ECETOC database was a fundamental source of in vivo data used in validation studies conducted in the 1990s, e.g. EC/HO (Balls et al. 1995) and COLIPA, now called Cosmetics Europe (Brantom et al. 1997), the scope of chemicals selection was, to a certain extent, limited. Subsequently, a second more comprehensive version of the ECETOC data bank extended to 132 chemicals (149 in vivo studies in total) was published in 1998 (Bagley et al. 1999; ECETOC 1998). Furthermore, variability of the responses observed between rabbits from historical data normally used as the in vivo reference in validation studies (Scott et al. 2010) was identified as potentially influencing the outcome of validation activities and acceptance of in vitro test methods. The impact of the uncertainty of in vivo reference data on the evaluation/validation of alternative methods was already illustrated by the resampling analysis presented by Adriaens et al. (2014). This analysis showed that the Draize eye test is prone to high misclassification errors. Importantly, these misclassification errors are unidirectional towards lower classifications. This means that about 12 % of the chemicals classified as Cat 2 and at least 11 % of those classified as Cat 1 could in fact be equally identified as No Cat and as Cat 2, respectively, by the in vivo Draize eye test considering only its within-test variability. This demonstrates that the way the Draize eye test data are interpreted is very conservative and may over-predict the true irritation potential of chemicals. As such, this over-prediction of Cat 1 and Cat 2 chemicals in the in vivo Draize eye test needs to be taken into account for determining acceptance of in vitro test methods. Based on this, Adriaens and co-workers therefore suggested to reconsider the UN GHS/EU CLP decision criteria for classification. This is in terms of the biological relevance of persistence of low-level conjunctival effects in driving Cat 1 classification in the absence of any other Cat 1 triggering effects and Cat 1 classification driven by persistent effects or corneal opacity of grade 4 in a single animal, whereas the majority of the animals recover completely by day 21.

It is of importance to note that although more recent validation studies/activities such as those mentioned in the first paragraph have used the UN GHS/EU CLP classification for serious eye damage/eye irritation (UN 2013; EC 2008) to interpret the in vivo data, consideration of the ocular tissues effects that drive classification were not integrated in most of them. More recently, a comprehensive in-depth analysis of historical in vivo rabbit eye data co-sponsored by Cosmetics Europe and the European Commission was performed. This provided more insight into which of the observed in vivo effects are important in driving the classification of chemicals for serious eye damage/eye irritation (Adriaens et al. 2014) according to the UN GHS/EU CLP classification (UN 2013; EC 2008). The insights gained have identified that, in fact, the main reason that partial replacement only has been achieved, can be attributed to not taking into account the impact of the individual in vivo tissue effects driving classification. In the Draize rabbit eye test, the hazard potential of a test chemical is determined based on its effect on corneal opacity (CO), iritis (IR), conjunctival redness (CR) and conjunctival chemosis (CC) in combination with full reversibility or persistence of any effect on the 21st day after instillation. In order to achieve full replacement of the in vivo Draize eye test, it is clear that in vitro test methods, alone or in combination, need to address the main ocular tissue effects that drive classification. In this respect, a thorough understanding of what drives classification of chemicals in the in vivo rabbit Draize eye test is a critical and essential element to consider in the development of alternative methods, evaluation of their predictive capacity and limitations and identification of the applicability of a specific assay. The BCOP and ICE, for example, were developed to detect immediate corneal effects, equivalent to the first three observation days in the Draize eye test. However, both test methods, using current protocols (OECD 2013a, b), lack the ability to consistently identify delayed in vivo effects or mild/moderate in vivo effects that persist until day 21. Two other organotypic test methods, the Ex Vivo Eye Irritation Test (EVEIT) using isolated rabbit corneas (Spöler et al. 2007; Frentz et al. 2008) and the Porcine Corneal Ocular Reversibility Assay (PorCORA) using isolated porcine corneas (Piehl et al. 2010, 2011), have been developed to address reversibility/persistence of effects. Both test methods are intended to directly monitor recovery in exposed excised corneas kept in culture over several days following test item administration. However, neither test method has yet undergone formal validation.

To succeed in the future to fully replace the Draize rabbit eye test, a consistent approach is needed to identify chemicals covering the different drivers of classification (Barroso et al. 2013; Adriaens et al. 2014) for use in development, evaluation and validation of in vitro test methods. For this purpose, Cosmetics Europe undertook to compile an extensive list of chemicals for which historical in vivo Draize eye test data obtained according to OECD TG 405 (OECD 2012a) are available. These data were sourced from several external databases that had been compiled and were used to support past validation activities for in vitro test methods. The comprehensive database developed by Cosmetics Europe systematically covers all drivers of classification based on the observed tissue effects, relevant chemical classes and physical states. This means that it can be used to appropriately select chemicals for the development and evaluation of in vitro test methods. This approach will facilitate an early and thorough assessment of the performance of a new in vitro test method and will help better identify its limitations and applicability within testing strategies such as those suggested by Scott et al. (2010). Another consequence of an appropriate selection of chemicals is that future validation studies/activities may be performed on a smaller and more focused dataset of chemicals covering all important drivers of classification.

Taken together, the key goals for compiling this list were: (1) to enable a comprehensive analysis and understanding regarding in vivo drivers of classification based on the Draize eye test, (2) to further evaluate the variability of the Draize eye test based on data obtained from repeat studies, (3) to make available an extensive list of chemicals with TG 405 in vivo data, beyond those generally used historically, for further method development and validation and (4) to provide guidance for selecting reference chemicals based on understanding ocular tissue effects that drive classification in the in vivo rabbit Draize eye test. Beyond this, based on the unprecedented in-depth analysis presented in this paper, a critical revision of the UN GHS/EU CLP decision criteria for classification is advocated.

## Development of the Draize eye test Reference Database (DRD)

### Data sources

The Draize eye test Reference Database (DRD) provided in Supplementary Material 1 was compiled using different sources of historical in vivo Draize eye test data which were created to support past validation activities. These data were produced according to OECD TG 405 (OECD 2012a) using proprietary and commercially available chemicals. The data sources used were (1) the Eye Irritation Reference Chemicals Data Bank developed by ECETOC (Bagley et al. 1992, 1999; ECETOC 1998); (2) a database developed by ZEBET (Spielmann et al. 1996); (3) the database from Laboratoire National de la Santé (LNS) (Gautheron et al. 1994); (4) the database developed by the National Toxicology Program Interagency Center for the Evaluation of Alternative Toxicological Methods (NICEATM) to support the retrospective evaluations of the Bovine Corneal Opacity and Permeability (BCOP) test method, the Isolated Chicken Eye (ICE) test method, the Isolated Rabbit eye (IRE) test method, and the Hen’s Egg Test-Chorioallantoic Membrane (HET-CAM) that were performed by the Interagency Coordinating Committee on the Validation of Alternative Methods (ICCVAM) (ICCVAM 2007, 2010); (5) the database developed by EURL ECVAM to support the prospective validation study of RhCE-based test methods performed by EURL ECVAM and Cosmetics Europe; and (6) five studies that were not included in the other databases but that were used in the Cosmetics Europe study on the use of HPLC/UPLC spectrophotometry in Reconstructed human Tissue (RhT)-based test methods (Alépée et al. 2015).

### UN GHS classification

The studies on commercially and proprietary available chemicals collected in the DRD (Supplementary Material 1) were classified according to the serious eye damage/eye irritation classification criteria defined by UN GHS (UN 2013) and EU CLP (EC 2008), which implemented UN GHS in the EU. These classification criteria are derived from testing in albino rabbits according to the Draize eye test method (OECD 2012a) and are primarily based on the severity of effects and/or the timing of their reversibility. According to the UN GHS/EU CLP classification system, Category 1 (Cat 1) is defined as causing irreversible effects on the eye/serious damage to the eye and Category 2 (Cat 2) as causing reversible effects (fully reversible within 21 days) on the eye/eye irritation. UN GHS offers the possibility to further subcategorise Cat 2 into two optional categories, i.e. Category 2A (Cat 2A) (irritant to eyes) when the eye effects are not fully reversible within 7 days of observation and Category 2B (Cat 2B) (mildly irritant to eyes) when the eye effects fully reverse within 7 days of observation. These two optional categories were not implemented in EU CLP. If none of the criteria for Cat 1 and Cat 2 are met, the chemical does not require classification for serious eye damage/eye irritation and therefore No Category (No Cat) is assigned. An overview of the UN GHS/EU CLP classification criteria is presented in Table 1. According to this classification system there are 11 different criteria derived from the four tissue effects assessed in the Draize eye test, namely corneal opacity (CO), iritis (IR), conjunctival redness (CR) and conjunctival chemosis (CC), which can each independently drive the classification of a chemical. These different criteria are here named as “drivers of classification” and they are: “CO mean ≥ 3” and “IR mean > 1.5” for Cat 1 based on immediate severity appearing during the first three observation days in ≥60 % of the animals (i.e. in at least 2 out of 3, 3 out of 4, 3 out of 5 or 4 out of 6); “CO pers D21”, “CR pers D21”, “CC pers D21” and “IR pers D21” for Cat 1 based on persistence (pers) of effects on day 21 (D21) observed in at least 1 animal; “CO = 4” for Cat 1 based on specific observations made in at least 1 animal; “CO mean ≥ 1”, “CR mean ≥ 2”, “CC mean ≥ 2” and “IR mean ≥ 1” for Cat 2 based on effects appearing during the first three observation days in ≥60 % of the animals (i.e. in at least 2 out of 3, 3 out of 4, 3 out of 5 or 4 out of 6). In previous analyses it was shown that CC rarely drives the classification of chemicals on its own (about 2 % of the Cat 2 chemicals) (Adriaens et al. 2014; Barroso and Norman 2014) and can therefore be considered unimportant as a driver of classification. It was therefore decided not to report CR and CC separately in this study. CR and CC were thus independently assessed as defined in UN GHS/EU CLP but reported together as “Conj pers D21” for Cat 1, and “Conj mean ≥ 2” for Cat 2, leading to a total of nine different drivers of classification instead of the original 11 criteria mentioned above (Tables 1, 2).

**Table 1:** Classification rules defined by UN GHS (UN 2013) and EU CLP (EC 2008)

Endpoint	Range scores^a^	**Category 1** ^b^ Irreversible effects on the eye/serious eye damage	**Category 2** ^c^ Reversible effects on the eye/eye irritation
		**Draize severity scores** ^**a**^ **and/or persistence of effects on day 21**	**Draize severity scores** ^**a**^
Corneal opacity (CO)	0–4	**CO mean ≥ 3** (in ≥60 % of the tested animals), **OR** CO > 0 on day 21 in at least 1 animal (**CO pers D21**), **OR CO** **=** **4** at any time point in any animal, **OR**	**CO mean ≥ 1** (in ≥60 % of the tested animals), **OR**
Iritis (IR)	0–2	**IR mean > 1.5** (in ≥60 % of the tested animals), **OR** IR > 0 on day 21 in at least 1 animal (**IR pers D21**), **OR**	**IR mean ≥ 1** (in ≥60 % of the tested animals), **OR**
Conjunctival redness (CR)	0–3	CR > 0 on day 21 in at least 1 animal (**CR pers D21**), **OR**	**CR mean ≥ 2** (in ≥60 % of the tested animals), OR
Conjunctival chemosis (CC)	0–4	CC > 0 on day 21 in at least 1 animal (**CC pers D21**)	**CC mean ≥ 2** (in ≥60 % of the tested animals)

Bold text indicates criteria for the different ocular tissues that drive classification

^a^Mean scores are calculated for each animal from gradings at 24, 48, and 72 h after instillation of the test chemical and these “severity scores” are then used to determine the classification of the test chemical

^b^Cat 1 also applies when other severe reactions (e.g. destruction of cornea, discoloration of the cornea by a dye substance, adhesion, pannus, or interference with the function of the iris or other effects that impair sight) are observed at any time point in any rabbit during the observation period

^c^All effects have to fully reverse within an observation period of normally 21 days. UN GHS provides the option to distinguish this single hazard category into two optional subcategories (not implemented in EU CLP): “Category 2A” (irritant to eyes) when any of the eye effects in any animal is not fully reversible within 7 days of observation (i.e. CO, IR, CR and/or CC > 0 at 7 ≤ day < 21); “Category 2B” (mildly irritant to eyes) when all observed eye effects are fully reversible within 7 days of observation (i.e. CO, IR, CR and CC = 0 on day 7 and beyond)

**Table 2:** List of the in vivo drivers of UN GHS classification for the chemicals requiring classification and subgroups for the chemicals not requiring classification

Category 1						Category 2^a^			No Category			
Severity (mean scores of days 1–3)^b^		Persistence on day 21			Severe CO	Severity (mean scores of days 1–3)^b^						
**CO mean ≥ 3**	**IR mean > 1.5**	**CO**	**Conj**	**IR**	**CO** **=** **4**	**CO mean ≥ 1**	**Conj mean ≥ 2**	**IR mean ≥ 1**	**CO** **>** 0**	**CO** **>** **0**	**CO** **=** 0**	**CO** **=** **0**
In ≥60 % of the animals	In ≥60 % of the animals	In at least one animal	In at least one animal	In at least one animal	In at least one animal	In ≥60 % of the animals	In ≥60 % of the animals	In ≥60 % of the animals	In at least one observation time in at least one animal		In all observation times in all animals	

CO, corneal opacity; IR, iritis; Conj, conjunctival redness (CR) and/or conjunctival chemosis (CC)

** Indicates at least one animal with a mean score of days 1–3 above the classification cut-off for at least one endpoint

^a^Subcategorised in two categories: Category 2A (irritant to eyes) when any of the eye effects in any animal is not fully reversible within 7 days of observation (i.e. CO, IR, CR and/or CC > 0 at 7 ≤ day < 21) and 2B (mildly irritant to eyes) when all observed eye effects are fully reversible within 7 days of observation (i.e. CO, IR, CR and CC = 0 on day 7 and beyond)

^b^Mean scores are calculated from gradings at 24, 48 and 72 h after instillation of the test chemical

### Categorisation of the studies according to their main driver of classification

A chemical can be classified based on a single or multiple drivers of classification. All drivers of classification observed in each study are reported in the DRD (Supplementary Material 1). For the purpose of this publication, the Cat 1 and Cat 2 studies were grouped according to their main driver of classification (Table 2), which is shown in boldface within a greyed cell for every Cat 1 and Cat 2 study reported in the DRD (Supplementary Material 1). The selection of the main driver of classification in each study was done according to the following prioritisation scheme. Chemicals classified as Cat 1 were first grouped based on (1) severity (mean scores of days 1–3); (2) persistence of any ocular effect on day 21 in the absence of severity; or (3) CO = 4 (at any observation time during the study) in the absence of both severity and persistence (or if unknown). CO = 4 was given the lowest priority because, in many cases for ethical reasons, studies showing CO = 4 were terminated before day 21, thereby having less complete data and lacking information on the persistence/reversibility of the effects. In some cases, information on severity was even not available because the study was terminated before day 3. Next, for the severity and persistence groups, the endpoint (“CO”, “IR”, “Conj”) showing the largest number of animals fulfilling the classification criterion was chosen as the main in vivo driver of classification. If equal number of animals fulfilled the classification criteria for different drivers, highest priority was given to CO, followed by Conj and finally IR when selecting the main driver of classification (Table 2). Chemicals classified as Cat 2 were allocated to one of three different groups, with the endpoint (“CO”, “Conj”, and “IR”) showing the largest number of animals fulfilling the classification criterion being chosen as the main in vivo driver of classification (Table 2). If equal number of animals fulfilled the classification criteria for different drivers, highest priority was again given to CO, followed by Conj and finally IR when selecting the main driver of classification (Table 2). For the Cat 2 chemicals, the persistence of effects on day 7 was also annotated to allow differentiation between Cat 2B and Cat 2A (Supplementary Material 1).

The prioritisation of the different endpoints within the severity and persistence groups, i.e. CO > Conj > IR, is based and builds on the analyses published by Adriaens et al. (2014). In that paper evidence was provided that CO is the most important endpoint driving the classification of Cat 1 chemicals and is almost as important as CR in driving the classification of Cat 2 chemicals (Adriaens et al. 2014; Barroso and Norman 2014). Considering that effects on the cornea can lead to visual impairment while conjunctival effects are of lesser importance in this respect, CO was given the highest priority followed by conjunctival effects (CR/CC). Iritis was found to rarely drive the classification of chemicals (<4 % of both Cat 1 and Cat 2 chemicals) and is mostly accompanied by corneal effects generating the same classification (Supplementary Material 1). Iritis was therefore given the lowest priority.

Studies with chemicals not requiring classification for serious eye damage/eye irritation (No Cat) were distributed in four different groups depending on whether they showed CO scores equal to 0 in all animals and all observed time points (CO = 0) or not (CO > 0). Only CO was analysed for scores equal to or higher than 0, in line with it being defined as the endpoint with highest priority. Nevertheless, No Cat studies for which at least one animal had a mean of the scores of days 1–3 above the classification cut-off for at least one endpoint but not enough animals to generate a classification (borderline cases) were marked with **. For example, study No. 324 (*N*,*N*-Dimethyl guanidine sulphate) was assigned to the subgroup CO = 0** because the CO scores were equal to 0 in all animals and all time points, and because one out of three animals showed a mean of the CR scores of days 1–3 equal to or higher than 2 (Supplementary Material 1). If any of the other two animals had also fulfilled this criterion (CR mean ≥ 2), the chemical would have been classified as Cat 2. Study No. 275 (1,2,3-Trichloropropane) is an example of a chemical assigned to subgroup CO > 0 because all three tested animals showed a CO score > 1 at hour 1 and two of those animals also on day 1 (Supplementary Material 1). However, none of these animals showed a mean of the scores of days 1–3 above the classification cut-off for any of the endpoints and therefore the ** do not apply.

Finally, for several studies the data available were not sufficient to allow a definitive and unambiguous classification of the tested chemical due to one or several of the following reasons: (1) if the study was terminated before 21 days without full reversibility (scores equal to 0) of all endpoints in all animals, in the absence of any other effects driving a Cat 1 classification, (2) if only two animals were used and no effects driving a Cat 1 classification were observed, or (3) if only one animal was used and no CO = 4 and/or persistent effects were observed. These studies were identified as Study Criteria Not Met (SCNM). Where possible considering the available data, the most probable classification of the chemicals tested in these studies was indicated in brackets after SCNM (Supplementary Material 1).

### Draize eye test Reference Database (DRD)

The DRD (Supplementary Material 1) contains 681 independent Draize eye test studies, which are identified in the first three columns by a “Study Number” (ranging from 1 to 681), “Test Chemical Name” and the Chemical Abstracts Service Registry Number (“CAS RN”) of the test chemical. Chemical classes were assigned to most of the chemicals listed in the DRD according to OECD QSAR Toolbox analysis (version 3.2; http://www.oecd.org/chemicalsafety/risk-assessment/theoecdqsartoolbox.htm). A profiler comprising around 430 nested categories was implemented which assigns effective organic class according to full functional group, without subdivision of component fragments. For example, RCOOH would be classed as carboxylic acid only, without allocation of ketone and alcohol classes, respectively, to subsidiary CO and OH constituents. The chemical classes assigned to each chemical are listed in the column “Organic Functional Groups”. Additionally, three inorganic salts were identified.

The studies included in the DRD were first ordered by UN GHS classification starting with Cat 1 (No. 1–165), followed by Cat 2A (No. 166–216), Cat 2 (No. 217), Cat 2B (No. 218–244), No Cat (No. 245–587), and finally SCNM (No. 588–681). Within each one of these UN GHS groups, the studies were sorted by main driver of classification. Within each main driver of classification, the studies were sorted by “physical form as tested” in the in vivo study (i.e. (1) liquid: “L”, (2) tested in solvent but physical state of neat chemical unknown: “L (tested in solvent, neat chemical unknown)”, (3) tested in solvent but neat chemical available as solid: “L (tested in solvent, available as S)”, (4) waxy/viscous solid: “S (waxy)”, (5) solid: “S”, and (6) unknown physical state: “unknown”). Finally, within each category of “physical form as tested” the studies were sorted in alphabetic order of the test chemical name. The column “Data Source” refers to the source of the data (i.e. ECETOC, ZEBET, LNS, NICEATM, EURL ECVAM and Cosmetics Europe). The commercial source (availability today; provided as an example) and the available purity are also provided. The CAS RN, name and physical form of the raw chemical were verified one by one by three independent investigators using Material Safety Data Sheets (MSDS), where available, or any other reliable source of information. This resulted in the identification of some transposition errors in the original in vivo data source that were corrected in the current database (e.g. study No. 532 was reported with CAS RN 118-82-3 in ECETOC, while the correct CAS RN for this chemical, as mentioned in the DRD, is 118-82-1). In a few cases, the physical state of the raw chemical provided in the DRD could not be verified with information available today, but since a specific physical state was clearly indicated in the in vivo study report it was decided to report this in the DRD rather than “unknown”. The column “Number of Studies” gives information on the number of available in vivo Draize eye test studies for that specific chemical: 1 of 1 refers to a single study, and 1 of 2 and 2 of 2 indicate that there are two independent in vivo studies for this chemical, etc. The following columns give detailed information on the in vivo drivers of classification observed in each study, as described in Table 2, with the main driver of classification marked in boldface within a greyed cell. The column “Comments” contains all available detailed information on special observations such as CO = 4 (timing of appearance, reversibility, the number of animals that were affected) and persistence of effects (e.g. number of animals affected and tissue scores). Finally, the last column in the DRD highlights those chemicals in the database that are not recommended to be used in method development and/or validation due to limited quality and/or reliability of the in vivo classification, despite the fact that they may have been used in past validation activities (for more details see chapter “Drivers of classification criteria to consider when selecting reference chemicals” below.

## Results and discussion

For the successful development and validation of alternative methods and strategies to fully replace the Draize rabbit eye test, a thorough understanding of the in vivo tissue effects that drive classification is of primary importance. In the “Results and discussion”, each chapter focuses on an individual element of this in-depth analysis. Detailed information is provided and discussed from which key conclusions (hereinafter referred to as evidence 1–9) are drawn in each chapter. The first two chapters focus on the distribution of the studies presented in the DRD (Supplementary Material 1) according to the UN GHS classification and the in vivo drivers of classification. Next, key points such as the variability between repeat Draize eye studies and the classification of chemicals as Cat 1 based only on persistence of effects are discussed in detail and are illustrated with typical examples. Based on these in-depth analyses, a critical review of the UN GHS/EU CLP decision criteria for classification is presented, and a revision of these criteria is advocated. This paper concludes with guidance and key criteria that should be considered when selecting references chemicals for the development and/or validation of alternative methods and/or for the development and evaluation of testing strategies. The evidence numbers provided in the various chapters below link the various observations and conclusions in the manuscript with the key criteria recommended for selecting reference chemicals. Evidence numbers were further subdivided into a, b, c, etc., depending on the effect being discussed, in order to provide further precision in the linking between evidence and recommendations.

### Distribution of studies according to UN GHS classification

The DRD contains data on 681 Draize eye test studies representing 634 unique chemicals and chemical solutions/suspensions in a solvent. For 94 of these 681 studies (13.8 %) UN GHS study criteria allowing an unambiguous classification were not met. These are identified as SCNM in the DRD. Among the 587 studies which met the UN GHS criteria for classification (representing 547 unique chemicals and chemical solutions/suspensions in a solvent), 41.6 % were classified, with 13.5 % being Cat 2 and 28.1 % being Cat 1 (Table 3). This distribution is very similar to that reported by Adriaens et al. (2014) for three reference databases that are included in the DRD (ECETOC + ZEBET + LNS: 17.2 % Cat 2 and 22.6 % Cat 1), but differs substantially from the prevalence of Cat 2 (10.4 %) and Cat 1 (6.9 %) chemicals in the European New Chemicals Database (NCD) of the ex-European Chemicals Bureau containing data on “New Chemicals” notified under Directive 67/548/EEC and introduced to the EU industrial market after September 1981 (Adriaens et al. 2014). Although the DRD contains several studies on NCD chemicals for which full raw data are available, most of the studies included in the DRD have been collected over the years specifically to support validation activities, where the goal was probably to increase the proportion of Cat 2 and Cat 1 chemicals rather than be reflective of what is observed in reality. The distribution of UN GHS categories observed in the NCD is therefore expected to represent more closely the true prevalence of Cat 2 and Cat 1 chemicals.

**Table 3:**
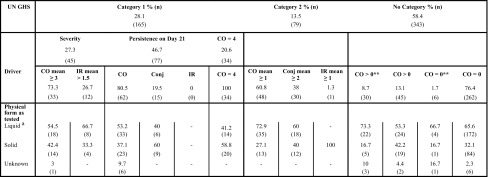
Proportion (%) and number (*n*) of studies according to main driver of classification (chemicals requiring classification) or according to the subgroups (No Cat); the proportions within a framed cell add up to 100 %

CO, corneal opacity; IR, iritis; Conj, conjunctival redness (CR) and/or conjunctival chemosis (CC)

A few chemicals (*n* = 9, 5.5 %: No. 157–165) (1 liquid, 6 solids and 2 physical state unknown) were classified based on “other observations” but not severity, or persistence or CO = 4 and are therefore omitted in the distribution of Cat 1 chemicals

** Indicates at least one animal with a mean score of days 1–3 above the classification cut-off for at least one endpoint

^a^ Includes solids and liquids tested in solvent

### Distribution of studies according to in vivo drivers of classification

**Fig. 1:**
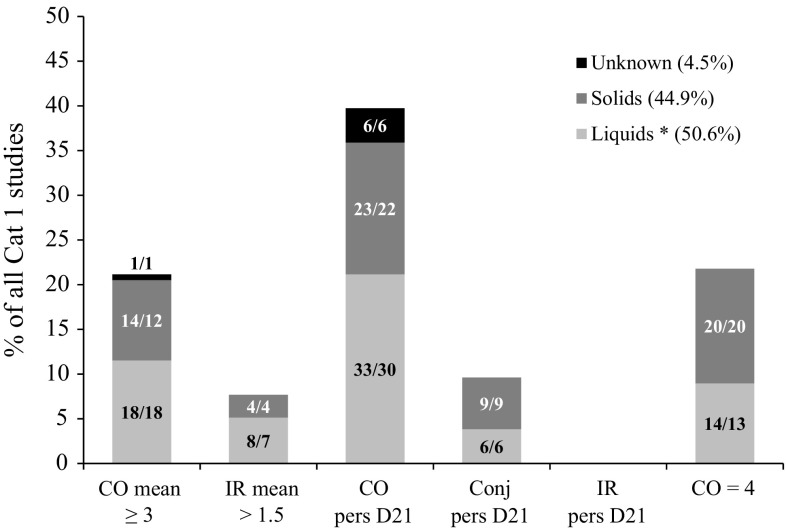
Distribution of 156 UN GHS/EU CLP Cat 1 studies according to their main drivers of classification and the physical state of the chemical as tested. The *numbers* in the *bars* correspond to the number of studies/the number of unique test chemicals. For selecting the main driver of classification in each study, highest priority was first given to severity (CO mean ≥ 3 and/or IR mean > 1.5 of days 1–3), followed by persistence of effects on day 21 in the absence of severity (CO, Conj and/or IR pers D21), followed by CO = 4 (at any observation time during the study) in the absence of both severity and persistence (or if unknown) Within the severity and persistence groups, the main driver corresponds to the one showing the largest number of animals fulfilling the classification criterion. If equal number of animals fulfilled the classification criteria for different drivers, the main driver was selected following the order shown on the *x*-axis, with priority decreasing from left to right. Note: three studies performed with a single animal showed CO mean ≥ 3, IR mean > 1.5, CO pers D21, Conj pers D21 and IR pers D21 (studies No. 50, 73 and 78), but were placed in the group CO pers D21 because the group CO mean ≥ 3 can only be selected when this occurs in ≥60 % of the animals in studies with at least three animals. Similarly, another five studies performed with a single animal showed CO mean ≥ 3 and CO = 4 (one also showed IR mean > 1.5), but were placed in the group CO = 4 (studies No. 136, 138, 142, 143 and 146). Liquids *: includes solids and liquids tested in solvent

In order to better understand the relative importance of the different drivers of classification, each study was allocated to a single main driver of classification according to the described prioritization scheme (Table 2). The studies classified as Cat 1 (*n* = 165) were distributed according to six main drivers, two for severity in the first three observation days, three for persistence on day 21 and one for CO = 4. As can be observed in Table 3, 27.3 % of all Cat 1 studies were classified based on severity, with CO mean ≥ 3 being the main driver of classification for the majority of these studies (73.3 %) (evidence 1a) and IR mean > 1.5 being the main driver in only 26.7 % of these studies (evidence 2a). About 47 % of all Cat 1 studies were classified based only on persistence of effects on day 21 (i.e. with CO mean < 3 and IR mean ≤ 1.5). For 80.5 % of these studies CO persistence was the main driver (evidence 1b), while only 19.5 % of these studies had conjunctival (CR and/or CC) persistence as the main driver (evidence 2b) and none had IR persistence as the main driver (evidence 2c). Studies classified based on CO = 4 as main driver, represented 20.6 % of all Cat 1 studies (evidence 1c). Of note, a few studies (*n* = 9, 5.5 %) led to a Cat 1 classification based on other observations (e.g. pannus formation, discoloration of the cornea) but not on severity, persistence or CO = 4 (evidence 3). These studies will not be further discussed in the current paper and therefore the total number of Cat 1 studies considered hereafter is reduced to 156. The distribution of these 156 Cat 1 studies according to their main drivers of classification and subgrouping according to the physical form of the chemicals as tested are presented in a cumulative bar chart in Fig. 1 (in % relative to the 156 studies). Table 3 also shows the distribution of the physical form of the chemicals as tested within each main driver of classification (in % relative to the total number of studies for each individual driver). Among the 156 Cat 1 studies, 50.6 % were performed with liquids or chemicals tested in solvent, 44.9 % were performed with solids and for 4.5 % the physical state of the test chemical is unknown (Fig. 1). Studies classified based on conjunctival persistence on day 21 or studies classified based on CO = 4 as main driver involved more solids than liquids (including chemicals tested in solvent) (Fig. 1; Table 3). For the other main drivers, the distribution was mostly similar to the overall distribution of liquids versus solids seen for all Cat 1 chemicals (Fig. 1; Table 3). Among the six possible drivers that can result in a Cat 1 classification, CO persistence on day 21 is the one observed most often as main driver (evidence 1b). This tissue effect is responsible for classification of about 40 % of the studies. In contrast, none of the Cat 1 studies was classified based on IR persistence on day 21 as main driver (Fig. 1) (evidence 2c). Furthermore, corneal opacity (including CO mean ≥ 3, CO persistence on day 21, and CO = 4) is the main endpoint driving Cat 1 classification, representing 82.7 % of all Cat 1 studies (evidence 1a, b, c). IR mean > 1.5 (7.7 %) and conjunctival persistence on day 21 (9.6 %) were less often observed as main drivers of classification (Fig. 1) (evidence 2a, b).

**Fig. 2:**
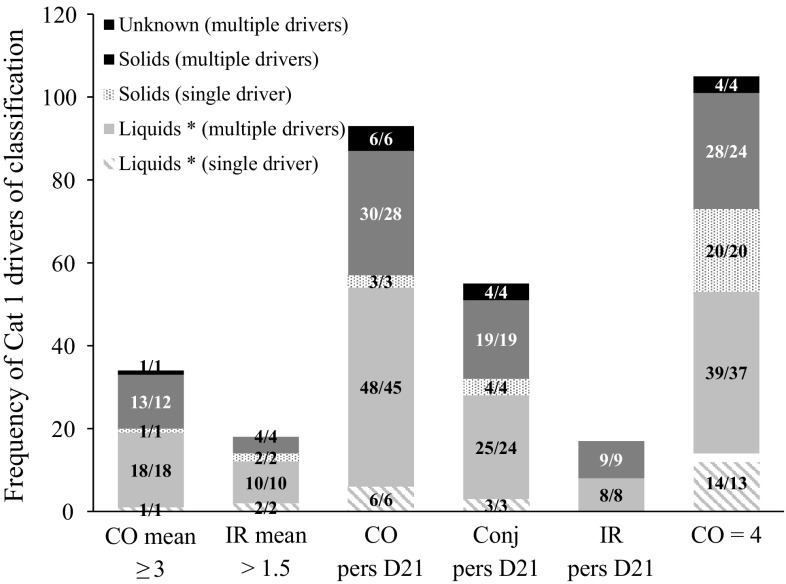
Total frequency of Cat 1 drivers of classification observed in 156 UN GHS/EU CLP Cat 1 studies. The *numbers* in the *bars* correspond to the number of studies/the number of unique test chemicals. The individual drivers appearing in each study were distributed in different groups depending if they occurred alone (single driver) or together with other Cat 1 drivers of classification (multiple drivers) and on the physical state of the chemical as tested (Liquid, Solid or Unknown). Note: CO mean ≥ 3 observed in eight single animal studies (No. 50, 73, 78, 136, 138, 142, 143 and 146) and IR mean > 1.5 observed in five single animal studies (No. 50, 73, 78 and 138) are not counted in the chart because these effects can only be considered drivers of classification when they occur in ≥60 % of the animals in studies with at least three animals. Liquids *: includes solids and liquids tested in solvent

In the previous paragraph the distribution of the Cat 1 studies was presented in terms of the main driver of classification. However, as mentioned earlier, a study can be classified based on more than one driver. The total frequency of each of the six individual drivers of Cat 1 classification was therefore also investigated (Fig. 2). The data presented in Fig. 2 confirm that corneal opacity is the most important endpoint driving the classification of Cat 1 chemicals (evidence 1a, b, c). It is the effect observed most frequently, accounting for 72 % (232/322) of the total frequency of Cat 1 drivers (10.6 % for CO mean ≥ 3, 28.9 % for CO pers D21 and 32.6 % for CO = 4) (Fig. 2). In fact, as shown in the Supplementary Material 1, 92.9 % (145/156) of the Cat 1 studies had sufficient corneal involvement to generate a Cat 1 classification, with 21.8 % being classified based on CO mean ≥ 3, 47.4 % based on CO persistence on day 21 but with CO mean < 3, and 23.7 % based on CO = 4 but with CO mean < 3 and CO reversible by day 21 or unknown (evidence 1a, b, c). The few remaining Cat 1 studies (*n* = 11) were classified based on IR mean > 1.5 only (1.9 %) (evidence 2a) or conjunctival persistence only (5.1 %) (evidence 2b). Figure 2 also demonstrates that the drivers of Cat 1 classification rarely appear on their own (i.e. in the absence of other Cat 1 drivers). The only exception is CO = 4, which was often the only observed effect driving a Cat 1 classification, but this is mostly due to early, ethical termination of the study. Indeed, when CO mean ≥ 3 is the main driver of Cat 1 classification, it is accompanied by CO = 4 in about 91 % (30/33) of the studies and when CO pers D21 is the main driver, it is accompanied by CO = 4 in 53 % (33/62) of the studies. CO = 4 is therefore the driver of Cat 1 classification that was most frequently observed (Fig. 2). Finally, Figs. 1 and 2 show that persistence of conjunctival effects and IR on day 21 appeared in several studies but, while the latter was never observed alone and was never the main driver of classification (evidence 2c), the former was sometimes observed on its own and was also the main driver of classification in these and a few other studies (evidence 2b).

**Fig. 3:**
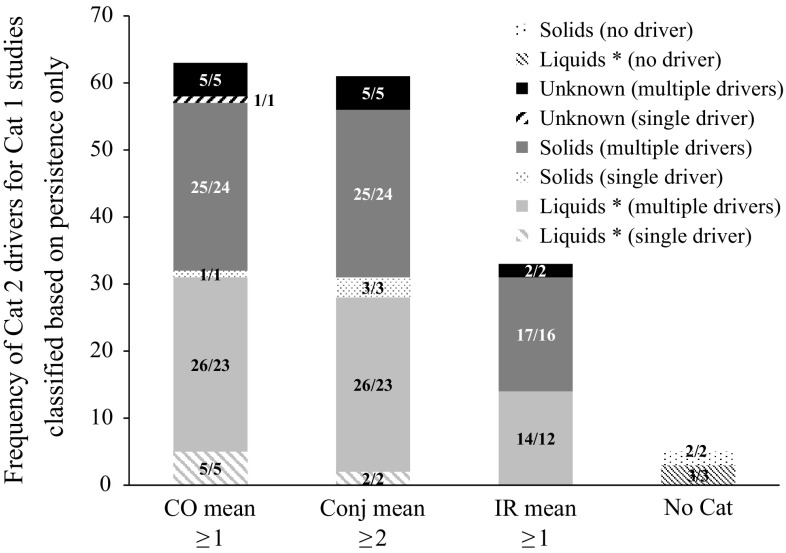
Total frequency of drivers of Cat 2 classification observed in 74 UN GHS/EU CLP Cat 1 studies classified based on persistence of effects only (i.e. with CO mean < 3 and IR mean ≤ 1.5). The *numbers* in the *bars* correspond to the number of studies/the number of unique test chemicals. The individual Cat 2 drivers appearing in each study were distributed in different groups depending if they occurred alone (single driver) or together with other drivers of Cat 2 classification (multiple drivers) and on the physical state of the chemical as tested (Liquid, Solid or Unknown). Five of these 74 Cat 1 studies showed no classifiable effects in days 1–3 (no driver) and are therefore shown as “No Cat”. Note: three studies performed with a single animal that showed CO mean ≥ 3, IR mean > 1.5, CO pers D21, Conj pers D21 and IR pers D21 (studies No. 50, 73 and 78), which were placed in the group CO pers D21, are not included in this figure. Liquids *: includes solids and liquids tested in solvent

Almost 50 % of the Cat 1 studies (*n* = 77) were classified based only on persistence of effects on day 21 (i.e. with CO mean < 3 and IR mean ≤ 1.5). In order to evaluate the character of observed tissue damages in the initial phase of the study after instillation of the substance, the severity scores of days 1–3 in these studies were evaluated in more detail. Three of these studies resulted in CO mean ≥ 3 and IR mean > 1.5, but since these were single animal studies, the classification was driven by persistence and not based on severity. For this reason, they were not considered in this analysis. Figure 3 shows the total frequency of each of the three individual drivers of Cat 2 classification and of absence of any drivers based on the mean scores of days 1–3 for the remaining 74 Cat 1 studies classified based only on persistence of effects. CO mean ≥ 1 and Conj mean ≥ 2 were observed with similar frequencies of 38.9 % (63/162) and 37.7 % (61/162), respectively, while IR mean ≥ 1 was observed in only about half of that (20.4 %, 33/162). It follows that 85.1 % (63/74), 82.4 % (61/74) and 44.6 % (33/74) of these Cat 1 studies showed CO mean ≥ 1, Conj mean ≥ 2 and IR mean ≥ 1, respectively. CO mean ≥ 1 and Conj mean ≥ 2 also occurred alone in 7 and 5 studies, respectively, while IR mean ≥ 1 always occurred together with CO mean ≥ 1 and/or Conj mean ≥ 2. For 6.8 % (*n* = 5) of these Cat 1 studies the mean tissue scores of days 1–3 were below all the cut-offs that trigger classification (i.e. CO mean < 1, IR mean < 1, CR mean < 2 and CC mean < 2, in the majority of the animals). Two of these latter five Cat 1 studies (studies No. 46 and 72) showed delayed effects that persisted until day 21 in a minority of the animals. Most probably, these delayed effects were not directly induced by the test chemical but rather by other phenomena, such as microbial infection (the so-called secondary inflammatory process), differences in animal behaviour and/or absence of post-treatment care (Prinsen 2006, 2014), which raises questions on the relevance of a Cat 1 classification here. The other three (studies No. 83, 92, and 109) showed low-level CO effects from the beginning until the end of the study (day 21) in only a single animal, with all other animals fully reversing before day 21. Also here a Cat 1 classification is highly questionable. Figure 3 shows that persistence of effects on day 21 is mainly driven by effects already appearing on the first 3 days. However, based on the mean scores of days 1–3, these Cat 1 studies cannot be distinguished from Cat 2 studies classified based on CO mean ≥ 1 and/or Conj mean ≥ 2. It is likely that methods that predict immediate severe effects but do not necessarily provide direct information on the persistence or reversibility of effects [for example BCOP and ICE with their current protocols (OECD 2013a, b, respectively)], will not be able to discriminate between these Cat 1 and Cat 2 chemicals. OECD TGs 437 and 438 (OECD 2013a, b) state that both BCOP and ICE generate a high number of false negatives for solids when used to identify chemicals inducing serious eye damage (UN GHS Cat 1). Looking at the validation databases of these two methods (ICCVAM 2007, 2010), it can indeed be confirmed that 25 % (4/16) and 55 % (6/11) of the Cat 1 solids that were tested with BCOP and ICE, respectively, were underpredicted. However, a closer analysis considering the in vivo drivers of classification described in this paper reveals that even higher false-negative rates were obtained with Cat 1 chemicals classified in vivo based on persistence without severity: 46 % (6/13) for BCOP and 69 % (9/13) for ICE. It further shows that all the solids that were underpredicted by these two methods are classified in vivo based only on persistence of effects. It can therefore be concluded that the main limitation of these two methods [with their current protocols (OECD 2013a, b)] in terms of underprediction of Cat 1 chemicals is related to their lack of capacity to accurately predict persistence of effects rather than having a specific limitation for solid chemicals. This simple analysis demonstrates the importance of looking at drivers of classification to better elucidate potential limitations of alternative methods. Although this has not been checked for other in vitro methods accepted or proposed to identify serious eye damage (e.g. FL, STE), the same outcome is expected, i.e. a limitation to predict in vivo persistent effects that occur without enough severity in the first three observation days to generate a Cat 1 classification. The discrimination between reversible and irreversible effects may, however, be feasible using methods that were specifically developed for this purpose, e.g. the EVEIT (Spöler et al. 2007; Frentz et al. 2008) and PorCORA (Piehl et al. 2010, 2011), and/or further development of the protocols of existing methods such as those currently accepted by the OECD.

**Fig. 4:**
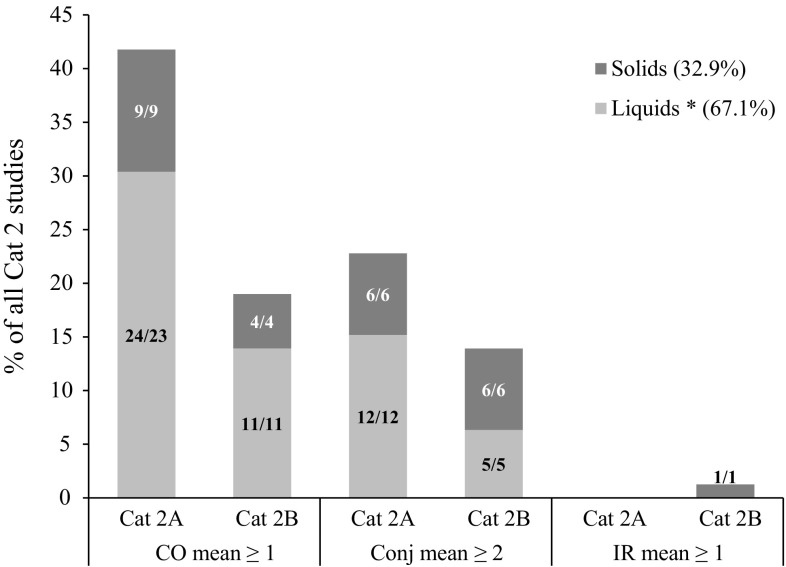
Distribution of 51 UN GHS Cat 2A and 27 UN GHS Cat 2B studies according to their main drivers of classification and the physical state of the chemical as tested. The *numbers* in the *bars* correspond to the number of studies/the number of unique test chemicals. The main driver corresponds to the one showing the largest number of animals fulfilling the classification criterion. If equal number of animals fulfilled the classification criteria for different drivers, the main driver was selected following the order shown on the *x*-axis, with priority decreasing from *left* to *right*. Note: one Cat 2 study is not included in the bar chart because a distinction between Cat 2A and Cat 2B is not possible due to lack of grading on day 7. Liquids *: includes solids and liquids tested in solvent

**Fig. 5:**
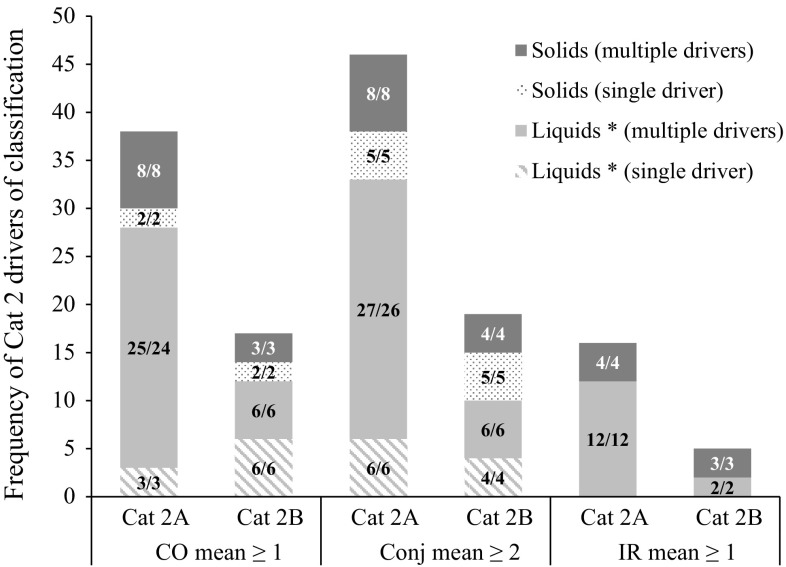
Total frequency of Cat 2 drivers of classification observed in 51 UN GHS Cat 2A and 27 UN GHS Cat 2B studies. The *numbers* in the *bars* correspond to the number of studies/the number of unique test chemicals. The individual drivers appearing in each study were distributed in different groups depending if they occurred alone (single driver) or together with other Cat 2 drivers of classification (multiple drivers) and on the physical state of the chemical as tested (Liquid or Solid). Note: one Cat 2 study is not included in the bar chart because a distinction between Cat 2A and Cat 2B is not possible due to lack of grading on day 7. Liquids *: includes solids and liquids tested in solvent

The studies classified as Cat 2 (*n* = 79: Cat 2A being reversible within 21 days and Cat 2B being reversible within 7 days) were distributed according to three main drivers of classification, namely CO mean ≥ 1, Conj mean ≥ 2 and IR mean ≥ 1. As can be observed in Table 3, 60.8 % of all Cat 2 studies were classified based on CO mean ≥ 1 as the main driver of classification (evidence 4a), followed by 38.0 % with Conj mean ≥ 2 (evidence 4b) and 1.3 % with IR mean ≥ 1 (evidence 2d) as the main drivers. Moreover, in previous analyses it was shown that CC rarely drives the classification of chemicals on its own (about 2 % of the Cat 2 chemicals) (Adriaens et al. 2014; Barroso and Norman 2014) and can therefore be considered unimportant as a driver of classification (evidence 2e). Therefore, studies with Conj mean ≥ 2 as main driver correspond almost always with CR mean ≥ 2 as main driver (evidence 4b). The distribution of Cat 2A (*n* = 51) and Cat 2B (*n* = 27) studies according to their main drivers of classification and subgrouping according to the physical form of the chemicals as tested, is presented in a cumulative bar chart in Fig. 4 (in % relative to the total 79 studies). For one Cat 2 study (No. 217, liquid, main driver Conj mean ≥ 2 but also showing CO mean ≥ 1), it is not possible to distinguish between Cat 2A (reversible within 21 days) and Cat 2B (reversible within 7 days) since no grading was recorded on day 7. Therefore, this study is not shown in Fig. 4. Table 3 also shows the distribution of the physical form of the chemicals as tested within each main driver of classification (in % relative to the total number of studies for each individual driver). A larger percentage of those studies which resulted in Cat 2 classifications were performed with liquids or chemicals tested in solvent (67.1 %) than with solids (32.9 %) (Fig. 4). The majority of the studies was classified based on CO mean ≥ 1 as main driver (60.8 % in total: 41.8 % Cat 2A and 19.0 % Cat 2B) (evidence 4a), followed by Conj mean ≥ 2 (38.0 %: 22.8 % Cat 2A and 13.9 % Cat 2B) (evidence 4b), and only one study (1.3 %) was classified (Cat 2B) based on IR mean ≥ 1 as main driver (Fig. 4) (evidence 2d). The total frequency of each of the three individual drivers of Cat 2 classification is also shown in Fig. 5. Corneal effects were previously shown to be very important in driving Cat 2 classification (Adriaens et al. 2014) and, indeed, 69.6 % (55/79) of the Cat 2 studies (48.1 % (38/79) Cat 2A and 21.5 % (17/79) Cat 2B) showed CO mean ≥ 1 (Fig. 5). However, it is important to note that an even higher number of the Cat 2 studies showed sufficient conjunctival effects (Conj mean ≥ 2) to generate a Cat 2 classification [83.5 % (66/79): 58.2 % (46/79) Cat 2A and 24.1 % (19/79) Cat 2B]. Moreover, while 16.5 % (13/79) of the Cat 2 studies [6.3 % (5/79) Cat 2A and 10.1 % (8/79) Cat 2B] showed CO mean ≥ 1 but Conj mean < 2, almost double the amount [29.1 % (23/79): 16.5 % (13/79) Cat 2A and 12.7 % (10/79) Cat 2B] showed Conj mean ≥ 2 but CO mean < 1 (two of the Cat 2A and one of the Cat 2B studies also showed IR mean ≥ 1) (Fig. 5). It follows that, although CO is an important endpoint driving the classification of Cat 2 chemicals (evidence 4a), conjunctival effects (mainly CR) are even more important (evidence 4b). IR mean ≥ 1, on the other hand, never appears isolated and is also the least frequently observed driver of Cat 2 classification (evidence 2d). The other two Cat 2 drivers CO mean ≥ 1 and Conj mean ≥ 2 appear a considerable number of times on their own (i.e. in the absence of other Cat 2 drivers) (evidence 4a, b), and more often for Cat 2B than for Cat 2A chemicals (Fig. 5). This is in contrast with the Cat 1 chemicals that rarely induce a single, isolated driver of classification. As expected, the stronger the hazard properties of a chemical, the higher the diversity of the induced adverse effects.

**Fig. 6:**
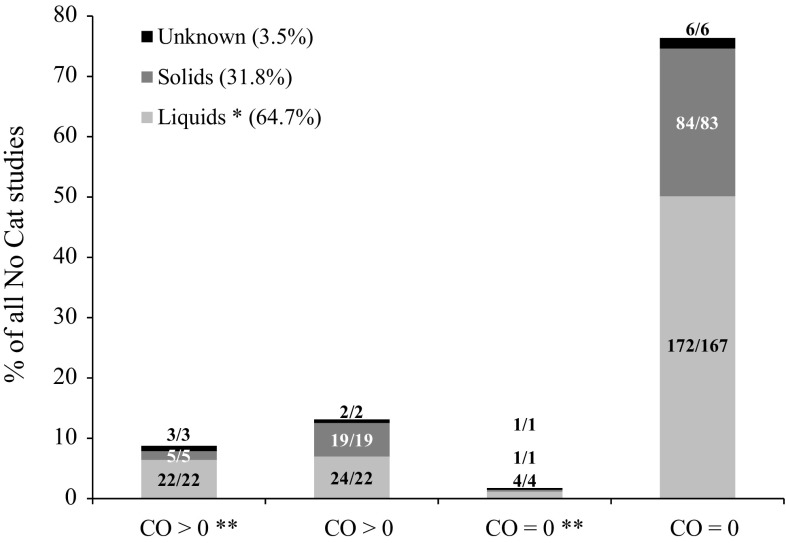
Distribution of 343 UN GHS/EU CLP No Cat studies according the physical state of the chemical as tested and to the following four subgroups: (1) CO > 0 in at least one observation time in at least one animal and at least one animal showing a mean score of days 1–3 above the classification cut-off for at least one endpoint (CO > 0**), (2) CO > 0 in at least one observation time in at least one animal and all animals showing mean scores of days 1–3 below the classification cut-offs for all endpoints (CO > 0), (3) CO = 0 in all observation times in all animals and at least one animal showing a mean score of days 1–3 above the classification cut-off for at least one endpoint (CO = 0**), and (4) CO = 0 in all observation times in all animals and all animals showing mean scores of days 1–3 below the classification cut-offs for all endpoints (CO = 0). The *numbers* in the *bars* correspond to the number of studies/the number of unique test chemicals. Liquids *: includes solids and liquids tested in solvent

The studies with chemicals not requiring classification (No Cat) (*n* = 343) were divided into four subgroups, CO > 0**, CO > 0, CO = 0**, and CO = 0, as explained above. The majority of the No Cat studies was performed with liquids or chemicals tested in solvent (64.7 %). Of the remaining studies, 31.8 % were performed with solids and 3.5 % with a test chemical of unknown physical state. The breakdown of the No Cat studies by subgroup and physical state is presented in Fig. 6 (in % relative to 343 studies) and in Table 3. Table 3 also shows the distribution of the physical form of the chemicals as tested within each subgroup (in % relative to the total number of studies for each subgroup). The majority of the No Cat studies had CO = 0 in all animals (78.1 %) with a small percentage of these studies (1.7 %) showing a conjunctival effect above the classification cut-off in at least one animal (subgroup CO = 0**) (evidence 5). The remaining 21.9 % of the No Cat studies showed CO > 0, with 8.7 % of these studies showing a mean score of days 1–3 for at least one endpoint in at least one animal above the classification cut-off (subgroup CO > 0**) (evidence 5).

In conclusion, it is clear that effects on the cornea (93 % of Cat 1 studies) play a major role in driving Cat 1 classification (evidence 1a, b, c). They are also those of highest concern because they can lead to visual impairment. Furthermore, a substantial proportion of the Cat 1 studies in the DRD shows CO persistence on day 21 (47.4 %, 74/156) but not enough CO severity in the first three observation days to generate a Cat 1 classification (evidence 1b). Both corneal opacity (71 % of Cat 2 studies) and conjunctival effects (84 % of Cat 2 studies) are important in driving Cat 2 classification, but the latter appear to hold a higher weight because they occur alone more often than corneal opacity does (29 vs. 16 %) (evidence 4a, b). Most of the available alternative methods were developed to predict first and foremost immediate corneal effects, although some may also correctly predict irritancy observed only in the conjunctiva in vivo. Nevertheless, in order to achieve full replacement of the Draize eye test, it will be important that persistence of effects is also correctly predicted with alternative methods.

### Variability between repeat Draize eye studies

Since several chemicals in the DRD were tested more than once in independent studies performed by different laboratories, the reproducibility of the Draize eye test could be assessed for these chemicals. The reproducibility of the repeat studies was evaluated in terms of agreement of classifications and/or classification drivers and is summarised in Table 4 for chemicals classified as Cat 1 in at least one study and for chemicals for which the maximum classification obtained in the repeat studies was Cat 2. Table 5 summarises the data for chemicals not requiring classification in all repeat studies. Of note, studies that resulted in a Cat 1 classification based on severity in the first three observation days or CO = 4 were sometimes terminated before day 21. For these chemicals, no information on persistence was available. Absence of information for this driver for a Cat 1 study, which is indicated by “unknown” in the DRD, is therefore no indication of reversibility or persistence of effects.

**Table 4:** Drivers of classification: details for replicate Draize eye test studies

Chemical	ID Nr	*N*	Category 1						Category 2			No Category		SCNM	Agree
			Severity		Persistence D21										
			CO	IR	CO	Conj	IR	CO = 4	CO	Conj	IR	CO > 0	CO = 0		
Pyridine	13, 184	2	A		A			A	B	B	B				No
2-Benzyl-4-chlorophenol	19, 84	2	A		AB	B	B	AB	B	B	B				Yes (≠main driver)
Dibenzoyl-L-tartaric acid	26, 27	2	A‡B					B							Yes
Imidazole	30, 89	2	A‡	A‡	B			A‡	B	B	B				Yes (≠main driver)
Promethazine HCL	31, 32	2	A‡B		B			A‡B							Yes
Triton X-100 (100 %)	36, 37, 590	3		A‡B‡	(C)?			B‡	(C)					C: Cat 1 assumed	Yes (≠main driver)
Quinacrine	44, 96	2		A‡	B		B	A‡B	B	B	B				Yes (≠main driver)
Butoxyethanol	51, 110, 217	3			A	B			ABC	ABC	AB				No
Ethanol (100 %)	57, 171, 201, 602	4			A				AB(D)	ABC	A			D: at least Cat 2A	No
Benzalkonium chloride (1 %)	66, 67	2			AB	AB		B	B	AB	B				Yes
Cetyltrimethyl ammonium bromide (10 %)	69, 70	2			AB			AB	AB	AB	AB				Yes
Sodium lauryl sulphate (10 %)	74, 75, 189	3			AB			AB	ABC	AB	A				No
Sodium oxalate	98, 99	2			AB			AB	AB	AB	AB				Yes
(3-Aminopropyl)triethoxy silane	123, 124	2						A‖B§	B§	B§	B§				Yes
n-Butanol (100 %)	128, 179	2						A§	A§B	A§B	A§				No
Iso-Butanol	175, 589	2			(B)?				A(B)	A(B)				B: Cat 1 assumed	No
Gamma-Butyrolactone	173, 174	2							AB	AB	B				Yes
Methyl acetate	176, 225	2							AB	AB	B				2A/2B
Methyl *N*,*N*,*N*-trimethyl-4… (30 %, aqueous)^a^	178, 205	2							A	AB	A				Yes (≠main driver)
n-Octanol	181, 228	2							AB	AB	AB				2A/2B
Tetra aminopyrimidine sulphate	198, 317	2							A			B			No
Triton X-100 (5 %)	207, 647	2							A(B)	A(B)				B: Cat 2A	Yes (≠main driver)
Toluene	321, 627	2								(B)			A**	B: at least Cat 2	No
o-Phenylenediamine	614, 615	2							(AB)	(A)	(A)			A and B: at least Cat 2A	Uncertain

*N* corresponds with the number of repeated studies. Repeated studies are assigned with different uppercase letters (e.g. A). The appearance of a capital in a given column indicates that the specific driver of classification is (or is expected to be) met. Underlined capitals (e.g. A) indicate the main driver of classification. Upper case letters with ‡ (e.g. A‡) indicate studies classified Cat 1 based on severity and terminated before day 21, for which it is therefore unknown if tissue effects are persistent on day 21. Upper case letters with § (e.g. A§) indicate studies classified Cat 1 based on CO = 4 and terminated before day 21, for which it is therefore unknown if tissue effects are persistent on day 21. The upper case letter with ‖ (A‖), indicates a study classified Cat 1 based on CO = 4 and terminated on day 1, for which the severity and persistence of tissue effects are therefore unknown. Upper case letters between brackets (e.g. (B)) indicate studies for which study criteria allowing an unambiguous classification were not met (study criteria not met, SCNM) because the study was terminated before day 21 without full reversibility of all endpoints and in the absence of any other effects driving a Cat 1 classification. Upper case letters between brackets with a question mark (e.g. (C)?) indicate that CO persistence was assumed but cannot be confirmed because the study was terminated before day 21. Upper case letters with ** (e.g. A**) indicate at least one animal with a mean score of days 1–3 above the classification cut-off for at least one endpoint

CO, corneal opacity; IR, iritis; Conj, conjunctival redness (CR) and/or conjunctival chemosis (CC)

^a^Methyl *N*,*N*,*N*-trimethyl-4-[(4,7,7-trimethyl-3-oxobicyclo[2.2.1]hept-2-ylidene)methyl]anilinium sulphate (30 %, aqueous)

**Table 5:** Distribution of chemicals not requiring classification by subgroups: details for replicate Draize eye test studies

Chemical	ID Nr	N	No Category				SCNM	Agree
			CO > 0**	CO > 0	CO = 0**	CO = 0		
Methyl amyl ketone	257, 284	2	A	B				Yes (≠group)
Phosphoric acid, tributyl ester	260, 446	2	A			B		Yes (≠group)
1,2,3-Trichloropropane	275, 327	2		A		B		Yes (≠group)
Methyl iso-butyl ketone	285, 286	2		AB				Yes
Triethanolamine (100 %)	292, 468	2		A		B		Yes (≠group)
Sodium lauryl sulphate (1 %)	296, 297	2		AB				Yes
Xylene	322, 483	2			A	B		Yes (≠group)
3-Phenoxy benzaldehyde (100 %)	358, 359	2				AB		Yes
Gamma-Glycidyloxypropyltrimethoxy silane	391, 392	2				AB		Yes
Gamma-Mercaptopropyl trimethoxy silane (100 %)	393, 394	2				AB		Yes
Glycerol (100 %)	400, 401	2				AB		Yes
Kronitex TXP	425, 426	2				AB		Yes
Perfluoro-n-hexane	443, 444	2				AB		Yes
Polyethylene glycol 400 (100 %)	448, 449	2				AB		Yes
Tricresyl phosphate	465, 466	2				AB		Yes
Tween 20	476, 477	2				AB		Yes
PEG-40 hydrogenated castor oil	501, 502	2				AB		Yes
Tetrabromobisphenol A	574, 677	2				A(B)	B: No Cat	Yes

*N* corresponds with the number of repeated studies. Repeated studies are assigned with different capitals. The appearance of a capital in a given column indicates the subgroup of the study. Upper case letters between brackets (e.g. (B)) indicate studies for which study criteria allowing an unambiguous classification were not met (study criteria not met, SCNM) because the study was terminated before day 21 without full reversibility of all endpoints

** At least one animal with a mean score of days 1–3 above the classification cut-off for at least one endpoint

Sixteen of the chemicals with repeat studies resulted in a Cat 1 classification in at least one study (Table 4). The repeat studies were reproducible for 37.5 % (6/16) of the chemicals, resulting in the same UN GHS classification based on the same main driver of classification. For another four chemicals (25 %) the repeat studies also resulted in a Cat 1 classification but based on a different main driver of classification (severity versus persistence). The remaining six chemicals (37.5 %) resulted in a different classification in the repeat studies (Cat 1 vs. Cat 2). Therefore, these 16 chemicals showed an overall concordance of classifications of only 62.5 % (10/16) (Table 4). Two of the six chemicals with discordant classifications, namely Butoxyethanol and 10 % Sodium lauryl sulphate, showed persistence of effects without severity in the first three observation days in two independent studies (Butoxyethanol: studies No. 51 and 110; 10 % Sodium lauryl sulphate: studies No. 74 and 75), while the same effects fully reversed within 21 days in a third study (No. 217 and No. 189, respectively). One of the Cat 1 studies obtained with Butoxyethanol (study No. 51) showed persistence of CO (CO ≤ 2) in a minority of the animals (1 or 2 out of 6) (evidence 6a), while the other (study No. 110) showed CR = 1 on day 21 in 2 of 3 animals and CC = 1 on day 21 in the third animal (evidence 7a). For another chemical (iso-Butanol), one of the available studies (No. 589) was terminated on day 14 with a CO = 3 in one out of three animals while in the two other animals CO fully reversed by day 1 and day 14. Although the persistence of CO on day 21 in the first animal cannot be confirmed, this is assumed to be the case due to the high CO score on day 14. This SCNM study was therefore assumed to be Cat 1 for the purpose of this analysis. The repeat study (No. 175) resulted in a Cat 2A classification based on CO mean ≥ 1 and Conj mean ≥ 2 in all tested animals. In three cases of inconsistent classification (including the one described above), the Cat 1 classifications were driven by a single animal (No. 57, 128 and 175) (evidence 6a). One of these studies (100 % Ethanol: study No. 57) resulted in CO persistence in one out of six animals, with the mean CO scores of days 1–3 falling between the Cat 2 and the Cat 1 classification cut-offs, while in at least two other studies (No. 171 and 201) the effects fully reversed by day 21 in all animals resulting in a Cat 2A classification. For another one of these three chemicals (n-Butanol), CO = 4 was observed in one out of three animals in the Cat 1 study (No. 128), whereas the repeat study (No. 179) resulted in a Cat 2A classification based on CO mean ≥ 1 and Conj mean ≥ 2 in four out of four animals. Only one of the six chemicals with a discordant classification (Pyridine) showed Cat 1 severity (CO mean ≥ 3) in one of the studies (No. 13) and Cat 2 severity (e.g. CO mean ≥ 1) in the second study (No. 184). Thus, the difference between repeat studies with non-concordant classification (Cat 1 vs. Cat 2) is mostly related to the presence of persistent and/or CO = 4 effects in a minority of the animals (evidence 6a), or the presence of CR and/or CC = 1 on day 21 in the Cat 1 studies versus the absence of such effects in the Cat 2 studies, with no meaningful differences being observed in the scores of the first three observation days (evidence 7a). These results support an earlier suggestion by Adriaens et al. (2014) to revise some of the current UN GHS and EU CLP decision criteria for the classification of chemicals as inducing serious eye damage (Cat 1) (see also chapter “Critical Review of UN GHS/EU CLP classification criteria” below).

For seven of the chemicals with repeat studies, the highest classification obtained was Cat 2 (Table 4). Only one of these seven chemicals with a Cat 2 classification in at least one study (14.3 %), was classified consistently (Cat 2A) and with the same main driver of classification (CO mean ≥ 1) across studies (No. 173 and 174). Two chemicals were classified consistently in two independent studies but based on a different driver (No. 178 and 205; No. 207 and 647). Two other chemicals showed the same main driver of classification in two independent studies but a difference in the persistence of the effects on day 7, with one study fully reversing by day 7 (Cat 2B) (No. 225 and 228) while the other did not (Cat 2A) (No. 176 and 181). Two chemicals resulted in a different categorisation in two independent studies, with one study not requiring classification (No. 317 and 321) and another study resulting in a Cat 2A classification based on CO mean ≥ 1 (No. 198) or a Cat 2 or higher classification based on Conj mean ≥ 2 (No. 627). The overall concordance of classifications for the chemicals having Cat 2 as the highest classification obtained is therefore 71.4 % (5/7) (Table 4), when considering Cat 2A and Cat 2B as concordant classifications (as in EU where these two optional subcategories were not implemented). If Cat 2A and Cat 2B are considered as different classifications, the concordance decreases to 42.9 % (3/7). Finally, one chemical has two SCNM studies that were terminated before day 21 without full reversibility of effects (No. 614 and 615). Both studies resulted in at least a Cat 2A classification, but no conclusion can be made in terms of reproducibility without information on the persistence/reversibility of the effects on day 21. Indeed, it cannot be precluded that one of the two studies would have shown persistent effects and would have been classified as Cat 1 should the study have been completed. These two studies were therefore not considered in the analysis of reproducibility between repeat Draize eye test studies presented here.

Eighteen chemicals showed a concordant No Cat outcome in two repeat studies (Table 5). However, the reproducibility of these repeat studies in terms of their subgroup was only 72.2 % (13/18) (Table 5). Two chemicals have two repeat studies both belonging to the CO > 0 subgroup (studies No. 285 and 286 and studies No. 296 and 297). For another 11 chemicals, both repeat studies resulted in CO scores equal to 0 in all animals and all observed time points and none of the tested animals showed a mean score of days 1–3 for any of the endpoints above their classification cut-offs (CO = 0 subgroup). Since these chemicals do not induce any significant effects on the eye, observation of no effects is expected to be highly reproducible. Still, four other chemicals resulted in CO = 0 in one study (No. 446, 327, 468 and 483) and in CO > 0** (No. 260), CO > 0 (No. 275 and 292) or CO = 0** (No. 322) in another study. The studies marked with ** are considered borderline since they show at least one animal fulfilling at least one of the classification criteria, but not enough animals fulfilling the same criteria to actually generate a classification. In many of these cases, however, the difference between Cat 2 or No Cat lies in a single score in a single animal, so they do differ significantly from studies in the CO = 0 subgroup. Finally, one chemical has two repeat studies, one resulting in CO > 0** (study No. 257) and the other in CO > 0 (study No. 284). These two studies are, however, very similar since both show CO > 0 on at least one observation day in three out of four animals. Moreover, while one showed mean CC scores of days 1–3 of 2 (**), 0.33, 0 and 0 (study No. 257), the other showed mean CC scores of days 1–3 of 1.33, 1.33, 1.33 and 1 (study No. 284), so the differences between the two are minimal.

Overall, for the chemicals requiring classification in at least one of multiple studies, the observed concordance of UN GHS classifications when considering a unified Cat 2 classification is 65.2 % (15/23). If Cat 2A and Cat 2B are considered as different classifications, the observed concordance of UN GHS classifications is 56.5 % (13/23). Concordance of the same main driver of classification occurs for 39.1 % (9/23) of the chemicals. The statistical resampling analysis performed by Adriaens et al. (2014), demonstrated an overall probability of at least 11 % that chemicals classified as Cat 1 by the Draize eye test could be equally identified as Cat 2 and of about 12 % for Cat 2 chemicals to be equally identified as No Cat. These proportions of misclassifications reflect the within-test variability of the Draize eye test only. Although there are only a limited number of repeat studies available in the DRD, there is evidence that the reproducibility of the Draize eye test reduces substantially when its between-laboratory reproducibility is taken into account. Thus, in the current data set, 37.5 % (6/16) of the chemicals with at least one Cat 1 study could be equally identified as Cat 2 and 28.6 % (2/7) of the Cat 2 chemicals could be equally identified as No Cat. This confirms earlier findings of Weil and Scala (1971) and Cormier et al. (1996). These studies already reported a high between-laboratory variability of the Draize eye test method, although a weighted sum score of ocular lesions that gives more weight to corneal injury, was used in the analyses instead of the individual ocular tissue scores and UN GHS classification considered in the current analyses. It is therefore important not to disregard the substantial variability of responses between Draize eye test studies and even between animals in the same study, as reported here and in previous studies (Weil and Scala 1971; Cormier et al. 1996; Prinsen 2006, 2014; Adriaens et al. 2014), when discussing the validity, regulatory acceptance and use of alternative test methods and testing strategies for serious eye damage/eye irritation. Such variability can be caused by differences in animal behaviour, differences in exposure times, mechanical damage induced by solid chemicals that are not readily washed off the eye, secondary inflammatory processes, absence (or presence) of post-treatment care and/or subjective scoring (especially for those chemicals causing effects near the thresholds for classification) (Prinsen 2014). Moreover, if the variability and quality of the in vivo data are not carefully considered when selecting reference test chemicals for the validation of alternative in vitro methods, the chance of success of such studies may substantially decrease.

### Analyses of studies classified Cat 1 based on persistence only

About 47 % of the Cat 1 studies were classified based on persistence in absence of enough CO and IR severity in the first three observation days (i.e. CO mean < 3 and IR mean ≤ 1.5). In order to better understand persistence of tissue effects, tissue scores were compared between (1) studies showing persistence of any given effect on day 21 in the majority of the animals (i.e. in ≥60 % of the animals in accordance with the UN GHS/EU CLP classification criteria for effects appearing during the first three observation days: 2 or more out of 3, 3 or more out of 4, 3 or more out of 5, or 4 or more out of 6) and (2) studies showing persistence of any given effect on day 21 in the minority of the animals (i.e. in <60 % of the animals: 1 out of 3, 2 or less out of 4, 2 or less out of 5, or 3 or less out of 6). The relation between the tissue scores of these two groups of studies and the tissue scores observed in Cat 2A studies was also explored. These comparisons were performed for each tissue separately and single animal studies were not included in the analyses.

**Fig. 7:**
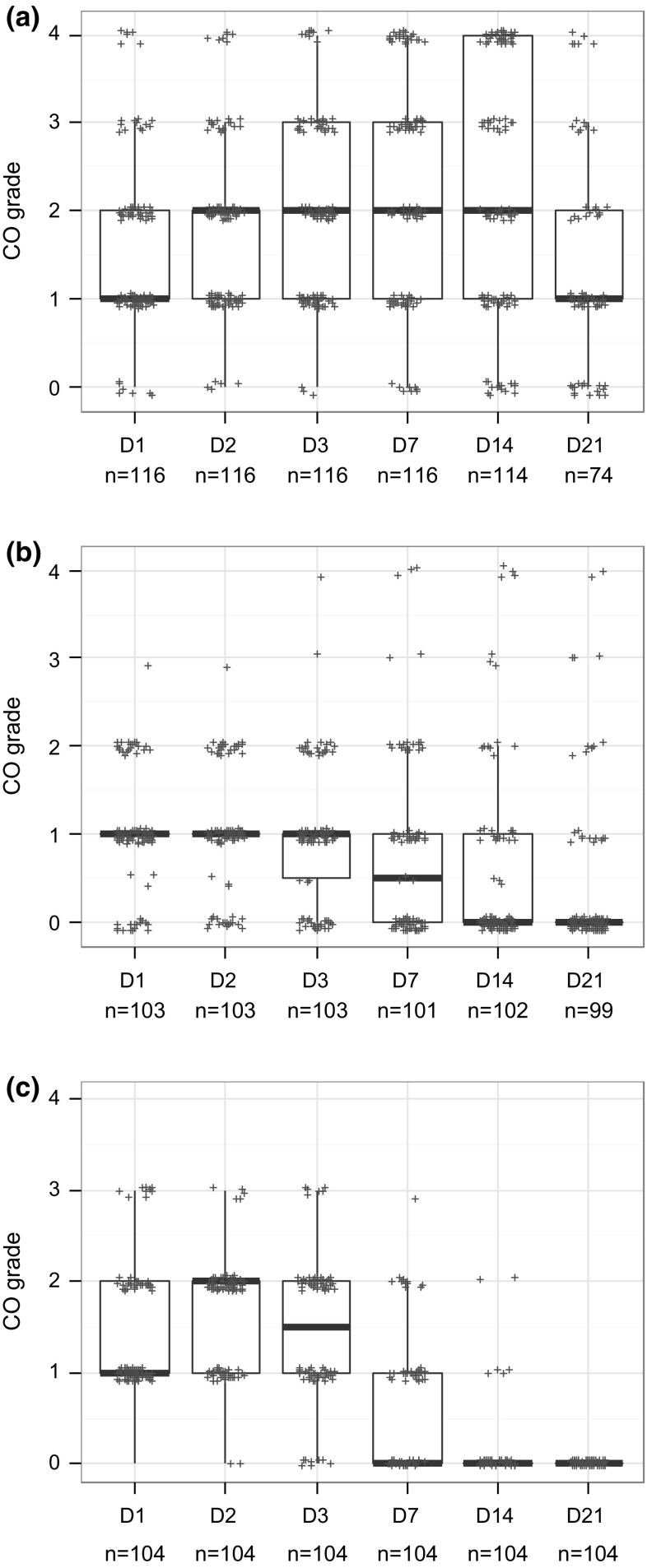
*Boxplots* presenting the distribution of individual animal CO grades at 1, 2, 3, 7, 14 and 21 days after instillation of the test chemical for **a** Cat 1 studies showing CO persistence in the majority of the animals but with CO mean < 3 and IR mean ≤ 1.5 in the majority of the animals (32 studies with 116 animals), **b** Cat 1 studies showing CO persistence in the minority of the animals but with CO mean < 3 and IR mean ≤ 1.5 in the majority of the animals (25 studies with 104 animals), and **c** Cat 2A studies showing persistence of CO on day 7 in at least one animal (28 studies with 104 animals). The *symbols* (+) present individual observations, the thick horizontal lines correspond to the medians of all observations, and the *whiskers* correspond to the smallest and largest observation that fall within a distance of 1.5 times the length of the *box* (Interquartile Range, IQR) from the lower quartile (*bottom side* of the *box,* 25th percentile) and upper quartile (*upper side* of the *box,* 75th percentile), respectively

Figure 7 illustrates the distribution of CO scores as a function of observation time for Cat 1 studies showing CO persistence in the majority of the animals (boxplot A), Cat 1 studies showing CO persistence in the minority of the animals (boxplot B), and Cat 2A studies with CO persistence on day 7 in at least one animal (boxplot C). CO persistence in the majority of the animals was observed in 32 Cat 1 studies with 116 animals. At least 50 % of the animals in these studies showed CO scores equal to or greater than 2 from day 2 until day 14 (evidence 8a). The difference in distribution observed on day 21 as compared to day 14 is mostly explained by the fact that 14 of these 32 studies were terminated before day 21, with 42 of the 45 animals included in these studies showing CO scores equal to or greater than 2 on day 14. In fact, 27 of these 42 animals actually showed a CO = 4 on day 14 and another 12 had CO = 3 on that last observation day. It is highly unlikely that such high scores would decrease by day 21, and therefore, if the 14 studies terminated on day 14 had been fully completed until day 21, the distribution of scores on day 21 would have probably more closely resembled the distribution observed on day 14 (evidence 8a). In clear contrast, the CO scores of the 25 Cat 1 studies with CO persistence in the minority of the animals decreased with time, with 50 % of the animals already having CO scores ≤ 1 on days 1, 2, and 3 (evidence 6b). By day 14 at least 50 % of the animals had CO = 0 and on day 21, CO = 0 was observed in 74 % (76/103) of the animals. For 19 studies (No. 55, 57, 58, 59, 62, 65, 66, 76, 80, 83, 92, 93, 97, 102, 103, 107, 109, 118, and 119), persistent effects on the cornea appeared in only 1 out of 3, 4, or 6 animals, whereas all other animals had low scores that reversed to 0 by day 21 or earlier (evidence 6a). Three other studies (No. 46, 72, and 98) were terminated on day 14, each with a single animal showing CO > 0 on this last observation day. CO persistence was assumed in all the three cases due to delayed CO = 4 appearing on day 3 in study No. 98 and on day 7 in studies No. 46 and 72 (Fig. 7b), which persisted until day 14 on those single animals. In contrast, all other animals showed full reversibility of CO scores to 0 by day 14 or earlier. In total, five animals in five different studies (No. 46, 72, 80, 98, and 107) showed delayed effects with a CO = 4 appearing on day 3, 7, 14, or 21 (Fig. 7b), while all the other animals consistently showed CO ≤ 2 (several times equal to 0) throughout the entire study that fully reversed on day 21 or earlier (Supplementary Material 1) (evidence 6a). In general, the CO scores of the Cat 1 chemicals classified based on CO persistence in the minority of the animals (Fig. 7b) have a similar distribution as those of the Cat 2A chemicals showing CO persistence on day 7 (Fig. 7c) (evidence 6b). In fact, based on the CO scores observed over the first 3 days, it is not possible to distinguish the Cat 1 studies with CO persistence in the minority of the animals (Fig. 7b) from the Cat 2A studies (Fig. 7c). CO persistence in a minority of the animals should therefore not be used to drive a Cat 1 classification, nor should isolated extreme effects (CO = 4) appearing late in the study, as these are most probably not related to the test chemical itself.

**Fig. 8:**
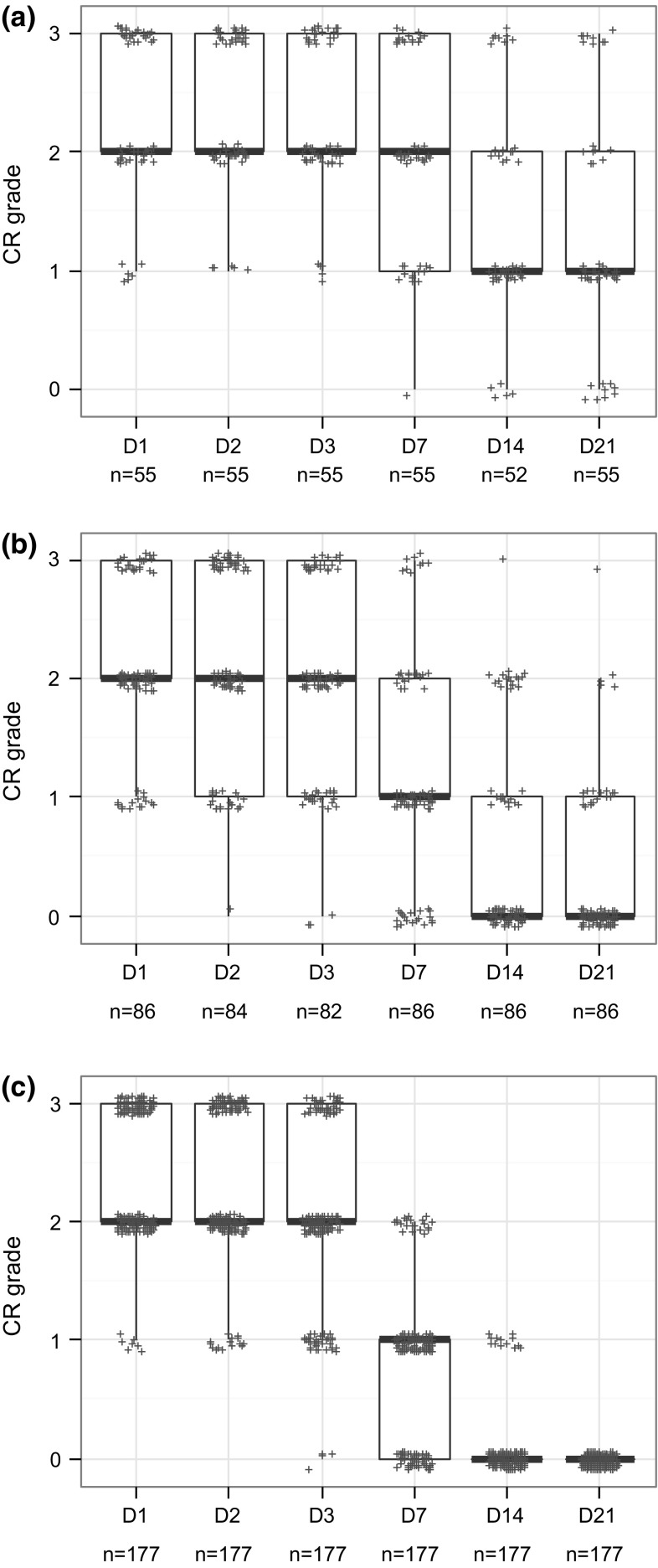
*Boxplots* presenting the distribution of individual animal CR grades at 1, 2, 3, 7, 14 and 21 days after instillation of the test chemical for **a** Cat 1 studies showing CR persistence in the majority of the animals but with CO mean < 3 and IR mean ≤ 1.5 in the majority of the animals (16 studies with 55 animals), **b** Cat 1 studies showing CR persistence in the minority of the animals but with CO mean < 3 and IR mean ≤ 1.5 in the majority of the animals (20 studies with 86 animals), and **c** Cat 2A studies showing persistence of CR on day 7 in at least one animal (49 studies with 177 animals). The *symbols* (+) present individual observations, the thick horizontal lines correspond to the medians of all observations, and the *whiskers* correspond to the smallest and largest observation that fall within a distance of 1.5 times the length of the *box* (Interquartile Range, IQR) from the lower quartile (*bottom side* of the *box,* 25th percentile) and upper quartile (*upper side* of the *box,* 75th percentile), respectively

Figure 8 shows the distribution of the CR scores as a function of observation time for Cat 1 studies showing CR persistence in the majority of the animals (boxplot A), Cat 1 studies showing CR persistence in the minority of the animals (boxplot B), and Cat 2A studies with CR persistence on day 7 in at least one animal (boxplot C). CR persistence in the majority of the animals was observed in 16 Cat 1 studies with 55 animals. In contrast with CO, the CR scores of these animals appear to decrease with time (Figs. 7a, 8a) (evidence 8b). CR persistence in the minority of the animals was in turn observed in 20 Cat 1 studies with 86 animals. About 31 % (17/55) of the animals from Cat 1 studies with CR persistence on day 21 in the majority of the animals had CR ≥ 2 on day 21, while 51 % (28/55) had CR = 1 (Fig. 8a). In contrast, only 7 % (6/86) of the animals in the Cat 1 studies with CR persistence in the minority of the animals had CR ≥ 2 on day 21, with 20 % (17/86) having CR = 1 (Fig. 8b). Of note, the majority of the animals with CR ≥ 2 on day 21 also had CO ≥ 1 at the end of the study [83 %, (14 + 5)/(17 + 6)]. CR ≥ 2 on day 21 is therefore almost always associated with some degree of corneal opacity (evidence 7b). The only exceptions here are studies No. 114 and 117. Study No. 114 resulted in CR = 2 (with CC = 1 and IR = 1) on day 21 in one animal, whereas the CO of this animal and all tissue scores of the two other animals reversed to 0 latest by day 15. In study No. 117, two out of six animals had CR = 3 on day 21 without any additional tissue effects, but the third animal showed full reversibility of all tissue effects (to score 0) by day 21 or earlier. Study No. 84 also includes one animal that showed CR = 2 and CO = 0 on day 21, but the same study includes five other animals that showed CO persistence on day 21 (scores of 1, 2, 2, 3 and 4), coupled with CR ≥ 2 in the four animals with highest CO scores. Studies No. 55, 62, 65, 66, 83, 93, 94, 97, 102, 112, and 113 showed CR = 1 on day 21 in a single animal, study No. 100 in two out of six animals, and studies No. 54 and 109 in two out of four animals. CO persistence on day 21 was also observed in 14 of these 17 animals (82 %) while, for the other three animals, CR = 1 on day 21 was observed in the absence of any other persistent effects (studies No. 109, 112, and 113). Study No. 109 is, however, classified based on CO persistence on day 21 in another animal. Although less pronounced, CO persistence was also observed in 54 % (15/28) of the animals with CR = 1 on day 21 in the studies showing CR persistence in the majority of the animals. Based on these data, it can be concluded that studies showing persistent CR on day 21 are generally also classified as Cat 1 based on CO persistence (evidence 7b). Furthermore, no important difference in the distribution of the CR scores can be observed between Cat 1 studies with CR persistence in the minority of the animals (Fig. 8b) and Cat 2A studies with CR persistence on day 7 (Fig. 8c) (evidence 6c). CR persistence in a minority of the animals should therefore not be used to drive a Cat 1 classification. In fact, it is not possible to distinguish the Cat 1 studies (Fig. 8a, b) from the Cat 2A studies with CR persistence on day 7 (Fig. 8c) based on the distribution of the CR scores from the first three observation days, which demonstrates that CR is not useful to identify Cat 1 chemicals, at least when it comes to effects observed in the first 3 days after instillation.

**Fig. 9:**
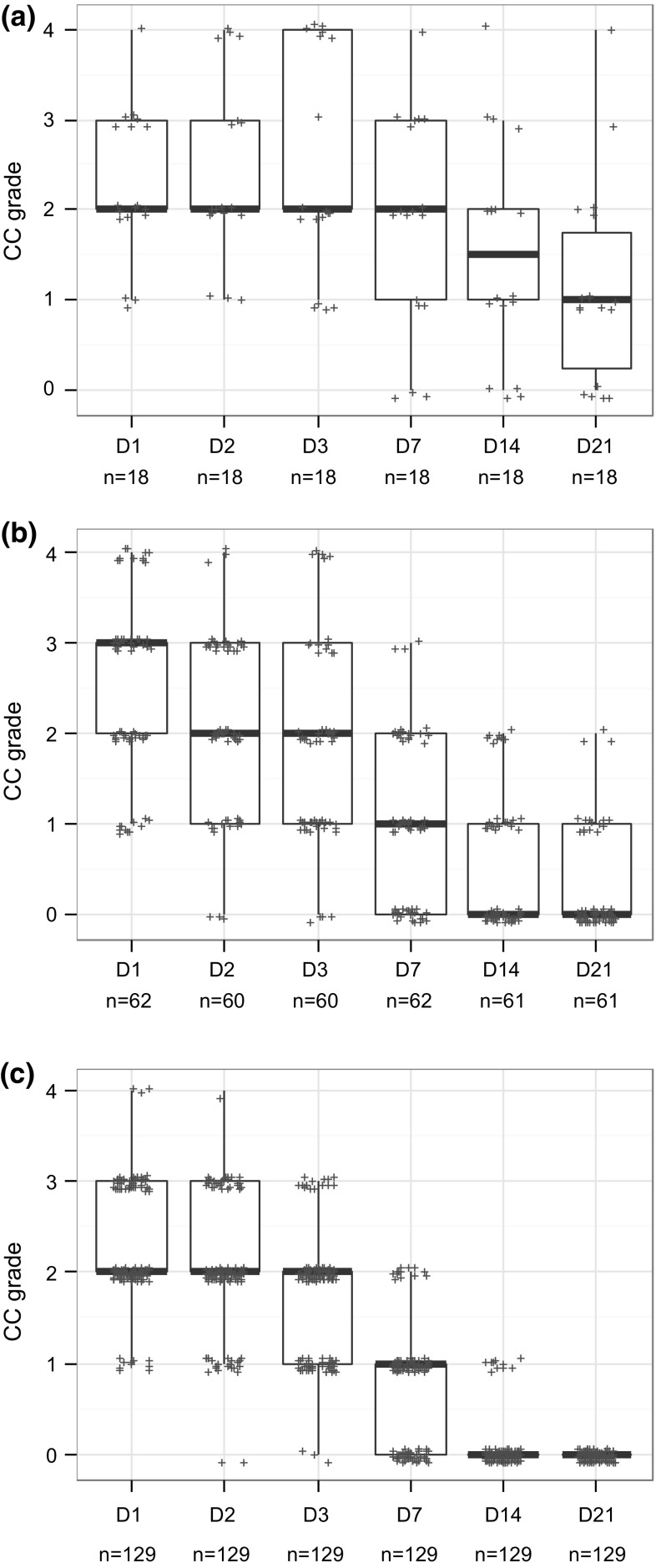
*Boxplots* presenting the distribution of individual animal CC grades at 1, 2, 3, 7, 14 and 21 days after instillation of the test chemical for **a** Cat 1 studies showing CC persistence in the majority of the animals but with CO mean < 3 and IR mean ≤ 1.5 in the majority of the animals (4 studies with 18 animals), **b** Cat 1 studies showing CC persistence in the minority of the animals but with CO mean < 3 and IR mean ≤ 1.5 in the majority of the animals (15 studies with 62 animals), and **c** Cat 2A studies showing persistence of CC on day 7 in at least one animal (34 studies with 129 animals). The *symbols* (+) present individual observations, the thick horizontal lines correspond to the medians of all observations, and the *whiskers* correspond to the smallest and largest observation that fall within a distance of 1.5 times the length of the *box* (Interquartile Range, IQR) from the lower quartile (*bottom side* of the *box,* 25th percentile) and upper quartile (*upper side* of the *box,* 75th percentile), respectively

The distributions of the CC scores as a function of observation time for Cat 1 studies showing CC persistence in the majority of the animals (boxplot A), Cat 1 studies showing CC persistence in the minority of the animals (boxplot B), and Cat 2A studies with CC persistence on day 7 in at least one animal (boxplot C) are shown in Fig. 9. Figure 9 reveals that CC persistence on day 21 occurs less often then CO and CR persistence (evidence 2b). Indeed, CC persistence in the majority of the animals was observed in only four studies (studies No. 81, 84, 105, and 120), as compared to 16 and 32 studies with CR and CO persistence in the majority of the animals, respectively. Similar to CR, the CC scores of the 18 animals in the studies showing CC persistence in the majority of the animals visibly decrease with time (Fig. 9a) (evidence 8c). This is in clear contrast with CO (Fig. 7a) (evidence 8a). CC persistence in the minority of the animals was observed in 15 studies (studies No. 59, 66, 67, 80, 88, 93, 94, 95, 103, 107, 108, 109, 110, 114, and 119), as compared to 20 and 25 studies with CR and CO persistence in the minority of the animals, respectively. The percentages of animals showing CC ≥ 2 or CC = 1 on day 21 are, however, similar to those observed for CR, being 28 % (5/18) and 44 % (8/18), respectively, in the Cat 1 studies with CC persistence on day 21 in the majority of the animals (Fig. 9a), and 5 % (3/62) and 21 % (13/62), respectively, in the Cat 1 studies with CC persistence on day 21 in the minority of the animals (Fig. 9b). Most important, the majority of the animals with CC > 0 on day 21 also had CO ≥ 1 at the end of the study (evidence 7c). Indeed, 25 out of 29 animals (86 %) with CC > 0 on day 21 also showed CO persistence at the end of the study (12 out of 13 and 13 out of 16 in the majority and minority groups, respectively) (Fig. 9a, b). The four animals with persistent CC but CO = 0 on day 21 (studies No. 84, 108, 110, and 114) showed CC = 1 at the end of the study. In studies No. 84 and 108, however, several other animals showed CO persistence on day 21, thus driving a Cat 1 classification on their own. For the other two animals from studies No. 110 and 114, the CC = 1 on day 21 was observed in the absence of CO persistence in any of the animals from those studies. It should, however, be noted that CC fully reversed to 0 by day 7 in the other two animals of study No. 114 and by days 14 and 21 in the other two animals of study No. 110. As with CO and CR, no important difference in the distribution of the CC scores can be observed between Cat 1 studies with CC persistence in the minority of the animals (Fig. 9b) and Cat 2A studies with CC persistence on day 7 (Fig. 9c) (evidence 6d) and therefore, CC persistence in a minority of the animals should not be used to drive a Cat 1 classification. Also similar to CR, it is not possible to distinguish the Cat 1 studies (Fig. 9a, b) from the Cat 2A studies with CC persistence on day 7 (Fig. 9c) based on the distribution of the CC scores of days 1–3.

In conclusion, for Cat 1 studies classified based only on persistence of effects, CO persistence on day 21 is the most important driver of classification as it often occurs without CR and CC persistence (evidence 1b). CR = 0 or CC = 0 on day 21 occur in 41 % (33/81) and 70 % (57/81) of the animals showing CO persistence on day 21. CR and CC persistence on the other hand almost always occur together with CO persistence on day 21 (71 % (48/68) and 86 % (25/29) of the animals for CR and CC, respectively) and, when they do not, the scores on day 21 are usually low (i.e. score 1) (evidence 7a, b, c). Interestingly, the presence of CR or CC persistence in parallel with CO persistence on day 21 is about 10 % higher for the Cat 1 studies showing CO persistence in the minority of the animals (67 % of the animals for CR and 38 % for CC) than for the Cat 1 studies showing CO persistence in the majority of the animals (56 % of the animals for CR and 26 % for CC). This supports the view that in many of the studies showing effects on the minority of the animals, these are not directly induced by the test chemical but rather by other phenomena like differences in animal behaviour, mechanical damage, and/or a secondary inflammatory process (Prinsen 2014), which are expected to affect all tissues more indiscriminately. Overall, there were only two out of 14 studies (14 %) where a CR ≥ 2 on day 21 in one animal did not occur together with CO persistence in that or any other animal (studies No. 114 and 117) (evidence 7b). This was never observed for CC (evidence 7c). Furthermore, a gradual decrease in CR and CC scores can be observed as a function of time (Figs. 8, 9) (evidence 6c, d and 8b, c), which does not occur with CO scores obtained in Cat 1 studies with CO persistence in the majority of the animals (Fig. 7a) (evidence 8a). This indicates that conjunctival effects are generally reversible by nature and thus suggests that they are not important and should not be used for driving Cat 1 classifications, especially when the score on day 21 is equal to 1 (evidence 7a). Accordingly, the US EPA classification system considers CR and CC scores of less than 2 as fully cleared (US EPA 1998, 2011; ICCVAM 2010). Moreover, conjunctival scores higher than 1 on day 21 are almost always accompanied by CO persistence on day 21 and are therefore unimportant from a classification perspective (evidence 7b, c).

### Critical review of UN GHS/EU CLP classification criteria

According to the UN GHS classification criteria, a Cat 1 classification can be triggered based on tissue effects observed in a single animal only. For example, some chemicals considered as Cat 1 are not classified based on severity (days 1–3) but based on one animal with low-level persistent effects or a single animal showing delayed effects, whereas all other animals have low scores that reverse to 0 by day 21 or earlier. Some examples of this situation were already mentioned above which, unsurprisingly, were linked to variability of responses and classifications between repeat studies (evidence 6a). Other examples like this but for which only a single study is available are 1,4-dimethylbenzene (No. 46) and 1-naphthalene acetic acid (No. 80). Both were tested in six animals and resulted in a delayed effect in one single animal (CO = 4 appearing on day 7 and day 14, respectively), whereas all tissue scores reversed to 0 by day 21 or earlier in all other animals. If the single animal with persistent effects for 1-naphthalene acetic acid was not taken into account, this chemical would be classified as Cat 2A. The Cat 1 classification of 1,4-dimethylbenzene is even more questionable since this chemical would not require classification (subgroup CO > 0**) if the one animal showing a delayed effect was not taken into account. Four other studies included in the DRD (No. 72, 83, 92 and 109) show a similar profile to 1,4-dimethylbenzene, i.e. they are classified as Cat 1 due to CO persistence in a single animal but do not fulfil any of the classification criteria (for Cat 1 or Cat 2) on the basis of the tissue scores recorded in the first three observation days (evidence 6a). Clear examples of studies where the majority of the animals showed full reversibility of effects by day 21 or earlier and one animal (or the minority of animals) showed persistence of low-level CO appearing throughout the study or persistence of delayed high level CO effects appearing beyond day 3 are studies No. 46, 51, 55, 57, 58, 59, 62, 65, 66, 67, 71, 72, 76, 80, 83, 92, 93, 97, 98, 102, 103, 107, 109, 118 and 119. Detailed information on individual tissue scores is provided in column “Comments” of the DRD (Supplementary Material 1). It is questionable whether this type of effects should lead to a Cat 1 classification, especially the cases of delayed effects observed in a single animal as these occur probably for reasons unrelated to the test chemical, such as mechanical abrasion due to prolonged exposure, microbial infection (the so-called secondary inflammatory process), and/or differences in animal behaviour (Prinsen 2006, 2014) (evidence 6a). Many of these studies were probably conducted before the 2002 update of OECD TG 405 (OECD 2012a), when rinsing of the eye was not allowed before the 24-h reading (changed to 1 h for solids in 2002). This may have led to a large variation of contact time between the test chemical and the eye from a couple of minutes to 24 h in different animals and may have thus led in some cases to an exacerbated exposure that could explain the discordant effects observed in a single animal (or the minority of the animals). To add to this, animals are immediately released into their home cages after treatment, where they can move freely. While some animals may immediately start grooming and/or scratching and do this excessively, others may not react at all. These variations in behaviour are another important source of variability between animals (Prinsen 2006, 2014).

A Cat 1 classification based only on persistence of low-level conjunctival effects (score 1) in the absence of any other Cat 1-triggering effects is also highly questionable. As already mentioned above, the US EPA classification system considers CR and CC scores of less than 2 as fully cleared (US EPA 1998, 2011; ICCVAM 2010). Adriaens et al. (2014) demonstrated that a significant proportion of the No Cat studies show mean CR scores over days 1–3 equal to or greater than 1. The information provided in the DRD (Supplementary Material 1) further demonstrates that a Conj mean ≥ 2 over days 1–3 in at least one animal can even occur in studies that do not lead to classification of the test chemical. This happens in 23 of the 343 No Cat studies (6.7 %) included in the DRD (Supplementary Material 1), none of which requires classification because such effects were not observed in the majority of the animals (subgroups CO >0** and CO = 0**). Indeed, conjunctival effects can only trigger a Cat 2 classification in case a CR mean ≥ 2 and/or a CC mean ≥ 2 is observed in the majority of the animals and therefore it seems logical to consider CR and CC scores less than 2 as “fully reversed” (evidence 7a). Of the 45 Cat 1 studies in the DRD (Supplementary Material 1) that show CR and/or CC > 0 on day 21 in at least one animal but have CO mean < 3 and IR mean ≤ 1.5 (in days 1–3), 27 (60 %) show maximum CR and CC = 1 on day 21 (studies No. 50, 52, 54, 55, 62, 65, 66, 67, 82, 83, 90, 93, 94, 95, 97, 100, 102, 108, 109, 110, 112, 113, 115, 116, 118, 121, and 122) and 18 (40 %) show CR and/or CC > 1 on day 21 in at least one animal (studies No. 53, 59, 61, 73, 78, 80, 81, 84, 85, 88, 103, 105, 107, 111, 114, 117, 119, and 120). Sixteen of 23 studies (69.6 %) with maximum CR and CC = 1 on day 21 (studies No. 54, 55, 62, 65, 66, 67, 83, 93, 94, 95, 97, 100, 102, 109, 112, and 113) and 6 of 15 studies (40 %) with CR and/or CC > 1 on day 21 in at least one animal (studies No. 59, 61, 80, 103, 107, and 114) show CR and/or CC > 0 on day 21 in a single animal or in a minority of the animals. For seven studies no conclusion could be made regarding minority versus majority of animals because persistence data are only available for one animal. More important, seven of the 45 Cat 1 studies mentioned above (No. 110, 112, 113, 115, 117, 121, and 122) are classified based only on persistence of conjunctival effects. Of these, 85.7 % (*n* = 6) show maximum CR and CC = 1 on day 21 (all but study No. 117) and in two studies (No. 112–10 % Triton X-100 and No. 113–10 % Igepon AC-78) a CR = 1 on day 21 was observed in only one out of six animals. From these data it can be concluded that: (1) in the majority of the studies where conjunctival effects do not reverse to 0 by day 21, the CR and/or CC scores obtained on day 21 are equal to or less than 1, and (2) in more than half of the studies where conjunctival effects do not reverse to 0 by day 21, these “persistent” effects are observed in only a single animal or in a minority of the animals. Conjunctival scores of 1 on day 21 have nevertheless an important weight in driving the Cat 1 classification of chemicals according to current UN GHS/EU CLP classification criteria. We question whether this is scientifically justifiable. The biological relevance of persistence of conjunctival effects in driving Cat 1 classification in the absence of any other Cat 1 triggering effects was already questioned by Adriaens et al. (2014). The analyses presented here provide further evidence in support of a recommendation to revise UN GHS and EU CLP classification criteria for classification of chemicals as Cat 1 based on persistence of conjunctival effects on day 21. It is strongly recommended that chemicals should not be classified as Cat 1 based on CR and/or CC scores of 1 on day 21, in the absence of any other Cat 1-triggering effects (evidence 7a).

The DRD (Supplementary Material 1) also contains several studies with animals for which a CO = 4 was noted that reversed to 0 by day 21 or earlier. These studies are marked in green in the column “Comments” in the DRD (Supplementary Material 1). In the current dataset, a CO = 4 scored anytime during the observation period was noted for 261 animals out of a total of 406 used in those studies. This represents only 64 % of the tested animals, which indicates that when CO = 4 is observed in one animal it is not consistently observed in the other animals used in the same study. Moreover, corneal opacity scores equal to 4 reversed to 0 by day 21 or earlier in 4.6 % (12/261) of the animals (2 animals in studies No. 2, 27, and 155; 1 animal in studies No. 11, 63, 125, 126, 136, and 144) and persisted (CO > 0) until day 21 in 25.7 % (67/261) of the animals. The majority of the animals having CO = 4 (69.7 %, 182/261) were killed before day 21 with CO > 0 due to animal welfare concerns, but 28 % (73/261) were killed on day 14 with CO = 4 and 4.6 % (12/261) with CO = 3 also on day 14. For all of these, it is quite probable that CO would have persisted until day 21 but for the other 37.2 % (97/261) it remains unknown whether full recovery could have occurred. According to the US EPA classification system, test chemicals showing CO = 4 that reverses to 0 by day 21 or earlier are not classified as US EPA cat 1 if CO and IR revert to 0 and CR and CC revert to less than 2 by day 21 in all tested animals (US EPA 1998, 2011; ICCVAM 2010). The UN GHS/EU CLP Cat 1 classification is also driven by the occurrence of “irreversible effects on the eye”, being defined as “the production of tissue damage in the eye,… which is not fully reversible within 21 days of appli-cation” (UN 2103; EC 2008). Conversely, Cat 2 is associated with “reversible effects on the eye”, being defined as “the production of changes in the eye …, which are fully reversible within 21 days of application”. UN GHS and EU CLP also state that “grade 4 cornea lesions and other severe reactions… observed at any time during the test…” are considered as serious eye damage and should be classified as Cat 1. These criteria were defined to permit the ethical early termination of studies where such extreme CO scores are observed because these effects are generally not expected to reverse within 21 days. A question arises, however, when these studies are followed through to the end and the observed grade 4 CO actually reverses to 0 within 21 days. Studies in the DRD (Supplementary Material 1) with CO = 4 recorded any time during the observation period for one or more animals in the absence of any other Cat 1-triggering effects and showing or expected to show (if terminated before day 21) full reversibility of all endpoints by day 21 at latest are studies No. 125 (Methoxyethyl acrylate), 126 (Methyl thioglycolate), 144 (Acid blue 40), and 155 (Thiourea). Considering the high subjectivity of the scoring of ocular lesions in the in vivo Draize eye test (Prinsen 2014) and the fact that no other Cat 1-triggering effects (severity or persistence) were observed in these studies, it is questionable if these chemicals should be classified Cat 1. In fact, the Cat 2 classification criteria are fulfilled in all these cases, including full reversibility within 21 days. It is therefore proposed that a Cat 2 classification would be more appropriate (evidence 9).

The data presented and discussed in this paper further support the various recommendations made earlier by Adriaens et al. (2014) on critical revisions of the UN GHS and EU CLP decision criteria for the classification of chemicals based on the in vivo Draize eye test. On the basis of the evidence provided in this paper, implementation of the following recommendations should thus be considered: (1) CR and CC scores of less than 2 on day 21 should be recognised as fully reversed and should therefore not drive a Cat 1 classification in the absence of any other Cat 1 triggering effects; (2) the classification of chemicals as Cat 1 based on persistence of effects (i.e. CO > 0, IR > 0, CR > 1 or CC > 1 on day 21) or CO = 4 observed anytime during the study should follow a majority rule as currently done for effects observed in days 1–3 (severity), i.e. in at least 2 out of 3, 3 out of 4, 3 out of 5, or 4 out of 6 animals—in particular, low-level persistent effects or persistence occurring due to delayed effects, which are observed in a single animal are probably not related to the test chemical and should therefore not drive a Cat 1 classification in the absence of any other Cat 1 triggering effects in the study and (3) grade 4 CO scores that fully reverse within 21 days should not trigger a Cat 1 classification in the absence of any other Cat 1 triggering effects. Importantly, until such time as these proposed revisions are implemented, chemicals that are considered as Cat 1 on the basis of the effects described above should not be included in validation studies of alternative methods. Such chemicals could lead to a decision of non-validity of an alternative method when validation criteria such as the absence of under classified Cat 1 chemicals is used which, in our opinion, would not be scientifically justifiable. Therefore, the establishment of validity criteria and chemicals selection should be carefully considered before initiating a validation study.

### Drivers of classification criteria to consider when selecting reference chemicals

On the basis of the in-depth analyses provided in this paper, a number of key criteria have been identified that should be taken into consideration when selecting reference chemicals for the development, evaluation and/or validation of alternative methods and/or strategies for serious eye damage/eye irritation testing. These are:

- The expected applicability of the alternative method in terms of UN GHS/EU CLP hazard category prediction needs to be established. Depending on this applicability, different alternative methods will predict different classification scenarios within the UN GHS/EU CLP classification systems, which are: (1) discriminating all individual categories, i.e. “Cat 1 vs. Cat 2 vs. No Cat”, (2) “No Cat vs. [Classified (Cat 2/Cat 1)]” and (3) “Cat 1 vs. (Cat 2/No Cat)”.
- Important drivers of classification for each UN GHS/EU CLP category need to be represented in the chemicals selection. These are:
  - For Cat 1: CO mean ≥ 3 (days 1–3) in ≥60 % of the animals; CO persistence on day 21 in ≥60 % of the animals (with CO mean < 3); CO = 4 in ≥60 % of the animals in the absence of persistence and with CO mean < 3 (or if unknown) (conclusion supported by evidence 1a, b, c and 8a, b, c).
  - For Cat 2: CO mean ≥ 1 and CR mean ≥ 2 (including some chemicals where each of these two drivers appears in the absence of the other one) (conclusion supported by evidence 4a, b).
  - No Cat: CO = 0 and CO > 0, i.e. clear negative results in which no or minor effects are observed; a few CO = 0** and CO > 0**, i.e. borderline in vivo results (conclusion supported by evidence 5). Some chemicals with borderline in vivo results should be tested during method development in order to avoid the development of highly overpredictive methods and should also be tested in validation studies to properly assess the specificity of the alternative methods.
  The distribution of the drivers of classification may differ depending on the purpose of the alternative method. The inclusion of IR and CC as drivers of classification in the chemicals selection is not important since they rarely drive the classification of chemicals in vivo (conclusion supported by evidence 2a, b, c, d, e).
- Chemicals classified in vivo (Draize eye test) as Cat 1 based only on CO = 4 and/or persistent effects appearing in a minority (<60 %) of the animals, should not be used in either prospective studies or retrospective evaluations (conclusion supported by evidence 6a, b, c, d).
- Chemicals classified in vivo (Draize eye test) as Cat 1 on the basis of single animal studies should not be used in prospective studies due to the high level of uncertainty associated with the classifications derived from such studies (conclusion supported by evidence 6a). Caution should be applied for their use in retrospective evaluations for which the physico-chemical parameters should also be taken into consideration.
- Chemicals classified in vivo (Draize eye test) as Cat 1 based only on persistence of CR and/or CC equal to 1 on day 21 should not be used in either prospective studies or retrospective evaluations (conclusion supported by evidence 7a). Furthermore, inclusion of CR and/or CC persistence ≥2 on day 21 as drivers of classification is not important, since when this occurs it is mostly accompanied by CO persistence on day 21 (conclusions supported by evidence 7b, c).
- Chemicals classified in vivo (Draize eye test) as Cat 1 based on CO = 4 that reversed to 0 before day 21, even if CO = 4 was observed in the majority of the animals, should not be used in either prospective studies or retrospective evaluations (conclusion supported by evidence 9).
- Only those chemicals that have full observed data from which a definitive classification can be derived should be selected. Chemicals that have in vivo data that are SCNM should not be routinely used in prospective studies, unless other studies for the same chemical are available in the DRD having a clear classification that appears to be consistent with what was observed in the SCNM study. Chemicals with only a SCNM study but for which a classification can be assumed with some level of certainty, i.e. those that are identified in the DRD as “SCNM (Cat 1)”, “SCNM (Cat 2A)”, “SCNM (Cat 2)”, “SCNM (Cat 2B)”, or “SCNM (No Cat)”, should still not be used in prospective studies because better options with a clear classification are available in the DRD. Chemicals that are identified in the DRD as “SCNM (Cat 1)”, “SCNM (Cat 2A or higher)”, “SCNM (Cat 2 or Cat 1)”, “SCNM (Cat 2A)”, “SCNM (Cat 2)”, or “SCNM (Cat 2B)” may however be used in retrospective evaluations of alternative methods for the classification scenario “No Cat versus Classified (Cat 2/Cat 1)” since the available in vivo data allows the conclusion that all of these chemicals require classification (the uncertainty lies only on the correct classification). Such chemicals should nevertheless not be used for the development/evaluation of testing strategies, where the aim is to fully replace the in vivo Draize eye test.
- Chemicals that are classified Cat 1 based on specific observations in the absence of any other Cat 1 triggering effect should in general not be selected for prospective testing in validation studies as this accounts for a very limited number of studies in the DRD and better reference chemicals are available for selection (conclusion supported by evidence 3). An exception could be the testing of strong colourants in order to demonstrate the capacity of an alternative method to correctly classify chemicals classified in vivo due to discoloration of the cornea (e.g. studies No. 159 and 160 in the DRD).
- Chemicals for which repeat in vivo studies are identified in the DRD should have the following considerations taken into account:
  - Repeat in vivo studies with discordant classifications should not be used in either prospective studies or retrospective evaluations. Chemicals with repeat studies identifying Cat 2A and Cat 2B classifications (e.g. Methyl acetate with studies No. 176 and 225, and n-Octanol with studies No. 181 and 228) may still be used when these two optional categories are not implemented and a unified Cat 2 classification is used (e.g. under the EU CLP classification system).
  - The selection of chemicals with repeat in vivo studies with concordant classifications but based on different main drivers depends on the purpose of the alternative method.
  - For No Cat chemicals in which studies showing in vivo responses CO > 0** and CO = 0 (e.g. phosphoric acid, tributyl ester; studies No. 260 and 446) should not be used in either prospective studies or retrospective evaluations as this is considered a large variation in the in vivo responses that could lead to an apparent over-classification by alternative methods.
- Chemicals that were tested at different concentrations and where the highest classification was obtained with the lowest concentration should only be selected with caution and depending on the test chemical in question (it’s chemical class and functional use) and the in vitro test method under evaluation.
- It is important to take into account the physical form (liquids, solids, waxy/viscous) of the chemicals as tested and balance the selection appropriately, where possible. For chemicals that are tested in solvent, the choice of vehicle is important as is the identification of the physical form of the neat chemical and of the chemical as tested, e.g. solution, suspension, emulsion.
- The purity of the chemicals should be as high as possible and ideally ≥95 %.
- Chemicals should be relevant in terms of their representative functional and chemical classes and industrial use. This may involve choosing chemicals covering wide ranging or selected organic functional groups (determined using, e.g. the OECD QSAR Toolbox, as provided in column “Organic Functional Groups” of the DRD), and covering wide ranging or selected functionality/industrial use (e.g. industrial chemicals/intermediates, pesticides, pharmaceuticals, food additives.).

Based on the criteria described above, chemicals that should not be selected for the development, evaluation and/or prospective validation of alternative methods and/or testing strategies for serious eye damage/eye irritation are marked with an “X” in the DRD in the column entitled “Should Not Be Used” (Supplementary Material 1). Some chemicals that may be selected in certain circumstances are marked with “(X)”. This is the case for strong colourants (studies No. 159, 160 and 162), for chemicals that were tested in a single animal but that showed severe and persistent effects classifiable as Cat 1 (studies No. 50, 73 and 78) and for chemicals with repeat studies identifying Cat 2A and Cat 2B classifications (studies No. 176, 181, 225 and 228). Finally, chemicals that could in principle be chosen in future studies but that are proprietary or have unknown commercial source are marked with a “?”. All remaining chemicals that are not marked in the column entitled “Should Not Be Used” are considered to be good reference chemicals. Considering the chemicals in the DRD that are commercially available today (511 individual chemicals tested in 556 studies), only about 73 % (375 individual chemicals tested in 402 studies) are considered good reference chemicals that can be selected for future studies. About 1 % (6 individual chemicals tested in 8 studies) are not generally recommended for selection but may be used depending on the purpose of the study. That leaves about 25 % (130 individual chemicals tested in 146 studies) that are not recommended for selection. This is quite a large number considering that all of these chemicals were used in past validation studies of alternative methods and many may also have been used in their development. Regrettably, this may have led to sub-optimal development of the methods or, more importantly, to the methods being considered not scientifically valid in validation studies.

In conclusion, the DRD provided in Supplementary Material 1 is an invaluable tool for selecting reference chemicals with an appropriate coverage of the relevant in vivo drivers of classification for use in the development, evaluation and/or validation of alternative methods and testing strategies to assess the serious eye damage/eye irritation potential of chemicals.

## Electronic supplementary material

Below is the link to the electronic supplementary material.

The online version of this article contains the Draize eye test Reference Database (DRD) as supplementary material in PDF format (mentioned in the manuscript as Supplementary Material 1). The unlimited database will be available as Excel file upon request from Cosmetics Europe. To receive this file please contact Dr. Bertrand Desprez (bdesprez@cosmeticseurope.eu) (PDF 866 kb).

## Abbreviations

BCOP: Bovine Corneal Opacity and Permeability
CAS RN: Chemical Abstracts Service Registry Number
Cat 1: UN GHS/EU CLP classification for chemicals causing irreversible effects on the eye/serious damage to the eye
Cat 2: UN GHS/EU CLP classification for chemicals causing reversible effects on the eye/eye irritation, sub-categorised in 2A (irritant to eyes, eye effects are not fully reversible within 7 days of observation) and 2B (mildly irritant to eyes, eye effects fully reversible within 7 days of observation)
CC: Conjunctival chemosis
CO: Corneal opacity
Conj: CR and/or CC
CR: Conjunctival redness
CM: Cytosensor Microphysiometer
DRD: Draize eye test Reference Database
ECETOC: European Centre for Toxicology and Ecotoxicology of Chemicals
EIT: Eye Irritation Test
EU CLP: European Union Regulation on Classification, Labelling and Packaging of chemicals implementing UN GHS in the EU
EURL ECVAM: European Union Reference Laboratory for Alternatives to Animal Testing
EVEIT: Ex Vivo Eye Irritation Test
FL: Fluorescein Leakage
HET-CAM: Hen’s Egg Test-Chorioallantoic Membrane
HPLC/UPLC: High-Performance Liquid Chromatography/Ultra-Performance Liquid Chromatography
ICCVAM: Interagency Coordinating Committee on the Validation of Alternative Methods
ICE: Isolated Chicken Eye
IR: Iritis
IRE: Isolated Rabbit Eye
LNS: Laboratoire National de la Santé
MMAS: Modified Maximum Average Score
MSDS: Material Safety Data Sheet
NCD: European New Chemicals Database
NICEATM: National Toxicology Program Interagency Center for the Evaluation of Alternative Toxicological Methods
No Cat: Chemicals not classified for serious eye damage/eye irritation under UN GHS/EU CLP
OECD: Organisation for Economic Co-operation and Development
pers: Persistence
PorCORA: Porcine Corneal Ocular Reversibility Assay
RCD: Reference Chemicals Database
RhCE: Reconstructed human Cornea-like Epithelium
SCNM: Study Criteria Not Met
STE: Short-Time Exposure
TG: Test Guideline
UN GHS: United Nations Globally Harmonized System of classification and Labelling of Chemicals
US EPA: United States Environmental Protection Agency
ZEBET: Zentralstelle zur Erfassung und Bewertung von Ersatz- und Ergänzungsmethoden zum Tierversuch (Centre for Documentation and Evaluation of Alternatives to Animal Experiments at the Federal Institute for Risk Assessment (BfR))
