# Supplementary material for: Cosmetics Europe compilation of historical serious eye damage/eye irritation in vivo data analysed by drivers of classification to support the selection of chemicals for development and evaluation of alternative methods/strategies: the Draize eye test Reference Database (DRD)
Source: Arch Toxicol. 2016 Mar 21;91(2):521–47. doi: 10.1007/s00204-016-1679-x (PMC5306081; doi:10.1007/s00204-016-1679-x)
Supplement: Supplementary file 1 — The online version of this article contains the Draize eye test Reference Database (DRD) as supplementary material in PDF format (mentioned in the manuscript as Supplementary Material 1). The unlimited database will be available as Excel file upon request from Cosmetics Europe. To receive this file please contact Dr. Bertrand Desprez (bdesprez@cosmeticseurope.eu) (PDF 866 kb). [file 204_2016_1679_MOESM1_ESM.pdf]

January 6, 2016

Cosmetics Europe compilation of historical serious eye damage/eye irritation *in vivo* data analysed by drivers of classification to support the selection of chemicals for development and evaluation of alternative methods/strategies: the Draize eye test Reference Database (DRD)

João Barroso[1,2,a], Uwe Pfannenbecker[3,b], Els Adriaens[4], Nathalie Alépée[5], Magalie Cluzel[6], Ann De Smedt[7], Jalila Hibatallah[8], Martina Klaric[1], Karsten R. Mewes[9], Marion Millet[10], Marie Templier[10], Pauline McNamee[11]

[1]Cosmetics Europe - The Personal Care Association, Brussels, Belgium
[2]European Commission Joint Research Centre, Institute for Health and Consumer Protection, EURL ECVAM / Systems Toxicology Unit, Ispra (VA),
[3]Italy Beiersdorf AG, Front End Innovation - Toxicology, Hamburg, Germany
[4]Adriaens Consulting BVBA, Aalter, Belgium
[5]L'Oréal Research & Innovation, Department of Predictive Biological Methods and Models, Aulnay Sous Bois, France
[6]LVMH Recherche - LVMH Parfums et Cosmétiques, Product Safety Department, St. Jean de Braye Cedex, France
[7]Janssen Research & Development, Preclinical Development & Safety, Beerse, Belgium
[8]Chanel Parfums Beauté, Toxicology Department, Neuilly sur Seine, France
[9]Henkel AG & Co. KGaA, Biological & Clinical Research, Düsseldorf, Germany
[10]Pierre Fabre, In Vitro Metabolism and Alternative Methods, Castres, France
[11]The Procter & Gamble Company, Central Product Safety, Egham, Surrey, UK

[a]JB's current affiliation is European Commission Joint Research Centre (EC JRC). His contribution to this work while affiliated to Cosmetics Europe occurred between October 2011 and October 2013.

[b]Corresponding author; e-mail: Uwe.Pfannenbecker@Beiersdorf.com; phone: +49 40 49093916

Supplementary Material 1 (Archives of Toxicology)

Draize eye test Reference Database (DRD)

Cosmetics Europe
Draize eye test Reference Database (DRD)
Barroso *et al.* , 2016

Study Number	Test Chemical Name	CAS RN	Organic Functional Groups	Physical Form as tested	Physical Form Confirmation e.g., by MSDS (Y / N)	Data Source	Commercial Source	Available Purity	Number of Studies	UN GHS Classification	Drivers of Classification						Comments	Should Not Be Used
											Severity		Persistence		Specific Observations			
											Cut-off values	Number of animals	Cut-off time	Number of animals	CO = 4 or other observations	Number of animals		
1	2-Hydroxy iso-butyric acid ethyl ester	80-55-7	Alcohol| Carboxylic acid ester	L	Y	ZEBET	Sigma-Aldrich	98%	1 of 1	Cat 1	**CO mean ≥ 3**	3/3	Unknown				Study terminated on D3	
2	3-Diethylaminopropionitrile	5351-04-2	Aliphatic Amine, tertiary| Nitrile	L	Y	ECETOC	Chem Service, Inc.	98%	1 of 1	Cat 1	**CO mean ≥ 3**	2/3	Unknown		CO = 4	3/3	CO = 4 between D1 and D3 in 1/3 that reverts to CO ≤ 2 on D7 with study terminated on D7 with CC = 4; CO = 4 between D1 and D2 in 1/3 fully reversed by D7; CO = 4 on D1 in 1/3 fully reversed by D7	
3	Benzensulphonylchloride	98-09-9	Aryl| Sulfonyl halide	L	Y	NICEATM	Sigma-Aldrich	≥99%	1 of 1	Cat 1	**CO mean ≥ 3**; IR mean > 1.5	4/6; 4/6	CO pers D21; Conj pers D21	at least 5/6;<br>at least 4/6	CO = 4	5/6	CO = 4 between D3 and D7 and on D14 with CO = 2 on D21 and D24, CR = 3 between D2 and D7 with CR = 2 on D21 and CR = 1 on D24, and CC = 2 between D1 and D7 with CC = 1 on D21 and D24 in 1/3; CO = 4 between D3 and D7 and on D14 with CO = 2 on D21 and D24, CR = 3 between D2 and D7 with CR = 2 on D21 and CR = 1 on D24, and CC = 3 on D1 with CC = 1 on D21 and D24 in 1/3; CO = 4 between D3 and D14 with CO = 3 on D21 and CO = 2 on D24, CR = 3 between D1 and D7 with CR = 1 on D21 and D24, and CC = 2 between D1 and D2 and on D7 fully reversed by D21 in 1/3; CO = 4 between D3 and D4 with CO = 2 on D21 and D24, CR = 3 between D1 and D7 fully reversed by D17, and CC = 2 between D1 and D14 fully reversed by D17 in 1/3; CO = 4 between D3 and D14 with remaining scores unknown due to pannus formation, CR = 3 between D2 and D7 with CR = 2 on D21 and CR = 1 on D24, and CC = 2 between D1 and D7 with CC = 1 on D21 and D24 in 1/3; One animal sacrificed on D4 due to ruptured cornea with CO = 3 between D2 and D4, CR = 3 between D2 and D4, and CC = 2 on D1 with CC = 1 on D4	
4	bis-(3-Aminopropyl)-tetramethyldisiloxane	2469-55-8	Aliphatic Amine, primary| Disiloxane	L	Y	NICEATM	Alfa Aesar	97%	1 of 1	Cat 1	**CO mean ≥ 3**; IR mean > 1.5	2/2; 2/2	Unknown		CO = 4	2/2	Study terminated on D3; CO = 4 between Hr1 and D3 in 2/2	
5	Cyclohexanol	108-93-0	Alcohol| Cycloalkane	L	Y	ECETOC	Sigma-Aldrich	99%	1 of 1	Cat 1	**CO mean ≥ 3**	3/4	CO pers D21	1/4			CO = 3 between D1 and D7 with CO = 1 on D21 in 1/4; CO = 3 between D2 and D3 fully reversed by D10 in 1/4; CO = 3 between D1 and D7 fully reversed by D14 in 1/4; CO = 3 between D1 and D3 fully reversed by D10 in 1/4	
6	Cyclohexyl isocyanate	3173-53-3	Cycloalkane| Isocyanate	L	Y	NICEATM	Sigma-Aldrich	98%	1 of 1	Cat 1	**CO mean ≥ 3**	2/2	CO pers D21	2/2	CO = 4	2/2	CO = 4 between D1 and D21 in 2/2	
7	Diethylethanolamine (neat)	100-37-8	Alcohol| Aliphatic Amine, tertiary	L	Y	NICEATM	Sigma-Aldrich	≥99.5%	1 of 1	Cat 1	**CO mean ≥ 3**	4/6	CO pers D21; Conj pers D21	5/6; 5/6	CO = 4	5/6	CO = 4 between D2 and D21, IR scores unknown, CR = 3 on D2 with CR = 1 on D21 and CC = 3 between D1 and D8 with CC = 1 on D21 in 1/6; CO = 4 on Hr1 and between D1 and D21, IR scores unknown, CR = 3 on D1 with CR = 1 on D21 and CC = 3 between D1 and D3 with CC = 2 on D21 in 1/6; CO = 4 between D8 and D21, IR = 1 between D1 and D2 with remaining scores unknown, CR = 2 between D1 and D14 with CR = 1 on D21 and CC = 3 between D3 and D14 with CC = 2 on D21 in 1/6; CO = 4 between D2 and D21, IR scores unknown, CR = 3 between D1 and D2 fully reversed by D14 and CC = 2 between D1 and D10 with CC = 1 on D21 in 1/6; CO = 4 between Hr1 and D21, IR scores unknown, CR = 3 between D1 and D2 fully reversed by D21 and CC = 3 between D1 and D14 with CC = 1 on D21 in 1/6; CO = 1 between D1 and D3 fully reversed by D8, IR = 1 between D1 and D8 fully reversed by D10, CR = 2 between D1 and D3 fully reversed by D21 and CC = 2 between D1 and D3 fully reversed by D8 in 1/6	
8	Diethylethanolamine (50%)	100-37-8	Alcohol| Aliphatic Amine, tertiary	L	Y	NICEATM	Sigma-Aldrich	≥99.5%	1 of 1	Cat 1	**CO mean ≥ 3**	6/6	CO pers D21; Conj pers D21	6/6; 6/6	CO = 4	6/6	CO = 4 between D8 and D21, IR scores unknown, CR = 2 between D1 and D10 with CR = 1 on D21 and CC = 2 between D1 and D21 in 1/6; CO = 4 between Hr1 and D21, IR scores unknown, CR = 3 on D1 with CR = 1 on D21 and CC = 3 between D3 and D8 with CC = 2 on D21 in 1/6; CO = 4 between D8 and D21, IR scores unknown, CR = 3 on D1 with CR = 1 on D21 and CC = 3 on D8 with CC = 1 on D21 in 1/6; CO = 4 between Hr1 and D21, IR scores unknown, CR = 3 on D1 with CR = 1 on D21 and CC = 2 between D1 and D21 in 1/6; CO = 4 between Hr1 and Hr4 and between D2 and D21, IR scores unknown, CR = 3 on D1 with CR = 1 on D21 and CC = 3 between D10 and D14 with CC = 2 on D21 in 1/6; CO = 4 between Hr1 and D21, IR scores unknown, CR = 3 between D1 and D2 with CR = 1 on D21 and CC = 3 between D3 and D14 with CC = 2 on D21 in 1/6	
9	Diethylethanolamine (25%)	100-37-8	Alcohol| Aliphatic Amine, tertiary	L	Y	NICEATM	Sigma-Aldrich	≥99.5%	1 of 1	Cat 1	**CO mean ≥ 3**	4/6	CO pers D21; Conj pers D21	6/6; 6/6	CO = 4	6/6	CO = 4 between D8 and D21, IR scores unknown, CR = 3 on D1 with CR = 1 on D21 and CC = 3 between D1 and D2 with CC = 2 on D21 in 1/6; CO = 4 between D8 and D21, IR = 1 between D1 and D2 with remaining scores unknown, CR = 2 between D1 and D10 with CR = 1 on D21 and CC = 3 between D10 and D14 with CC = 2 on D21 in 1/6; CO = 4 between D8 and D21, IR scores unknown, CR = 2 between D1 and D14 with CR = 1 on D21 and CC = 3 on D1 and D10 with CC = 1 on D21 in 1/6; CO = 4 between D8 and D21, IR = 1 between D1 and D2 with remaining scores unknown, CR = 2 between D1 and D10 with CR = 1 on D21 and CC = 2 between D1 and D14 with CC = 1 on D21 in 1/6; CO = 4 on D2 and between D8 and D21, IR scores unknown, CR = 2 between D1 and D14 fully reversed by D21 and CC = 2 between D1 and D14 with CC = 1 on D21 in 1/6; CO = 4 between D2 and D21, IR scores unknown, CR = 2 between D1 and D10 fully reversed by D21 and CC = 3 between D8 and D14 with CC = 1 on D21 in 1/6	
10	Ethylhexyl acid phosphate ester	12645-31-7	Alkoxy| Phosphate ester	L	Y	NICEATM	Merck	≥95%	1 of 1	Cat 1	**CO mean ≥ 3**	6/6	Unknown		CO = 4	6/6	Study terminated on D10 in 4/6 and on D7 in 2/6; CO = 4 between D2 and D10 in 2/6; CO = 4 between D1 and D2 and on D7 in 1/6; CO = 4 between D1 and D2 and between D7 and D10 in 1/6; CO = 4 on Hr1, between D2 and D3 and on D10 in 1/6; CO = 4 between D1 and D2 and on D7 in 1/6	
11	Lactic acid (neat)	50-21-5	Alcohol| Carboxylic acid	L	Y	NICEATM	Sigma-Aldrich	90%	1 of 1	Cat 1	**CO mean ≥ 3**	3/3	CO pers D21 (at least)	2/3	CO = 4	3/3	Study terminated on D14; CO = 4 between Hr1 and D13 fully reversed by D14 and IR = 2 between D2 and D8 fully reversed by D9 in 1/3; CO = 4 between Hr1 and D14 and IR = 2 between D5 and D14 in 1/3; CO = 4 between Hr1 and D14 and IR = 2 between D4 and D14 in 1/3; CR = 3 on D14 in 1/3; CR = 2 on D14 in 2/3; CC = 2 on D14 in 3/3; Delayed IR effects; Assumed CO irreversible	
12	Phosphorodicloridic acid, ethyl ester	1498-51-7	Alkoxy	L	Y	NICEATM	Sigma-Aldrich	96%	1 of 1	Cat 1	**CO mean ≥ 3**	6/6	CO pers D21 (at least)	6/6	CO = 4	6/6	Study terminated on D14; CO = 4 between Hr1 and D14 in 6/6; IR scores unknown; CR = 3 on D14 in 6/6; CC = 4 on D14 in 6/6; Assumed CO irreversible; A second study is also reported in NICEATM ALTTOX with the exact same animal scores and is considered a duplicate entry	
13	Pyridine (1 of 2)	110-86-1	Aryl| Pyridine	L	Y	ECETOC	Sigma-Aldrich	>99.9%	1 of 2	Cat 1	**CO mean ≥ 3**	2/3	CO pers D21 (at least)	at least 1/3	CO = 4	1/3	Study terminated on D14; Delayed effects; CO = 3 between D1 and D7 with CO = 4 on D14, IR = 2 between D2 and D14, CR = 3 between D1 and D14 and CC = 4 on D1 and D3 with CC = 3 on D14 in 1/3; CO = 2 between D1 and D7 with CO = 1 on D14, IR = 1 between D1 and D7 fully reversed by D14, CR = 3 between D2 and D7 with CR = 1 on D14 and CC = 4 on D3 with CC = 1 on D14 in 1/3; CO = 3 between D1 and D4 with CO = 2 on D14, IR = 1 between D1 and D4 fully reversed by D7, CR = 3 between D1 and D4 fully reversed by D14 and CC = 3 between D1 and D3 fully reversed by D14 in 1/3; Assumed CO irreversible	X
14	4-tert-Butylcatechol (85%, probably in methanol)	98-29-3	Precursors quinoid compounds| tert-Butyl	L (tested in solvent, available as S)	Y	NICEATM	Sigma-Aldrich	≥99%	1 of 1	Cat 1	**CO mean ≥ 3**; IR mean > 1.5	6/6; 6/6	CO pers D21; Conj pers D21; IR pers D21	at least 4/6;<br>at least 3/6;<br>at least 3/6	CO = 4	6/6	Two animals sacrificed on D7 and 1 animal died on D20; CO = 4 between Hr1 and D21, IR = 2 between D1 and D7 and on D21, CR = 2 between D2 and D14 with CR = 1 on D21 and CC = 4 on D3 with CC = 2 on D21 in 1/6; CO = 4 between Hr1 and D21, IR = 2 between D1 and D7 with IR = 1 on D21, CR = 2 between D3 and D21 and CC = 4 between D3 and D7 with CC = 2 on D21 in 1/6; CO = 4 between Hr1 and D21, IR = 2 between D1 and D21, CR = 3 on D14 with CR = 2 on D21 and CC = 4 between D1 and D3 with CC = 3 on D21 in 1/6; CO = 4 between Hr1 and D14, IR = 2 between D1 and D14, CR = 2 between D1 and D14 and CC = 4 between D1 and D3 with CC = 3 on D14 in 1/6; CO = 4 between Hr1 and D7, IR = 2 between D1 and D7, CR = 2 on D1 and between D3 and D7 and CC = 4 on D1 and between D3 and D7 in 1/6; CO = 4 between Hr1 and D7, IR = 2 between D1 and D7, CR = 2 between D3 and D7 and CC = 4 between D3 and D7 in 1/6	
15	Aluminium chloride (concentration unknown)	16603-84-2	N/A	L (tested in solvent, available as S)	N	NICEATM	Unknown	Unknown	1 of 1	Cat 1	**CO mean ≥ 3**	4/6	CO pers D21; Conj pers D21	at least 5/6;<br>at least 3/6	CO = 4	5/6	CO = 4 on D7 (score of D14 unknown) with CO = 2 on D21, CR = 3 on D14 with CR = 1 on D21 and CC = 3 between D1 and D2 with CC = 2 on D7 (scores of D14 and D21 unknown) in 1/6; CO = 4 between D1 and D7 (score of D14 unknown) with CO = 1 on D21, CR = 2 between D1 and D7 (scores of D14 and D21 unknown) and CC = 3 between D1 and D3 with CC = 2 on D7 (scores of D14 and D21 unknown) in 1/6; CO = 4 on D7 with CO = 2 on D21, CR = 2 between D2 and D14 with CR = 1 on D21 and CC = 3 on D1 with CC = 2 on D7 (scores of D14 and D21 unknown) in 1/6; CO = 2 between D2 and D3 with CO = 1 on D7 (scores of D14 and D21 unknown), CR = 2 between D1 and D14 with CR = 1 on D21 and CC = 2 between D1 and D7 fully reversed by D14 in 1/6; CO = 4 between D2 and D7 with CO = 2 on D21, CR = 2 between D1 and D7 (scores of D14 and D21 unknown) and CC = 3 on D1 and D3 with CC = 2 on D7 (scores of D14 and D21 unknown) in 1/6; CO = 4 on D7 with CO = 1 on D21, CR = 2 between D1 and D7 (scores of D14 and D21 unknown) and CC = 3 on D1 and D3 with CC = 2 on D7 (scores of D14 and D21 unknown) in 1/6	?
16	Benzalkonium chloride (10%)	63449-41-2	Ammonium salt| Benzyl	L (tested in solvent, available as S)	Y	ECETOC	Sigma-Aldrich	≥95%	1 of 1	Cat 1	**CO mean ≥ 3**; IR mean > 1.5	3/3; 3/3	CO pers D21; IR pers D21; Conj pers D21	3/3; 3/3; 2/3	CO = 4	3/3	CO = 4 between D1 and D21, IR = 2 between D1 and D21, CR = 3 between D1 and D7 with CR = 1 on D21 and CC = 4 between D1 and D3 with CC = 2 on D21 in 1/3; CO = 4 between D2 and D21, IR = 2 between D1 and D21, CR = 3 between D1 and D7 fully reversed by D21 and CC = 4 between D1 and D3 with CC = 2 on D21 in 1/3; CO = 4 between D3 and D21, IR = 2 between D1 and D21, CR = 3 between D1 and D7 fully reversed by D21 and CC = 4 between D1 and D3 fully reversed by D21 in 1/3	
17	Benzethonium chloride (10%)	121-54-0	Alkane branched with quaternary carbon| Ammonium salt| Aryl| Benzyl| Ether| tert-Butyl	L (tested in solvent, available as S)	Y	LNS	Sigma-Aldrich	≥99%	1 of 1	Cat 1	**CO mean ≥ 3**	2/3	CO pers D21 (at least)	at least 2/3	CO = 4	2/3	Delayed effects; Irreversible lesions on D21 but scores unknown; CO = 3 between D2 and D7 with CO = 4 on D14 in 1/3; CO = 3 between D1 and D3 and on D7 with CO = 4 on D14 in 1/3; CO = 3 between D1 and D3 and on D14 in 1/3; IR = 1 on D14 in 3/3; CR and CC = 3 on D14 in 3/3	
18	Domiphen bromide (10%)	538-71-6	Ammonium salt| Aryl| Ether	L (tested in solvent, available as S)	Y	NICEATM	Sigma-Aldrich	97%	1 of 1	Cat 1	**CO mean ≥ 3**	2/3	CO pers D21 (at least)	at least 2/3	CO = 4	3/3	Study terminated on D14; Delayed effects; CO = 4 between D4 and D14 in 1/3; CO = 4 between D6 and D14 in 1/3; CO = 4 on D1 and between D3 and D7 with CO = 3 on D14 in 1/3; IR = 0 on D14 in 3/3; CR = 2 on D14 in 2/3; CR = 1 on D14 in 1/3; CC = 2 on D14 in 1/3; CC = 1 on D14 in 1/3; CC = 0 on D14 in 1/3; Assumed CO irreversible	
19	2-Benzyl-4-chlorophenol (1 of 2)	120-32-1	Aryl halide| Benzyl| Phenol	S	Y	NICEATM	Sigma-Aldrich	95%	1 of 2	Cat 1	**CO mean ≥ 3**	6/6	CO pers D21 (at least)	6/6	CO = 4	6/6	Study terminated on D16; CO = 4 between D1 and D16 in 6/6; IR scores unknown; CR = 3 on D16 in 6/6; CC = 3 on D16 in 2/6; CC = 2 on D16 in 4/6; Assumed CO irreversible	
20	2-Hydroxy iso-butyric acid	594-61-6	Alcohol| Carboxylic acid	S	Y	ZEBET	Sigma-Aldrich	99%	1 of 1	Cat 1	**CO mean ≥ 3**	3/3	Unknown		CO = 4	3/3	Study terminated on D3; CO = 4 between Hr1 and D3 in 2/3; CO = 4 between D1 and D3 in 1/3	
21	4,4'-(4,5,6,7-Tetrabromo-3H-2,1-benzoxathiol-3-ylidene)bis[2,6-dibromophenol] S,S-dioxide	4430-25-5	Aromatic perhalogencarbons| Aryl halide| Benzoxathiole S-oxide| Phenol| Sulfonate ester	S	Y	EURL ECVAM	Chemos GmbH	96.3%	1 of 1	Cat 1	**CO mean ≥ 3**	2/3	Unknown		CO = 4	1/3	Study terminated on D3; CO = 4 between D2 and D3 in 1/3	
22	4-([2-Sulphatoethy] sulphony)-aniline	2494-89-5	Aniline| Sulfate| Sulfone	S	Y	ZEBET	IS Chemical Technology	98%	1 of 1	Cat 1	**CO mean ≥ 3**	2/3	Unknown		CO = 4	2/3	Study terminated on D3; CO = 4 between Hr1 and D3 in 2/3	
23	4-(1,1,3,3-Tetramethylbutyl)phenol	140-66-9	Precursors quinoid compounds	S	Y	NICEATM	Sigma-Aldrich	97%	1 of 1	Cat 1	**CO mean ≥ 3**; IR mean > 1.5	6/6; 6/6	Unknown		CO = 4	1/6	Study terminated on D7; CO = 4 on D7 in 1/6	
24	alpha-Ketoglutaric acid	328-50-7	Carboxylic acid| Ketone| Oxocarboxylic acid	S	Y	ZEBET	Sigma-Aldrich	≥99%	1 of 1	Cat 1	**CO mean ≥ 3**	3/3	Unknown		CO = 4	2/3	Study terminated on D3; CO = 4 on Hr1 and between D2 and D3 in 1/3; CO = 4 between D2 and D3 in 1/3	
25	Calcium thioglycolate (neat)	814-71-1	Carboxylic acid| Thioalcohol	S	Y	NICEATM	Sigma-Aldrich	≥98%	1 of 1	Cat 1	**CO mean ≥ 3**	3/3	CO pers D21 (at least)	3/3	CO = 4	3/3	Study terminated on D14; CO = 4 between Hr1 and D14 in 1/3; CO = 4 between D3 and D14 in 2/3; IR = 0 on D14 in 3/3; CR = 2 on D14 in 3/3; CC = 3 on D14 in 3/3; Assumed CO irreversible	

Study Number	Test Chemical Name	CAS RN	Organic Functional Groups	Physical Form as tested	Physical Form Confirmation e.g., by MSDS (Y / N)	Data Source	Commercial Source	Available Purity	Number of Studies	UN GHS Classification	Drivers of Classification						Comments	Should Not Be Used
											Severity		Persistence		Specific Observations			
											Cut-off values	Number of animals	Cut-off time	Number of animals	CO = 4 or other observations	Number of animals		
26	Dibenzoyl-L-tartaric acid (1 of 2)	2743-38-6	Aryl| Carboxylic acid| Carboxylic acid ester	S	Y	ECETOC	Sigma-Aldrich	98%	1 of 2	Cat 1	**CO mean ≥ 3**	2/3	Unknown				Study terminated on D14 with CO = 2, IR = 1, CC = 2 and CR = 2 in 1/3	
27	Dibenzoyl-L-tartaric acid (2 of 2)	2743-38-6	Aryl| Carboxylic acid| Carboxylic acid ester	S	Y	LNS	Sigma-Aldrich	98%	2 of 2	Cat 1	**CO mean ≥ 3**	2/3			CO = 4	2/3	CO = 4 between Hr1 and D4 in 2/3 fully reversed by D7 in 1/3 and by D21 in 1/3; Animal 1 with full reversibility of all endpoints on D7 (CO = 2 between D1 and D3 fully reversible by D4), animal 2 with full reversibility of all endpoints on D21 (CO = 4 between Hr1 and D4 fully reversible by D21) and animal 3 with full reversibility of all endpoints on D14 (CO = 4 between Hr1 and D4 fully reversible by D7)	
28	Distearyldimethylammonium chloride (neat)	107-64-2	Ammonium salt	S	Y	NICEATM	Sigma-Aldrich	≥97%	1 of 1	Cat 1	**CO mean ≥ 3**	3/3	CO pers D21 (at least)	3/3	CO = 4	3/3	Study terminated on D14; CO = 4 between D2 and D14 in 2/3; CO = 4 between D6 and D14 in 1/3; IR = 1 on D14 in 3/3; CR = 2 on D14 in 3/3; CC = 1 on D14 in 3/3; Assumed CO irreversible	
29	Ethyl lauroyl arginate HCl	60372-77-2	Aliphatic Amine, primary| Amidin| Carboxamide| Carboxylic acid ester| Guanidine	S	Y	EURL ECVAM	Proprietary	90.1%	1 of 1	Cat 1	**CO mean ≥ 3**	3/3	CO pers D21; Conj pers D21; IR pers D21	3/3; 3/3;<br>at least 2/3	CO = 4	3/3	CO = 4 between Hr1 and D3 with CO = 3 on D21, IR scores unknown between Hr1 and D14 with IR = 2 on D21, CR = 3 between D1 and D3 with CR = 2 on D21 and CC = 4 between D1 and D3 with CC = 2 on D21 in 1/3; CO = 4 between Hr1 and D7 with CO = 3 on D21, IR scores unknown between Hr1 and D14 with IR = 2 on D21, CR = 3 between D1 and D7 with CR = 2 on D21 and CC = 4 between D1 and D14 with CC = 3 on D21 in 1/3; CO = 4 between Hr1 and D21, IR scores unknown between Hr1 and D21, CR = 3 between D1 and D7 with CR = 2 on D21 and CC = 4 between D1 and D7 with CC = 3 on D21 in 1/3	?
30	Imidazole (1 of 2)	288-32-4	Aryl| Imidazole	S	Y	ECETOC	Sigma-Aldrich	≥99%	1 of 2	Cat 1	**CO mean ≥ 3**; IR mean > 1.5	3/3; 2/3	Possibly CO pers D21 (at least)	at least 2/3	CO = 4	2/3	Study terminated on D14; CO = 4 between D2 and D4 with CO = 2 on D14 in 1/3; CO = 4 between D2 and D3 with CO = 3 on D14 in 1/3; CO = 3 between D1 and D14 in 1/3; IR = 2 on D14 in 1/3; IR = 0 on D14 in 2/3; CR = 2 on D14 in 1/3; CR = 1 on D14 in 1/3; CR = 0 on D14 in 1/3; CC = 3 on D14 in 1/3; CC = 1 on D14 in 2/3	
31	Promethazine HCL (1 of 2)	58-33-3	Aliphatic Amine, tertiary| Phenothiazine	S	Y	ECETOC	Sigma-Aldrich	analytical grade	1 of 2	Cat 1	**CO mean ≥ 3**	3/3	Possibly CO pers D21 (at least)	3/3	CO = 4	3/3	Study terminated on D14; CO = 4 on Hr1 with CO = 3 on D14 in 3/3; IR = 0 on D14 in 3/3; CR = 1 on D14 in 2/3; CR = 0 on D14 in 1/3; CC = 1 on D14 in 2/3; CC = 0 on D14 in 1/3	
32	Promethazine HCL (2 of 2)	58-33-3	Aliphatic Amine, tertiary| Phenothiazine	S	Y	LNS	Sigma-Aldrich	analytical grade	2 of 2	Cat 1	**CO mean ≥ 3**	2/3	CO pers D21 (at least)	3/3	CO = 4	3/3	Delayed effects; Irreversible lesions on D21 but scores unknown; CO = 4 on Hr1 and D14 in 1/3; CO = 4 on Hr1 and between D7 and D14 in 1/3; CO = 4 on Hr1, D2 and D14 in 1/3; IR = 0 on D14 in 3/3; CR = 1 on D14 in 3/3; CC = 2 on D14 in 1/3; CC = 1 on D14 in 2/3	
33	Alkyl phosphoric acid ester/amine salt	Unknown	N/A	Unknown	N	NICEATM	Unknown	Unknown	1 of 1	Cat 1	**CO mean ≥ 3**	6/6	Unknown		CO = 4	5/6	Study terminated on D7; CO = 4 between D1 and D3 and on D7 in 1/6; CO = 4 between D1 and D2 and on D7 in 1/6; CO = 4 between D1 and D7 in 2/6; CO = 4 between D3 and D7 in 1/6	?
34	Hydroxyethyl acrylate	818-61-1	Acrylate| Alcohol	L	Y	NICEATM	Sigma-Aldrich	96%	1 of 1	Cat 1	**IR mean > 1.5**	6/6	CO pers D21 (at least)	at least 4/6	CO = 4	4/6	Study terminated on D14; Delayed effects; Data missing for D2 in 6/6 but all 6 animals had IR = 2 on D1 and D3; CO = 4 between D3 and D14 in 2/6; CO =4 on D14 in 2/6; CO = 3 on D14 in 1/6; CO =2 between D3 and D14 in 1/6; IR = 2 on D14 in 6/6; CR = 3 on D14 in 4/6; CR = 2 on D14 in 2/6; CC = 4 on D14 in 5/6; CC = 1 on D14 in 1/6; Assumed CO irreversible	
35	Tetraethylene glycol diacrylate	17831-71-9	Acrylate| Ether	L	Y	NICEATM	Sigma-Aldrich	technical grade	1 of 1	Cat 1	**IR mean > 1.5**	6/6	CO pers D21 (at least)	at least 5/6	CO = 4	5/6	Study terminated on D14; Data missing for D2 in 6/6 and for IR on D1 in 1/6; All 6 animals had IR = 2 on D3 and 5/6 also had IR = 2 on D1; Delayed effects; CO = 4 between D3 and D14 in 2/6; CO = 4 between D7 and D14 in 3/6; CO = 3 on D3 with CO = 2 on D14 in 1/6; IR = 2 on D14 in 6/6; CR = 3 on D14 in 6/6; CC = 4 on D14 in 6/6; Assumed CO irreversible	
36	Triton X-100 (neat) (1 of 3)	9002-93-1	Alcohol| Alkane branched with quaternary carbon| Aryl| Ether| tert-Butyl	L	Y	NICEATM	Sigma-Aldrich	laboratory grade	1 of 3	Cat 1	**IR mean > 1.5**	5/6	Unknown				Study terminated on D7; CO = 2 on D7 in 2/6; CO = 1 on D7 in 4/6; IR = 1 on D7 in 6/6	
37	Triton X-100 (neat) (2 of 3)	9002-93-1	Alcohol| Alkane branched with quaternary carbon| Aryl| Ether| tert-Butyl	L	Y	NICEATM	Sigma-Aldrich	laboratory grade	2 of 3	Cat 1	**IR mean > 1.5**	4/6	Unknown		CO = 4	2/6	Study terminated on D7; Delayed effects; CO = 4 on D7 in 2/6; CO = 3 on D7 in 1/6; CO = 2 on D7 in 2/6; CO = 1 on D7 in 1/6; IR = 2 on D7 in 2/6; IR = 1 on D7 in 3/6; IR = 0 on D7 in 1/6	
38	Triton X-100/Magnesium carbonate (40%/60%)	Mixture (CAS: 9002-93-1; CAS: 546-93-0)	N/A	L	Y	NICEATM	Triton X-100: Sigma-Aldrich; Magnesium carbonate: Sigma-Aldrich	Triton X-100: laboratory grade; Magnesium carbonate: USP grade	1 of 1	Cat 1	**IR mean > 1.5**	4/6	Unknown		CO = 4	2/6	Study terminated on D7; Delayed effects; CO = 4 on D7 in 2/6; CO = 3 on D7 in 1/6; CO = 2 on D7 in 2/6; CO = 1 on D7 in 1/6; IR = 2 on D7 in 2/6; IR = 1 on D7 in 3/6; IR = 0 on D7 in 1/6<br><br>Triton X-100 is Triton X-120 of Triton X-100 series; all have same CASRN for Triton X-100	
39	Benzalkonium chloride (5%)	63449-41-2	Ammonium salt| Benzyl	L (tested in solvent, available as S)	Y	ECETOC	Sigma-Aldrich	≥95%	1 of 1	Cat 1	**IR mean > 1.5**	4/4	CO pers D21; Conj pers D21; IR pers D21	2/4; 1/4; 1/4	CO = 4	1/4	CO = 4 between D7 and D21, IR = 2 between D1 and D21, CR = 2 between D2 and D3 fully reversed by D21 and CC = 3 between D1 and D3 fully reversed by D21 in 1/4; CO = 3 between D1 and D3 with CO = 2 on D21, IR = 2 between D2 and D7 fully reversed by D14, CR = 2 between D1 and D10 with CR = 1 on D21 and CC = 3 between D1 and D2 with CC = 1 on D21 in 1/4; CO = 3 between D1 and D2 fully reversed by D10, IR = 2 between D1 and D3 fully reversed by D10, CR = 3 between D1 and D2 fully reversed by D21 and CC = 3 between D1 and D2 fully reversed by D10 in 1/4; CO = 3 between D1 and D2 fully reversed by D10, IR = 2 between D1 and D2 fully reversed by D7, CR = 2 between D1 and D3 fully reversed by D21 and CC = 3 between D1 and D3 fully reversed by D21 in 1/4	
40	Cetyl pyridinium bromide (10%)	140-72-7	Ammonium salt| Aryl| Pyridine	L (tested in solvent, available as S)	Y	ECETOC	Sigma-Aldrich	98%	1 of 1	Cat 1	**IR mean > 1.5**; CO mean ≥ 3	6/6; 4/6	CO pers D21; Conj pers D21; IR pers D21	at least 4/6 (max 5);<br>at least 3/6 (max 4);<br>3/6	CO = 4	2/6	CO = 4 between D2 and D21, IR = 2 between D1 and D14 with IR = 1 on D21, CR = 2 between D1 and D14 with CR = 1 on D21 and CC = 4 on D1 with CC = 1 on D21 in 1/6; CO = 4 between D14 and D21, IR = 2 between D1 and D14 with IR = 1 on D21, CR = 3 on D7 with CR = 2 on D21 and CC = 4 between D1 and D7 with CC = 2 on D21 in 1/6; CO = 3 between D1 and D21, IR = 2 between D1 and D7 with IR = 1 on D21, CR = 2 between D1 and D21 and CC = 4 between D1 and D3 with CC = 2 on D21 in 1/6; CO = 3 between D1 and D21, IR = 2 between D1 and D3 fully reversed by D14, CR = 2 between D1 and D7 fully reversed by D21 and CC = 4 between D1 and D2 fully reversed by D21 in 1/6; CO = 3 between D2 and D3 fully reversed by D10, IR = 2 between D1 and D2 fully reversed by D7, CR = 2 between D1 and D3 fully reversed by D14 and CC = 4 on D1 fully reversed by D7 in 1/6; Study terminated on D14 in 1/6: CO = 3 between D3 and D7 with CO = 1 on D14, IR = 2 between D1 and D7 fully reversed by D14, CR = 2 between D1 and D10 with CR = 1 on D14 and CC = 4 between D1 and D3 with CC = 1 on D14	
41	Cetyl pyridinium bromide (6%)	140-72-7	Ammonium salt| Aryl| Pyridine	L (tested in solvent, available as S)	Y	ECETOC	Sigma-Aldrich	98%	1 of 1	Cat 1	**IR mean > 1.5**	3/4	Unknown				Study terminated on D10 for 1/4 with CO and IR = 1 and CR and CC = 2; Study terminated on D14 for 3/4 with CO = 1 in two animals and CR and CC = 1 in all three animals	
42	Antimony trioxide	1309-64-4	Carboxamide	S	Y	NICEATM	Sigma-Aldrich	99%	1 of 1	Cat 1	**IR mean > 1.5**	6/6	CO pers D21 (at least)	6/6	CO = 4	6/6	Study terminated on D14; Delayed effects; CO = 4 on D14 in 6/6; IR = 2 on D14 in 6/6; CR = 3 on D14 in 6/6; CC = 4 on D14 in 3/6; CC = 3 on D14 in 2/6; CC = 1 on D14 in 1/6; Assumed CO irreversible; A second study is also reported in NICEATM ALTTOX with the exact same animal scores and is considered a duplicate entry	
43	Benzothiazolium derivative	87699-86-3	N/A	S	N	NICEATM	Unknown	Unknown	1 of 1	Cat 1	**IR mean > 1.5**	3/3	Unknown				Study terminated on D2 due to irreversible iridic and conjunctival effects; CO = 0 in 3/3	?
44	Quinacrine (1 of 2)	69-05-6	Acridine| Aliphatic Amine, tertiary| Aryl halide| Ether	S	Y	ECETOC	Sigma-Aldrich	98%	1 of 2	Cat 1	**IR mean > 1.5**	2/3	Possibly CO pers D21 (at least)	3/3	CO = 4	3/3	Study terminated on D14 with CO = 3 in 3/3, IR = 2 in 3/3, and CR and CC = 2 in 3/3; CO = 4 on Hr1 in 3/3	
45	Xanthylium, 3,6-bis(diethylamino)-9-[2-(methoxy carbonyl)phenyl]-tetrafluoroborate	134429-57-5	Arene| Carboxylic acid ester| Conjugated hydrocarbon| Ether (cyclic)| Heterocyclic fragment| Imine	S	Y	EURL ECVAM	Proprietary	>99%	1 of 1	Cat 1	**IR mean > 1.5**	at least 2/3	Unknown		Other observations	3/3	Colour staining of cornea; Study terminated on D7	?
46	1,4-Dimethylbenzene	106-42-3	Aryl	L	Y	NICEATM	Sigma-Aldrich	≥99%	1 of 1	Cat 1	CO > 0 **	** Conj 2/6	**CO pers D21** (at least)	1/6	CO = 4	1/6	Study terminated on D14 in 1/6; Delayed effects; CO = 4 between D7 and D14 (CO = 0 between D2 and D3), IR = 2 between D7 and D14, CR = 3 on D1 and between D3 and D14 and CC = 4 between D3 and D7 with CC = 1 on D14 in 1/6; CO and IR = 0 between Hr1 and D21 in 5/6; CR = 0 between D1 and D21 in 1/6; CR fully reversible by D2 in 1/6; CR fully reversible by D7 in 1/6; CR fully reversible by D14 in 2/6; CR fully reversible by D7 in 2/6; CR fully reversible by D14 in 2/6; CR fully reversible by D21 in 1/6; Assumed CO irreversible	X
47	1-Chloroctan-8-ol	23144-52-7	Alcohol| Alkyl halide	L	Y	ZEBET	Sigma-Aldrich	98%	1 of 1	Cat 1	CO mean ≥ 1; Conj mean ≥ 2	3/3; 3/3	**CO pers D21**	3/3			CO = 1 between D1 and D21 in 3/3	
48	Acetic acid (10%)	64-19-7	Acetoxy| Carboxylic acid	L	Y	NICEATM	Sigma-Aldrich	≥99.7%	1 of 1	Cat 1	CO mean ≥ 1	3/3	**CO pers D21** (at least)	at least 2/3	CO = 4	2/3	Study terminated on D14; Delayed effects; CO = 4 on D14 in 2/3 and CO = 3 between D7 and D14 in 1/3; IR = 0 on D14 in 3/3; CR = 0 on D14 in 3/3; CC = 1 on D14 in 3/3; Assumed CO irreversible	
49	Benzyl alcohol (neat)	100-51-6	Alcohol| Benzyl	L	Y	NICEATM	Sigma-Aldrich	99.8%	1 of 1	Cat 1	CO mean ≥ 1	2/3	**CO pers D21** (at least)	at least 1/3	CO = 4	2/3	Study terminated on D14 in 2/3 and on D7 with full reversibility of all endpoints in 1/3; Delayed effects; CO = 4 between D10 and D14 in 1/3; CO = 4 between D8 and D12 with CO =3 on D14 in 1/3; CO = 1 between D3 and D4 fully reversed by D5 in 1/3; IR = 0 on D14 in 2/3; CR = 1 on D14 in 2/3; CC = 1 on D14 in 1/3; CC = 0 on D14 in 1/3; Assumed CO irreversible	
50	Bisphenol A, diethylene triamine, epichlorohydrin, polypropylene glycol diglycidyl ether, polymer (~60%, aqueous)	118569-52-1	Aliphatic Amine, primary| Aliphatic Amine, secondary| Alkyl halide| Epoxide| Ether| Phenol| Saturated heterocyclic fragment	L	Y	EURL ECVAM	Proprietary	~60%, aqueous	1 of 1	Cat 1	CO mean ≥ 3; IR mean > 1.5	1/1; 1/1	**CO pers D21**; Conj pers D21; IR pers D21	1/1; 1/1; 1/1			CO = 3 between D1 and D21, IR = 2 between D1 and D21 and CC = 3 on D1 with CC = 1 on D21 (CC = 0 between D7 and D14)	(X)/?
51	Butoxyethanol (1 of 3)	111-76-2	Alcohol| Alkoxy| Ether	L	Y	EURL ECVAM	Sigma-Aldrich	≥99%	1 of 3	Cat 1	CO mean ≥ 1; Conj mean ≥ 2; IR mean ≥ 1	6/6; 6/6; 5/6	**CO pers D21** (at least)	at least 1/6 (max 2)			CO = 1 between D1 and D3 with CO = 2 on D21 in 1/6; CO = 1 between D1 and D3 fully reversed by D7 in 3/6; CO = 2 on D3 fully reversed by D14 in 1/6; Study terminated on D14 for humane reasons after signs of discomfort with CO = 2 (CO = 1 between D1 and D3), IR = 1 (IR = 1 between D1 and D3) and CR and CC = 2 (CR and CC = 2 between D1 and D3) in 1/6; Scores between D1 and D3 almost identical in all 6 animals	X
52	Calcium sulphydrate (20% solution)	12133-28-7	N/A	L	N	ECETOC	Unknown	Unknown	1 of 1	Cat 1	CO mean ≥ 1; Conj mean ≥ 2	1/1; 1/1	**CO pers D21**; Conj pers D21	1/1; 1/1			CO= 2 between D1 and D3 with CO = 1 on D21 in 1/1; CC = 4 between D1 and D3 with CC = 1 on D21 in 1/1	X
53	Coco alkyl dimethyl betaine (~30%, aqueous)	68424-94-2	Ammonium salt| Carboxylic acid	L	Y	EURL ECVAM	Proprietary	~30%, aqueous	1 of 1	Cat 1	CO mean ≥ 1; Conj mean ≥ 2	4/4; 4/4	**CO pers D21**; Conj pers D21	4/4; 3/4			CO = 1 between D1 and D21 in 4/4; CR = 2 between D1 and D21 in 1/4; CR = 2 between D1 and D10 with CR = 1 on D21 in 1/4; CR = 3 between D1 and D3 with CR = 1 on D21 in 1/4; CR = 3 between D1 and D10 fully reversed by D21 in 1/4	?
54	Coco amidopropyl betaine (~30%, aqueous)	61789-40-0	Ammonium salt| Carboxamide| Carboxylic acid	L	Y	EURL ECVAM	Proprietary	~30%, aqueous	1 of 1	Cat 1	CO mean ≥ 1; Conj mean ≥ 2	4/4; 4/4	**CO pers D21**; Conj pers D21	3/4; 2/4			CO = 1 between D1 and D21 and CR = 2 between D1 and D17 with CR = 1 on D21 in 1/4; CO = 1 between D1 and D21 and CR = 3 between D2 and D3 with CR = 1 on D21 in 1/4; CO = 1 between D1 and D21 and CR = 3 between D1 and D3 fully reversed by D21 in 1/4; CO = 1 between D1 and D3 fully reversed by D7 and CR = 3 between D2 and D3 fully reversed by D14 in 1/4; IR readings not reported	?
55	Di-sodium alkyl ether sulphosuccinate (~30%, aqueous)	68815-56-5	Alkoxy| Carboxylic acid| Ether| Sulfonic acid	L	Y	EURL ECVAM	Proprietary	~30%, aqueous	1 of 1	Cat 1	Conj mean ≥ 2; CO mean ≥ 1	4/4; 3/4	**CO pers D21**; Conj pers D21	1/4; 1/4			CO = 1 between D1 and D21 and CR = 3 between D2 and D3 with CR = 1 on D21 in 1/4; CO = 1 between D1 and D2 fully reversed by D3 and CR = 3 between D2 and D3 fully reversed by D10 in 1/4; CO = 1 between D1 and D3 fully reversed by D7 and CR = 3 on D2 fully reversed by D17 in 1/4; CO = 1 between D1 and D7 fully reversed by D10 and CR = 3 on D2 fully reversed by D10 in 1/4	X
56	EPIKOTE 604L	28768-32-3	Aromatic amine| Benzyl| Saturated heterocyclic fragment	L	Y	NICEATM	Sigma-Aldrich	Unknown	1 of 1	Cat 1	CO mean ≥ 1	1/1	**CO pers D21**	1/1			CO = 2 between D2 and D3 with CO = 1 on D21 in 1/1	X
57	Ethanol (neat) (1 of 4)	64-17-5	Alcohol	L	Y	NICEATM	Sigma-Aldrich	>99.8%	1 of 4	Cat 1	Conj mean ≥ 2; CO mean ≥ 1; IR mean ≥ 1	6/6; 5/6; 5/6	**CO pers D21**	1/6			CO = 2 between D2 and D21 in 1/6; CO = 2 on D3 fully reversed by D7 in 1/6; CO = 2 on D2 fully reversed by D3 in 1/6; CO = 2 between D1 and D3 fully reversed by D7 in 1/6; CO = 2 between D2 and D7 fully reversed by D14 in 1/6; CO = 1 between D1 and D2 fully reversed by D3 in 1/6	X

Study Number	Test Chemical Name	CAS RN	Organic Functional Groups	Physical Form as tested	Physical Form Confirmation e.g., by MSDS (Y / N)	Data Source	Commercial Source	Available Purity	Number of Studies	UN GHS Classification	Drivers of Classification: Severity		Drivers of Classification: Persistence		Drivers of Classification: Specific Observations		Comments	Should Not Be Used
											Cut-off values	Number of animals	Cut-off time	Number of animals	CO = 4 or other observations	Number of animals		
58	Ethoxylated (5 EO) alkyl (C10-14) alcohol	66455-15-0	Alcohol| Alkoxy| Ether	L	Y	EURL ECVAM	Proprietary	90.5%	1 of 1	Cat 1	CO mean ≥ 1; Conj mean ≥ 2	3/3; 3/3	**CO pers D21**	1/3			Delayed effects; CO = 1 between D1 and D6 increasing to CO = 3 on D8 until D21 in 1/3; CO = 2 between D2 and D8 fully reversed by D10 in 1/3; CO = 1 between D1 and D7 fully reversed by D8 in 1/3	X
59	Methyl cellosolve acetate	110-49-6	Acetoxy| Carboxylic acid ester| Ether	L	Y	NICEATM	Sigma-Aldrich	>98%	1 of 1	Cat 1	Conj mean ≥ 2	5/6	**CO pers D21**; Conj pers D21; IR pers D21	1/6; 1/6; 1/6			CO = 2 between D2 and D7 with CO = 2 on D21, IR = 1 between D1 and D21, CR = 3 between D2 and D21 and CC = 4 between D1 and D2 with CC = 2 on D21 in 1/6; CO = 0 between D1 and D21, IR = 1 on D1 fully reversed by D2, CR = 3 on D1 fully reversed by D7 and CC = 2 on D1 fully reversed by D7 in 1/6; CO = 0 between D1 and D21, IR = 0 between D1 and D21, CR = 2 on D1 fully reversed by D14 and CC = 1 on D1 fully reversed by D3 in 1/6; CO = 1 between D1 and D3 fully reversed by D7, IR = 1 between D1 and D3 fully reversed by D7, CR = 3 between D2 and D3 fully reversed by D7 and CC = 4 between D1 and D2 fully reversed by D7 in 1/6; CO = 1 between D1 and D2 fully reversed by D3, IR = 1 between D1 and D2 fully reversed by D3, CR = 3 between D1 and D2 fully reversed by D7 and CC = 3 between D1 and D2 fully reversed by D7 in 1/6; CO = 1 between D1 and D3 fully reversed by D7, IR = 1 between D1 and D2 fully reversed by D3, CR = 3 between D1 and D2 fully reversed by D14 and CC = 3 between D1 and D2 fully reversed by D7 in 1/6	X
60	Methyl pentynol	77-75-8	Alcohol| Alkyne	L	Y	ZEBET	Sigma-Aldrich	98%	1 of 1	Cat 1	CO mean ≥ 1; Conj mean ≥ 2	1/1; 1/1	**CO pers D21** (at least)	1/1	CO = 4	1/1	Study terminated on D14; Delayed effects; CO = 4 on D14 in 1/1; IR = 0 on D7 with unknown score on D14; CR and CC = 2 on D14 in 1/3; Assumed CO irreversible	X
61	Sodium alkyl ether sulphate (~27%, aqueous)	68891-38-3	Alkoxy| Ether| Sulfate	L	Y	EURL ECVAM	Proprietary	~27%, aqueous	1 of 1	Cat 1	CO mean ≥ 1; Conj mean ≥ 2	4/4; 4/4	**CO pers D21**; Conj pers D21	2/4; 1/4			CO = 1 between D1 and D21 and CR = 3 between D1 and D10 with CR = 2 on D21 in 1/4; CO = 1 between D1 and D21 and CR = 3 between D1 and D3 fully reversed by D14 in 1/4; CO = 1 between D1 and D10 fully reversed by D14 and CR = 3 between D1 and D2 fully reversed by D17 in 1/4; CO = 1 between D1 and D7 fully reversed by D10 and CR = 3 on D2 fully reversed by D21 in 1/4	X
62	Sodium coco amphoacetate (~30%, aqueous)	61791-32-0	Alcohol| Aliphatic Amine, tertiary| Carboxamide| Carboxylic acid	L	Y	EURL ECVAM	Proprietary	~30%, aqueous	1 of 1	Cat 1	CO mean ≥ 1	3/4	**CO pers D21**; Conj pers D21	1/4; 1/4			CO = 1 between D1 and D21 and CR = 2 between D1 and D3 and between D14 and D17 with CR = 1 on D21 in 1/4; CO = 1 on D1 fully reversed by D2 and CR = 2 on D2 fully reversed by D3 in 1/4; CO = 1 between D1 and D3 fully reversed by D7 and CR = 2 between D1 and D2 fully reversed by D10 in 1/4; CO = 1 between D1 and D14 fully reversed by D17 and CR = 3 between D2 and D3 fully reversed by D17 in 1/4	X
63	Surfonic HDL-1 (Chemical name: Nonyl phenol ethoxylate, branched)	9016-45-9	Alcohol| Benzyl| Ether	L	Y	NICEATM	Unknown	Unknown	1 of 1	Cat 1	CO mean ≥ 1; Conj mean ≥ 2; IR mean ≥ 1	6/6; 6/6; 6/6	**CO pers D21** (at least)	at least 4/6 (max 5)	CO = 4	5/6	Study terminated on D14; Delayed effects; CO = 4 on D14 in 3/6; CO = 4 between D7 and D14 in 1/6; CO = 4 between D3 and D7 fully reversed by D14 in 1/6; CO = 3 between D1 and D7 with CO = 2 on D14; IR = 1 on D14 in 2/6; IR = 2 on D7 with unknown score on D14 in 1/6; IR = 0 on D14 in 3/6; CR = 2 on D14 in 3/6; CR = 1 on D14 in 3/6; CC = 3 on D14 in 1/6; CC = 2 on D14 in 4/6; CC = 1 on D14 in 1/6; Assumed CO irreversible	?
64	Surfonic N-102 (Chemical name: Nonyl phenol ethoxylate, branched)	9016-45-9	Alcohol| Benzyl| Ether	L	Y	NICEATM	Unknown	Unknown	1 of 1	Cat 1	CO mean ≥ 1; Conj mean ≥ 2; IR mean ≥ 1	6/6; 6/6; 6/6	**CO pers D21** (at least)	at least 2/6 (max 4)	CO = 4	2/6	Study terminated on D14; Delayed effects; CO = 4 on D14 in 2/6; CO = 2 between D1 and D7 fully reversed by D14 in 1/6; CO = 2 between D1 and D7 in 1/6 with animal dying on D11 for reasons unrelated to the test chemical; CO = 2 between D1 and D3 fully reversed by D7 with unknown score on D14 in 1/6; CO = 3 on D7 with unknown score on D14; IR = 1 on D14 in 1/6; IR = 0 on D14 in 4/6; CR = 1 on D14 in 5/6; CC = 3 on D14 in 1/6; CC = 2 on D14 in 4/6; Assumed CO irreversible	?
65	Triethanol ammonium alkyl sulphate (~40%, aqueous)	90583-18-9	Alcohol| Aliphatic Amine, tertiary| Alkoxy| Sulfate	L	Y	EURL ECVAM	Proprietary	~40%, aqueous	1 of 1	Cat 1	CO mean ≥ 1; Conj mean ≥ 2	4/4; 4/4	**CO pers D21**; Conj pers D21	1/4; 1/4			CO = 1 between D1 and D21 and CR = 3 between D1 and D3 with CR = 1 on D21 in 1/4; CO = 1 between D1 and D3 fully reversed by D7 and CR = 3 between D1 and D3 fully reversed by D14 in 1/4; CO = 1 between D1 and D3 fully reversed by D7 and CR = 3 between D1 and D2 fully reversed by D14 in 1/4; CO = 1 between D1 and D7 fully reversed by D10 and CR = 3 between D1 and D2 fully reversed by D14 in 1/4	X
66	Benzalkonium chloride (1%) (1 of 2)	63449-41-2	Ammonium salt| Benzyl	L (tested in solvent, available as S)	Y	ECETOC	Sigma-Aldrich	≥95%	1 of 2	Cat 1	Conj mean ≥ 2	3/4	**CO pers D21**; Conj pers D21	1/4; 1/4			Delayed effects; CO = 3 on D7 with CO = 1 on D21 and CR and CC = 2 between D2 and D7 with CR and CC = 1 on D21 in 1/4; CO = 2 on D2 fully reversed by D7, CR = 2 between D1 and D2 fully reversed by D10 and CC = 3 on D1 fully reversed by D10 in 1/4; CO = 1 between D1 and D2 fully reversed by D3, CR = 2 on D1 fully reversed by D7 and CC = 2 between D1 and D2 fully reversed by D7 in 1/4; CO = 1 on D1 fully reversed by D2, CR = 2 on D1 fully reversed by D10 and CC = 3 on D1 fully reversed by D10 in 1/4	X
67	Benzalkonium chloride (1%) (2 of 2)	63449-41-2	Ammonium salt| Benzyl	L (tested in solvent, available as S)	Y	ECETOC	Sigma-Aldrich	≥95%	2 of 2	Cat 1	CO mean ≥ 1; Conj mean ≥ 2; IR mean ≥ 1	6/6; 6/6; 6/6	**CO pers D21**; Conj pers D21	at least 2/6 (max 3);<br>at least 2/6 (max 3)	CO = 4	1/6	Delayed effects; CO = 4 on D14 with CO = 3 on D21, CC = 3 between D1 and D3 with CC = 1 on D21 in 1/6; CO = 3 on D3 with CO = 2 on D21, CC = 4 on D1 fully reversed by D14 in 1/6; CO = 2 between D1 and D14 fully reversed by D21, CC = 3 between D1 and D3 with CC = 1 on D21 in 1/6; CO =2 between D1 and D10, CC = 4 between D1 and D2 with CC = 2 on D10, study terminated on D10 in 1/6; CO = 2 between D1 and D7 fully reversed by D10, CC = 3 on D1 fully reversed by D10 in 1/6; CO = 2 between D1 and D3 fully reversed by D7, CC = 3 between D1 and D2 fully reversed by D10 in 1/6	X
68	Cetylpyridinium chloride (10%)	6004-24-6	Ammonium salt| Aryl| Pyridine	L (tested in solvent, available as S)	Y	NICEATM	Sigma-Aldrich	>99%	1 of 1	Cat 1	CO mean ≥ 1; Conj mean ≥ 2; IR mean ≥ 1	3/3; 3/3; 3/3	**CO pers D21** (at least)	at least 2/3	CO = 4	3/3	Study terminated on D14; CO = 4 between D7 and D14 in 1/3; CO = 4 between D1 and D14 in 1/3; CO = 4 between D1 and D13 with CO = 3 on D14 in 1/3; IR = 1 on D14 in 3/3; CR = 2 on D14 in 2/3; CR = 1 on D14 in 1/3; CC = 2 on D14 in 1/3; CC = 1 on D14 in 2/3; Assumed CO irreversible	
69	Cetyltrimethyl ammonium bromide (CTAB) (10%) (1 of 2)	57-09-0	Ammonium salt	L (tested in solvent, available as S)	Y	NICEATM	Sigma-Aldrich	≥99%	1 of 2	Cat 1	CO mean ≥ 1; Conj mean ≥ 2; IR mean ≥ 1	3/3; 2/3; 2/3	**CO pers D21** (at least)	3/3	CO = 4	3/3	Study terminated on D14; Delayed effects; CO = 4 between D10 and D14 in 2/3; CO = 4 between D8 and D14 in 1/3; IR = 0 on D14 in 3/3; CR = 3 on D14 in 1/3; CR = 2 on D14 in 2/3; CC = 2 on D14 in 3/3; Assumed CO irreversible	
70	Cetyltrimethyl ammonium bromide (CTAB) (10%) (Hexadecyltrimethylammonium bromide reported in LNS) (2 of 2)	57-09-0	Ammonium salt	L (tested in solvent, available as S)	Y	LNS	Sigma-Aldrich	≥99%	2 of 2	Cat 1	CO mean ≥ 1; Conj mean ≥ 2; IR mean ≥ 1	3/3; 3/3; 3/3	**CO pers D21** (at least)	at least 1/3	CO = 4	1/3	Delayed effects; Persistent corneal lesions; Irreversible lesions on D21 but scores unknown; CO = 4 on D14 in 1/3; CO = 3 between D7 and D14 in 1/3; CO = 3 on D14 in 1/3; IR = 1 on D14 in 2/3; IR = 0 on D14 in 1/3; CR and CC = 3 on D14 in 1/3; CR and CC = 2 on D14 in 2/3	
71	Di(2-ethylhexyl)sodium sulphosuccinate (10%)	577-11-7	Alkane, branched with tertiary carbon| Carboxylic acid ester| Sulfonic acid	L (tested in solvent, available as S)	Y	NICEATM	Sigma-Aldrich	98%	1 of 1	Cat 1	CO mean ≥ 1; Conj mean ≥ 2	3/3; 2/3	**CO pers D21** (at least)	at least 1/3 (max 2)	CO = 4	1/3	Delayed effects; Study terminated on D14; CO = 4 on D14 in 1/3; CO = 3 between D3 and D6 with CO = 1 on D14 in 1/3; CO = 2 between D1 and D9 fully reversed by D12 in 1/3; IR = 0 on D14 in 3/3; CR and CC = 1 on D14 in 1/3; CR and CC = 0 on D14 in 2/3; Assumed CO irreversible	
72	Potassium laurate (10%)	10124-65-9	Carboxylic acid	L (tested in solvent, available as S)	Y	NICEATM	MP Biomedicals	90-100%	1 of 1	Cat 1	CO > 0 **	** CO 1/3; Conj 1/3; IR 1/3	**CO pers D21** (at least)	1/3	CO = 4	1/3	Study terminated on D14 in 1/3 and on D7 with full reversibility of all endpoints in 2/3; Delayed effects; CO = 4 between D7 and D14 in 1/3; CO = 0 between D1 and D7 in 1/3; CO = 1 between D1 and D2 fully reversed by D3 in 1/3; IR = 0 on D14 in 1/3; CR = 2 on D14 in 1/3; CC = 1 on D14 in 1/3; Assumed CO irreversible	X
73	Sodium hydroxide (10%)	1310-73-2	Hydrazine	L (tested in solvent, available as S)	Y	ECETOC	Sigma-Aldrich	≥98%	1 of 1	Cat 1	CO mean ≥ 3; IR mean > 1.5	1/1; 1/1	**CO pers D21**; Conj pers D21; IR pers D21	1/1; 1/1; 1/1	CO = 4	1/1	CO = 4 between D1 and D21, IR = 2 between D1 and D21 and CR and CC = 3 between D1 and D21 in 1/1	(X)
74	Sodium lauryl sulphate (10%) (1 of 3)	151-21-3	Alkoxy| Sulfate	L (tested in solvent, available as S)	Y	NICEATM	Sigma-Aldrich	≥99%	1 of 3	Cat 1	CO mean ≥ 1; Conj mean ≥ 2; IR mean ≥ 1	3/3; 2/3; 2/3	**CO pers D21** (at least)	3/3	CO = 4	3/3	Study terminated on D14; Delayed effects; CO = 4 between D7 and D14 in 2/3; CO = 4 between D5 and D14 in 1/3; IR = 1 on D14 in 2/3; IR = 0 on D14 in 1/3; CR = 3 on D14 in 1/3; CR = 1 on D14 in 2/3; CC = 2 on D14 in 1/3; CC = 1 on D14 in 1/3; CC = 0 on D14 in 1/3; Assumed CO irreversible	X
75	Sodium lauryl sulphate (10%) (2 of 3)	151-21-3	Alkoxy| Sulfate	L (tested in solvent, available as S)	Y	NICEATM	Sigma-Aldrich	≥99%	2 of 3	Cat 1	CO mean ≥ 1; Conj mean ≥ 2	2/3; 2/3	**CO pers D21** (at least)	at least 1/3 (max 2)	CO = 4	1/3	Study terminated on D14 in 2/3 and on D7 with full reversibility of all endpoints in 1/3; Delayed effects; CO = 4 between D5 and D14 in 1/3; CO = 3 between D3 and D14 in 1/3; CO = 1 between D1 and D2 fully reversed by D3 in 1/3; IR = 0 on D14 in 2/3; CR and CC = 2 on D14 in 1/3; CR and CC = 1 on D14 in 1/3; Assumed CO irreversible	X
76	Sodium lauryl sulphate (15%)	151-21-3	Alkoxy| Sulfate	L (tested in solvent, available as S)	Y	ECETOC	Sigma-Aldrich	≥99%	1 of 1	Cat 1	CO mean ≥ 1; Conj mean ≥ 2; IR mean ≥ 1	6/6; 5/6; 4/6	**CO pers D21**	1/6			CO = 2 on D1 with CO = 1 on D21 in 1/6; CO = 2 between D1 and D2 fully reversed by D7 in 3/6; CO = 2 between D1 and D3 fully reversed by D7 in 1/6; CO = 2 on D1 fully reversed by D7 in 1/6	X
77	Stearyltrimethylammonium chloride (10%)	112-03-8	Ammonium salt	L (tested in solvent, available as S)	Y	NICEATM	Sigma-Aldrich	≥95%	1 of 1	Cat 1	CO mean ≥ 1; IR mean ≥ 1; Conj mean ≥ 2	3/3; 3/3; 2/3	**CO pers D21** (at least)	3/3	CO = 4	3/3	Study terminated on D14; Delayed effects; CO = 4 between D2 and D14 in 1/3; CO = 4 between D6 and D14 in 1/3; CO = 4 between D12 and D14 in 1/3; IR = 1 on D14 in 1/3; IR = 0 on D14 in 2/3; CR = 3 on D14 in 1/3; CR = 2 on D14 in 2/3; CC = 2 on D14 in 2/3; CC = 1 on D14 in 1/3; Assumed CO irreversible	
78	Trichloroacetic acid (30%)	76-03-9	Alkyl halide| Carboxylic acid	L (tested in solvent, available as S)	Y	ECETOC	Sigma-Aldrich	>99%	1 of 1	Cat 1	CO mean ≥ 3; IR mean > 1.5	1/1; 1/1	**CO pers D21**; Conj pers D21; IR pers D21	1/1; 1/1; 1/1	CO = 4	1/1	CO = 4 between D1 and D3 with CO = 2 on D21, IR = 2 between D1 and D21, CR = 2 between D1 and D7 with CR = 2 on D21 and CC = 3 between D1 and D3 with CC = 2 on D21 in 1/1	(X)
79	1,2-Dihydro-1,3,4,6-tetramethyl-2-oxo-pyrimidinium hydrogensulphate	54424-29-2	Aliphatic Amine, tertiary| Allyl| Unsaturated heterocyclic amine| Unsaturated heterocyclic fragment	S	Y	EURL ECVAM	Proprietary	89.4%	1 of 1	Cat 1	CO mean ≥ 1; Conj mean ≥ 2; IR mean ≥ 1	3/3; 3/3; 2/3	**CO pers D21**; IR pers D21	2/3; 1/3			CO = 3 between D3 and D14 with CO = 2 on D21 and IR = 1 between D1 and D21 in 1/3; CO = 1 between D1 and D21 and IR = 0 between D1 and D21 in 1/3; CO = 3 on D1 fully reversed by D7 and IR = 1 between D1 and D3 fully reversed by D7 in 1/3	?
80	1-Naphthalene acetic acid	86-87-3	Benzyl| Carboxylic acid| Fused carbocyclic aromatic| Naphtalene	S	Y	ECETOC	Sigma-Aldrich	>95%	1 of 1	Cat 1	CO mean ≥ 1; Conj mean ≥ 2	6/6; 4/6	**CO pers D21**; Conj pers D21; IR pers D21	1/6; 1/6; 1/6	CO = 4	1/6	Delayed effects; CO = 4 between D14 and D21, IR = 2 between D2 and D3 with IR = 1 on D21, CR = 2 between D1 and D3 and between D10 and D21 and CC = 2 between D2 and D3 and between D10 and D21 in 1/6; CO = 2 on D1 fully reversed by D7, IR = 1 on D1 and D3 fully reversed by D7, CR = 2 on D1 fully reversed by D7 and CC = 1 on D1 fully reversed by D2 in 1/6; CO = 1 between D1 and D10 fully reversed by D14, IR = 1 on D1 fully reversed by D2, CR = 2 on D1 fully reversed by D7 and CC = 1 on D1 fully reversed by D2 in 1/6; CO = 1 between D1 and D7 fully reversed by D10, IR = 1 on D1 fully reversed by D2, CR = 2 between D1 and D3 fully reversed by D10 and CC = 1 between D1 and D3 fully reversed by D7 in 1/6; CO = 2 on D1 fully reversed by D21, IR = 2 between D2 and D3 fully reversed by D10, CR = 2 between D1 and D3 fully reversed by D14 and CC = 2 on D1 fully reversed by D7 in 1/6; CO = 2 between D1 and D3 fully reversed by D10, IR = 1 between D1 and D3 fully reversed by D7, CR = 3 on D1 fully reversed by D10 and CC = 1 between D1 and D3 fully reversed by D7 in 1/6	X
81	1-Naphthalene acetic acid Na salt	61-31-4	Benzyl| Carboxylic acid| Fused carbocyclic aromatic| Naphtalene	S	Y	ECETOC	TCI	≥99%	1 of 1	Cat 1	CO mean ≥ 1; IR mean ≥ 1; Conj mean ≥ 2	6/6; 6/6; 4/6	**CO pers D21**; Conj pers D21; IR pers D21	4/6; 4/6; 1/6	CO = 4	1/6	CO = 4 between D1 and D21, IR = 2 between D1 and D21, CR = 2 on D14 with CR = 1 on D21 and CC = 4 between D1 and D2 with CC = 3 on D21 in 1/6; CO = 3 between D1 and D14 with CO = 1 on D21, IR = 1 between D1 and D10 fully reversed by D14, CR = 2 between D1 and D10 with CR = 1 on D21 and CC = 2 between D2 and D3 and between D10 and D14 with CC = 1 on D21 in 1/6; CO = 3 on D1 and between D7 and D10 with CO = 2 on D21, IR = 1 between D1 and D7 fully reversed by D10, CR = 3 on D1 with CR = 1 on D21 and CC = 3 on D1 with CC = 2 on D21 in 1/6; CO = 3 between D2 and D21, IR = 1 between D1 and D7 fully reversed by D10, CR = 1 between D1 and D21 and CC = 2 between D1 and D2 and between D7 and D21 in 1/6; CO = 1 between D1 and D10 fully reversed by D14, IR = 1 between D1 and D7 fully reversed by D10, CR = 2 between D1 and D3 fully reversed by D10 and CC = 2 between D1 and D3 fully reversed by D7 in 1/6; CO = 1 between D1 and D3 fully reversed by D7, IR = 1 between D1 and D7 fully reversed by D10, CR = 2 between D1 and D3 fully reversed by D7 and CC = 1 between D1 and D3 fully reversed by D7 in 1/6	
82	2,7-Naphthalenediol	582-17-2	Fused carbocyclic aromatic| Naphtalene| Phenol	S	Y	EURL ECVAM	Sigma-Aldrich	97%	1 of 1	Cat 1	CO mean ≥ 1; Conj mean ≥ 2; IR mean ≥ 1	1/1; 1/1; 1/1	**CO pers D21**; Conj pers D21	1/1; 1/1			CO = 2 between D1 and D14 with CO = 1 on D21 and CR = 3 between D2 and D3 with CR = 1 on D21 in 1/1	X
83	2-[(4-Amino-2-nitrophenyl)amino]benzoic acid	117907-43-4	Aniline| Aryl| Carboxylic acid| Nitrobenzene	S	Y	Cosmetics Europe	Proprietary	Unknown	1 of 1	Cat 1	CO > 0 **	** CO 2/4; Conj 2/4	**CO pers D21**; Conj pers D21	1/4; 1/4			CO = 1 between D1 and D21 and CR = 2 between D1 and D14 with CR = 1 on D21 in 1/4; CO = 1 and CR = 2 between D1 and D3 fully reversed by D7 in 1/4; CO = 0 between D1 and D21 and CR = 1 between D1 and D2 fully reversed by D3 in 1/4; CO = 0 between D1 and D21 and CR = 1 on D1 fully reversed by D2 in 1/4; IR effects not numerically recorded but the study report states that no IR effects were detected	X

Cosmetics Europe
Draize eye test Reference Database (DRD)
Barroso *et al.* , 2016

Study Number	Test Chemical Name	CAS RN	Organic Functional Groups	Physical Form as tested	Physical Form Confirmation e.g., by MSDS (Y / N)	Data Source	Commercial Source	Available Purity	Number of Studies	UN GHS Classification	Drivers of Classification: Severity: Cut-off values	Severity: Number of animals	Persistence: Cut-off time	Persistence: Number of animals	Specific Observations: CO = 4 or other observations	Specific Observations: Number of animals	Comments	Should Not Be Used
84	2-Benzyl-4-chlorophenol (2 of 2)	120-32-1	Aryl halide| Benzyl| Phenol	S	Y	NICEATM	Sigma-Aldrich	95%	2 of 2	Cat 1	CO mean ≥ 1; Conj mean ≥ 2; IR mean ≥ 1	6/6; 6/6; 6/6	**CO pers D21**; Conj pers D21; IR pers D21	5/6; 5/6; at least 5/6	CO = 4	1/6	Delayed effects; CO = 4 between D4 and D21, IR = 1 between D1 and D3 with remaining scores unknown, CR = 3 between D1 and D21 and CC = 4 between D2 and D21 in 1/6; CO = 3 between D10 and D19 with CO = 2 on D21, IR = 2 between D4 and D14 with IR = 1 on D21, CR = 3 between D2 and D7 with CR = 2 on D21 and CC = 4 between D3 and D4 with CC = 1 on D21 in 1/6; CO = 3 on D4 and between D16 and D21, IR = 2 on D4 with IR = 1 on D21, CR = 3 between D1 and D10 and on D21 and CC = 4 between D2 and D4 with CC = 1 on D21 in 1/6; CO = 3 on D4 and D10 with CO = 1 on D21, IR = 2 between D4 and D14 with IR = 1 on D21, CR = 3 between D1 and D4 fully reversed by D16 and CC = 4 between D2 and D4 fully reversed by D19 in 1/6; CO = 2 between D1 and D16 fully reversed by D21, IR = 2 on D7 with IR = 1 on D21, CR = 3 between D1 and D14 with CR = 2 on D21 and CC = 4 between D3 and D4 with CC = 1 on D21 in 1/6; CO = 3 between D2 and D13 with CO = 2 on D21, IR = 2 between D4 and D14 with IR = 1 on D21, CR = 3 between D1 and D21 and CC = 4 between D3 and D4 and on D10 with CC = 1 on D21 in 1/6	
85	2-Methylresorcinol	608-25-3	Precursors quinoid compounds	S	Y	EURL ECVAM	Sigma-Aldrich	98%	1 of 1	Cat 1	CO mean ≥ 1; Conj mean ≥ 2; IR mean ≥ 1	1/1; 1/1; 1/1	**CO pers D21**; Conj pers D21	1/1; 1/1			CO = 3 between D1 and D2 with CO = 1 on D21 and CR = 3 between D1 and D7 with CR = 2 on D21 in 1/1	X
86	3,4-Dichlorophenyl isocyanate	102-36-3	Aryl halide| Isocyanate	S	Y	NICEATM	Sigma-Aldrich	97%	1 of 1	Cat 1	Conj mean ≥ 2	2/3	**CO pers D21**	3/3			Delayed effects; CO = 2 on D7 with CO = 1 on D21 in 1/3; CO = 1 between D7 and D21 in 1/3; CO = 1 between D2 and D21 in 1/3	
87	Acid Red 92 (Chemical name: 3,4,5,6-Tetrachloro-2-(1,4,5,8-tetrabromo-6-hydroxy-3-oxoxanthen-9-yl)-benzoic acid) (neat)	18472-87-2	Aromatic perhalogencarbons| Aryl halide| Fused carbocyclic aromatic| Fused saturated heterocycles| Heterocyclic spiro rings| Isobenzofuran| Lactone| Phenol| Xanthene	S	Y	NICEATM	Sigma-Aldrich	≥80%	1 of 1	Cat 1	CO mean ≥ 1	3/3	**CO pers D21** (at least)	at least 2/3	CO = 4	3/3	Study terminated on D14; Delayed effects; CO = 4 between D10 and D14 in 1/3; CO = 4 between D4 and D14 in 1/3; CO = 4 between D6 and D8 with CO = 3 on D14 in 1/3; IR = 0 on D14 in 3/3; CR = 2 on D14 in 1/3; CR = 1 on D14 in 2/3; CC = 2 on D14 in 1/3; CC = 1 on D14 in 1/3; CC = 0 on D14 in 1/3; Assumed CO irreversible	
88	Captan 90-concentrate	133-06-2	Alkyl halide| Allyl| Sulfen amide| Tetrahydrophthalimide	S	Y	ECETOC	TCI	>98%	1 of 1	Cat 1	CO mean ≥ 1; Conj mean ≥ 2; IR mean ≥ 1	3/3; 3/3; 3/3	**CO pers D21**; Conj pers D21; IR pers D21	3/3; 2/3; 1/3	CO = 4	3/3	Delayed effects; CO = 4 between D3 and D21, IR = 2 between D10 and D21, CR = 3 between D1 and D21 and CC = 4 on D1 and D3 with CC = 1 on D21 in 1/3; CO = 4 between D2 and D3 with CO = 2 on D21, IR = 1 between D1 and D3 fully reversed by D7, CR = 3 between D1 and D7 fully reversed by D15 and CC = 3 between D1 and D2 fully reversed by D15 in 1/3; CO = 4 between D7 and D10 with CO = 3 on D21, IR = 2 on D10 fully reversed by D15, CR = 3 between D1 and D10 with CR = 1 on D21 and CC = 3 between D1 and D3 fully reversed by D21 in 1/3	
89	Imidazole (2 of 2)	288-32-4	Aryl| Imidazole	S	Y	LNS	Sigma-Aldrich	≥99%	2 of 2	Cat 1	CO mean ≥ 1; Conj mean ≥ 2; IR mean ≥ 1	3/3; 3/3; 3/3	**CO pers D21** (at least)	at least 1/3			Delayed effects; Irreversible lesions on D21 but scores unknown; CO = 2 between D1 and D14 in 1/3; CO = 3 on D14 in 2/3; IR = 0 on D14 in 3/3; CR = 2 on D14 in 2/3; CR = 0 on D14 in 1/3; CC = 2 on D14 in 3/3	
90	Lauric acid	143-07-7	Carboxylic acid	S	Y	ECETOC	Sigma-Aldrich	>99%	1 of 1	Cat 1	Conj mean ≥ 2; CO mean ≥ 1	3/3; 2/3	**CO pers D21**; Conj pers D21	3/3; 3/3			CO = 2 between D7 and D21 and CR = 3 between D1 and D7 with CR = 1 on D21 in 1/3; CO = 2 between D7 and D21 and CR = 3 between D1 and D2 with CR = 1 on D21 in 1/3; CO = 2 between D7 and D14 with CO = 1 on D21 and CR = 3 between D1 and D7 with CR = 1 on D21 in 1/3	
91	m-Phenylene diamine (neat)	108-45-2	Aminoaniline, meta	S	Y	NICEATM	Sigma-Aldrich	≥99%	1 of 1	Cat 1	CO mean ≥ 1; Conj mean ≥ 2; IR mean ≥ 1	3/3; 3/3; 2/3	**CO pers D21** (at least)	3/3	CO = 4	3/3	Study terminated on D14; Delayed effects; CO = 4 between D1 and D14 in 1/3 (CO = 3 on D13); CO = 4 between D11 and D14 in 1/3; CO = 4 between D9 and D14 in 1/3; IR = 0 on D14 in 3/3; CR = 2 on D14 in 1/3; CR = 1 on D14 in 2/3; CC = 1 on D14 in 2/3; CC = 0 on D14 in 1/3; Assumed CO irreversible	
92	N-(2-Amino-4,6-dichloropyrimidin-5-yl) formamide	171887-03-9	Aromatic heterocyclic halide| Aryl halide| Formylamino	S	Y	EURL ECVAM	Chemos GmbH	99.5%	1 of 1	Cat 1	CO > 0 **	** CO 2/4; IR 2/4; Conj 1/4	**CO pers D21**	1/4			CO = 2 between D2 and D3 with CO = 1 on D21 in 1/4; CO = 0 between D1 and D3 in 2/4; CO = 1 between D1 and D3 fully reversed by D7 in 1/4	X
93	Pinoxaden	243973-20-8	Carboxylic acid ester| Ether (cyclic)| Heterocyclic fragment| Hydrazide| Lactam	S	Y	EURL ECVAM	Sigma-Aldrich	analytical standard	1 of 1	Cat 1	CO mean ≥ 1; Conj mean ≥ 2	3/3; 3/3	**CO pers D21**; Conj pers D21	1/3; 1/3			Delayed effect; CO = 3 on D17 with CO = 2 on D21 fully reversed by D28, CR = 2 on D3 with CR = 1 on D21 and CC = 3 between D1 and D3 with CC = 1 on D21 in 1/3; CO = 1 between D1 and D7 fully reversed on D10, CR = 2 on D3 fully reversed by D17 and CC = 3 between D1 and D2 fully reversed by D7 in 1/3; CO = 1 between D1 and D3 fully reversed on D7, CR = 1 between D1 and D7 fully reversed by D10 and CC = 3 on D1 fully reversed by D7 in 1/3	X
94	p-tert-Butylphenol (neat) (10 mg)	98-54-4	Phenol| tert-Butyl	S	Y	NICEATM	Sigma-Aldrich	≥99%	1 of 1	Cat 1	CO mean ≥ 1; IR mean ≥ 1	6/6; 4/6	**CO pers D21**; Conj pers D21	4/6; 1/6	CO = 4	1/6	Delayed effects; 10 mg dose; CO = 4 between D14 and D21, IR = 1 between D1 and D3 with remaining scores unknown, CR = 2 on D14 with CR = 1 on D21 and CC = 2 between D1 and D2 and on D14 with CC = 1 on D21 in 1/6; CO = 3 on D3 with CO = 1 on D21, IR = 1 between D1 and D3 fully reversed by D7, CR = 1 between D1 and D3 fully reversed by D7 and CC = 2 between D1 and D3 fully reversed by D14 in 1/6; CO = 2 between D1 and D7 with CO = 1 on D21, IR = 1 between D1 and D3 fully reversed by D7, CR = 1 between D1 and D3 fully reversed by D7 and CC = 2 on D1 fully reversed by D7 in 1/6; CO = 2 between D1 and D7 with CO = 1 on D21, IR = 1 between D1 and D3 fully reversed by D7, CR = 2 on D3 fully reversed by D7 and CC = 1 between D1 and D3 fully reversed by D7 in 1/6; CO = 2 on D1 and D3 fully reversed by D14, IR = 1 between D1 and D3 fully reversed by D7, CR = 1 between D1 and D3 fully reversed by D7 and CC = 3 on D1 fully reversed by D7 in 1/6; CO = 1 between D1 and D7 fully reversed by D14, IR = 1 on D1 fully reversed by D2, CR = 1 between D1 and D3 fully reversed by D7 and CC = 2 on D1 fully reversed by D7 in 1/6	
95	p-tert-Butylphenol (neat) (80 mg)	98-54-4	Phenol| tert-Butyl	S	Y	NICEATM	Sigma-Aldrich	≥99%	1 of 1	Cat 1	CO mean ≥ 1; Conj mean ≥ 2	6/6; 6/6	**CO pers D21**; Conj pers D21	5/6; 1/6	CO = 4	4/6	Delayed effects; 80 mg dose; CO = 4 between D7 and D21, IR scores unknown and CC = 4 on D1 with CC = 1 on D21 in 1/6; CO = 4 between D7 and D14 with CO = 3 on D21, IR = 1 on D1 with remaining scores unknown and CC = 4 on D1 and D3 fully reversed by D21 in 1/6; CO = 4 between D7 and D14 with CO = 3 on D21, IR scores unknown and CC = 4 on D1 and D3 fully reversed by D21 in 1/6; CO = 4 between D7 and D14 with CO = 3 on D21, IR scores unknown and CC = 4 on D1 and D3 fully reversed by D21 in 1/6; CO = 3 between D1 and D2 with CO = 2 on D21, IR scores unknown between D1 and D14 but fully reversed by D21 and CC = 4 on D1 and D3 fully reversed by D14 in 1/6; CO = 2 between D1 and D3 fully reversed by D14, IR = 1 between D1 and D3 fully reversed by D7 and CC = 4 on D1 and D3 fully reversed by D14 in 1/6	
96	Quinacrine (2 of 2)	69-05-6	Acridine| Aliphatic Amine, tertiary| Aryl halide| Ether	S	Y	LNS	Sigma-Aldrich	98%	2 of 2	Cat 1	CO mean ≥ 1; Conj mean ≥ 2; IR mean ≥ 1	3/3; 3/3; 3/3	**CO pers D21**; IR pers D21 (at least)	at least 1/3;<br>at least 1/3	CO = 4	2/3	Delayed and persistent IR effects; Irreversible lesions on D21 but scores unknown; CO = 4 between Hr1 and D1 with CO = 3 on D14 and IR = 2 on D14 in 1/3; CO = 4 on Hr1 with CO = 3 on D14 and IR = 2 on D14 in 1/3; CO = 2 between D1 and D14 and IR = 2 on D14 in 1/3; CR and CC =2 on D14 in 3/3	
97	Soap from 80/20 tallow/coconut oil	Mixture	N/A	S	Y	ECETOC	Unknown	Unknown	1 of 1	Cat 1	Conj mean ≥ 2	3/3	**CO pers D21**; Conj pers D21	1/3; 1/3			Very slight opacity (0.5) between D1 and D14 with CO = 1 on D21 and CR = 3 between D1 and D7 with CR = 1 on D21 in 1/3; Very slight opacity (0.5) between D1 and D14 fully reversed by D21 and CR = 3 between D1 and D3 fully reversed by D14 in 1/3; Very slight opacity (0.5) between D1 and D14 fully reversed by D21 and CR = 3 between D2 and D3 fully reversed by D14 in 1/3	X
98	Sodium oxalate (1 of 2)	62-76-0	Oxocarboxylic acid	S	Y	LNS	Sigma-Aldrich	≥99.5%	1 of 2	Cat 1	CO mean ≥ 1; Conj mean ≥ 2; IR mean ≥ 1	3/3; 3/3; 3/3	**CO pers D21** (at least)	1/3	CO = 4	1/3	Irreversible lesions on D21 in 1/3 but scores unknown; CO = 4 between D3 and D14 irreversible on D21 (score unknown), IR = 1 between D1 and D7 fully reversed by D14, CR = 3 between D1 and D7 with CR = 2 on D14 and CC = 4 between D1 and D7 with CC = 2 on D14 in 1/3; CO = 2 between D1 and D7 fully reversed by D14, IR = 1 between D1 and D4 fully reversed by D7, CR = 3 between D1 and D4 with CR = 1 on D14 and CC = 4 on D1 and between D3 and D4 with CC = 1 on D14 in 1/3; CO = 2 between D1 and D7 fully reversed by D14, IR = 1 between D1 and D3 fully reversed by D4, CR = 3 between D1 and D4 fully reversed by D14 and CC = 4 between D1 and D4 fully reversed by D14 in 1/3	X
99	Sodium oxalate (2 of 2)	62-76-0	Oxocarboxylic acid	S	Y	ECETOC	Sigma-Aldrich	≥99.5%	2 of 2	Cat 1	CO mean ≥ 1; Conj mean ≥ 2; IR mean ≥ 1	3/3; 3/3; 3/3	**CO pers D21** (at least)	at least 1/3 (max 2)	CO = 4	1/3	Study terminated on D14; Delayed effects; CO = 4 between D7 and D14, IR = 2 between D2 and D3 and on D14, CR = 3 between D1 and D14 and CC = 4 between D1 and D3 with CC = 3 on D14 in 1/3; CO = 3 between D3 and D7 with CO = 2 on D14, IR = 1 between D1 and D7 fully reversed by D14, CR = 3 between D1 and D7 fully reversed by D14 and CC = 4 between D1 and D3 fully reversed by D14 in 1/3; CO = 2 between D1 and D7 fully reversed by D14, IR = 1 between D1 and D3 fully reversed by D4, CR = 3 between D1 and D4 fully reversed by D14 and CC = 3 on D2 fully reversed by D14 in 1/3; Assumed CO irreversible	X
100	Sodium perborate tetrahydrate	10486-00-7	N/A	S	Y	ECETOC	Sigma-Aldrich	≥97%	1 of 1	Cat 1	Conj mean ≥ 2; CO mean ≥ 1; IR mean ≥ 1	6/6; 5/6; 4/6	**CO pers D21**; Conj pers D21	4/6; 2/6	CO = 4	2/6	Delayed effects; CO = 4 between D14 and D21 and CR = 3 between D1 and D7 with CR = 1 on D21 in 1/6; CO = 4 on D21 and CR = 3 between D1 and D7 fully reversed by D21 in 1/6; CO = 2 between D1 and D7 with CO = 1 on D21 and CR = 3 between D1 and D7 fully reversed by D21 in 1/6; CO = 2 between D2 and D14 with CO = 1 on D21 and CR = 3 between D1 and D7 with CR = 1 on D21 in 1/6; CO = 2 between D1 and D3 fully reversed by D14 and CR = 3 between D1 and D3 fully reversed by D21 in 1/6; CO = 0 between D1 and D21 and CR = 3 between D1 and D3 fully reversed by D14 in 1/6	
101	Sodium salicylate (neat)	54-21-7	Carboxylic acid| Phenol	S	Y	NICEATM	Sigma-Aldrich	≥99.5%	1 of 1	Cat 1	CO mean ≥ 1; Conj mean ≥ 2	3/3; 3/3	**CO pers D21** (at least)	at least 2/3	CO = 4	3/3	Study terminated on D14; CO = 4 between D4 and D14 in 2/3; CO = 4 between Hr1 and D13 with CO = 3 on D14 in 1/3; IR = 0 on D14 in 3/3; CR = 2 on D14 in 2/3; CR = 0 on D14 in 1/3; CC = 2 on D14 in 2/3; CC = 1 on D14 in 1/3; Assumed CO irreversible	
102	3,6-Dimethyloctanol	151-19-9	Alcohol| Alkane, branched with tertiary carbon	Unknown	N	ZEBET	Sigma-Aldrich	Unknown	1 of 1	Cat 1	CO mean ≥ 1	3/3	**CO pers D21**; Conj pers D21	1/3; 1/3			CO = 1 between D1 and D14 with CO = 2 on D21 and CR = 2 between D1 and D3 with CR = 1 on D21 in 1/3; CO = 1 between D1 and D3 fully reversed by D7 and CR = 2 between D1 and D2 fully reversed by D7 in 1/3; CO = 1 between D1 and D3 fully reversed by D7 and CR = 1 between D1 and D3 fully reversed by D7 in 1/3	X
103	7-Acetoxyheptanal	29425-54-5	Acetoxy| Aldehyde| Carboxylic acid ester	Unknown	N	ZEBET	Unknown	Unknown	1 of 1	Cat 1	CO mean ≥ 1; Conj mean ≥ 2	3/3; 3/3	**CO pers D21**; Conj pers D21	1/3; 1/3			Delayed effects; CO = 3 between D14 and D21, CR = 2 between D1 and D21 and CC = 2 on D1, D3 and D14 with CC = 1 on D21 in 1/3; CO = 1 between D1 and D3 fully reversed by D7, CR = 3 between D1 and D3 fully reversed by D14 and CC = 2 between D1 and D3 fully reversed by D7 in 1/3; CO = 1 between D1 and D7 fully reversed by D14, CR = 2 between D1 and D3 fully reversed by D7 and CC = 2 between D1 and D3 fully reversed by D7 in 1/3	X
104	AU-358 LTV	Proprietary to Sewer Sciences Inc.	N/A	Unknown	N	NICEATM	Unknown	Unknown	1 of 1	Cat 1	CO mean ≥ 1; Conj mean ≥ 2; IR mean ≥ 1	3/3; 3/3; 2/3	**CO pers D21**	3/3	CO = 4	1/3	CO = 4 between D7 and D10 with CO = 1 on D21 in 1/3; CO = 1 between D1 and D21 in 2/3	?
105	C12/C14-Glucoside	Unknown	N/A	Unknown	N	ZEBET	Unknown	Unknown	1 of 1	Cat 1	CO mean ≥ 1; Conj mean ≥ 2	3/3; 3/3	**CO pers D21**; Conj pers D21	3/3; 3/3			CO = 2 between D7 and D21, CR = 2 between D1 and D21 and CC = 2 between D1 and D14 with CC = 1 on D21 in 1/3; CO = 2 on D3 and D21, CR = 2 between D1 and D7 with CR = 2 on D21 and CC = 2 between D1 and D3 with CC = 2 on D21 in 1/3; CO = 2 on D21, CR = 2 between D1 and D3 with CR = 2 on D21 and CC = 1 between D1 and D3 fully reversed by D7	?
106	Diphocars	154836-02-9	N/A	Unknown	N	ZEBET	Unknown	Unknown	1 of 1	Cat 1	CO mean ≥ 1; Conj mean ≥ 2; IR mean ≥ 1	1/1; 1/1; 1/1	**CO pers D21** (at least)	1/1	CO = 4	1/1	Study terminated on D14; Delayed effects; CO = 4, IR = 2, CR = 3 and CC = 1 on D14 in 1/1; Assumed CO irreversible	X
107	Polyhexamethylene guanidine	31961-54-3	Guanidine	Unknown	N	ZEBET	Unknown	Unknown	1 of 1	Cat 1	CO mean ≥ 1; Conj mean ≥ 2	3/3; 2/3	**CO pers D21**; Conj pers D21	1/3; 1/3	CO = 4	1/3	Delayed effects; CO = 4 on D21, IR = 1 between D1 and D4 with IR = 0 on D14 and scores on D7 and D21 unknown, CR = 3 between D2 and D7 with CR = 2 on D21 and CC = 2 between D1 and D14 with CC = 1 on D21 in 1/3; CO = 2 between D3 and D4 fully reversed by D14, IR = 1 between D3 and D7 fully reversed by D14, CR = 1 between D1 and D7 fully reversed by D14 and CC = 1 between D1 and D7 fully reversed by D14 in 1/3; CO = 1 between D1 and D7 fully reversed by D14, IR = 1 between D1 and D4 fully reversed by at least D14 (score on D7 unknown), CR = 3 between D2 and D7 fully reversed by D14 and CC = 2 on D2 and between D4 and D7 fully reversed by D14 in 1/3	X
108	(Ethylenediamine propyl)trimethoxysilane	1760-24-3	Aliphatic Amine, primary| Aliphatic Amine, secondary| AlkoxySilane	L	Y	EURL ECVAM	Sigma-Aldrich	≥80%	1 of 1	Cat 1	Conj mean ≥ 2; IR mean ≥ 1; CO mean ≥ 1	3/3; 3/3; 2/3	**Conj pers D21**; CO pers D21	3/3; 2/3			CO = 2 on D4 with CO = 1 on D21, CR = 3 on D1 with CR = 1 on D21 and CC = 3 on D1 fully reversed by D14 in 1/3; CO = 1 between D1 and D21, CR = 3 on D1 with CR = 1 on D21 and CC = 2 between D1 and D4 fully reversed by D7 in 1/3; CO = 3 on D7 fully reversed by D21, CR = 3 between D2 and D7 with CR = 1 on D21 and CC = 2 between D1 and D7 with CC = 1 on D21 in 1/3	

Study Number	Test Chemical Name	CAS RN	Organic Functional Groups	Physical Form as tested	Physical Form Confirmation e.g., by MSDS (Y / N)	Data Source	Commercial Source	Available Purity	Number of Studies	UN GHS Classification	Drivers of Classification: Severity – Cut-off values	Drivers of Classification: Severity – Number of animals	Drivers of Classification: Persistence – Cut-off time	Drivers of Classification: Persistence – Number of animals	Drivers of Classification: Specific Observations – CO = 4 or other observations	Drivers of Classification: Specific Observations – Number of animals	Comments	Should Not Be Used
109	Alkyl (C10-16) glucoside (~50%, aqueous)	110615-47-9	Dihydroxyl group	L	Y	EURL ECVAM	Proprietary	~50%, aqueous	1 of 1	Cat 1	CO > 0 **	** CO 2/4; Conj 2/4	**Conj pers D21**; CO pers D21	2/4; 1/4			CO = 1 between D1 and D21, CR = 3 between D1 and D2 with CR = 1 on D21 and CC = 2 between D1 and D2 with CC = 1 on D21 in 1/4; CO = 1 between D1 and D17 fully reversed by D21, CR = 3 on D1 (scores of D2 and D3 unknown) with CR = 1 on D21 and CC = 2 on D1 (score of D2 unknown) fully reversed by D21 in 1/4; CO = 0 between D1 and D21, CR = 2 on D1 fully reversed by D10 and CC = 1 between D1 and D2 fully reversed by D3 in 1/4; CO = 0 between D1 and D21, CR = 2 on D1 fully reversed by at least D7 (score of D3 unknown) and CC = 1 between D1 and D2 fully reversed by at least D7 (score of D3 unknown) in 1/4	X
110	Butoxyethanol (Butyl cellosolve reported in ECETOC) (2 of 3)	111-76-2	Alcohol| Alkoxy| Ether	L	Y	ECETOC	Sigma-Aldrich	≥99%	2 of 3	Cat 1	CO mean ≥ 1; Conj mean ≥ 2; IR mean ≥ 1	3/3; 3/3; 3/3	**Conj pers D21**	3/3			CR = 3 between D1 and D3 with CR = 1 on D21 and CC = 3 between D1 and D3 fully reversed by D14 in 1/3; CR = 2 between D1 and D3 with CR = 1 on D21 and CC = 3 between D1 and D3 fully reversed by D21 in 1/3; CR = 2 between D1 and D7 fully reversed by D21 and CC = 3 on D1 with CC = 1 on D21 in 1/3	X
111	Protectol PP	80-54-6	Aldehyde| Benzyl| tert-Butyl	L	Y	NICEATM	Sigma-Aldrich	analytical standard	1 of 1	Cat 1	CO mean ≥ 1; Conj mean ≥ 2	3/3; 3/3	**Conj pers D21**; CO pers D21	3/3; 2/3			CO = 1 between D1 and D21 and CR = 2 between D1 and D21 in 1/3; CO = 2 between D8 and D17 with CO = 1 on D21 and CR = 3 between D17 and D21 in 1/3; CO = 1 between D1 and D3 fully reversed by D8 and CR = 2 between D1 and D3 with CR = 1 on D21	
112	Triton X-100 (10%)	9002-93-1	Alcohol| Alkane branched with quaternary carbon| Aryl| Ether| tert-Butyl	L	Y	ECETOC	Sigma-Aldrich	laboratory grade	1 of 1	Cat 1	CO mean ≥ 1; Conj mean ≥ 2; IR mean ≥ 1	6/6; 6/6; 6/6	**Conj pers D21**	1/6			CR = 2 between D1 and D3 with CR = 1 on D21 in 1/6; CR = 2 between D1 and D3 fully reversed by D10 in 1/6; CR = 2 between D1 and D3 fully reversed by D14 in 4/6	X
113	Igepon AC-78 (10%) (Chemical name: Sodium cocoyl isethionate)	58969-27-0 (original CAS # deleted and replaced by 61789-32-0)	Carboxylic acid ester| Sulfonic acid	L (tested in solvent, neat chemical unknown)	Y	ECETOC	Unknown	Unknown	1 of 1	Cat 1	CO mean ≥ 1	6/6	**Conj pers D21**	1/6			CR = 3 on D1 with CR = 1 on D21 in 1/6; CR = 2 between D1 and D2 fully reversed by D10 in 2/6; CR = 2 on D1 fully reversed by D14 in 1/6; CR = 2 on D1 fully reversed by D7 in 1/6; CR = 3 on D1 fully reversed by D10 in 1/6	X
114	2,5-Dimethylhexanediol	110-03-2	Dihydroxyl group	S	Y	ECETOC / ZEBET	Sigma-Aldrich	97%	1 of 1	Cat 1	CO mean ≥ 1; Conj mean ≥ 2	3/3; 3/3	**Conj pers D21**; IR pers D21	1/3; 1/3			IR = 1 between D1 and D21, CR = 3 between D1 and D3 with CR = 2 on D21 and CC = 3 between D1 and D2 with CC = 1 on D21 in 1/3; IR = 0 between D1 and D21, CR = 2 between D1 and D3 fully reversed by D15 and CC = 2 between D1 and D2 fully reversed by D8 in 1/3; IR = 0 between D1 and D21, CR = 2 between D1 and D3 fully reversed by D15 and CC = 2 on D1 fully reversed by D8; Two studies are reported in ECETOC and ZEBET with the exact same animal scores and are considered duplicate entries	X
115	2-[(4-Aminophenyl)azo]-1,3-dimethyl-1H-imidazolium chloride	97404-02-9	Ammonium salt| Aniline| Aryl| Azo| Guanidine| Imidazole	S	Y	EURL ECVAM	Sigma-Aldrich	93.4%	1 of 1	Cat 1	Conj mean ≥ 2; IR mean ≥ 1; CO mean ≥ 1	3/3; 3/3; 2/3	**Conj pers D21** (at least)	at least 1/3			CR = 3 between D2 and D4 with CR = 1 on D21 in 1/3; CR = 3 between D2 and D4 (study terminated on D4) in 2/3	X
116	3,4-Dimethyl-1H-pyrazole	2820-37-3	Allyl| Aryl| Pyrazole	S	Y	EURL ECVAM	Sigma-Aldrich	≥97%	1 of 1	Cat 1	CO mean ≥ 1; IR mean ≥ 1; Conj mean ≥ 2	3/3; 3/3; 2/3	**Conj pers D21**; CO pers D21; IR pers D21	3/3; 2/3; 2/3			CO = 3 between D14 and D21, IR = 2 between D7 and D21 and CR = 2 on D7 with CR = 1 on D21 in 1/3; CO = 2 between D1 and D21, IR = 2 between D1 and D7 with IR = 2 on D21 and CR = 2 on D7 with CR = 1 on D21 in 1/3; CO = 1 between D1 and D7 fully reversed by D14, IR = 1 between D1 and D14 fully reversed by D21 and CR = 2 between D1 and D7 with CR = 1 on D21 in 1/3	
117	5,6-Dihydroxyindoline HBr	138937-28-7	Indoline| Precursors quinoid compounds	S	Y	EURL ECVAM	Chemos GmbH	99.7%	1 of 1	Cat 1	Conj mean ≥ 2; IR mean ≥ 1	3/3; 2/3	**Conj pers D21**	2/3			CR = 3 between D1 and D21 in 2/3; CR = 3 between D1 and D7 fully reversed by D21 in 1/3	
118	5-Chloro-2-(4-chlorophenoxy)phenol	3380-30-1	Arene| Aryl halide| Phenol	S	Y	EURL ECVAM	Proprietary	Unknown	1 of 1	Cat 1	Conj mean ≥ 2	2/3	**Conj pers D21**; CO pers D21	2/3; 1/3			CO = 1 between D2 and D21 and CR = 1 between D1 and D21 in 1/3; CO = 1 between D2 and D7 fully reversed by D14 and CR = 2 between D1 and D3 with CR = 1 on D21 in 1/3; CO = 0 between D1 and D21 and CR = 2 between D1 and D3 fully reversed by D14 in 1/3	X
119	Aminopyralid	150114-71-9	Aromatic heterocyclic halide| Aryl halide| Carboxylic acid	S	Y	EURL ECVAM	Sigma-Aldrich	analytical standard	1 of 1	Cat 1	CO mean ≥ 1; Conj mean ≥ 2; IR mean ≥ 1	3/3; 3/3; 2/3	**Conj pers D21**; CO pers D21	3/3; 1/3	Other observations	3/3	CO = 2 between D1 and D21, CR = 3 on D3 with CR = 2 on D21 and CC = 3 between D1 and D3 with CC = 2 on D21 in 1/3; CO = 2 between D1 and D14 fully reversed by D21, CR = 3 on D1 with CR = 1 on D21 and CC = 2 on D1 fully reversed by D21 in 1/3; CO = 2 on D1 fully reversed by D21, CR = 3 between D1 and D7 with CR = 1 on D21 and CC = 3 on D1 fully reversed by D21 in 1/3; Pannus formation in 3/3 reversible in at least 2/3	X
120	Benzoic acid	65-85-0	Aryl| Carboxylic acid	S	Y	EURL ECVAM	Sigma-Aldrich	≥99.5%	1 of 1	Cat 1	Conj mean ≥ 2; CO mean ≥ 1	3/3; 2/3	**Conj pers D21**; CO pers D21	3/3; 2/3			CO = 3 between D1 and D3 with CO = 2 on D21, IR = 2 on D14 with sore on D21 unknown, CR = 3 between D1 and D21 and CC = 2 between D1 and D2 and on D7 with CC = 1 on D21 in 1/3; CO = 1 between D1 and D21, IR = 1 on D2 fully reversed by D3, CR = 3 between D1 and D21 and CC = 2 between D1 and D7 with CC = 1 on D21 in 1/3; CO = 1 between D2 and D3 fully reversed by D7, IR = 0 between D1 and D21, CR = 3 between D1 and D7 with CR = 1 on D21 and CC = 2 on D1 and D3 fully reversed by D14 in 1/3	
121	Disodium 2,2'-([1,1'-biphenyl]-4,4'-diyldivinylene) bis(benzene sulphonate)	27344-41-8	Alkene| Aryl| Biphenyl| Sulfonic acid	S	Y	EURL ECVAM	TCI	>97%	1 of 1	Cat 1	CO mean ≥ 1; Conj mean ≥ 2	3/3; 3/3	**Conj pers D21**	2/3			CR = 2 between D1 and D3 with CR = 1 on D21 in 1/3; CR = 2 between D1 and D7 with CR = 1 on D21 in 1/3; CR = 2 between D1 and D3 fully reversed by D14 in 1/3	X
122	Soap from 80/20 palm oil/coconut oil	Mixture	N/A	S	Y	ECETOC	Unknown	Unknown	1 of 1	Cat 1	Conj mean ≥ 2; CO mean ≥ 1	3/3; 2/3	**Conj pers D21**	2/3			CR = 3 between D1 and D2 with CR = 1 on D21 in 2/3; CR = 3 between D2 and D3 fully reversed by D21 in 1/3	X
123	(3-Aminopropyl)triethoxy silane (1 of 2)	919-30-2	Aliphatic Amine, primary| Alkoxy| AlkoxySilane	L	Y	NICEATM	Sigma-Aldrich	99%	1 of 2	Cat 1	NA		Unknown		**CO = 4**	2/3	Study terminated on D1	
124	(3-Aminopropyl)triethoxy silane (2 of 2)	919-30-2	Aliphatic Amine, primary| Alkoxy| AlkoxySilane	L	Y	NICEATM	Sigma-Aldrich	99%	2 of 2	Cat 1	CO mean ≥ 1; Conj mean ≥ 2; IR mean ≥ 1	6/6; 6/6; at least 5/6	Unknown		**CO = 4**	6/6	Delayed effects; Study terminated on D7 with CO = 4 in 5/6 and CO = 2 in 1/6; CO = 4 on D7 in 3/6, between Hr1 and D7 in 1/6, between D2 and D7 in 1/6 and between Hr1 and H4 in 1/6	
125	Methoxyethyl acrylate	3121-61-7	Acrylate| Ether	L	Y	ECETOC	Sigma-Aldrich	98%	1 of 1	Cat 1	Conj mean ≥ 2; CO mean ≥ 1	3/3; 2/3	Unknown		**CO = 4**	2/3	CO = 4 on D1 in 2/3 fully reversed by D3 in 1/3; Study terminated for the other animal on D14 with CO = 1	X
126	Methyl thioglycolate	2365-48-2	Carboxylic acid ester| Thioalcohol	L	Y	ECETOC	Sigma-Aldrich	95%	1 of 1	Cat 1	CO mean ≥ 1; Conj mean ≥ 2; IR mean ≥ 1	3/3; 3/3; 2/3			**CO = 4**	1/3	CO = 4 between Hr1 and D1 in 1/3 fully reversed by D10; Animal 1 with full reversibility of all endpoints on D6 (CO on D3, max CO = 3), animal 2 on D7 (CO on D7, max CO = 3) and animal 3 on D10 (CO on D10, max CO = 4)	X
127	n-Butanol (10%)	71-36-3	Alcohol	L	Y	NICEATM	Sigma-Aldrich	99.8%	1 of 1	Cat 1	CO mean ≥ 1	2/3	Unknown		**CO = 4**	1/3	Study terminated on D14 with CO = 1 in 1/3; CO = 4 in this animal between D3 and D9, which reduces to score 1 on D13; No corneal effects on one of the other animals and CO = 1 in the last animal fully reversed by D5	X
128	n-Butanol (neat) (1 of 2)	71-36-3	Alcohol	L	Y	ZEBET	Sigma-Aldrich	99.8%	1 of 2	Cat 1	CO mean ≥ 1; Conj mean ≥ 2; IR mean ≥ 1	3/3; 3/3; at least 2/3	Unknown		**CO = 4**	1/3	Study terminated on D7 with CO = 4 in 1/3; CO = 4 between D2 and D7	X
129	n-Octylamine	111-86-4	Aliphatic Amine, primary	L	Y	NICEATM	Sigma-Aldrich	≥99.5%	1 of 1	Cat 1	NA		Unknown		**CO = 4**	4/4	Study terminated on D1	
130	Polyethylene glycol 400, dichloride	27252-69-3	N/A	L	Y	NICEATM	Unknown	Unknown	1 of 1	Cat 1	Conj mean ≥ 2; IR mean ≥ 1; CO mean ≥ 1	6/6; 5/6; 4/6	Unknown		**CO = 4**	4/6	Delayed effects; Study terminated on D7 with CO = 4 in 4/6; CO = 4 on D7 in 4/6	?
131	tert-Butyl hydroperoxide (70%, aqueous)	75-91-2	Aliphatic hydroperoxide| tert-Butyl	L	Y	NICEATM	Sigma-Aldrich	70%, aqueous	1 of 1	Cat 1	CO mean ≥ 1	6/6	Unknown		**CO = 4**	1/6	Delayed effects; Study terminated on D7 with CO = 4 in 1/6, CO = 3 in 3/6 and CO = 2 in 2/6; CO = 4 on D7 in 1/6	X
132	Tetrahydrofuran	109-99-9	Oxolane| Saturated heterocyclic fragment	L	Y	NICEATM	Sigma-Aldrich	≥99.9%	1 of 1	Cat 1	CO mean ≥ 1; IR mean ≥ 1; Conj mean ≥ 2	6/6; 6/6; 5/6	Unknown		**CO = 4**	2/6	Delayed effects; Study terminated on D7 with CO = 4 in 2/6; CO = 4 between D4 and D7 in 2/6	X
133	Tributyltin oxide	56-35-9	Stannoxane	L	Y	NICEATM	Sigma-Aldrich	96%	1 of 1	Cat 1	NA		Unknown		**CO = 4**	4/6	Study terminated on D3 with CO = 4 in 4/6, CO = 1 in 1/6 and CO unknown in 1/6; CO = 4 on D3 in 4/6; No CO data reported for D1 to D3 in 1/6 and for D1 to D2 in 2/6 (obscured)	
134	Trichloroacetyl chloride	76-02-8	Acyl halide| Perhalogenated carbons derivatives	L	Y	NICEATM	Sigma-Aldrich	99%	1 of 1	Cat 1	NA		Unknown		**CO = 4**	4/4	Study terminated on D7 with CO = 4 in 4/4; Data missing between D2 and D4; CO = 4 between Hr1 and D7 in 4/4	
135	Acid Red 92 (Chemical name: 3,4,5,6-Tetrachloro-2-(1,4,5,8-tetrabromo-6-hydroxy-3-oxoxanthen-9-yl)-benzoic acid) (10%)	18472-87-2	Aromatic perhalogencarbons| Aryl halide| Fused carbocyclic aromatic| Fused saturated heterocycles| Heterocyclic spiro rings| Isobenzofuran| Lactone| Phenol| Xanthene	L (tested in solvent, available as S)	Y	NICEATM	Sigma-Aldrich	≥80%	1 of 1	Cat 1	CO mean ≥ 1; Conj mean ≥ 2	2/3; 2/3	Unknown		**CO = 4**	1/3	Study terminated on D14 with CO = 1 in 1/3; CO = 4 in this animal between D9 and D11, which reduces to score 1 on D12	X
136	Sodium undecylenate solution (33.2%, aqueous)	3398-33-2	Allyl| Carboxylic acid	L (tested in solvent, available as S)	Y	ECETOC	SCBT	90-100%	1 of 1	Cat 1	CO mean ≥ 3	1/1			**CO = 4**	1/1	CO = 4 between D1 and D3 in 1/1 fully reversed by D11; Study terminated on D11 with CO, IR, CR and CC = 0	X
137	(-)-trans-4-(4'-Fluorophenyl)-3-hydroxymethyl-1-methyl piperidine	105812-81-5	Alcohol| Alkane, branched with tertiary carbon| Aryl halide| Piperidine| Saturated heterocyclic amine| Saturated heterocyclic fragment	S	Y	EURL ECVAM	Chemos GmbH	98.5%	1 of 1	Cat 1	NA		Unknown		**CO = 4**	1/3	Study terminated on D2 with CO = 4, IR = 1, CR = 3 and CC = 4 in 1/3; CO = 2, IR = 1, CR and CC = 3 in 1/3; CO = 2, IR = 1, CR = 3 and CC = 4 in 1/3	X
138	1,2,4-Triazole Na salt	41253-21-8	Aryl| Triazole	S	Y	ECETOC	Sigma-Aldrich	90%	1 of 1	Cat 1	CO mean ≥ 3; IR mean > 1.5	1/1; 1/1	Unknown		**CO = 4**	1/1	CO = 4 between Hr1 and D3; Study terminated on D3 with CO = 4, IR = 2, CR = 3 and CC = 3	X
139	1,2-Benzisothiazol-3(2H)-one	2634-33-5	Benzothiazole/ Benzoisothiazole	S	Y	EURL ECVAM	Chemos GmbH	99.8%	1 of 1	Cat 1	NA		Unknown		**CO = 4**	4/6	Study terminated on D2; CO = 4 on D2 in 4/6	
140	1,3-Diiminobenz (f)-isoindoline	65558-69-2	Fused carbocyclic aromatic| Iminoisoindole| Iminolactams| Naphtalene	S	Y	NICEATM	Sigma-Aldrich	95%	1 of 1	Cat 1	NA		Unknown		**CO = 4**	4/4	Study terminated on D7 with CO = 4 in 4/4; Data only for D1 and D7; CO = 4 between D1 and D7 in 2/4 and on D7 in 2/4	
141	3-Amino-2,4-dichlorophenol HCl	61693-43-4	Aniline| Aryl halide| Phenol	S	Y	EURL ECVAM	Unknown	Unknown	1 of 1	Cat 1	NA		Unknown		**CO = 4**	1/1	Study terminated on D1 with CO = 4 in 1/1	X
142	4-Amino-5-methoxy-2-methylbenzene-sulphonic acid	6471-78-9	Aniline| Ether| Sulfonic acid	S	Y	ZEBET	SCBT	96%	1 of 1	Cat 1	CO mean ≥ 3	1/1	Unknown		**CO = 4**	1/1	Study terminated on D3 with CO = 4 in 1/1; CO = 4 between D1 and D3	X
143	4-Chloro-methanilic acid	98-36-2	Aniline| Aryl halide| Sulfonic acid	S	Y	ZEBET	TCI	>98%	1 of 1	Cat 1	CO mean ≥ 3	1/1	Unknown		**CO = 4**	1/1	Delayed effects; Study terminated on D7 with CO = 4 in 1/1; CO = 4 on D7	X
144	Acid blue 40 (Chemical name: 1-Amino-4-[4-(acetylamino)anilino]-9,10-dioxoanthracene-2-sulfonic acid monosodium salt)	6424-85-7	Aniline| Anthracenone/ Antracendione| Aryl| Carboxamide| Sulfonic acid	S	Y	NICEATM	SCBT	100%	1 of 1	Cat 1	Conj mean ≥ 2; CO mean ≥ 1	5/6; 4/6	Unknown		**CO = 4**	2/6	Study terminated on D14 with CO = 2 in 1/6 and CO = 0 in 5/6; CO = 4 on D1 in 1/6 (reduced to CO = 2 on D14) and between D2 and D3 in 1/6 (fully reversed by D14); CO = 0 between Hr1 and D14 in 2/6	X
145	Benzalkonium chloride (neat)	63449-41-2 (another possible CAS # is 8001-54-5, which is reported in NICEATM ALTTOX)	Ammonium salt| Benzyl	S	Y	NICEATM	Sigma-Aldrich	≥95%	1 of 1	Cat 1	CO mean ≥ 1; Conj mean ≥ 2	3/3; 3/3	Possibly CO pers D21 (at least)	at least 1/3	**CO = 4**	1/3	Study terminated on D14 with CO = 3 in 1/3, CO = 2 in 2/3, IR = 1 in 3/3, CR = 1 in 2/3, CR = 0 in 1/3 and CC = 2 in 3/3; CO = 4 between D1 and D2 in 1/3	X
146	beta-Resorcyclic acid	89-86-1	Carboxylic acid| Phenol	S	Y	ZEBET	Sigma-Aldrich	≥97%	1 of 1	Cat 1	CO mean ≥ 3	1/1	Unknown		**CO = 4**	1/1	Study terminated on D3 with CO = 4 in 1/1; CO = 4 between D1 and D3	X

Study Number	Test Chemical Name	CAS RN	Organic Functional Groups	Physical Form as tested	Physical Form Confirmation e.g., by MSDS (Y / N)	Data Source	Commercial Source	Available Purity	Number of Studies	UN GHS Classification	Drivers of Classification<br>Severity<br>Cut-off values	Drivers of Classification<br>Severity<br>Number of animals	Drivers of Classification<br>Persistence<br>Cut-off time	Drivers of Classification<br>Persistence<br>Number of animals	Drivers of Classification<br>Specific Observations<br>CO = 4 or other observations	Drivers of Classification<br>Specific Observations<br>Number of animals	Comments	Should Not Be Used
147	Chlorhexidine	55-56-1	Aryl halide| Guanidine	S	Y	ECETOC / ZEBET	Sigma-Aldrich	≥99.5%	1 of 1	Cat 1	CO mean ≥ 1; Conj mean ≥ 2; IR mean ≥ 1	3/3; 3/3; 3/3	Unknown		**CO = 4**	1/3	Study terminated on D3 with CO = 4 in 1/3, CO = 3 in 1/3 and CO = 2 in 1/3; CO = 4 between Hr1 and D3 in 1/3; Two studies are reported in ECETOC and ZEBET with the exact same animal scores (apart from 1 single CO score for animal 2 on D3, probably a typo) and are considered duplicate entries	X
148	Chlorophenacyl	6305-04-0	Alkyl halide| Ketone| Phenol	S	Y	NICEATM	Sigma-Aldrich	Unknown	1 of 1	Cat 1	NA		Unknown		**CO = 4**	4/6	10 mg dose; Study terminated on D3 with CO = 4 in 3/6, CO = 3 in 2/6 and CO = 2 in 1/6; CO = 4 between D1 and D2 in 1/6, between D2 and D3 in 2/6 and on D3 in 1/6	
149	Granuform (Chemical name: Paraformaldehyde)	30525-89-4	Alcohol	S	Y	ZEBET	Sigma-Aldrich	95%	1 of 1	Cat 1	CO mean ≥ 1; Conj mean ≥ 2; IR mean ≥ 1	3/3; 3/3; at least 2/3	Unknown		**CO = 4**	1/3	Study terminated on D3 with CO = 4 in 1/1; CO = 4 on D3	X
150	N-(2-Methylphenyl)-iminodicarbonimidic diamide (1-(o-Tolyl)biguanide)	93-69-6	Aryl| Guanidine	S	Y	ZEBET	Sigma-Aldrich	98%	1 of 1	Cat 1	NA		Unknown		**CO = 4**	3/3	Study terminated after Hr1; Considered Cat 1 based on consistency of CO = 4 in 3/3	
151	N-Acetyl-DL-methionine	1115-47-5	Carboxamide| Sarcosine| Sulfide	S	Y	ZEBET	Sigma-Aldrich	99%	1 of 1	Cat 1	CO mean ≥ 1	3/3	Unknown		**CO = 4**	1/3	Study terminated on D3 with CO = 4 in 1/3; CO = 4 between Hr1 and D3	X
152	Sodium disilicate	13870-28-5	N/A	S	Y	ZEBET	Unknown	Unknown	1 of 1	Cat 1	NA		Unknown		**CO = 4**	3/3	Study terminated on D1 with CO = 4 in 3/3; CO = 4 on D1 in 3/3	?
153	Sodium hydrogen sulphate	7681-38-1	N/A	S	Y	ZEBET	Sigma-Aldrich	95%	1 of 1	Cat 1	NA		Unknown		**CO = 4**	1/1	Study terminated on D2 with CO = 4 in 1/1; CO = 4 between D1 and D2	X
154	Sodium silicate (2.0 ratio hydrous)	1344-09-8	N/A	S	Y	NICEATM	PQ Corporation; Samarth Chemical Products	Unknown	1 of 1	Cat 1	NA		Unknown		**CO = 4**	1/6	Study terminated on D3; No data reported for D1 to D3 in 3/6, for D1 to D2 in 1/6 and for D2 to D3 in 1/6 (obscured); CO = 4 on D1 in 1/6; CO = 0 on D3 in 2/6; 10 mg dose	X
155	Thiourea	62-56-6	Thiourea derivatives	S	Y	LNS / ECETOC	Sigma-Aldrich	≥99%	1 of 1	Cat 1	Conj mean ≥ 2; CO mean ≥ 1; IR mean ≥ 1	3/3; 2/3; 2/3			**CO = 4**	2/3	In LNS: CO = 4 on Hr1 in 2/3 fully reversed by D7 and D21 respectively; Animal 1 with full reversibility of all endpoints on D14 (CO on D7, max CO = 4), animal 2 on D14 (CO on D2, max CO = 2) and animal 3 on D21 (CO on D21, max CO = 4); In ECETOC 3/3 died (no rinse)	X
156	Triethanolamine orthovanadate (neat)	13476-99-8	Diketone	S	Y	NICEATM	Sigma-Aldrich	97%	1 of 1	Cat 1	NA		Unknown		**CO = 4**	4/6	Delayed effects; Study terminated on D7 with CO = 4 in 4/6, CO = 2 in 1/6 and CO = 0 in 1/6; CO & IR data missing for D1; CO = 4 on D7 in 3/6 and between D2 and D7 in 1/6	
157	2-(N-Methyl-2-pyridylamino)ethanol	122321-04-4	Alcohol| Aryl| Pyridine	L	Y	EURL ECVAM	Chemos GmbH	99%	1 of 1	Cat 1	CO mean ≥ 1; Conj mean ≥ 2; IR mean ≥ 1	1/1; 1/1; 1/1	Unknown		**Other observations**	1/1	Study terminated on D20; Pannus formation	X
158	1-(3,4-Dichlorophenyl)-5-isopropylbiguanide HCL	537-21-3	Aryl halide| Guanidine| Isopropyl	S	N	NICEATM	Toronto Research Chemicals	Unknown	1 of 1	Cat 1	NA		Unknown		**Other observations**	1/1	Study terminated on D1; Tranlucent corneal opacity, iridal inflammation, severe conjunctivitis with dulling of normal lustre of cornea, hemorrhage of iris, pale appearance of nictitating and lower conjunctival membranes; Animal sacrificed due to severity of reaction	X
159	2-Nitro-4-thiocyanoaniline	54029-45-7	Aniline| Nitrobenzene| Thiocyanate	S	Y	NICEATM	Sigma-Aldrich	95%	1 of 1	Cat 1	NA		Unknown		**Other observations**	1/1	Orange staining of cornea; Study terminated on D1	(X)
160	4-((4-Amino-3-methylphenyl)(4-imino-3-methyl-2,5-cyclohexadien-1-ylidene)methyl)-2-methyl benzenamine hydrochloride	3248-91-7	Allyl| Anilines| Benzyl| Conjugated hydrocarbon| Dianilines| Ketimine	S	Y	EURL ECVAM	Sigma-Aldrich	analytical standard	1 of 1	Cat 1	NA		Unknown		**Other observations**	1/1	Colour staining of cornea; Study terminated on D1	(X)
161	Iodine chloride with pyridine (1:1)	6443-90-9	Aryl| Pyridine	S	Y	NICEATM	Unknown	Unknown	1 of 1	Cat 1	NA		Unknown		**Other observations**	1/1	Study terminated after Hr1; Dulling of cornea; Animal sacrificed due to severity of reaction	X
162	Phosphoric acid compound with 4-[(2,6-dichlorophenyl)(4-imino-3,5-dimethyl-2,5-cyclohexadien-1-ylidene)methyl]-2,6-dimethylaniline (1:1) (neat)	74578-10-2	Alkene| Allyl| Aryl| Aryl halide| Ketimine| Precursors quinoid compounds	S	Y	Cosmetics Europe	Proprietary	≥95%	1 of 1	Cat 1	NA		Unknown		**Other observations**	1/1	Blue staining of cornea; Study terminated on D10	(X)/?
163	Tetra-N-octylammonium bromide	14866-33-2	Ammonium salt	S	Y	NICEATM	Sigma-Aldrich	98%	1 of 1	Cat 1	NA		Unknown		**Other observations**	1/1	Study terminated after Hr6; Translucent corneal opacity, iridal inflammation and severe conjunctivitis with petechial hemorrhage scattered over nictitating membrane and blood stained discharge; Animal sacrificed due to severity of reaction	X
164	iso-Propyl dicyanamide	35695-36-4	Cyanamide| Isopropyl	Unknown	N	NICEATM	Unknown	Unknown	1 of 1	Cat 1	CO mean ≥ 1; Conj mean ≥ 2; IR mean ≥ 1	1/1; 1/1; 1/1	Unknown		**Other observations**	1/1	Study terminated on D3; Translucent corneal opacity, iridial inflammation, severe conjunctivitis with dulling of normal lustre of cornea, hemorrhage and pale appearance of nictitating membrane; Animal sacrificed due to severity of reaction	X
165	Mebrophen hydramide HCL	13977-28-1	Aliphatic Amine, tertiary| Aryl| Aryl halide| Ether	Unknown	N	NICEATM	Unknown	Unknown	1 of 1	Cat 1	NA		Unknown		**Other observations**	1/1	Study terminated on D1; Grade 3 opacity over 1/2 with grade 2 over remaining half; Pale appearance of lower conjunctival membrane and white area on nictitating membrane; Areas of hemorrhage scattered over nictitating membrane; Opalescent corneal opacity, iridal inflammation, and severe conjunctivitis, pale appearance of lower conjunctival membrane and white area and hemorrhage over nictitating membrane; Abnormally small pupil; Animal sacrificed due to severe ocular reactions and pain and discomfort	X
166	2,6-Dichlorobenzoyl chloride	4659-45-4	Acyl halide| Aryl halide	L	Y	ECETOC	Sigma-Aldrich	99%	1 of 1	Cat 2A	**CO mean ≥ 1**; Conj mean ≥ 2	6/6; 6/6	**Conj pers D7**; CO pers D7	6/6; 2/6				
167	2-Ethyl-1-hexanol	104-76-7	Alcohol| Alkane, branched with tertiary carbon	L	Y	ECETOC	Sigma-Aldrich	≥99.6%	1 of 1	Cat 2A	**CO mean ≥ 1**; Conj mean ≥ 2	4/4; 3/4	**Conj pers D7**; CO pers D7	3/4; 1/4				
168	Acetone	67-64-1	Ketone	L	Y	ECETOC	Sigma-Aldrich	≥99.9%	1 of 1	Cat 2A	**CO mean ≥ 1**; Conj mean ≥ 2; IR mean ≥ 1	4/4; 4/4; 4/4	**Conj pers D7**; CO pers D7; IR pers D7	4/4; 1/4; 1/4				
169	Chlorhexidine gluconate (20%, aqueous)	18472-51-0	Aryl halide| Carboxylic acid| Dihydroxyl group| Guanidine	L	Y	NICEATM	Sigma-Aldrich	20%, aqueous	1 of 1	Cat 2A	**CO mean ≥ 1**	3/3	**CO pers D7**	1/3				
170	Cyclopentanol	96-41-3	Alcohol| Cycloalkane	L	Y	ECETOC	Sigma-Aldrich	99%	1 of 1	Cat 2A	**CO mean ≥ 1**; Conj mean ≥ 2	3/3; 3/3	**CO pers D7**; Conj pers D7	1/3; 1/3				
171	Ethanol (neat) (2 of 4)	64-17-5	Alcohol	L	Y	ECETOC	Sigma-Aldrich	>99.8%	2 of 4	Cat 2A	**CO mean ≥ 1**; Conj mean ≥ 2	2/3; 2/3	**Conj pers D7**	2/3				X
172	Ethyl trans-3-ethoxyacrylate	5941-55-9	Acrylate| Alkoxy| Ether	L	Y	EURL ECVAM	Alfa Aesar	98%	1 of 1	Cat 2A	**CO mean ≥ 1**; Conj mean ≥ 2; IR mean ≥ 1	3/3; 3/3; 2/3	**Conj pers D7**; IR pers D7; CO pers D7	3/3; 2/3; 1/3				
173	gamma-Butyrolactone (1 of 2)	96-48-0	Lactone| Oxolane| Saturated heterocyclic fragment	L	Y	ECETOC	TCI	>99%	1 of 2	Cat 2A	**CO mean ≥ 1**; Conj mean ≥ 2	3/3; 3/3	**Conj pers D7**; CO pers D7	2/3; 1/3				
174	gamma-Butyrolactone (2 of 2)	96-48-0	Lactone| Oxolane| Saturated heterocyclic fragment	L	Y	LNS	TCI	>99%	2 of 2	Cat 2A	**CO mean ≥ 1**; Conj mean ≥ 2; IR mean ≥ 1	3/3; 2/3; 2/3	**Conj pers D7**; CO pers D7	2/3; 1/3				
175	iso-Butanol (1 of 2)	78-83-1	Alcohol| Isopropyl	L	Y	ECETOC	Sigma-Aldrich	≥99%	1 of 2	Cat 2A	**CO mean ≥ 1**; Conj mean ≥ 2	4/4; 4/4	**Conj pers D7**; CO pers D7	4/4; 1/4				X
176	Methyl acetate (1 of 2)	79-20-9	Acetoxy| Carboxylic acid ester	L	Y	ECETOC	Sigma-Aldrich	≥99.8%	1 of 2	Cat 2A	**CO mean ≥ 1**; Conj mean ≥ 2	3/4; 3/4	**Conj pers D7**; CO pers D7	4/4; 1/4				(X)
177	Methyl ethyl ketone	78-93-3	Ketone	L	Y	ECETOC	Sigma-Aldrich	≥99.7%	1 of 1	Cat 2A	**CO mean ≥ 1**; Conj mean ≥ 2	3/4; 3/4	**Conj pers D7**	3/4				
178	Methyl N,N,N-trimethyl-4-[(4,7,7-trimethyl-3-oxobicyclo[2.2.1]hept-2-ylidene)methyl]anilinium sulphate (30%, aqueous) (1 of 2)	52793-97-2	Alkene| Aromatic amine| Bicycloheptane| Bridged-ring carbocycles| Cycloalkane| Cycloketone| Sulfate	L	Y	EURL ECVAM	Proprietary	30%, aqueous	1 of 2	Cat 2A	**CO mean ≥ 1**; Conj mean ≥ 2; IR mean ≥ 1	3/3; 3/3; 3/3	**Conj pers D7**	3/3				?
179	n-Butanol (neat) (2 of 2)	71-36-3	Alcohol	L	Y	ECETOC	Sigma-Aldrich	99.8%	2 of 2	Cat 2A	**CO mean ≥ 1**; Conj mean ≥ 2	4/4; 4/4	**Conj pers D7**; CO pers D7	3/4; 1/4				X
180	n-Hexanol	111-27-3	Alcohol	L	Y	ECETOC	Sigma-Aldrich	≥99.5%	1 of 1	Cat 2A	**CO mean ≥ 1**; Conj mean ≥ 2	4/4; 4/4	**Conj pers D7**	4/4				
181	n-Octanol (1 of 2)	111-87-5	Alcohol	L	Y	ECETOC	Sigma-Aldrich	≥99%	1 of 2	Cat 2A	**CO mean ≥ 1**; Conj mean ≥ 2; IR mean ≥ 1	3/3; 2/3; 2/3	**Conj pers D7**; CO pers D7	2/3; 1/3				(X)
182	Polyether E810 (Chemical name: Polyether polyol)	25214-63-5	N/A	L	Y	NICEATM	Bayer	95-100%	1 of 1	Cat 2A	**CO mean ≥ 1**; Conj mean ≥ 2	3/3; 3/3	**Conj pers D7**	3/3				
183	Propasol Solvent P	1569-01-3	Alcohol| Alkoxy| Ether	L	Y	NICEATM	Sigma-Aldrich	99%	1 of 1	Cat 2A	**CO mean ≥ 1**	5/6	**CO pers D7**; Conj pers D7	1/6; 1/6				
184	Pyridine (2 of 2)	110-86-1	Aryl| Pyridine	L	Y	LNS	Sigma-Aldrich	>99.9%	2 of 2	Cat 2A	**CO mean ≥ 1**; Conj mean ≥ 2; IR mean ≥ 1	3/3; 3/3; 2/3	**Conj pers D7**; CO pers D7	3/3; 2/3				X
185	Cetyl pyridinium bromide (1%)	140-72-7	Ammonium salt| Aryl| Pyridine	L (tested in solvent, available as S)	Y	ECETOC	Sigma-Aldrich	98%	1 of 1	Cat 2A	**CO mean ≥ 1**; Conj mean ≥ 2; IR mean ≥ 1	6/6; 6/6; 4/6	**Conj pers D7**; CO pers D7; IR pers D7	6/6; 3/6; 2/6				
186	Deoxycholic acid Na salt (10%)	302-95-4	Alkane, branched with tertiary carbon| Carboxylic acid| Cycloalkane| Dihydroxyl group	L (tested in solvent, available as S)	Y	LNS	Sigma-Aldrich	≥98%	1 of 1	Cat 2A	**CO mean ≥ 1**; Conj mean ≥ 2; IR mean ≥ 1	3/3; 3/3; 3/3	**Conj pers D7**; CO pers D7	2/3; 1/3				
187	Lauryl sulphobetaine (10%)	14933-08-5	Ammonium salt| Sulfonic acid	L (tested in solvent, available as S)	Y	LNS	Sigma-Aldrich	≥97%	1 of 1	Cat 2A	**CO mean ≥ 1**; Conj mean ≥ 2	3/3; 3/3	**Conj pers D7**; CO pers D7	3/3; 2/3				
188	Sodium lauryl sulphate (30%)	151-21-3	Alkoxy| Sulfate	L (tested in solvent, available as S)	Y	ECETOC	Sigma-Aldrich	≥99%	1 of 1	Cat 2A	**CO mean ≥ 1**; Conj mean ≥ 2; IR mean ≥ 1	6/6; 6/6; 4/6	**Conj pers D7**; CO pers D7	3/6; 1/6				X
189	Sodium lauryl sulphate (10%) (3 of 3)	151-21-3	Alkoxy| Sulfate	L (tested in solvent, available as S)	Y	NICEATM	Sigma-Aldrich	≥99%	3 of 3	Cat 2A	**CO mean ≥ 1**	2/3	**CO pers D7**; Conj pers D7	1/3; 1/3				X
190	(2R,3R)-3-((R)-1-(Tert-butyldimethylsiloxy)ethyl)-4-oxoazetidin-2-yl acetate	76855-69-1	Acetoxy| AlkoxySilane| Lactam| tert-Butyl	S	Y	EURL ECVAM	Chemos GmbH	99.8%	1 of 1	Cat 2A	**CO mean ≥ 1**; Conj mean ≥ 2; IR mean ≥ 1	3/3; 3/3; 3/3	**CO pers D7**; Conj pers D7	3/3; 3/3				
191	3,3'-Dithiopropionic acid	1119-62-6	Carboxylic acid| Disulfide	S	Y	ECETOC	Sigma-Aldrich	99%	1 of 1	Cat 2A	**CO mean ≥ 1**	3/3	**CO pers D7**	1/3				
192	4-Carboxybenzaldehyde	619-66-9	Aldehyde| Aryl| Carboxylic acid	S	Y	ECETOC	Sigma-Aldrich	97%	1 of 1	Cat 2A	**CO mean ≥ 1**; Conj mean ≥ 2; IR mean ≥ 1	3/3; 3/3; 3/3	**CO pers D7**; Conj pers D7	2/3; 2/3				

Cosmetics Europe
Draize eye test Reference Database (DRD)
Barroso *et al.* , 2016

Study Number	Test Chemical Name	CAS RN	Organic Functional Groups	Physical Form as tested	Physical Form Confirmation e.g., by MSDS (Y / N)	Data Source	Commercial Source	Available Purity	Number of Studies	UN GHS Classification	Drivers of Classification						Comments	Should Not Be Used
											Severity		Persistence		Specific Observations			
											Cut-off values	Number of animals	Cut-off time	Number of animals	CO = 4 or other observations	Number of animals		
193	6,7-Dihydro-2,3-dimethyl-imidazo[1,2-a]pyridin-8(5H)-one	362525-73-3	Aryl| Cycloketone| Fused saturated heterocycles| Fused unsaturated heterocycles| Imidazole| Piperidine| Saturated heterocyclic amine| Saturated heterocyclic fragment	S	Y	EURL ECVAM	Proprietary	>99.8%	1 of 1	Cat 2A	**CO mean ≥ 1**; Conj mean ≥ 2; IR mean ≥ 1	3/3; 3/3; 2/3	**Conj pers D7**; IR pers D7	3/3; 1/3				?
194	Dibenzyl phosphate	1623-08-1	Benzyl| Phosphate ester	S	Y	ECETOC	Sigma-Aldrich	99%	1 of 1	Cat 2A	**CO mean ≥ 1**; Conj mean ≥ 2	3/3; 3/3	**Conj pers D7**; CO pers D7	3/3; 1/3				
195	Disodium (E)-1,2-bis-(4-(4-methylamino-6-(4-methylcarbamoylphenylamino)-1,3,5-triazin-2-ylamino)phenyl-2-sulphonato)ethene	180850-95-7	Alkene| Arene| Carboxamide| Melamines| Sulfonic acid	S	Y	EURL ECVAM	Proprietary	Unknown	1 of 1	Cat 2A	**CO mean ≥ 1**; Conj mean ≥ 2	5/6; 4/6	**CO pers D7**; Conj pers D7; IR pers D7	3/6; 3/6; 2/6				?
196	gamma-(Aminocarbonyl)-N-methyl-N,N-bis(1-methyletheyl)-gamma-phenyl-, iodide	71-81-8	Ammonium salt| Aryl| Carboxamide| Isopropyl	S	Y	NICEATM	SCBT	Unknown	1 of 1	Cat 2A	**CO mean ≥ 1**; Conj mean ≥ 2; IR mean ≥ 1	3/3; 3/3; 3/3	**CO pers D7**; Conj pers D7	1/3; 1/3				
197	Methyl (2E)-[2-(chloromethyl)phenyl](methoxyimino) acetate	189813-45-4	Alkyl halide| Benzyl| Carboxylic acid ester| Ketoxime derivatives	S	Y	EURL ECVAM	Proprietary	100%	1 of 1	Cat 2A	**CO mean ≥ 1**; Conj mean ≥ 2	3/3; 3/3	**Conj pers D7**	3/3				?
198	Tetra aminopyrimidine sulphate (1 of 2)	5392-28-9	Anilines| Anilines(meta)| Anilines(ortho)| Anilines(para)	S	Y	LNS	Sigma-Aldrich	97%	1 of 2	Cat 2A	**CO mean ≥ 1**	2/3	**CO pers D7**; Conj pers D7	1/3; 1/3				X
199	Alkyl (C10-16) glucoside sodium carboxylate (~30%, aqueous)	383178-66-3	Dihydroxyl group	L	Y	EURL ECVAM	Proprietary	~30%, aqueous	1 of 1	Cat 2A	**Conj mean ≥ 2**; IR mean ≥ 1	3/3; 2/3	**Conj pers D7**	1/3				?
200	Allyl alcohol	107-18-6	Alcohol| Allyl	L	Y	LNS	Sigma-Aldrich	≥99%	1 of 1	Cat 2A	**Conj mean ≥ 2**; CO mean ≥ 1; IR mean ≥ 1	3/3; 2/3; 2/3	**Conj pers D7**	3/3				
201	Ethanol (neat) (3 of 4)	64-17-5	Alcohol	L	Y	LNS	Sigma-Aldrich	>99.8%	3 of 4	Cat 2A	**Conj mean ≥ 2**	2/3	**CO pers D7**; Conj pers D7	1/3; 1/3				X
202	Furfural	98-01-1	Alpha,beta unsaturated aldehyde| Aryl| Furane	L	Y	EURL ECVAM	Sigma-Aldrich	99%	1 of 1	Cat 2A	**Conj mean ≥ 2**	2/3	**Conj pers D7**; CO pers D7	3/3; 1/3				
203	iso-Propanol	67-63-0	Alcohol| Isopropyl	L	Y	ECETOC	Sigma-Aldrich	≥99.7%	1 of 1	Cat 2A	**Conj mean ≥ 2**	3/4	**Conj pers D7**	4/4				
204	Methyl cyanoacetate	105-34-0	Carboxylic acid ester| Nitrile	L	Y	ECETOC	Sigma-Aldrich	99%	1 of 1	Cat 2A	**Conj mean ≥ 2**; IR mean ≥ 1	3/3; 3/3	**Conj pers D7**	2/3				
205	Methyl N,N,N-trimethyl-4-[(4,7,7-trimethyl-3-oxobicyclo[2.2.1]hept-2-ylidene)methyl]anilinium sulphate (30%, aqueous) (2 of 2)	52793-97-2	Alkene| Aromatic amine| Bicycloheptane| Bridged-ring carbocycles| Cycloalkane| Cycloketone| Sulfate	L	Y	EURL ECVAM	Proprietary	30%, aqueous	2 of 2	Cat 2A	**Conj mean ≥ 2**	2/3	**Conj pers D7**	3/3				?
206	o-Toluene diamine, propoxylated, ethoxylated	67800-94-6	Epoxide| Precursors quinoid compounds| Saturated heterocyclic fragment	L	Y	NICEATM	Dow	100%	1 of 1	Cat 2A	**Conj mean ≥ 2**	3/3	**Conj pers D7**	2/3				
207	Triton X-100 (5%) (1 of 2)	9002-93-1	Alcohol| Alkane branched with quaternary carbon| Aryl| Ether| tert-Butyl	L	Y	ECETOC	Sigma-Aldrich	laboratory grade	1 of 2	Cat 2A	**Conj mean ≥ 2**; CO mean ≥ 1	6/6; 4/6	**Conj pers D7**	2/6				
208	Igepon AC-78 (5%) (Chemical name: Sodium cocoyl isethionate)	58969-27-0 (original CAS # deleted and replaced by 61789-32-0)	Carboxylic acid ester| Sulfonic acid	L (tested in solvent, neat chemical unknown)	Y	ECETOC	Unknown	Unknown	1 of 1	Cat 2A	**Conj mean ≥ 2**; CO mean ≥ 1	6/6; 5/6	**Conj pers D7**	6/6				?
209	N-Lauroyl sarcosine Na salt (10%)	137-16-6 (7631-98-3 reported by Gautheron et al. 1994, which corresponds to N-Dodecyl sarcosine Na salt or Sodium lauryl sarcosine)	Carboxamide| Carboxylic acid	L (tested in solvent, available as S)	Y	LNS	Sigma-Aldrich	≥99%	1 of 1	Cat 2A	**Conj mean ≥ 2**; CO mean ≥ 1	3/3; 2/3	**Conj pers D7**	1/3			Gautheron et al. 1994 reports CAS # 7631-98-3; However, it also mentions that the chemical tested was supplied by Sigma with catalogue number L5125, which in fact corresponds to CAS # 137-16-6	X
210	Sodium hydroxide (1%)	1310-73-2	Hydrazine	L (tested in solvent, available as S)	Y	ECETOC	Sigma-Aldrich	≥98%	1 of 1	Cat 2A	**Conj mean ≥ 2**	3/4	**Conj pers D7**	1/4				
211	1,3-bis-(2,4-Diaminophenoxy) propane tetrachloride	74918-21-1	Aminoaniline, meta| Ether	S	Y	EURL ECVAM	Chemos GmbH	99.3%	1 of 1	Cat 2A	**Conj mean ≥ 2**	3/3	**Conj pers D7**	2/3				
212	1,5-Naphthalenediol	83-56-7	Fused carbocyclic aromatic| Naphtalene| Phenol	S	Y	EURL ECVAM	Sigma-Aldrich	97%	1 of 1	Cat 2A	**Conj mean ≥ 2**	3/3	**Conj pers D7**	3/3				
213	2-Amino-3-hydroxy pyridine	16867-03-1	Heterocyclic Phenol	S	Y	EURL ECVAM	Sigma-Aldrich	98%	1 of 1	Cat 2A	**Conj mean ≥ 2**	3/3	**Conj pers D7**	3/3				
214	4-Amino-3-nitrophenol	610-81-1	Anilines| Nitro| Phenol	S	Y	EURL ECVAM	Sigma-Aldrich	98%	1 of 1	Cat 2A	**Conj mean ≥ 2**; CO mean ≥ 1	3/3; 2/3	**Conj pers D7**	1/3				
215	Ammonium nitrate	6484-52-2	Inorganic Salt	S	Y	ECETOC	Sigma-Aldrich	≥99.5%	1 of 1	Cat 2A	**Conj mean ≥ 2**	3/3	**Conj pers D7**	1/3				
216	Sodium benzoate	532-32-1	Aryl| Carboxylic acid	S	Y	EURL ECVAM	Sigma-Aldrich	≥99%	1 of 1	Cat 2A	**Conj mean ≥ 2**	3/3	**Conj pers D7**	3/3				
217	Butoxyethanol (3 of 3)	111-76-2	Alcohol| Alkoxy| Ether	L	Y	EURL ECVAM	Sigma-Aldrich	≥99%	3 of 3	Cat 2	**Conj mean ≥ 2**; CO mean ≥ 1	3/3; 2/3	Unknown				No scoring recorded on D7: unable to distinguish between Cat 2A and Cat 2B	X
218	2-Methyl-1-pentanol	105-30-6	Alcohol| Alkane, branched with tertiary carbon	L	Y	ECETOC	Sigma-Aldrich	99%	1 of 1	Cat 2B	**CO mean ≥ 1**	2/3						
219	3-Chloropropionitrile	542-76-7	Alkyl halide| Nitrile	L	Y	ECETOC	Sigma-Aldrich	98%	1 of 1	Cat 2B	**CO mean ≥ 1**	2/3						
220	Alcohol SDA 39C (79%)	64-17-5 (Ethanol)<br>84-66-6 (Diethylphthalate)	Ethanol	L	Y	NICEATM	Fisher Scientific; PHARMCO-AAPER	Unknown	1 of 1	Cat 2B	**CO mean ≥ 1**; Conj mean ≥ 2	3/3; 3/3					SDA = special denatured alcohol (99.01% (v/v) Ethanol + 0.99% (v/v) Diethylphthalate)	X
221	Butyl Dipropasol Solvent	29911-27-1	Alcohol| Alkoxy| Ether	L	Y	NICEATM	Sigma-Aldrich	≥98.5%	1 of 1	Cat 2B	**CO mean ≥ 1**	4/6						
222	Diethyl toluamide	134-62-3	Benzamide	L	Y	EURL ECVAM	Chemos GmbH	98%	1 of 1	Cat 2B	**CO mean ≥ 1**	3/3						
223	Ethyl-2-methyl acetoacetate	609-14-3	Carboxylic acid ester| Ketone	L	Y	ECETOC	Sigma-Aldrich	90%	1 of 1	Cat 2B	**CO mean ≥ 1**	2/3						
224	iso-Butanal	78-84-2	Aldehyde| Isopropyl	L	Y	ZEBET	Sigma-Aldrich	≥99.5%	1 of 1	Cat 2B	**CO mean ≥ 1**; Conj mean ≥ 2	2/3; 2/3						
225	Methyl acetate (2 of 2)	79-20-9	Acetoxy| Carboxylic acid ester	L	Y	ZEBET	Sigma-Aldrich	≥99.8%	2 of 2	Cat 2B	**CO mean ≥ 1**; Conj mean ≥ 2; IR mean ≥ 1	3/3; 3/3; 3/3						(X)
226	Monoethanolamine (10%)	141-43-5	Alcohol| Aliphatic Amine, primary	L	Y	NICEATM	Sigma-Aldrich	≥99.5%	1 of 1	Cat 2B	**CO mean ≥ 1**	2/3						
227	n-Butanal	123-72-8	Aldehyde	L	Y	ZEBET	Sigma-Aldrich	≥99.5%	1 of 1	Cat 2B	**CO mean ≥ 1**; Conj mean ≥ 2	2/3; 2/3						
228	n-Octanol (2 of 2)	111-87-5	Alcohol	L	Y	LNS	Sigma-Aldrich	≥99%	2 of 2	Cat 2B	**CO mean ≥ 1**; Conj mean ≥ 2; IR mean ≥ 1	2/3; 2/3; 2/3						(X)
229	2,6-Dichloro-5-fluoro-beta-oxo-3-pyridinepropanoate	96568-04-6	Aromatic heterocyclic halide| Aryl halide| Carboxylic acid ester| Ketone	S	Y	NICEATM	Sigma-Aldrich	98%	1 of 1	Cat 2B	**CO mean ≥ 1**	2/3						
230	2-Methyl-1,1'-biphenyl-3-ylmethyl (Z)-3-(2-chloro-3,3,3-trifluoro-1-propenyl)-2,2-dimethyl cyclopropane carboxylate	82657-04-3	Alkenyl halide| Biphenyl| Carboxylic acid ester| Cycloalkane| Perhalogenated carbons derivatives	S	Y	EURL ECVAM	Chemos GmbH	97.1%	1 of 1	Cat 2B	**CO mean ≥ 1**	4/6						
231	2-Methyl-2-butanone-(4-sulfonamidophenyl) hydrazone	Unknown	N/A	S	Y	NICEATM	Unknown	Unknown	1 of 1	Cat 2B	**CO mean ≥ 1**; Conj mean ≥ 2; IR mean ≥ 1	3/3; 3/3; 3/3					Scores not recorded for D7; D8 scores show full reversibility for all endpoints; Assumed to be Cat 2B	?
232	Acetoacetic acid glycol ester	5459-04-1	Carboxylic acid ester| Diketone	S	N	ZEBET	Unknown	Unknown	1 of 1	Cat 2B	**CO mean ≥ 1**; Conj mean ≥ 2	2/3; 2/3						?
233	2-Pseudoionone	141-10-6	Alkene| Allyl| Conjugated system| Ketone	L	Y	ZEBET	Sigma-Aldrich	≥90%	1 of 1	Cat 2B	**Conj mean ≥ 2**	2/3						
234	iso-Propyl acetoacetate	542-08-5	Carboxylic acid ester| Isopropyl| Ketone	L	Y	ZEBET	Sigma-Aldrich	≥99%	1 of 1	Cat 2B	**Conj mean ≥ 2**	2/3						
235	Lupranol 3402 (Chemical name: Ethylenediamine, propoxylated)	25214-63-5	N/A	L	Y	NICEATM	BASF	100%	1 of 1	Cat 2B	**Conj mean ≥ 2**; CO mean ≥ 1	3/3; 2/3						
236	S-Bioallethrin	28434-00-6	Allyl| Carboxylic acid ester| Cycloalkane| Cycloalkene| Cycloketone	L	Y	EURL ECVAM	Sigma-Aldrich	analytical standard	1 of 1	Cat 2B	**Conj mean ≥ 2**	5/6						

Study Number	Test Chemical Name	CAS RN	Organic Functional Groups	Physical Form as tested	Physical Form Confirmation e.g., by MSDS (Y / N)	Data Source	Commercial Source	Available Purity	Number of Studies	UN GHS Classification	Drivers of Classification: Severity		Persistence		Specific Observations		Comments	Should Not Be Used
											Cut-off values	Number of animals	Cut-off time	Number of animals	CO = 4 or other observations	Number of animals		
237	Glycolic acid (10%)	79-14-1	Alcohol| Carboxylic acid	L (tested in solvent, available as S)	Y	NICEATM	Sigma-Aldrich	99%	1 of 1	Cat 2B	**Conj mean ≥ 2**	2/3						
238	1,4-Dibutoxybenzene	104-36-9	Alkoxy| Aryl| Ether	S	Y	ZEBET	TCI	>98%	1 of 1	Cat 2B	**Conj mean ≥ 2**	3/3						
239	2-Hydroxy-1,4-naphthoquinone	83-72-7	Enol| Naphthoquinone	S	Y	EURL ECVAM	Sigma-Aldrich	97%	1 of 1	Cat 2B	**Conj mean ≥ 2**	2/3						
240	Camphene	79-92-5	Alkane, branched with tertiary carbon| Alkene| Bicycloheptane| Bridged-ring carbocycles| Cycloalkane	S	Y	ZEBET	Sigma-Aldrich	≥95%	1 of 1	Cat 2B	**Conj mean ≥ 2**	3/3						
241	m-Dinitrobenzene	99-65-0	Nitrobenzene	S	Y	ZEBET	Sigma-Aldrich	97%	1 of 1	Cat 2B	**Conj mean ≥ 2**; IR mean ≥ 1	2/3; 2/3						
242	p-Nitrobenzoic acid	62-23-7	Carboxylic acid| Nitrobenzene	S	Y	ZEBET	Sigma-Aldrich	≥98%	1 of 1	Cat 2B	**Conj mean ≥ 2**	3/3						
243	Sodium monochloroacetate	3926-62-3	Alkyl halide| Carboxylic acid	S	Y	ZEBET	Sigma-Aldrich	98%	1 of 1	Cat 2B	**Conj mean ≥ 2**	3/3						
244	Di-iso-propyl aminoethyldiphenyl acetamide	Unknown	N/A	S	N	NICEATM	Unknown	Unknown	1 of 1	Cat 2B	**IR mean ≥ 1**; CO mean ≥ 1; Conj mean ≥ 2	3/3; 2/3; 2/3						?
245	2,2-Dimethyl-3-pentanol	3970-62-5	Alcohol| Alkane branched with quaternary carbon| tert-Butyl	L	Y	ECETOC	Sigma-Aldrich	97%	1 of 1	No Cat	CO > 0 **	** CO 1/3						
246	2-Ethoxyethanol	110-80-5	Alcohol| Alkoxy| Ether	L	Y	LNS	Sigma-Aldrich	≥99%	1 of 1	No Cat	CO > 0 **	** CO 1/3						
247	3-Phenoxybenzyl alcohol	13826-35-2	Alcohol| Aryl| Benzyl| Ether	L	Y	NICEATM	Sigma-Aldrich	98%	1 of 1	No Cat	CO > 0 **	** CO 1/3						
248	alpha-Hexyl cinnamic aldehyde (12.5% in Acohol SDA 39C)	101-86-0	Alpha,beta unsaturated aldehyde| Benzyl	L	Y	NICEATM	Sigma-Aldrich (for alpha-Hexyl cinnamic aldehyde); Fisher Scientific (for Alcohol SDA 39C)	≥95%	1 of 1	No Cat	CO > 0 **	** CO 1/3; Conj 1/3					SDA = special denatured alcohol (99.01% (v/v) Ethanol + 0.99% (v/v) Diethylphthalate)	X
249	Benzotrichloride	98-07-7	Alkyl halide| Aryl	L	Y	NICEATM	Sigma-Aldrich	98%	1 of 1	No Cat	CO > 0 **	** Conj 3/6						
250	Cellosolve acetate	111-15-9	Acetoxy| Alkoxy| Carboxylic acid ester| Ether	L	Y	ECETOC	Sigma-Aldrich	98%	1 of 1	No Cat	CO > 0 **	** CO 1/4						
251	Cyclohexanone	108-94-1	Cycloalkane| Cycloketone	L	Y	LNS	Sigma-Aldrich	≥99.5%	1 of 1	No Cat	CO > 0 **	** CO 1/3						
252	Ethyl thioglycolate	623-51-8	Carboxylic acid ester| Thioalcohol	L	Y	ECETOC	Sigma-Aldrich	97%	1 of 1	No Cat	CO > 0 **	** CO 1/3; Conj 1/3; IR 1/3						
253	Ethyltrimethyl acetate	3938-95-2	Carboxylic acid ester| tert-Butyl	L	Y	ECETOC	Sigma-Aldrich	99%	1 of 1	No Cat	CO > 0 **	** CO 1/6						
254	Glycidyl methacrylate	106-91-2	Epoxide| Methacrylate| Saturated heterocyclic fragment	L	Y	ECETOC	Sigma-Aldrich	≥97%	1 of 1	No Cat	CO > 0 **	** CO 1/3; Conj 1/3; IR 1/3						
255	Lactic acid (10%)	50-21-5	Alcohol| Carboxylic acid	L	Y	NICEATM	Sigma-Aldrich	90%	1 of 1	No Cat	CO > 0 **	** Conj 1/3						
256	Methanol	67-56-1	Alcohol	L	Y	LNS	Sigma-Aldrich	≥99.9%	1 of 1	No Cat	CO > 0 **	** CO 1/3; Conj 1/3						
257	Methyl amyl ketone (1 of 2)	110-43-0	Ketone	L	Y	ECETOC	Sigma-Aldrich	99%	1 of 2	No Cat	CO > 0 **	** Conj 1/4						
258	Methylal	109-87-5	Acetal	L	Y	NICEATM	Sigma-Aldrich	99%	1 of 1	No Cat	CO > 0 **	** CO 1/6					A second study is also reported in NICEATM ALTTOX with the exact same animal scores and is considered a duplicate entry	
259	Metolachlor	51218-45-2	Aryl| Ether| Haloacetamide	L	Y	EURL ECVAM	Sigma-Aldrich	analytical standard	1 of 1	No Cat	CO > 0 **	** Conj 1/6						
260	Phosphoric acid, tributyl ester (also known as Tributyl phosphate) (1 of 2)	126-73-8	Alkoxy| Phosphate ester	L	Y	NICEATM	Sigma-Aldrich	≥ 99%	1 of 2	No Cat	CO > 0 **	** CO 1/3; Conj 1/3						X
261	Polyalkenylsuccinate ester/amine salt	Unknown	N/A	L	N	NICEATM	Unknown	Unknown	1 of 1	No Cat	CO > 0 **	** Conj 2/6						?
262	Trimethoxy(3,3,3-trifluoropropyl)silane	429-60-7	AlkoxySilanes| Alkyl halide	L	Y	EURL ECVAM	Sigma-Aldrich	≥97%	1 of 1	No Cat	CO > 0 **	** Conj 1/3; IR 1/3						
263	Triton X-15 surfactant	9036-19-5	Alcohol| Alkane branched with quaternary carbon| Aryl| Ether| tert-Butyl	L	Y	NICEATM	Sigma-Aldrich	50-100%	1 of 1	No Cat	CO > 0 **	** CO 1/4; Conj 1/4						
264	Di-iso-propanolamine (10%)	110-97-4	Aliphatic Amine, secondary| Dihydroxyl group	L (tested in solvent, available as S)	Y	NICEATM	Sigma-Aldrich	≥ 98%	1 of 1	No Cat	CO > 0 **	** CO 1/3						
265	N-Lauroyl sarcosine Na salt (30%) (Sodium-N-lauroyl sarcosinate reported in NICEATM ALTTOX)	137-16-6	Carboxamide| Carboxylic acid	L (tested in solvent, available as S)	Y	NICEATM	Sigma-Aldrich	≥99%	1 of 1	No Cat	CO > 0 **	** CO 1/3						X
266	Sodium lauryl sulphate (3%)	151-21-3	Alkoxy| Sulfate	L (tested in solvent, available as S)	Y	ECETOC	Sigma-Aldrich	≥99%	1 of 1	No Cat	CO > 0 **	** CO 1/6						
267	1,2-Epoxycyclooctane	286-62-4	Cycloalkane| Epoxide| Fused saturated heterocycles| Saturated heterocyclic fragment	S	Y	ZEBET	Sigma-Aldrich	99%	1 of 1	No Cat	CO > 0 **	** CO 1/3						
268	Alcohol sulfate mono-C16-18	68955-20-4	Sulfate	S	Y	EURL ECVAM	Proprietary	93.4%	1 of 1	No Cat	CO > 0 **	** CO 2/4; Conj 2/4						?
269	Fluoxastrobin	361377-29-9	Aromatic heterocyclic halide| Aryl| Aryl halide| Ether| Ketoxime derivatives| Unsaturated heterocyclic fragment	S	Y	EURL ECVAM	Sigma-Aldrich	analytical standard	1 of 1	No Cat	CO > 0 **	** CO 1/3						
270	Iodosulphuron-methyl-sodium	144550-36-7	Aromatic heterocyclic halide| Aryl| Aryl halide| Carboxylic acid ester| Ether| Sulfonamide| Sulfonyl urea| Triazine| Urea derivatives	S	Y	EURL ECVAM	Chemos GmbH	90.8%	1 of 1	No Cat	CO > 0 **	** CO 1/3; Conj 1/3						
271	Sodium bisulphite	7631-90-5	Inorganic Salt	S	Y	ZEBET	Sigma-Aldrich	100%	1 of 1	No Cat	CO > 0 **	** CO 1/3; Conj 1/3						
272	2-Nitro-4-thio-N-propylaniline	54393-89-4	Aniline| Nitrobenzene| Sulfide	Unknown	N	NICEATM	Unknown	Unknown	1 of 1	No Cat	CO > 0 **	** Conj 1/3; IR 1/3						?
273	3-Hydroxy-2-phenyl-4-quinolinecarboxylic acid	485-89-2	Carboxylic acid| Heterocyclic Phenol| Quinoline/ Isoquinoline	Unknown	N	NICEATM	Unknown	Unknown	1 of 1	No Cat	CO > 0 **	** CO 1/3; Conj 1/3; IR 1/3						?
274	p-tert-Butylbenzoic acid, triethanolamine salt (neat)	59993-86-1 (98-73-7 reported in NICEATM ALTTOX, which corresponds to 4-tert-Butylbenzoic acid)	Alcohol| Aliphatic Amine, tertiary| Aryl| Carboxylic acid| Isopropyl	Unknown	N	NICEATM	Unknown	Unknown	1 of 1	No Cat	CO > 0 **	** CO 1/6						?
275	1,2,3-Trichloropropane (1 of 2)	96-18-4	Alkyl halide	L	Y	LNS	Sigma-Aldrich	99%	1 of 2	No Cat	CO > 0							
276	2,4-Pentanedione	123-54-6	Diketone	L	Y	LNS	Sigma-Aldrich	≥99.5%	1 of 1	No Cat	CO > 0							
277	2-Ethylhexanoyl chloride (neat)	760-67-8	Acyl halide	L	Y	NICEATM	Sigma-Aldrich	98%	1 of 1	No Cat	CO > 0						CO = 2 on Hr1 in 1/6; CO = 1 on Hr1 in 1/6	
278	2-Methoxyethanol	109-86-4	Alcohol| Ether	L	Y	LNS	Sigma-Aldrich	≥99.9%	1 of 1	No Cat	CO > 0							
279	Dimethyl carbonate	616-38-6	Carbonate	L	Y	NICEATM	Sigma-Aldrich	≥99%	1 of 1	No Cat	CO > 0							
280	Dimethyl sulfoxide (DMSO)	67-68-5	Sulfoxide	L	Y	LNS	Sigma-Aldrich	≥99.9%	1 of 1	No Cat	CO > 0						CO = 1 on Hr1 in 1/3	
281	Ethyl acetate	141-78-6	Acetoxy| Carboxylic acid ester	L	Y	ECETOC	Sigma-Aldrich	99.9%	1 of 1	No Cat	CO > 0							
282	Ethyl acetoacetate	141-97-9	Carboxylic acid ester| Ketone	L	Y	LNS	Sigma-Aldrich	≥99%	1 of 1	No Cat	CO > 0							
283	Furan	110-00-9	Aryl| Furane	L	Y	LNS	Sigma-Aldrich	≥99%	1 of 1	No Cat	CO > 0							
284	Methyl amyl ketone (2 of 2)	110-43-0	Ketone	L	Y	ECETOC	Sigma-Aldrich	99%	2 of 2	No Cat	CO > 0							

Study Number	Test Chemical Name	CAS RN	Organic Functional Groups	Physical Form as tested	Physical Form Confirmation e.g., by MSDS (Y / N)	Data Source	Commercial Source	Available Purity	Number of Studies	UN GHS Classification	Drivers of Classification						Comments	Should Not Be Used
											Severity		Persistence		Specific Observations			
											Cut-off values	Number of animals	Cut-off time	Number of animals	CO = 4 or other observations	Number of animals		
285	Methyl iso-butyl ketone (1 of 2)	108-10-1	Isopropyl| Ketone	L	Y	LNS	Sigma-Aldrich	≥99.5%	1 of 2	No Cat	CO > 0							
286	Methyl iso-butyl ketone (2 of 2)	108-10-1	Isopropyl| Ketone	L	Y	ECETOC	Sigma-Aldrich	≥99.5%	2 of 2	No Cat	CO > 0							
287	n-Butyl acetate	123-86-4	Acetoxy| Carboxylic acid ester	L	Y	ECETOC	Sigma-Aldrich	≥99.5%	1 of 1	No Cat	CO > 0							
288	Propiconazole	60207-90-1	Aryl| Aryl halide| Dioxolane| Saturated heterocyclic fragment| Triazole	L	Y	EURL ECVAM	Chemos GmbH	95.2%	1 of 1	No Cat	CO > 0							
289	Propylidynetrimethanol, propoxylated	25723-16-4	Alcohol| Alkane branched with quaternary carbon| Ether	L	Y	NICEATM	Sigma-Aldrich	Unknown	1 of 1	No Cat	CO > 0							
290	Sodium polyoxyethylene laurylether sulphate (27%, aqueous)	9004-82-4 (68585-34-2 reported in NICEATM ALTTOX, which corresponds to Sodium lauryl ether sulphate)	Alkoxy| Ether| Sulfate	L	Y	NICEATM	Oxiteno (ALKOPON® N)	27%, aqueous	1 of 1	No Cat	CO > 0							
291	Styrene	100-42-5	Alkene| Aryl	L	Y	ECETOC	Sigma-Aldrich	≥99%	1 of 1	No Cat	CO > 0							
292	Triethanolamine (neat) (1 of 2)	102-71-6	Alcohol| Aliphatic Amine, tertiary	L	Y	NICEATM	Sigma-Aldrich	≥99.5%	1 of 2	No Cat	CO > 0							
293	Triton X-155 (10%)	9010-44-0	N/A	L (tested in solvent, neat chemical unknown)	Y	LNS	Unknown	Unknown	1 of 1	No Cat	CO > 0						CO = 1 on Hr1 in 1/3	?
294	Cetyl pyridinium bromide (0.1%)	140-72-7	Ammonium salt| Aryl| Pyridine	L (tested in solvent, available as S)	Y	ECETOC	Sigma-Aldrich	98%	1 of 1	No Cat	CO > 0							
295	m-Phenylene diamine (10%)	108-45-2	Aminoaniline, meta	L (tested in solvent, available as S)	Y	NICEATM	Sigma-Aldrich	≥99%	1 of 1	No Cat	CO > 0						CO = 1 between Hr1 and Hr4 in 1/3	
296	Sodium lauryl sulphate (1%) (1 of 2)	151-21-3	Alkoxy| Sulfate	L (tested in solvent, available as S)	Y	NICEATM	Sigma-Aldrich	≥99%	1 of 2	No Cat	CO > 0						CO = 1 between Hr1 and Hr4 in 1/3	
297	Sodium lauryl sulphate (1%) (2 of 2)	151-21-3	Alkoxy| Sulfate	L (tested in solvent, available as S)	Y	NICEATM	Sigma-Aldrich	≥99%	2 of 2	No Cat	CO > 0						CO = 1 on Hr4 in 1/3	
298	Sucrose fatty acid ester (10%)	Unknown	N/A	L (tested in solvent, available as S)	Y	NICEATM	Unknown	Unknown	1 of 1	No Cat	CO > 0							?
299	(-)Phenylephrine	59-42-7	Alcohol| Aliphatic Amine, secondary| Phenol	S	Y	ZEBET	SCBT	98%	1 of 1	No Cat	CO > 0						CO = 1 on Hr1 in 1/4	
300	(+)Phenylephrine	614-03-9	Alcohol| Aliphatic Amine, secondary| Phenol	S	Y	ZEBET	Unknown	Unknown	1 of 1	No Cat	CO > 0							?
301	1,3,5-Trioxane	110-88-3	Acetal| Saturated heterocyclic fragment| Trioxane	S	Y	ZEBET	Sigma-Aldrich	≥99%	1 of 1	No Cat	CO > 0						CO = 2 on Hr1 in 1/3	
302	1-Phenyl-3-pyrazolidone	92-43-3	Aryl| Pyrazolidinedione/ Pyrazolidone	S	Y	LNS	Sigma-Aldrich	97%	1 of 1	No Cat	CO > 0							
303	2,4-Dichloro-5-sulphamoyl-benzoic acid	2736-23-4	Aryl halide| Carboxylic acid| Sulfonamide	S	Y	LNS	Sigma-Aldrich	98%	1 of 1	No Cat	CO > 0							
304	2-(Acetyloxy)-1-phenylethanone	2243-35-8	Acetoxy| Aryl| Carboxylic acid ester| Ketone	S	Y	NICEATM	Alfa Aesar	98%	1 of 1	No Cat	CO > 0							
305	5-Chloro-3-methylbenzo[b]thiophene-2-sulphonyl chloride	166964-33-6	Allyl| Aryl halide| Benzothiophene/ Benzoisothiophene| Sulfonyl halide	S	Y	NICEATM	Fisher Scientific	97%	1 of 1	No Cat	CO > 0							
306	5-Methyl-1,3,4-thiadiazol-2-amine	108-33-8	Amidine| Aryl| Thiadiazole	S	Y	ZEBET	Sigma-Aldrich	97%	1 of 1	No Cat	CO > 0						CO = 1 on Hr1 in 3/3	
307	6-Nitro-1,2,3,4-tetrahydroquinoxaline	41959-35-7	Fused saturated heterocycles| Nitrobenzene	S	Y	Cosmetics Europe	Fluorochem	≥95%	1 of 1	No Cat	CO > 0							
308	Acrylamidopropyltrimonium chloride/acrylamide copolymer	75150-29-7	Acrylamide| Ammonium salt	S	Y	EURL ECVAM	Proprietary	≥94%	1 of 1	No Cat	CO > 0							?
309	Betaine monohydrate	590-47-6	Ammonium salt| Carboxylic acid	S	Y	LNS	Sigma-Aldrich	99%	1 of 1	No Cat	CO > 0						CO = 1 on Hr1 in 2/3	
310	Bromethalin	63333-35-7	Alkyl halide| Aromatic amine| Aryl halide| Nitrobenzene	S	Y	EURL ECVAM	Sigma-Aldrich	analytical standard	1 of 1	No Cat	CO > 0							
311	Dimethyl biguanide	657-24-9	Guanidine	S	Y	LNS	Unknown	Unknown	1 of 1	No Cat	CO > 0							?
312	DL-Glutamic acid	19285-83-7	Alpha amino acid| Carboxylic acid	S	Y	LNS	Sigma-Aldrich	>99%	1 of 1	No Cat	CO > 0							
313	Ethylenediaminetetraacetic acid dipotassium salt (EDTA di-K salt)	25102-12-9	Aliphatic Amine, tertiary| Carboxylic acid	S	Y	LNS	Sigma-Aldrich	≥99%	1 of 1	No Cat	CO > 0							
314	Iminodibenzyl	494-19-9	Fused carbocyclic aromatic| Fused saturated heterocycles| Precursors quinoid compounds	S	Y	LNS	Sigma-Aldrich	97%	1 of 1	No Cat	CO > 0							
315	Magnesium carbonate	56378-72-4	N/A	S	Y	LNS	Sigma-Aldrich	99%	1 of 1	No Cat	CO > 0							
316	Propyl-4-hydroxybenzoate	94-13-3	Carboxylic acid ester| Phenol	S	Y	LNS	Sigma-Aldrich	≥99%	1 of 1	No Cat	CO > 0							
317	Tetra aminopyrimidine sulphate (2 of 2)	5392-28-9	Anilines| Anilines(meta)| Anilines(ortho)| Anilines(para)	S	Y	ECETOC	Sigma-Aldrich	97%	2 of 2	No Cat	CO > 0							X
318	1-(5-Amino-2-methoxyphenyl) piperazine hydrochloride	Unknown (CAS # for 1-(5-Amino-2-methoxyphenyl) piperazine is 148546-90-1)	N/A	Unknown	N	NICEATM	Unknown	Unknown	1 of 1	No Cat	CO > 0							?
319	2-Nitro-4-propoxyaniline	20367-34-4	Alkoxy| Aniline| Ether| Nitrobenzene	Unknown	N	NICEATM	Unknown	Unknown	1 of 1	No Cat	CO > 0							?
320	Piperonyl butoxide	51-03-6	Alkoxy| Benzodioxole| Benzyl| Ether	L	Y	EURL ECVAM	Chemos GmbH	94.8%	1 of 1	No Cat	CO = 0 **	** Conj 2/6						
321	Toluene (1 of 2)	108-88-3	Aryl	L	Y	ECETOC	Sigma-Aldrich	≥99.9%	1 of 2	No Cat	CO = 0 **	** Conj 1/4						X
322	Xylene (1 of 2)	1330-20-7	Imide	L	Y	ECETOC	Sigma-Aldrich	≥98.5% (Xylene mixture of isomers)	1 of 2	No Cat	CO = 0 **	** Conj 1/4						
323	Triethanolamine orthovanadate (30%)	13476-99-8	Diketone	L (tested in solvent, available as S)	Y	NICEATM	Sigma-Aldrich	97%	1 of 1	No Cat	CO = 0 **	** Conj 2/6						
324	N,N-Dimethyl guanidine sulphate	598-65-2	Aliphatic Amine, tertiary| Amidine| Guanidine	S	Y	ECETOC	Sigma-Aldrich	97%	1 of 1	No Cat	CO = 0 **	** Conj 1/3						
325	6-(Methylamino)-2-pyridine ethanol formate (1:1) (salt)	Intermediate - No CAS	N/A	Unknown	N	NICEATM	Unknown	Unknown	1 of 1	No Cat	CO = 0 **	** Conj 1/3						?
326	1,2,3-Propanetricarboxylic acid, 2-hydroxy-, monohydrate (9CI) and 1,2,3-Propanetricarboxylic acid, 2-hydroxy-, silver(1+) salt, monohydrate	Unknown	N/A	L	Y	EURL ECVAM	Proprietary	Unknown	1 of 1	No Cat	CO = 0							?
327	1,2,3-Trichloropropane (2 of 2)	96-18-4	Alkyl halide	L	Y	NICEATM	Sigma-Aldrich	99%	2 of 2	No Cat	CO = 0							
328	1,2,4-Trimethylbenzene	95-63-6	Aryl	L	Y	LNS	Sigma-Aldrich	98%	1 of 1	No Cat	CO = 0							
329	1,2,6-Hexanetriol	106-69-4	Alcohol| Dihydroxyl group	L	Y	ZEBET	Sigma-Aldrich	96%	1 of 1	No Cat	CO = 0							
330	1,3-Dibromopropane	109-64-8	Alkyl halide	L	Y	ECETOC	Sigma-Aldrich	99%	1 of 1	No Cat	CO = 0							
331	1,3-Di-iso-propyl benzene	99-62-7	Aryl| Isopropyl	L	Y	ECETOC	Sigma-Aldrich	96%	1 of 1	No Cat	CO = 0							
332	1,4-Dibromobutane	110-52-1	Alkyl halide	L	Y	ECETOC	Sigma-Aldrich	99%	1 of 1	No Cat	CO = 0							
333	1,5-Dibromopentane	111-24-0	Alkyl halide	L	Y	ECETOC	Sigma-Aldrich	97%	1 of 1	No Cat	CO = 0							
334	1,5-Hexadiene	592-42-7	Allyl	L	Y	ECETOC	Sigma-Aldrich	97%	1 of 1	No Cat	CO = 0							

Cosmetics Europe
Draize eye test Reference Database (DRD)
Barroso *et al.* , 2016

Study Number	Test Chemical Name	CAS RN	Organic Functional Groups	Physical Form as tested	Physical Form Confirmation e.g., by MSDS (Y / N)	Data Source	Commercial Source	Available Purity	Number of Studies	UN GHS Classification	Drivers of Classification						Comments	Should Not Be Used
											Severity		Persistence		Specific Observations			
											Cut-off values	Number of animals	Cut-off time	Number of animals	CO = 4 or other observations	Number of animals		
335	1,6-Dibromohexane	629-03-8	Alkyl halide	L	Y	ECETOC	Sigma-Aldrich	96%	1 of 1	No Cat	CO = 0							
336	1,9-Decadiene	1647-16-1	Allyl	L	Y	ECETOC	Sigma-Aldrich	98%	1 of 1	No Cat	CO = 0							
337	1-(2,6-Dimethylphenoxy)-2-propanol	61102-09-8 (53012-41-2 reported in ZEBET, which corresponds to 1-(2,6-Dimethylphenoxy)acetone)	Alcohol| Aryl| Ether	L	N	ZEBET	Unknown	Unknown	1 of 1	No Cat	CO = 0							?
338	1-Bromo-4-chlorobutane	6940-78-9	Alkyl halide	L	Y	ECETOC	Sigma-Aldrich	99%	1 of 1	No Cat	CO = 0							
339	1-Ethyl-3-methylimidazolium ethyl sulphate	342573-75-5	Alkoxy| Ammonium salt| Aryl| Imidazole| Sulfate	L	Y	EURL ECVAM	Sigma-Aldrich	≥95%	1 of 1	No Cat	CO = 0							
340	1-Methylpropylbenzene	135-98-8	Aryl	L	Y	ECETOC	Sigma-Aldrich	≥99%	1 of 1	No Cat	CO = 0							
341	1-Nitropropane	108-03-2	Nitro aliphatic	L	Y	LNS	Sigma-Aldrich	≥98.5%	1 of 1	No Cat	CO = 0							
342	2,2',2''-Nitrilotriethaniol, propoxylated	37208-53-0	Alcohol| Aliphatic Amine, tertiary| Ether	L	Y	NICEATM	Oltchim	>99%	1 of 1	No Cat	CO = 0							
343	2,4-Dicyano-1-butene	1572-52-7	Allyl| Nitrile	L	Y	NICEATM	TCI	>98%	1 of 1	No Cat	CO = 0							
344	2,4-Pentanediol	625-69-4	Dihydroxyl group	L	Y	ECETOC	Sigma-Aldrich	98%	1 of 1	No Cat	CO = 0							
345	2-(2-Ethoxy ethoxy) ethanol	111-90-0	Alcohol| Alkoxy| Ether	L	Y	EURL ECVAM	Sigma-Aldrich	≥99%	1 of 1	No Cat	CO = 0							
346	2-Chloro-2,4,4-trimethylpentane	6111-88-2	Alkane branched with quaternary carbon| Alkyl halide| tert-Butyl	L	Y	NICEATM	Fluorochem	100%	1 of 1	No Cat	CO = 0							
347	2-Ethoxyethyl methacrylate	2370-63-0	Alkoxy| Ether| Methacrylate	L	Y	ECETOC	Sigma-Aldrich	99%	1 of 1	No Cat	CO = 0							
348	2-Ethylhexyl p-dimethylamino benzoate (10%)	21245-02-3	Alkane, branched with tertiary carbon| Aromatic amine| Carboxylic acid ester	L	Y	NICEATM	Sigma-Aldrich	98%	1 of 1	No Cat	CO = 0							
349	2-Ethylhexyl p-dimethylamino benzoate (neat)	21245-02-3	Alkane, branched with tertiary carbon| Aromatic amine| Carboxylic acid ester	L	Y	NICEATM	Sigma-Aldrich	98%	1 of 1	No Cat	CO = 0							
350	2-Ethylhexylthioglycolate	7659-86-1 (7659-86-12 reported in ECETOC, which is wrong)	Alkane, branched with tertiary carbon| Carboxylic acid ester| Thioalcohol	L	Y	ECETOC	Sigma-Aldrich	≥95%	1 of 1	No Cat	CO = 0							
351	2-Methoxy-3,4-dihydropyran (neat)	4454-05-1 (103-75-3 reported in NICEATM ALTTOX, which corresponds to 2-Ethoxy-3,4-dihydropyran)	Allyl| Dihydropyran| Ether| Unsaturated heterocyclic fragment	L	Y	NICEATM	TCI	>97%	1 of 1	No Cat	CO = 0							
352	2-Methylpentane	107-83-5	Isopropyl	L	Y	ECETOC	Sigma-Aldrich	≥99%	1 of 1	No Cat	CO = 0							
353	2-Propylheptyl octanoate	868839-23-0	Alkane, branched with tertiary carbon| Carboxylic acid ester	L	Y	EURL ECVAM	Proprietary	Unknown	1 of 1	No Cat	CO = 0							?
354	3,3-Dimethylpentane	562-49-2	Alkane branched with quaternary carbon	L	Y	ECETOC	Sigma-Aldrich	99%	1 of 1	No Cat	CO = 0							
355	3-Ethyl toluene	620-14-4	Aryl	L	Y	ECETOC	Sigma-Aldrich	99%	1 of 1	No Cat	CO = 0							
356	3-Methoxy-1,2-propanediol	623-39-2	Dihydroxyl group| Ether| Glycerol and derivatives	L	Y	ECETOC	TCI	>98%	1 of 1	No Cat	CO = 0							
357	3-Methylhexane	589-34-4	Alkane, branched with tertiary carbon	L	Y	ECETOC	Sigma-Aldrich	99%	1 of 1	No Cat	CO = 0							
358	3-Phenoxy benzaldehyde (neat) (1 of 2)	39515-51-0	Aldehyde| Aryl| Ether	L	Y	NICEATM	Sigma-Aldrich	98%	1 of 2	No Cat	CO = 0							
359	3-Phenoxy benzaldehyde (neat) (2 of 2)	39515-51-0	Aldehyde| Aryl| Ether	L	Y	NICEATM	Sigma-Aldrich	98%	2 of 2	No Cat	CO = 0							
360	4-Bromophenetole	588-96-5 (589-10-6 reported in ECETOC, which corresponds to beta-Bromophenetole)	Alkoxy| Aryl halide| Ether	L	Y	ECETOC	Sigma-Aldrich	98%	1 of 1	No Cat	CO = 0							
361	Allyl methacrylate	96-05-9	Allyl| Methacrylate	L	Y	ECETOC	Sigma-Aldrich	98%	1 of 1	No Cat	CO = 0							
362	alpha-Hexyl cinnamic aldehyde (12.5% in Petrolatum (87.5%))	101-86-0	Alpha,beta unsaturated aldehyde| Benzyl	L	Y	NICEATM	Sigma-Aldrich (for alpha-Hexyl cinnamic aldehyde); Unknown (for Petrolatum)	≥95%	1 of 1	No Cat	CO = 0							X
363	alpha-Hexyl cinnamic aldehyde (6.25% in Alcohol SDA 39C)	101-86-0	Alpha,beta unsaturated aldehyde| Benzyl	L	Y	NICEATM	Sigma-Aldrich (for alpha-Hexyl cinnamic aldehyde); Fisher Scientific (for Alcohol SDA 39C)	≥95%	1 of 1	No Cat	CO = 0						SDA = special denatured alcohol (99.01% (v/v) Ethanol + 0.99% (v/v) Diethylphthalate)	X
364	Amyl acetate (neat)	628-63-7	Acetoxy| Carboxylic acid ester	L	Y	NICEATM	Sigma-Aldrich	≥99%	1 of 1	No Cat	CO = 0							
365	Bicyclo [2,2,1]hept-5-ene-2-carbonitrile (P30671)	95-11-4	Bridged-ring carbocycles| Nitrile	L	Y	NICEATM	Sigma-Aldrich	98%	1 of 1	No Cat	CO = 0						One of the six animals exhbited depression, emaciation and marked diarrhea; Death was noted prior to the 72 hour reading; From gross observation, death was not attributed to sample application	
366	Bisphenol A, diethylene triamine, epichlorohydrin polymer, ethoxylated, propoxylated (56%, aqueous emulsion)	455946-46-0	Alcohol| Aliphatic Amine, primary| Aliphatic Amine, secondary| Alkane, branched with quaternary carbon| Alkyl halide| Epoxide| Ether| Phenol| Saturated heterocyclic fragment	L	Y	EURL ECVAM	Proprietary	56%, aqueous	1 of 1	No Cat	CO = 0							?
367	Bisphenol A, epichlorohydrin polymer, ethoxylated, propoxylated (53-57%, aqueous emulsion)	68123-18-2	Alkyl halide| Epoxide| Phenol| Saturated heterocyclic fragment	L	Y	EURL ECVAM	Proprietary	~55%, aqueous	1 of 1	No Cat	CO = 0							?
368	bis-(3-Triethoxisilylpropyl)-tetrasulphide	40372-72-3	Alkoxy| AlkoxySilane| Sulfide, poly	L	Y	ZEBET	Sigma-Aldrich	≥90%	1 of 1	No Cat	CO = 0							
369	Bromo-2-butane	78-76-2	Alkyl halide	L	Y	ECETOC	Sigma-Aldrich	98%	1 of 1	No Cat	CO = 0							
370	Carsonon N-9 (Chemical name: Nonyl phenol ethoxylate, branched) (neat)	9016-45-9 (127087-87-0 reported in NICEATM ALTTOX, which corresponds to Tergitol NP nonionic surfactants)	Alcohol| Benzyl| Ether	L	Y	NICEATM	Unknown	Unknown	1 of 1	No Cat	CO = 0						Data missing for D1	?
371	cis-Cyclooctene	931-87-3 (931-88-4 reported in ECETOC, which corresponds to Cyclooctene)	Allyl| Cycloalkene	L	Y	ECETOC	Sigma-Aldrich	95%	1 of 1	No Cat	CO = 0							
372	Clarified slurry oil	64741-62-4	Fused carbocyclic aromatic	L	Y	NICEATM	Unknown	Unknown	1 of 1	No Cat	CO = 0							?
373	Dichlorotoluenes	Mixture	N/A	L	N	ECETOC	Unknown	Unknown	1 of 1	No Cat	CO = 0							?
374	Dimethylhydropolysiloxane (neat)	68037-59-2	Allyl| Aryl halide| Benzamide| Sarcosine	L	Y	NICEATM	Sigma-Aldrich	Unknown	1 of 1	No Cat	CO = 0							
375	Dimethyl siloxane, mono dimethylvinylsiloxy- and mono trimethoxysiloxy-terminated (95%)	471277-16-4	Alkene| AlkoxySilane| Silane	L	Y	EURL ECVAM	Proprietary	100%	1 of 1	No Cat	CO = 0							?
376	Dioctyl carbonate	1680-31-5	Carbonate	L	Y	EURL ECVAM	Proprietary	97%	1 of 1	No Cat	CO = 0							?
377	Dioctyl ether	629-82-3	Alkoxy| Ether	L	Y	EURL ECVAM	Sigma-Aldrich	99%	1 of 1	No Cat	CO = 0							
378	Di-iso-butyl ketone	108-83-8	Isopropyl| Ketone	L	Y	ECETOC	Sigma-Aldrich	≥99%	1 of 1	No Cat	CO = 0							
379	Di-n-propyl disulphide	629-19-6	Disulfide	L	Y	ECETOC	Sigma-Aldrich	98%	1 of 1	No Cat	CO = 0							
380	Dodecane	112-40-3	Methyl| Methylene	L	Y	ECETOC	Sigma-Aldrich	≥99%	1 of 1	No Cat	CO = 0							

Study Number	Test Chemical Name	CAS RN	Organic Functional Groups	Physical Form as tested	Physical Form Confirmation e.g., by MSDS (Y / N)	Data Source	Commercial Source	Available Purity	Number of Studies	UN GHS Classification	Drivers of Classification: Severity		Persistence		Specific Observations		Comments	Should Not Be Used
											Cut-off values	Number of animals	Cut-off time	Number of animals	CO = 4 or other observations	Number of animals		
381	Dow Corning Syltherm 444 heat transfer fluid (Chemical name: Polydimethylsiloxane)	63148-62-9	Silane	L	Y	NICEATM	Sigma-Aldrich	Unknown	1 of 1	No Cat	CO = 0							
382	Dow Corning X201133 heat transfer fluid (Chemical name: Polydimethylsiloxane)	63148-62-9	Silane	L	Y	NICEATM	Sigma-Aldrich	Unknown	1 of 1	No Cat	CO = 0							
383	Dynasylan VPS 8815 (Chemical name: Siloxanes and Silicones, 3-[[2-[(2-aminoethyl)amino]ethyl]amino]propyl hydroxy, hydroxy 3,3,4,4,5,5,6,6,7,7,8,8,8-tridecafluorooctyl, hydroxy-terminated, formates (salts))	273737-91-0	N/A	L	Y	NICEATM	Unknown	Unknown	1 of 1	No Cat	CO = 0							?
384	Ethanol (10%)	64-17-5	Alcohol	L	Y	NICEATM	Sigma-Aldrich	>99.8%	1 of 1	No Cat	CO = 0							
385	Ethylene oxide	75-21-8	Epoxide| Saturated heterocyclic fragment	L	Y	NICEATM	Sigma-Aldrich	≥99.9%	1 of 1	No Cat	CO = 0						Liquefied gaz	
386	Ethyleneglycol diethyl ether	629-14-1	Alkoxy| Ether	L	Y	ECETOC	Sigma-Aldrich	98%	1 of 1	No Cat	CO = 0							
387	Ethylthioethyl methacrylate	14216-25-2	Methacrylate| Sulfide	L	Y	ECETOC	Unknown	Unknown	1 of 1	No Cat	CO = 0							?
388	Ethyltriglycol methacrylate	39670-09-2 (36670-09-2 reported in ECETOC, which is wrong)	Alkoxy| Ether| Methacrylate	L	Y	ECETOC	Unknown	Unknown	1 of 1	No Cat	CO = 0							?
389	Fungitrol zinc 8% fungicide (Chemical name: Zinc naphthenate) (neat)	12001-85-3	Alkane, branched with tertiary carbon| Carboxylic acid| Cycloalkane	L	Y	NICEATM	Sigma-Aldrich	Unknown	1 of 1	No Cat	CO = 0							
390	gamma-Chloropropyltrimethoxy silane (neat)	2530-87-2	AlkoxySilane| Alkyl halide	L	Y	NICEATM	Sigma-Aldrich	≥97%	1 of 1	No Cat	CO = 0							
391	gamma-Glycidyloxypropyltrimethoxy silane (3-Glycidoxypropyltrimethoxysilane reported in LNS) (1 of 2)	2530-83-8	AlkoxySilane| Epoxide| Ether| Saturated heterocyclic fragment	L	Y	LNS	Sigma-Aldrich	≥98%	1 of 2	No Cat	CO = 0							
392	gamma-Glycidyloxypropyltrimethoxy silane ((3-Glycidoxypropyl)trimethoxy silane reported in NICEATM ALTTOX) (2 of 2)	2530-83-8	AlkoxySilane| Epoxide| Ether| Saturated heterocyclic fragment	L	Y	NICEATM	Sigma-Aldrich	≥98%	2 of 2	No Cat	CO = 0							
393	gamma-Mercaptopropyl trimethoxy silane (3-Mercaptopropyl trimethoxy silane reported in NICEATM ALTTOX) (neat) (1 of 2)	4420-74-0 (2530-87-2 reported in NICEATM ALTTOX, which corresponds to 3-Chloropropyl trimethoxy silane)	AlkoxySilane| Thioalcohol	L	Y	NICEATM	Sigma-Aldrich	95%	1 of 2	No Cat	CO = 0							
394	gamma-Mercaptopropyl trimethoxy silane (neat) (2 of 2)	4420-74-0	AlkoxySilane| Thioalcohol	L	Y	NICEATM	Sigma-Aldrich	95%	2 of 2	No Cat	CO = 0							
395	gamma-Methacryloxypropyltrimethoxy silane	2530-85-0	AlkoxySilane| Methacrylate	L	Y	NICEATM	Sigma-Aldrich	≥98%	1 of 1	No Cat	CO = 0							
396	Genagen (Chemical name: Polyethylene glycol 400 dilaurate)	68139-91-3	Carboxylic acid ester| Ether	L	N	ZEBET	Unknown	Unknown	1 of 1	No Cat	CO = 0							?
397	Genomoll P (Chemical name: Tris(2-chloroethyl) phosphate)	115-96-8	Alkyl halide| Phosphate ester	L	Y	ZEBET	Sigma-Aldrich	97%	1 of 1	No Cat	CO = 0							
398	Glycediol	556-52-5	Alcohol| Epoxide| Saturated heterocyclic fragment	L	Y	ZEBET	Sigma-Aldrich	96%	1 of 1	No Cat	CO = 0							
399	Glycerol (Glycerin reported in NICEATM ALTTOX) (10%)	56-81-5	Dihydroxyl group| Glycerol and derivatives	L	Y	NICEATM	Sigma-Aldrich	≥99.5%	1 of 1	No Cat	CO = 0							
400	Glycerol (Glycerin reported in NICEATM ALTTOX) (neat) (1 of 2)	56-81-5	Dihydroxyl group| Glycerol and derivatives	L	Y	NICEATM	Sigma-Aldrich	≥99.5%	1 of 2	No Cat	CO = 0							
401	Glycerol (neat) (2 of 2)	56-81-5	Dihydroxyl group| Glycerol and derivatives	L	Y	ECETOC	Sigma-Aldrich	≥99.5%	2 of 2	No Cat	CO = 0							
402	Glycerol tri-iso-stearate	26492-95-5	N/A	L	N	ECETOC	Unknown	Unknown	1 of 1	No Cat	CO = 0							?
403	Heptyl methacrylate	5459-37-0	Methacrylate	L	Y	ECETOC	Unknown	Unknown	1 of 1	No Cat	CO = 0							?
404	Hexahydrofarnesylacetone	502-69-2	Alkane, branched with tertiary carbon| Isopropyl| Ketone	L	Y	ZEBET	Bedoukian Research, Inc.	98%	1 of 1	No Cat	CO = 0							
405	Igepal CO-210 (Chemical name: Nonyl phenol ethoxylate, branched)	68412-54-4 or 9016-45-9 or 26027-38-3 (84852-15-3 reported in NICEATM ALTOX, which is wrong)	Alcohol| Benzyl| Ether	L	Y	NICEATM	Sigma-Aldrich	Unknown	1 of 1	No Cat	CO = 0							X
406	Igepal CO-990 (Chemical name: Nonyl phenol ethoxylate, branched)	68412-54-4 or 9016-45-9 or 26027-38-3 (84852-15-3 reported in NICEATM ALTOX, which is wrong)	Alcohol| Benzyl| Ether	L	Y	NICEATM	Sigma-Aldrich	Unknown	1 of 1	No Cat	CO = 0							X
407	Iron pentacarbonyl (neat)	13463-40-6	N/A	L	Y	NICEATM	Sigma-Aldrich	≥99.99%	1 of 1	No Cat	CO = 0							
408	iso-Bornyl acetate	125-12-2	Acetoxy| Alkane, branched with tertiary carbon| Bicycloheptane| Bridged-ring carbocycles| Carboxylic acid ester| Cycloalkane	L	Y	ZEBET	Sigma-Aldrich	≥95%	1 of 1	No Cat	CO = 0							
409	iso-Nonylaldehyde	5435-64-3 (35127-50-5 reported in ZEBET, which corresponds to Isononan-1-al)	Aldehyde| Alkane branched with quaternary carbon| Alkane, branched with tertiary carbon| tert-Butyl	L	Y	ZEBET	Sigma-Aldrich	≥95%	1 of 1	No Cat	CO = 0							
410	iso-Octyl acrylate	29590-42-9	Acrylate| Isopropyl	L	Y	ECETOC	Sigma-Aldrich	>90%	1 of 1	No Cat	CO = 0							
411	iso-Octyl thioglycolate	25103-09-7	Carboxylic acid ester| Isopropyl| Thioalcohol	L	Y	ECETOC	Merck	≥98%	1 of 1	No Cat	CO = 0							
412	iso-Propyl bromide	75-26-3	Alkyl halide| Isopropyl	L	Y	ECETOC	Sigma-Aldrich	99%	1 of 1	No Cat	CO = 0							
413	iso-Propyl iso-stearate	68171-33-5	Carboxylic acid ester| Isopropyl	L	N	ECETOC	Unknown	Unknown	1 of 1	No Cat	CO = 0							?
414	iso-Propyl myristate (10%)	110-27-0	Carboxylic acid ester| Isopropyl	L	Y	NICEATM	Sigma-Aldrich	≥98%	1 of 1	No Cat	CO = 0							
415	iso-Propyl myristate (neat)	110-27-0	Carboxylic acid ester| Isopropyl	L	Y	NICEATM	Sigma-Aldrich	≥98%	1 of 1	No Cat	CO = 0							
416	iso-Stearic acid	30399-84-9	Carboxylic acid| Isopropyl	L	Y	ECETOC	Jarchem	Unknown	1 of 1	No Cat	CO = 0							
417	iso-Stearyl alcohol	27458-93-1	Alcohol| Isopropyl	L	Y	ECETOC	Jarchem	100%	1 of 1	No Cat	CO = 0							
418	Kerosine	8008-20-6	N/A	L	Y	NICEATM	Sigma-Aldrich	100%	1 of 1	No Cat	CO = 0							
419	Kronitex 100 (Chemical name: synthetic iso-Propylphenyl phosphate)	Unknown (possibly 66797-44-2)	N/A	L	N	NICEATM	Unknown	Unknown	1 of 1	No Cat	CO = 0							?
420	Kronitex 200 (Chemical name: synthetic iso-Propylphenyl phosphate)	Unknown (possibly 96300-97-9)	N/A	L	N	NICEATM	Unknown	Unknown	1 of 1	No Cat	CO = 0							?
421	Kronitex 200B (Chemical name: tert-Butylphenyl phosphate)	Unknown	N/A	L	N	NICEATM	Unknown	Unknown	1 of 1	No Cat	CO = 0							?
422	Kronitex 300 (Chemical name: iso-Propylphenyl phosphate)	Unknown (possibly 67426-58-8)	N/A	L	N	NICEATM	Unknown	Unknown	1 of 1	No Cat	CO = 0							?
423	Kronitex 50 (Chemical name: synthetic iso-Propylphenyl phosphate)	Unknown (possibly 67426-57-7)	N/A	L	N	NICEATM	Unknown	Unknown	1 of 1	No Cat	CO = 0							?
424	Kronitex CDP (Chemical name: Cresyl diphenyl phosphate)	26444-49-5	Aryl| Phosphate ester	L	Y	NICEATM	Sigma-Aldrich	Unknown	1 of 1	No Cat	CO = 0							
425	Kronitex TXP (Chemical name: Trixylenyl phosphate) (1 of 2)	68952-33-0	Benzyl| Phosphate ester	L	N	NICEATM	Unknown	Unknown	1 of 2	No Cat	CO = 0							?
426	Kronitex TXP (Chemical name: Trixylenyl phosphate) (2 of 2)	68952-33-0	Benzyl| Phosphate ester	L	N	NICEATM	Unknown	Unknown	2 of 2	No Cat	CO = 0							?
427	Methyl cyclopentadiene dimer	26472-00-4	Alkane, branched with tertiary carbon| Allyl| Cycloalkene	L	Y	NICEATM	Sigma-Aldrich	93%	1 of 1	No Cat	CO = 0							
428	Methyl cyclopentane	96-37-7	Alkane, branched with tertiary carbon| Cycloalkane	L	Y	ECETOC	Sigma-Aldrich	97%	1 of 1	No Cat	CO = 0							
429	Methyl triglycol	112-35-6	Alcohol| Ether	L	Y	ZEBET	Sigma-Aldrich	≥97%	1 of 1	No Cat	CO = 0							
430	Methyl trimethyl acetate	598-98-1	Carboxylic acid ester| tert-Butyl	L	Y	ECETOC	Sigma-Aldrich	99%	1 of 1	No Cat	CO = 0							
431	Methyl tetraglycol	23783-42-8 (9004-74-4 reported in ZEBET, which corresponds to Poly(ethylene glycol) methyl ether)	Alcohol| Ether	L	Y	ZEBET	TCI	≥98%	1 of 1	No Cat	CO = 0							
432	m-Methoxthybenzaldehyde	591-31-1	Aldehyde| Aryl| Ether	L	Y	ZEBET	Sigma-Aldrich	≥97%	1 of 1	No Cat	CO = 0							
433	Nitrobenzene	98-95-3	Nitrobenzene	L	Y	NICEATM	Sigma-Aldrich	≥99%	1 of 1	No Cat	CO = 0							

<table>
<tr><th rowspan="3">Study Number</th><th rowspan="3">Test Chemical Name</th><th rowspan="3">CAS RN</th><th rowspan="3">Organic Functional Groups</th><th rowspan="3">Physical Form as tested</th><th rowspan="3">Physical Form Confirmation e.g., by MSDS (Y / N)</th><th rowspan="3">Data Source</th><th rowspan="3">Commercial Source</th><th rowspan="3">Available Purity</th><th rowspan="3">Number of Studies</th><th rowspan="3">UN GHS Classification</th><th colspan="6">Drivers of Classification</th><th rowspan="3">Comments</th><th rowspan="3">Should Not Be Used</th></tr>
<tr><th colspan="2">Severity</th><th colspan="2">Persistence</th><th colspan="2">Specific Observations</th></tr>
<tr><th>Cut-off values</th><th>Number of animals</th><th>Cut-off time</th><th>Number of animals</th><th>CO = 4 or other observations</th><th>Number of animals</th></tr>
<tr><td>434</td><td>Nonyl acrylate</td><td>2664-55-3</td><td>Acrylate</td><td>L</td><td>Y</td><td>ECETOC</td><td>Monomer-Polymer and Dajac Labs</td><td>Unknown</td><td>1 of 1</td><td>No Cat</td><td>CO = 0</td><td></td><td></td><td></td><td></td><td></td><td></td><td></td></tr>
<tr><td>435</td><td>Nonyl methacrylate</td><td>13453-03-7</td><td>Alkane branched with quaternary carbon|
Alkane, branched with tertiary carbon|
Methacrylate|
tert-Butyl</td><td>L</td><td>Y</td><td>ECETOC</td><td>Unknown</td><td>Unknown</td><td>1 of 1</td><td>No Cat</td><td>CO = 0</td><td></td><td></td><td></td><td></td><td></td><td></td><td>?</td></tr>
<tr><td>436</td><td>n-Amyl bromide</td><td>110-53-2</td><td>Alkyl halide</td><td>L</td><td>Y</td><td>ECETOC</td><td>Sigma-Aldrich</td><td>98%</td><td>1 of 1</td><td>No Cat</td><td>CO = 0</td><td></td><td></td><td></td><td></td><td></td><td></td><td></td></tr>
<tr><td>437</td><td>n-Hexane</td><td>110-54-3</td><td>N/A</td><td>L</td><td>Y</td><td>LNS</td><td>Sigma-Aldrich</td><td>≥97%</td><td>1 of 1</td><td>No Cat</td><td>CO = 0</td><td></td><td></td><td></td><td></td><td></td><td></td><td></td></tr>
<tr><td>438</td><td>n-Hexyl bromide</td><td>111-25-1</td><td>Alkyl halide</td><td>L</td><td>Y</td><td>ECETOC</td><td>Sigma-Aldrich</td><td>98%</td><td>1 of 1</td><td>No Cat</td><td>CO = 0</td><td></td><td></td><td></td><td></td><td></td><td></td><td></td></tr>
<tr><td>439</td><td>Octadecyl isocyanate</td><td>112-96-9</td><td>Isocyanate</td><td>L</td><td>Y</td><td>NICEATM</td><td>Sigma-Aldrich</td><td>98%</td><td>1 of 1</td><td>No Cat</td><td>CO = 0</td><td></td><td></td><td></td><td></td><td></td><td></td><td></td></tr>
<tr><td>440</td><td>Organofunctional silane 53-98 (neat)</td><td>26115-70-8</td><td>N/A</td><td>L</td><td>Y</td><td>NICEATM</td><td>SCBT</td><td>>98%</td><td>1 of 1</td><td>No Cat</td><td>CO = 0</td><td></td><td></td><td></td><td></td><td></td><td></td><td></td></tr>
<tr><td>441</td><td>n-Octyl bromide</td><td>111-83-1</td><td>Alkyl halide</td><td>L</td><td>Y</td><td>ECETOC</td><td>Sigma-Aldrich</td><td>99%</td><td>1 of 1</td><td>No Cat</td><td>CO = 0</td><td></td><td></td><td></td><td></td><td></td><td></td><td></td></tr>
<tr><td>442</td><td>Pentaerythritol, propoxylated</td><td>9051-49-4</td><td>Alcohol|
Alkane branched with quaternary carbon|
Dihydroxyl group|
Ether</td><td>L</td><td>Y</td><td>NICEATM</td><td>Sigma-Aldrich</td><td>Unknown</td><td>1 of 1</td><td>No Cat</td><td>CO = 0</td><td></td><td></td><td></td><td></td><td></td><td></td><td></td></tr>
<tr><td>443</td><td>Perfluoro-n-hexane (1 of 2)</td><td>355-42-0</td><td>Perflourocarbons derivatives</td><td>L</td><td>Y</td><td>NICEATM</td><td>Sigma-Aldrich</td><td>99%</td><td>1 of 2</td><td>No Cat</td><td>CO = 0</td><td></td><td></td><td></td><td></td><td></td><td></td><td></td></tr>
<tr><td>444</td><td>Perfluoro-n-hexane (2 of 2)</td><td>355-42-0</td><td>Perflourocarbons derivatives</td><td>L</td><td>Y</td><td>NICEATM</td><td>Sigma-Aldrich</td><td>99%</td><td>2 of 2</td><td>No Cat</td><td>CO = 0</td><td></td><td></td><td></td><td></td><td></td><td></td><td></td></tr>
<tr><td>445</td><td>Petroleum ether</td><td>8032-32-4</td><td>N/A</td><td>L</td><td>Y</td><td>LNS</td><td>Sigma-Aldrich</td><td>100%</td><td>1 of 1</td><td>No Cat</td><td>CO = 0</td><td></td><td></td><td></td><td></td><td></td><td></td><td></td></tr>
<tr><td>446</td><td>Phosphoric acid, tributyl ester (Tributyl phosphate reported in NICEATM ALTTOX) (2 of 2)</td><td>126-73-8</td><td>Alkoxy|
Phosphate ester</td><td>L</td><td>Y</td><td>NICEATM</td><td>Sigma-Aldrich</td><td>≥99%</td><td>2 of 2</td><td>No Cat</td><td>CO = 0</td><td></td><td></td><td></td><td></td><td></td><td></td><td>X</td></tr>
<tr><td>447</td><td>Polyethylene glycol 400 (10%)</td><td>25322-68-3</td><td>Dihydroxyl group</td><td>L</td><td>Y</td><td>NICEATM</td><td>Sigma-Aldrich</td><td>Unknown</td><td>1 of 1</td><td>No Cat</td><td>CO = 0</td><td></td><td></td><td></td><td></td><td></td><td></td><td></td></tr>
<tr><td>448</td><td>Polyethylene glycol 400 (neat) (1 of 2)</td><td>25322-68-3</td><td>Dihydroxyl group</td><td>L</td><td>Y</td><td>ECETOC</td><td>Sigma-Aldrich</td><td>Unknown</td><td>1 of 2</td><td>No Cat</td><td>CO = 0</td><td></td><td></td><td></td><td></td><td></td><td></td><td></td></tr>
<tr><td>449</td><td>Polyethylene glycol 400 (neat) (2 of 2)</td><td>25322-68-3</td><td>Dihydroxyl group</td><td>L</td><td>Y</td><td>NICEATM</td><td>Sigma-Aldrich</td><td>Unknown</td><td>2 of 2</td><td>No Cat</td><td>CO = 0</td><td></td><td></td><td></td><td></td><td></td><td></td><td></td></tr>
<tr><td>450</td><td>Polyethylene glycol 600</td><td>25322-68-3</td><td>Dihydroxyl group</td><td>L</td><td>Y</td><td>ECETOC</td><td>Fisher Scientific</td><td>>95%</td><td>1 of 1</td><td>No Cat</td><td>CO = 0</td><td></td><td></td><td></td><td></td><td></td><td></td><td></td></tr>
<tr><td>451</td><td>Polyethylene glycol dimethylether</td><td>24991-55-7</td><td>Ether</td><td>L</td><td>Y</td><td>ZEBET</td><td>Sigma-Aldrich</td><td>Unknown</td><td>1 of 1</td><td>No Cat</td><td>CO = 0</td><td></td><td></td><td></td><td></td><td></td><td></td><td></td></tr>
<tr><td>452</td><td>Polyethylene glycol monolaurate</td><td>9004-81-3</td><td>Alcohol|
Carboxylic acid ester</td><td>L</td><td>Y</td><td>NICEATM</td><td>Sigma-Aldrich</td><td>Unknown</td><td>1 of 1</td><td>No Cat</td><td>CO = 0</td><td></td><td></td><td></td><td></td><td></td><td></td><td></td></tr>
<tr><td>453</td><td>Polyglyceryl-3-diisooctadecanoate</td><td>63705-03-3</td><td>Alcohol|
Carboxylic acid ester|
Isopropyl</td><td>L</td><td>Y</td><td>EURL ECVAM</td><td>Proprietary</td><td>100%</td><td>1 of 1</td><td>No Cat</td><td>CO = 0</td><td></td><td></td><td></td><td></td><td></td><td></td><td>?</td></tr>
<tr><td>454</td><td>Polyol 355 UCB</td><td>25791-96-2</td><td>Alcohol|
Ether|
Glycerol and derivatives</td><td>L</td><td>Y</td><td>NICEATM</td><td>Dow</td><td>>99%</td><td>1 of 1</td><td>No Cat</td><td>CO = 0</td><td></td><td></td><td></td><td></td><td></td><td></td><td></td></tr>
<tr><td>455</td><td>Polyol XZ 95435.00</td><td>52625-13-5</td><td>Alcohol|
Dihydroxyl group|
Ether</td><td>L</td><td>Y</td><td>NICEATM</td><td>Dow</td><td>>99%</td><td>1 of 1</td><td>No Cat</td><td>CO = 0</td><td></td><td></td><td></td><td></td><td></td><td></td><td></td></tr>
<tr><td>456</td><td>Propylene glycol</td><td>57-55-6</td><td>Dihydroxyl group</td><td>L</td><td>Y</td><td>ECETOC</td><td>Sigma-Aldrich</td><td>≥99.5%</td><td>1 of 1</td><td>No Cat</td><td>CO = 0</td><td></td><td></td><td></td><td></td><td></td><td></td><td></td></tr>
<tr><td>457</td><td>p-Chlorobenzotrifluoride</td><td>98-56-6</td><td>Alkyl halide|
Aryl halide</td><td>L</td><td>Y</td><td>NICEATM</td><td>Sigma-Aldrich</td><td>98%</td><td>1 of 1</td><td>No Cat</td><td>CO = 0</td><td></td><td></td><td></td><td></td><td></td><td></td><td></td></tr>
<tr><td>458</td><td>p-Methyl thiobenzaldehyde</td><td>3446-89-7</td><td>Aldehyde|
Aryl|
Sulfide</td><td>L</td><td>Y</td><td>ECETOC</td><td>Sigma-Aldrich</td><td>95%</td><td>1 of 1</td><td>No Cat</td><td>CO = 0</td><td></td><td></td><td></td><td></td><td></td><td></td><td></td></tr>
<tr><td>459</td><td>p-tert-Butylbenzoic acid, triethanolamine salt (10%)</td><td>59993-86-1 (98-73-7 reported in NICEATM ALTTOX, which corresponds to 4-tert-Butylbenzoic acid)</td><td>Alcohol|
Aliphatic Amine, tertiary|
Aryl|
Carboxylic acid|
Isopropyl</td><td>L</td><td>Y</td><td>NICEATM</td><td>Unknown</td><td>Unknown</td><td>1 of 1</td><td>No Cat</td><td>CO = 0</td><td></td><td></td><td></td><td></td><td></td><td></td><td>?</td></tr>
<tr><td>460</td><td>Ricinoleic acid tin salt</td><td>71828-07-4</td><td>Dihydroxyl group</td><td>L</td><td>Y</td><td>EURL ECVAM</td><td>Proprietary</td><td>100%</td><td>1 of 1</td><td>No Cat</td><td>CO = 0</td><td></td><td></td><td></td><td></td><td></td><td></td><td>?</td></tr>
<tr><td>461</td><td>Silan 108 (Chemical name: Trimethoxyoctylsilane)</td><td>3069-40-7</td><td>AlkoxySilane</td><td>L</td><td>Y</td><td>ZEBET</td><td>Sigma-Aldrich</td><td>96%</td><td>1 of 1</td><td>No Cat</td><td>CO = 0</td><td></td><td></td><td></td><td></td><td></td><td></td><td></td></tr>
<tr><td>462</td><td>Silicone Y-4081 (Chemical name: Hexamethyldisiloxane) (neat)</td><td>107-46-0</td><td>Disiloxane</td><td>L</td><td>Y</td><td>NICEATM</td><td>Sigma-Aldrich</td><td>≥98.5%</td><td>1 of 1</td><td>No Cat</td><td>CO = 0</td><td></td><td></td><td></td><td></td><td></td><td></td><td></td></tr>
<tr><td>463</td><td>Steareth-10 allyl ether/acrylates copolymer (~40%, aqueous)</td><td>109292-17-3</td><td>Acrylate|
Alkoxy|
Allyl|
Carboxylic acid|
Ether</td><td>L</td><td>Y</td><td>EURL ECVAM</td><td>Proprietary</td><td>~40%, aqueous</td><td>1 of 1</td><td>No Cat</td><td>CO = 0</td><td></td><td></td><td></td><td></td><td></td><td></td><td>?</td></tr>
<tr><td>464</td><td>Thiodiglycol</td><td>111-48-8</td><td>Alcohol|
Sulfide</td><td>L</td><td>Y</td><td>ECETOC</td><td>Sigma-Aldrich</td><td>≥99%</td><td>1 of 1</td><td>No Cat</td><td>CO = 0</td><td></td><td></td><td></td><td></td><td></td><td></td><td></td></tr>
<tr><td>465</td><td>Tricresyl phosphate (Kronitex TCP reported in NICEATM ALTTOX) (1 of 2)</td><td>1330-78-5</td><td>Aryl|
Phosphate ester</td><td>L</td><td>Y</td><td>NICEATM</td><td>Sigma-Aldrich</td><td>90%</td><td>1 of 2</td><td>No Cat</td><td>CO = 0</td><td></td><td></td><td></td><td></td><td></td><td></td><td></td></tr>
<tr><td>466</td><td>Tricresyl phosphate (2 of 2)</td><td>1330-78-5</td><td>Aryl|
Phosphate ester</td><td>L</td><td>Y</td><td>NICEATM</td><td>Sigma-Aldrich</td><td>90%</td><td>2 of 2</td><td>No Cat</td><td>CO = 0</td><td></td><td></td><td></td><td></td><td></td><td></td><td></td></tr>
<tr><td>467</td><td>Triethanolamine (10%)</td><td>102-71-6</td><td>Alcohol|
Aliphatic Amine, tertiary</td><td>L</td><td>Y</td><td>NICEATM</td><td>Sigma-Aldrich</td><td>≥99.5%</td><td>1 of 1</td><td>No Cat</td><td>CO = 0</td><td></td><td></td><td></td><td></td><td></td><td></td><td></td></tr>
<tr><td>468</td><td>Triethanolamine (neat) (2 of 2)</td><td>102-71-6</td><td>Alcohol|
Aliphatic Amine, tertiary</td><td>L</td><td>Y</td><td>LNS</td><td>Sigma-Aldrich</td><td>≥99.5%</td><td>2 of 2</td><td>No Cat</td><td>CO = 0</td><td></td><td></td><td></td><td></td><td></td><td></td><td></td></tr>
<tr><td>469</td><td>Triethoxyoctylsilane (neat)</td><td>2943-75-1</td><td>Alkoxy|
AlkoxySilane</td><td>L</td><td>Y</td><td>NICEATM</td><td>Sigma-Aldrich</td><td>≥97.5%</td><td>1 of 1</td><td>No Cat</td><td>CO = 0</td><td></td><td></td><td></td><td></td><td></td><td></td><td></td></tr>
<tr><td>470</td><td>Triethylene glycol</td><td>112-27-6</td><td>Alcohol|
Ether</td><td>L</td><td>Y</td><td>NICEATM</td><td>Sigma-Aldrich</td><td>≥99%</td><td>1 of 1</td><td>No Cat</td><td>CO = 0</td><td></td><td></td><td></td><td></td><td></td><td></td><td></td></tr>
<tr><td>471</td><td>Trifluoroethyl methacrylate</td><td>352-87-4 (392-68-7 reported in ECETOC, which corresponds to Ethyl trifluoroacrylate)</td><td>Alkyl halide|
Methacrylate</td><td>L</td><td>Y</td><td>ECETOC</td><td>Sigma-Aldrich</td><td>99%</td><td>1 of 1</td><td>No Cat</td><td>CO = 0</td><td></td><td></td><td></td><td></td><td></td><td></td><td></td></tr>
<tr><td>472</td><td>Triisodecyl phosphite (neat)</td><td>25448-25-3</td><td>N/A</td><td>L</td><td>Y</td><td>NICEATM</td><td>Sigma-Aldrich</td><td>Unknown</td><td>1 of 1</td><td>No Cat</td><td>CO = 0</td><td></td><td></td><td></td><td></td><td></td><td></td><td></td></tr>
<tr><td>473</td><td>Triisooctylamine</td><td>25549-16-0</td><td>Aliphatic Amine, tertiary|
Isopropyl</td><td>L</td><td>Y</td><td>ZEBET</td><td>Merck</td><td>≥95%</td><td>1 of 1</td><td>No Cat</td><td>CO = 0</td><td></td><td></td><td></td><td></td><td></td><td></td><td></td></tr>
<tr><td>474</td><td>Triphenyl phosphite (neat)</td><td>101-02-0</td><td>Aryl|
Phosphite ester</td><td>L</td><td>Y</td><td>NICEATM</td><td>Sigma-Aldrich</td><td>97%</td><td>1 of 1</td><td>No Cat</td><td>CO = 0</td><td></td><td></td><td></td><td></td><td></td><td></td><td></td></tr>
<tr><td>475</td><td>Triton X-100 (1%)</td><td>9002-93-1</td><td>Alcohol|
Alkane branched with quaternary carbon|
Aryl|
Ether|
tert-Butyl</td><td>L</td><td>Y</td><td>ECETOC</td><td>Sigma-Aldrich</td><td>laboratory grade</td><td>1 of 1</td><td>No Cat</td><td>CO = 0</td><td></td><td></td><td></td><td></td><td></td><td></td><td></td></tr>
<tr><td>476</td><td>Tween 20 (1 of 2)</td><td>9005-64-5</td><td>Acetal|
Alcohol|
Allyl|
Carboxylic acid ester|
Ether|
Oxolane|
Saturated heterocyclic fragment</td><td>L</td><td>Y</td><td>ECETOC</td><td>Sigma-Aldrich</td><td>Unknown</td><td>1 of 2</td><td>No Cat</td><td>CO = 0</td><td></td><td></td><td></td><td></td><td></td><td></td><td></td></tr>
<tr><td>477</td><td>Tween 20 (2 of 2)</td><td>9005-64-5</td><td>Acetal|
Alcohol|
Allyl|
Carboxylic acid ester|
Ether|
Oxolane|
Saturated heterocyclic fragment</td><td>L</td><td>Y</td><td>NICEATM</td><td>Sigma-Aldrich</td><td>Unknown</td><td>2 of 2</td><td>No Cat</td><td>CO = 0</td><td></td><td></td><td></td><td></td><td></td><td></td><td></td></tr>
<tr><td>478</td><td>Tween 80 (10%)</td><td>9005-65-6</td><td>Acetal|
Alcohol|
Allyl|
Carboxylic acid ester|
Ether|
Oxolane|
Saturated heterocyclic fragment</td><td>L</td><td>Y</td><td>NICEATM</td><td>Sigma-Aldrich</td><td>Unknown</td><td>1 of 1</td><td>No Cat</td><td>CO = 0</td><td></td><td></td><td></td><td></td><td></td><td></td><td></td></tr>
<tr><td>479</td><td>Tween 80 (neat)</td><td>9005-65-6</td><td>Acetal|
Alcohol|
Allyl|
Carboxylic acid ester|
Ether|
Oxolane|
Saturated heterocyclic fragment</td><td>L</td><td>Y</td><td>NICEATM</td><td>Sigma-Aldrich</td><td>Unknown</td><td>1 of 1</td><td>No Cat</td><td>CO = 0</td><td></td><td></td><td></td><td></td><td></td><td></td><td></td></tr>
<tr><td>480</td><td>Vinyl 2-ethylhexanoate (neat)</td><td>94-04-2</td><td>Alkene|
Carboxylic acid ester</td><td>L</td><td>Y</td><td>NICEATM</td><td>TCI</td><td>>98%</td><td>1 of 1</td><td>No Cat</td><td>CO = 0</td><td></td><td></td><td></td><td></td><td></td><td></td><td></td></tr>
<tr><td>481</td><td>Vinyl tris (beta-methoxyethoxy) silane</td><td>1067-53-4</td><td>N/A</td><td>L</td><td>Y</td><td>NICEATM</td><td>Sigma-Aldrich</td><td>98%</td><td>1 of 1</td><td>No Cat</td><td>CO = 0</td><td></td><td></td><td></td><td></td><td></td><td></td><td></td></tr>
<tr><td>482</td><td>Vinyltrimethoxy silane</td><td>2768-02-7</td><td>N/A</td><td>L</td><td>Y</td><td>NICEATM</td><td>Sigma-Aldrich</td><td>98%</td><td>1 of 1</td><td>No Cat</td><td>CO = 0</td><td></td><td></td><td></td><td></td><td></td><td></td><td></td></tr>
</table>

Study Number	Test Chemical Name	CAS RN	Organic Functional Groups	Physical Form as tested	Physical Form Confirmation e.g., by MSDS (Y / N)	Data Source	Commercial Source	Available Purity	Number of Studies	UN GHS Classification	Drivers of Classification						Comments	Should Not Be Used
											Severity		Persistence		Specific Observations			
											Cut-off values	Number of animals	Cut-off time	Number of animals	CO = 4 or other observations	Number of animals		
483	Xylene (2 of 2)	1330-20-7	Imide	L	Y	ECETOC	Sigma-Aldrich	≥98.5% (Xylene mixture of isomers)	2 of 2	No Cat	CO = 0							
484	Igepon AC-78 (0.5%) (Chemical name: Sodium cocoyl isethionate)	58969-27-0 (original CAS # deleted and replaced by 61789-32-0)	Carboxylic acid ester| Sulfonic acid	L (tested in solvent, neat chemical unknown)	Y	ECETOC	Unknown	Unknown	1 of 1	No Cat	CO = 0							?
485	Acid Red 92 (Chemical name: 3,4,5,6-Tetrachloro-2-(1,4,5,8-tetrabromo-6-hydroxy-3-oxoxanthen-9-yl)-benzoic acid) (1%)	18472-87-2	Aromatic perhalogencarbons| Aryl halide| Fused carbocyclic aromatic| Fused saturated heterocycles| Heterocyclic spiro rings| Isobenzofuran| Lactone| Phenol| Xanthene	L (tested in solvent, available as S)	Y	NICEATM	SCBT	>95%	1 of 1	No Cat	CO = 0							
486	Benzyl alcohol (1%)	100-51-6	Alcohol| Benzyl	L (tested in solvent, available as S)	Y	NICEATM	Sigma-Aldrich	99.80%	1 of 1	No Cat	CO = 0							
487	Brij-35 (10%) (Chemical name: Polyoxyethylene 23 lauryl ether; also known as Brij L23)	9002-92-0	Alcohol| Alkoxy| Ether	L (tested in solvent, available as S)	Y	LNS	Sigma-Aldrich	100%	1 of 1	No Cat	CO = 0							
488	Calcium thioglycolate (10%)	814-77-1	N/A	L (tested in solvent, available as S)	Y	NICEATM	Sigma-Aldrich	≥98%	1 of 1	No Cat	CO = 0							
489	Cetylpyridinium chloride (0.1%)	6004-24-6	Ammonium salt| Aryl| Pyridine	L (tested in solvent, available as S)	Y	NICEATM	Sigma-Aldrich	>99%	1 of 1	No Cat	CO = 0							
490	Ivory soap solution (5%, aqueous)	Unknown	N/A	L (tested in solvent, available as S)	Y	NICEATM	Unknown	Unknown	1 of 1	No Cat	CO = 0							?
491	Myrj-45 (10%) (Chemical name: Polyoxyethylene 8-stearate)	9004-99-3	Alcohol| Carboxylic acid ester	L (tested in solvent, available as S)	Y	LNS	Croda	Unknown	1 of 1	No Cat	CO = 0							
492	Phosphoric acid compound with 4-[(2,6-dichlorophenyl)(4-imino-3,5-dimethyl-2,5-cyclohexadien-1-ylidene)methyl]-2,6-dimethylaniline (1:1) (2% (w/v), aqueous)	74578-10-2	Alkene| Allyl| Aryl| Aryl halide| Ketimine| Precursors quinoid compounds	L (tested in solvent, available as S)	Y	Cosmetics Europe	Proprietary	≥95%	1 of 1	No Cat	CO = 0							?
493	Sodium chloride isotonic solution	7647-14-5	N/A	L (tested in solvent, available as S)	Y	NICEATM	Sigma-Aldrich	≥99.5%	1 of 1	No Cat	CO = 0							
494	Sodium salicylate (10%)	54-21-7	Carboxylic acid| Phenol	L (tested in solvent, available as S)	Y	NICEATM	Sigma-Aldrich	≥99.5%	1 of 1	No Cat	CO = 0							
495	Theophylline sodium (0.1%)	3485-82-3	Dihydropurinedione| Urea derivatives	L (tested in solvent, available as S)	Y	NICEATM	Unknown	Unknown	1 of 1	No Cat	CO = 0						NICEATM ALTTOX database indicates 0.1 in the concentration field; Not clear if this is percent concentration or dose volume inserted by mistake in the concentration field	?
496	Trichloroacetic acid (3%)	76-03-9	Alkyl halide| Carboxylic acid	L (tested in solvent, available as S)	Y	ECETOC	Sigma-Aldrich	>99%	1 of 1	No Cat	CO = 0							
497	Triethanolamine orthovanadate (3%)	13476-99-8	Diketone	L (tested in solvent, available as S)	Y	NICEATM	Sigma-Aldrich	97%	1 of 1	No Cat	CO = 0							
498	2-(7-Ethyl-1H-indol-3-yl)-ethanol	41340-36-7	Alcohol| Allyl| Benzyl| Indole/ Isoindole	S (waxy)	Y	EURL ECVAM	Chemos GmbH	99.2%	1 of 1	No Cat	CO = 0							
499	Perfecto petrolatum	Unknown	N/A	S (waxy)	N	NICEATM	Unknown	Unknown	1 of 1	No Cat	CO = 0							?
500	Polyethylene glycol (PEG-120) methyl glucose dioleate	86893-19-8	Acetal| Alcohol| Allyl| Carboxylic acid ester| Ether| Ether (cyclic)| Heterocyclic fragment	S (waxy)	Y	EURL ECVAM	Proprietary	100%	1 of 1	No Cat	CO = 0							?
501	Polyethylene glycol (PEG-40) hydrogenated castor oil (Polyoxyethylene hydrogenated castor oil reported in NICEATM ALTTOX) (1 of 2)	61788-85-0	Acylal| Alcohol| Allyl| Ether	S (waxy)	Y	NICEATM	Sigma-Aldrich	100%	1 of 2	No Cat	CO = 0							
502	Polyethylene glycol (PEG-40) hydrogenated castor oil (2 of 2)	61788-85-0	Acylal| Alcohol| Allyl| Ether	S (waxy)	Y	EURL ECVAM	Sigma-Aldrich	100%	2 of 2	No Cat	CO = 0							
503	[3-Chloro-4-[(3-fluorobenzyl)oxy]phenyl](6-iodoquinazolin-4-yl)amine	231278-20-9	Aromatic amine| Aryl| Aryl halide| Benzyl| Ether| Quinazoline	S	Y	EURL ECVAM	Chemos GmbH	99%	1 of 1	No Cat	CO = 0							
504	1,5-Di(2,4-dimethylphenyl)-3-methyl-1,3,5-triazapenta-1,4-diene	33089-61-1	Amidine| Aryl	S	Y	EURL ECVAM	Chemos GmbH	98.1%	1 of 1	No Cat	CO = 0							
505	1,6,7,12-Tetrachloro-3,4,9,10-tetracarbonic acid anhydride	156028-26-1	N/A	S	Y	ZEBET	SCBT	98%	1 of 1	No Cat	CO = 0							
506	1H-Indole-2,3-dione	91-56-5	Cycloketone| Indoline| Lactams	S	Y	NICEATM	Sigma-Aldrich	≥99%	1 of 1	No Cat	CO = 0							
507	1-(4-Chlorophenyl)-3-(3,4-dichlorophenyl) urea	101-20-2	Aromatic heterocyclic halide| Aryl halide| Urea derivatives	S	Y	EURL ECVAM	Sigma-Aldrich	99%	1 of 1	No Cat	CO = 0							
508	1-(4-Phenyl-phenoxy)-1-(1,2,4-triazole-1)-3,3-dimethylbutan-2-ol	55179-31-2	Alcohol| Alkane, branched with quaternary carbon| Biphenyl| Ether| Hydrazone	S	Y	NICEATM	SCBT	100%	1 of 1	No Cat	CO = 0							
509	1-(9H-Carbazol-4-yloxy)-3-[[2-(2-methoxyphenoxy)ethyl]amino]propan-2-ol	72956-09-3	Alcohol| Aliphatic Amine, secondary| Aryl| Carbazole| Ether	S	Y	EURL ECVAM	Chemos GmbH	99.8%	1 of 1	No Cat	CO = 0							
510	2',6',8-Trifluoro-5-methoxy[1,2,4]triazolo[1,5-c]pyrimidine-2-sulphonanilide	145701-23-1	Amidine| Aromatic heterocyclic halide| Aryl halide| Ether| Sulfonamide	S	Y	EURL ECVAM	Chemos GmbH	98.9%	1 of 1	No Cat	CO = 0							
511	2,2'-[[3-Methyl-4-[(4-nitrophenyl)azo]phenyl]imino]bis-ethanol	3179-89-3	Alcohol| Aromatic amine| Aryl| Azo| Nitrobenzene	S	Y	EURL ECVAM	Chemos GmbH	100%	1 of 1	No Cat	CO = 0							
512	2,2'-[[4-[(2-Methoxyethyl)amino]-3-nitrophenyl]imino]bis-ethanol	23920-15-2	Alcohol| Aromatic amine| Ether| Nitrobenzene	S	Y	EURL ECVAM	Chemos GmbH	99.3%	1 of 1	No Cat	CO = 0							
513	2,2'-[6-(4-Methoxyphenyl)-1,3,5-triazine-2,4-diyl]bis[5-[(2-ethylhexyl)oxy]-phenol]	187393-00-6	Alkoxy| Aryl| Ether| Phenol| Triazine	S	Y	EURL ECVAM	Sigma-Aldrich	98%	1 of 1	No Cat	CO = 0							
514	2,2'-Methylene-bis-(6-(2H-benzotriazol-2-yl)-4-(1,1,3,3-tetramethylbutyl)phenol)	103597-45-1	Alkane branched with quaternary carbon| Aryl| Benzyl| Fused carbocyclic aromatic| Fused saturated heterocycles| Phenol| tert-Butyl	S	Y	EURL ECVAM	Sigma-Aldrich	99%	1 of 1	No Cat	CO = 0							
515	2,3-Dimethyl-2,3-dinitrobutane	3964-18-9 (3964-18-4 reported in NICEATM ALTTOX, which is wrong)	Nitro aliphatic	S	Y	NICEATM	Sigma-Aldrich	98%	1 of 1	No Cat	CO = 0							
516	2,5,6-Triamino-4-pyrimidinol sulphate	1603-02-7	Aryl| Pyrimidine| Sulfate	S	Y	EURL ECVAM	ChemReagents.com	94%	1 of 1	No Cat	CO = 0							
517	2,6-Dihydroxy-3,4-dimethylpyridine	84540-47-6	Heterocyclic Phenol	S	Y	EURL ECVAM	Chemos GmbH	98.5%	1 of 1	No Cat	CO = 0							

Cosmetics Europe
Draize eye test Reference Database (DRD)
Barroso *et al.* , 2016

Study Number	Test Chemical Name	CAS RN	Organic Functional Groups	Physical Form as tested	Physical Form Confirmation e.g., by MSDS (Y / N)	Data Source	Commercial Source	Available Purity	Number of Studies	UN GHS Classification	Drivers of Classification: Severity: Cut-off values	Drivers of Classification: Severity: Number of animals	Drivers of Classification: Persistence: Cut-off time	Drivers of Classification: Persistence: Number of animals	Drivers of Classification: Specific Observations: CO = 4 or other observations	Drivers of Classification: Specific Observations: Number of animals	Comments	Should Not Be Used
518	2-(4-Oxopentyl)-1H-isoindole-1,3 (2H)-dione	3197-25-9	Aryl| Fused carbocyclic aromatic| Fused saturated heterocycles| Imide| Ketone	S	Y	NICEATM	SCBT	≥95%	1 of 1	No Cat	CO = 0							
519	2-(Diphenylacetyl)-1,3-indandione	82-66-6	Aryl| Diketone| Indandione	S	Y	EURL ECVAM	Chemos GmbH	95.2%	1 of 1	No Cat	CO = 0							
520	2-(n-Dodecylthio)ethanol	1462-55-1	Alcohol| Sulfide	S	Y	ECETOC	Unknown	Unknown	1 of 1	No Cat	CO = 0							?
521	2-Aminophenol	95-55-6	Precursors quinoid compounds	S	Y	LNS	Sigma-Aldrich	99%	1 of 1	No Cat	CO = 0							
522	2-Anilino-4,6-dimethylpyrimidine	53112-28-0	Aromatic amine| Aryl| Pyrimidine	S	Y	EURL ECVAM	Chemos GmbH	98.2%	1 of 1	No Cat	CO = 0							
523	2-Hydroxyethyliminodisodium acetate	135-37-5	Alcohol| Aliphatic Amine, tertiary| Carboxylic acid	S	Y	ZEBET	American Custom Chemicals Corporation	>95%	1 of 1	No Cat	CO = 0							
524	2-Mercaptopyrimidine	1450-85-7	Heterocyclic Thiophenol	S	Y	LNS	Sigma-Aldrich	98%	1 of 1	No Cat	CO = 0							
525	3,4-Dimethoxybenzaldehyde	120-14-9	Aldehyde| Aryl| Ether	S	Y	NICEATM	Sigma-Aldrich	99%	1 of 1	No Cat	CO = 0							
526	3,5-Dihydroxyacetophenone	51863-60-6 (35086-59-0 reported in ZEBET, which corresponds to 3',5'-Diacetoxyacetophenone)	Ketone| Phenol	S	Y	ZEBET	Sigma-Aldrich	97%	1 of 1	No Cat	CO = 0							
527	3H-Pyrazole-3-one, 2(4-aminophenyl),4-dihydro-5-(1-pyrrolindinyl)	30707-77-8	Amidine| Aniline| Pyrazolone| Pyrrolidine| Saturated heterocyclic amine| Saturated heterocyclic fragment	S	Y	NICEATM	Unknown	Unknown	1 of 1	No Cat	CO = 0							?
528	3-((Benzylthio)methyl)-6-chloro-1,1-dioxide	91-33-8	Aryl| Aryl halide| Benzyl| Sulfide| Sulfonamide| Unsaturated heterocyclic fragment	S	Y	NICEATM	SCBT	>98%	1 of 1	No Cat	CO = 0							
529	3-(2-Chloro-thiazol-5-ylmethyl)-5-methyl[1,3,5]oxadiazinan-4-ylidene-N-nitroamine	153719-23-4	Allyl| Aryl halide| Guanidine| Saturated heterocyclic fragment	S	Y	EURL ECVAM	Chemos GmbH	96.3%	1 of 1	No Cat	CO = 0							
530	3-Chloro-4-fluoronitrobenzene	350-30-1 (446-35-5 reported in ECETOC, which corresponds to 2,4-Difluoronitrobenzene)	Aryl halide| Nitrobenzene	S	Y	ECETOC	Sigma-Aldrich	98%	1 of 1	No Cat	CO = 0							
531	4'-Aminoazobenzene-4-sulphonic acid	104-23-4	Aniline| Aryl| Azo| Sulfonic acid	S	Y	ZEBET	IS Chemical Technology	98%	1 of 1	No Cat	CO = 0							
532	4,4'-Methylene bis-(2,6-di-tert-butylphenol)	118-82-1 (118-82-3 reported in ECETOC, which is wrong)	Benzyl| Phenol| tert-Butyl	S	Y	ECETOC	Sigma-Aldrich	98%	1 of 1	No Cat	CO = 0							
533	4,4'-Sulfonylbisbenezenamide	80-08-0	Dianilines| Sulfone	S	Y	NICEATM	Sigma-Aldrich	97%	1 of 1	No Cat	CO = 0							
534	4,4-Dimethyl-3-oxopentanitrile	59997-51-2	Ketone| Nitrile| tert-Butyl	S	Y	ZEBET	Sigma-Aldrich	99%	1 of 1	No Cat	CO = 0							
535	4-Chloro-4-nitrodiphenylether	1836-74-4	Aryl| Aryl halide| Ether| Nitrobenzene	S	Y	ZEBET	Sigma-Aldrich	Unknown	1 of 1	No Cat	CO = 0							
536	Abamectin	71751-41-2	Acetal| Alkane, branched with tertiary carbon| Allyl| Cycloalkene| Dihydroxyl group| Ether| Ether, cyclic| Heterocyclic spiro rings| Lactone| Saturated heterocyclic fragment| Unsaturated heterocyclic fragment	S	Y	EURL ECVAM	Sigma-Aldrich	analytical standard	1 of 1	No Cat	CO = 0							
537	Aluminium hydroxide	21645-51-2	N/A	S	Y	LNS	Sigma-Aldrich	reagent grade	1 of 1	No Cat	CO = 0							
538	Ambuphylline	5634-34-4	Alcohol| Aliphatic Amine, primary| Dihydropurinedione| Urea derivatives	S	Y	ZEBET	Unknown	Unknown	1 of 1	No Cat	CO = 0							?
539	Amicarbazone	129909-90-6	Amidine| Carboxamide| Hydrazine| Hydrazone| Imide| Lactam	S	Y	EURL ECVAM	Sigma-Aldrich	analytical standard	1 of 1	No Cat	CO = 0							
540	Anthracene	120-12-7	Anthracene| Fused carbocyclic aromatic	S	Y	LNS	Sigma-Aldrich	99%	1 of 1	No Cat	CO = 0							
541	Benzoflex S-312 (Chemical name: Neopentyl glycol dibenzoate)	4196-89-8	Alkane branched with quaternary carbon| Aryl| Carboxylic acid ester	S	Y	NICEATM	TCI	>98%	1 of 1	No Cat	CO = 0							
542	beta-(4-Chlorophenoxy)-alpha-(1,1-dimethylethyl)-1H-1,2,4-triazole-1-ethanol	55219-65-3	Alcohol| Alkane branched with quaternary carbon| Aryl| Aryl halide| Ether| tert-Butyl| Triazole	S	Y	NICEATM	SCBT	≥96%	1 of 1	No Cat	CO = 0							
543	Brodifacoum	56073-10-0	Aryl| Aryl halide| Biphenyl| Chromene| Enol| Fused carbocyclic aromatic| Lactone| Tetralin	S	Y	NICEATM	Sigma-Aldrich	analytical standard	1 of 1	No Cat	CO = 0							
544	Caffeine sodium benzoate	8000-95-1	N/A	S	Y	ZEBET	Sigma-Aldrich	Unknown	1 of 1	No Cat	CO = 0							
545	Caffeine sodium salicylate	8002-85-5	N/A	S	Y	ZEBET	Carbone Scientific	98%	1 of 1	No Cat	CO = 0							
546	Cellulose,2-(2-hydroxy-3-(trimethylammonium)propoxy)ethyl ether chloride(91%)	68610-92-4	Alcohol| Ammonium salt| Ether	S	Y	EURL ECVAM	Sigma-Aldrich	91%	1 of 1	No Cat	CO = 0							
547	Chlorpyrifos	2921-88-2	Alkoxy| Aromatic heterocyclic halide| Aryl halide| Thiophosphate	S	Y	NICEATM	SCBT	≥98%	1 of 1	No Cat	CO = 0							
548	Dequest 2016	29329-71-3	Alcohol| Phosphonic acid	S	N	NICEATM	Unknown	Unknown	1 of 1	No Cat	CO = 0						No CAS # reported in NICEATM ALTTOX; CAS No. 29329-71-3 (x-Na), 3794-83-0 (4-Na), depends on the # of Na ions	?
549	Disperse red 60 (Chemical name: 1-amino-4-hydroxy-2-phenoxy-9,10-anthracenedione)	17418-58-5	Aniline| Anthracenone/ Antracendione| Aryl| Ether| Phenol	S	Y	NICEATM	Sigma-Aldrich	Unknown	1 of 1	No Cat	CO = 0							
550	EPIKURE 1061	2716-10-1	N/A	S	Y	NICEATM	Sigma-Aldrich	99%	1 of 1	No Cat	CO = 0							
551	EPIKURE 1062	2716-12-3	N/A	S	Y	NICEATM	Unknown	Unknown	1 of 1	No Cat	CO = 0							?
552	EPIKOTE RSS 1079	47758-37-2	Aryl| Ether| Fluorene| Saturated heterocyclic fragment	S	Y	NICEATM	TCI	>98%	1 of 1	No Cat	CO = 0							

Study Number	Test Chemical Name	CAS RN	Organic Functional Groups	Physical Form as tested	Physical Form Confirmation e.g., by MSDS (Y / N)	Data Source	Commercial Source	Available Purity	Number of Studies	UN GHS Classification	Drivers of Classification						Comments	Should Not Be Used
											Severity		Persistence		Specific Observations			
											Cut-off values	Number of animals	Cut-off time	Number of animals	CO = 4 or other observations	Number of animals		
553	Etoxazole	153233-91-1	Alkoxy| Aryl| Aryl halide| Ether| tert-Butyl| Unsaturated heterocyclic fragment	S	Y	EURL ECVAM	Sigma-Aldrich	analytical standard	1 of 1	No Cat	CO = 0							
554	Famoxadone	131807-57-3	Aryl| Carbamate| Ether| Imide| Oxazolidine derivatives| Saturated heterocyclic fragment	S	Y	EURL ECVAM	Toronto Research Chemicals	Unknown	1 of 1	No Cat	CO = 0							
555	Fenamidone	161326-34-7	Aryl| Imidazoline| Sulfide| Unsaturated heterocyclic amine| Unsaturated heterocyclic fragment	S	Y	EURL ECVAM	Sigma-Aldrich	analytical standard	1 of 1	No Cat	CO = 0							
556	Flonicamid	158062-67-0	Alkyl halide| Aryl| Carboxamide| Nitrile| Pyridine	S	Y	EURL ECVAM	Sigma-Aldrich	analytical standard	1 of 1	No Cat	CO = 0							
557	Fluopicolide	239110-15-7	Alkyl halide| Aromatic heterocyclic halide| Aryl halide| Benzamide	S	Y	EURL ECVAM	Sigma-Aldrich	analytical standard	1 of 1	No Cat	CO = 0							
558	Gluconolactone	90-80-2	Dihydroxyl group| Lactone| Saturated heterocyclic fragment	S	Y	LNS	Sigma-Aldrich	≥99%	1 of 1	No Cat	CO = 0							
559	Hexabromocyclododecane	25637-99-4	Alkyl halide| Cycloalkane	S	Y	NICEATM	MPI Chemie BV	≥98%	1 of 1	No Cat	CO = 0							
560	Hexamethylenetetraamine	100-97-0	Bridged-ring heterocycles| Saturated heterocyclic amine| Saturated heterocyclic fragment	S	Y	ZEBET	Sigma-Aldrich	≥99%	1 of 1	No Cat	CO = 0							
561	Hexyl 2-(1-(diethylaminohydroxyphenyl)methanoyl) benzoate	302776-68-7	Aromatic amine| Aryl| Carboxylic acid ester| Ketone| Phenol	S	Y	EURL ECVAM	Sigma-Aldrich	≥98%	1 of 1	No Cat	CO = 0							
562	Methyl p-hydroxybenzoate (neat)	99-76-3	Carboxylic acid ester| Phenol	S	Y	NICEATM	Sigma-Aldrich	≥99%	1 of 1	No Cat	CO = 0							
563	Myristyl myristate	3234-85-3	Carboxylic acid ester	S	Y	ECETOC	Chemos GmbH	98.4%	1 of 1	No Cat	CO = 0							
564	N-Allyl-N-(4-amino-2-nitrophenyl)amine	160219-76-1	Allyl| Aniline| Nitrobenzene	S	Y	Cosmetics Europe	Proprietary	>98%	1 of 1	No Cat	CO = 0							?
565	Phenothiazine	92-84-2	Phenothiazine	S	Y	NICEATM	Sigma-Aldrich	≥98%	1 of 1	No Cat	CO = 0							
566	Phenylbutazone	50-33-9	Aryl| Pyrazolidinedione/ Pyrazolidone	S	Y	LNS	Sigma-Aldrich	≥98.5%	1 of 1	No Cat	CO = 0							
567	Phenylephrine hydrochloride	61-76-7	Alcohol| Aliphatic Amine, secondary| Phenol	S	Y	ZEBET	Fisher Scientific	99%	1 of 1	No Cat	CO = 0							
568	Phenylthiourea	103-85-5	Aryl| Thiourea derivatives	S	Y	ZEBET	Sigma-Aldrich	≥98%	1 of 1	No Cat	CO = 0							
569	Piperazine	110-85-0 (18833-13-1 reported in ZEBET, which corresponds to Acefylline piperazinate)	Piperazine| Saturated heterocyclic amine| Saturated heterocyclic fragment	S	Y	ZEBET	Sigma-Aldrich	≥99%	1 of 1	No Cat	CO = 0							
570	Potassium tetrafluoroborate	14075-53-7	Inorganic Salt	S	Y	ECETOC	Sigma-Aldrich	96%	1 of 1	No Cat	CO = 0							
571	Potato starch	56780-58-6	N/A	S	Y	ZEBET	Unknown	Unknown	1 of 1	No Cat	CO = 0							?
572	Silicic acid (neat)	1343-98-2	N/A	S	Y	NICEATM	Sigma-Aldrich	99.9%	1 of 1	No Cat	CO = 0							
573	Sodium tripolyphosphate (Grade E, anhydrous) (10 mg)	7758-29-4	N/A	S	Y	NICEATM	Sigma-Aldrich	≥98%	1 of 1	No Cat	CO = 0						10 mg dose	
574	Tetrabromobisphenol A (1 of 2)	79-94-7	Aryl halide| Phenol	S	Y	NICEATM	Sigma-Aldrich	97%	1 of 2	No Cat	CO = 0							
575	Theobromine	83-67-0	N/A	S	Y	ZEBET	Sigma-Aldrich	≥99%	1 of 1	No Cat	CO = 0							
576	Theophylline sodium (neat)	3485-82-3	Dihydropurinedione| Urea derivatives	S	Y	ZEBET	Unknown	Unknown	1 of 1	No Cat	CO = 0							?
577	Theophylline sodium acetate	8002-89-9	N/A	S	Y	ZEBET	TCI	≥98%	1 of 1	No Cat	CO = 0							
578	Thiencarbazone-methyl	317815-83-1	Allyl| Amidine| Carboxylic acid ester| Ether| Hydrazone| Imide| Lactam| Sulfonamide| Sulfonyl urea| Thiophene	S	Y	EURL ECVAM	Sigma-Aldrich	analytical standard	1 of 1	No Cat	CO = 0							
579	Tris(2-ethylhexyl)-4,4',4''-(1,3,5-triazine-2,4,6-triyltriimino) tribenzoate	88122-99-0	Alkane, branched with tertiary carbon| Aromatic amine| Aryl| Carboxylic acid ester| Melamine	S	Y	EURL ECVAM	Proprietary	>98%	1 of 1	No Cat	CO = 0							?
580	Trisodium mono-(5-(1,2-dihydroxyethyl)-4-oxido-2-oxo-2,5-dihydro-furan-3-yl) phosphate	66170-10-3	Dihydroxyl group| Enol| Furanone/ Furanondione| Phosphate ester	S	Y	EURL ECVAM	Sigma-Aldrich	≥95%	1 of 1	No Cat	CO = 0							
581	Xanthinol nicotinate	437-74-1	Alcohol| Aliphatic Amine, tertiary| Aryl| Carboxylic acid| Fused unsaturated heterocycles| Pyridine| Pyrimidine derivatives| Urea derivatives	S	Y	ZEBET	Sigma-Aldrich	≥99%	1 of 1	No Cat	CO = 0							
582	5-Chloro-2,4-disulphamoyl chloroacetanilide	Unknown	N/A	Unknown	N	NICEATM	Unknown	Unknown	1 of 1	No Cat	CO = 0							?
583	C10 alpha-Olefins (neat)	68855-58-3	Allyl	Unknown	N	NICEATM	Unknown	Unknown	1 of 1	No Cat	CO = 0							?
584	C16 alpha-Olefin (neat)	68855-58-3	Allyl	Unknown	N	NICEATM	Unknown	Unknown	1 of 1	No Cat	CO = 0							?
585	Cerium-2-ethylhexanoate	24593-34-8 (other possible CAS # may be 7435-02-1 and 56797-01-4)	Carboxylic acid	Unknown	N	ZEBET	Unknown	Unknown	1 of 1	No Cat	CO = 0							?
586	iso-Tridecanal	61497-46-9	Aldehyde| Isopropyl	Unknown	N	ZEBET	Unknown	Unknown	1 of 1	No Cat	CO = 0							?
587	Silan 165 (Chemical name: 3,3,11,11-Tetramethoxy-2,12-dioxa-7-thia-3,11-disilatridecane)	29055-11-6	AlkoxySilane| Sulfide	Unknown	N	ZEBET	Unknown	Unknown	1 of 1	No Cat	CO = 0							?
588	2,2-Dimethylbutanoic acid	595-37-9	Carboxylic acid	L	Y	ECETOC	Sigma-Aldrich	96%	1 of 1	SCNM (Cat 1)	CO mean ≥ 1; Conj mean ≥ 2	6/6; 6/6	**Possibly CO pers D21** (at least)	at least 1/6			Study terminated on D14 with CO = 3 in 1/6, CO = 2 in 2/6, CO = 1 in 3/6, IR = 2 in 1/6, IR = 1 in 1/6, IR = 0 in 4/6, CR = 1 in 1/6, CR = 0 in 5/6 and CC = 0 in 6/6; Possibly CO irreversible	X
589	iso-Butanol (2 of 2)	78-83-1	Alcohol| Isopropyl	L	Y	ZEBET	Sigma-Aldrich	≥99%	2 of 2	SCNM (Cat 1)	Conj mean ≥ 2; CO mean ≥ 1	3/3; 2/3	**Possibly CO pers D21** (at least)	1/3			Delayed effects; Study terminated on D14 with CO = 3 in 1/3, CO = 0 in 2/3, IR = 0 in 3/3, CR = 1 in 1/3, CR = 0 in 2/3, and CC = 0 in 3/3; CO fully reversible by D14 in 1/3 and by D1 in 1/3; D7 not recorded; Possibly CO irreversible	X
590	Triton X-100 (neat) (3 of 3)	9002-93-1	Alcohol| Alkane branched with quaternary carbon| Aryl| Ether| tert-Butyl	L	Y	NICEATM	Sigma-Aldrich	laboratory grade	3 of 3	SCNM (Cat 1)	CO mean ≥ 1	3/3	**Possibly CO pers D21** (at least)	1/3			Delayed effects; Study terminated on D14 with CO = 3 in 1/3, CO = 0 in 2/3, IR = 1 in 3/3, CR = 1 in 1/3, CR = 0 in 2/3, CC = 2 in 1/3 and CC = 0 in 2/3; Possibly CO irreversible	
591	Benzyl alcohol (10%)	100-51-6	Alcohol| Benzyl	L (tested in solvent, available as S)	Y	NICEATM	Sigma-Aldrich	99.8%	1 of 1	SCNM (Cat 1)	CO mean ≥ 1	3/3	**Possibly CO pers D21** (at least)	1/3			Delayed effects; Study terminated on D14 with CO = 3, IR = 1, CR = 3 and CC = 2 in 1/3 and all other animals fully reversed by D9 or earlier; Possibly CO irreversible	X
592	Dibutyltin dilaurate	77-58-7	Carboxylic acid| Tin, organo	L	Y	NICEATM	Sigma-Aldrich	95%	1 of 1	SCNM (Cat 1)	CO mean ≥ 1; Conj mean ≥ 2; IR mean ≥ 1	3/3; 3/3; 3/3	**Possibly Conj pers D21**	2/3			Study terminated on D14 with CO = 0 in 3/3, IR = 0 in 3/3, CR = 3 in 2/3, CR = 0 in 1/3, CC = 4 in 1/3, CC = 3 in 1/3 and CC = 0 in 1/3; 1 animal fully reversed by D14; Possibly Conj irreversible	X
593	Surfonic N-95 (Chemical name: Nonyl phenol ethoxylate, branched)	9016-45-9	Alcohol| Benzyl| Ether	L	Y	NICEATM	Huntsman	100%	1 of 1	SCNM (Cat 1)	CO mean ≥ 1; Conj mean ≥ 2; IR mean ≥ 1	6/6; 6/6; 5/6	Conj pers D7; CO pers D7; IR pers D7	5/6; 4/6; 1/6			Study terminated on D14 with CO = 2 in 1/6, CO = 1 in 1/6, IR = 1 in 1/6, CR = 2 in 1/6, and CC = 3 in 1/6; 1 animal fully reversed by D7; 3 animals fully reversed by D14	X
594	Surfonic N-85 (Chemical name: Nonyl phenol ethoxylate, branched)	9016-45-9	Alcohol| Benzyl| Ether	L	Y	NICEATM	Unknown	Unknown	1 of 1	SCNM (Cat 1)	IR mean ≥ 1; CO mean ≥ 1; Conj mean ≥ 2	6/6; 5/6; 5/6	Conj pers D7; CO pers D7; IR pers D7	6/6; 4/6; 2/6			Study terminated on D14 with CO = 2 in 3/6, IR = 1 in 1/6, CR = 1 in 6/6, CC = 2 in 4/6 and CC = 1 in 1/6	X

Study Number	Test Chemical Name	CAS RN	Organic Functional Groups	Physical Form as tested	Physical Form Confirmation e.g., by MSDS (Y / N)	Data Source	Commercial Source	Available Purity	Number of Studies	UN GHS Classification	Drivers of Classification						Comments	Should Not Be Used
											Severity		Persistence		Specific Observations			
											Cut-off values	Number of animals	Cut-off time	Number of animals	CO = 4 or other observations	Number of animals		
595	Chlorendic anhydride	115-27-5	Acid anhydride| Alkenyl halide| Alkyl halide| Bridged-ring carbocycles| Fused saturated heterocycles| Oxolane| Saturated heterocyclic fragment	S	Y	NICEATM	Sigma-Aldrich	97%	1 of 1	SCNM<br>(Cat 1)	IR mean ≥ 1; Conj mean ≥ 2	5/6; 4/6	Conj pers D7; IR pers D7; CO pers D7	5/6; 4/6; 2/6			Study terminated on D14 with CO = 2 in 3/6, CO = 1 in 2/6, IR = 1 in 4/6, CR = 1 in 5/6, CC = 2 in 1/6 and CC = 1 in 4/6	X
596	iso-Decylglucoside	Unknown	N/A	Unknown	N	ZEBET	Unknown	Unknown	1 of 1	SCNM<br>(Cat 1)	CO mean ≥ 1; Conj mean ≥ 2	3/3; 3/3	CO pers D7; Conj pers D7	3/3; 3/3			Delayed effects; Study terminated on D14 with CO = 2, CR = 2 and CC = 2 or 1 in 2/3; 1 animal fully reversed by D14	X
597	Methyl phosphonic acid bis (oxiranylmethyl) ester	77375-34-9 (77275-34-9 reported in ZEBET, which is wrong)	N/A	Unknown	N	ZEBET	Unknown	Unknown	1 of 1	SCNM<br>(Cat 1)	CO mean ≥ 1; Conj mean ≥ 2	2/3; 2/3	CO pers D7; Conj pers D7; IR pers D7	1/3; 1/3; 1/3			Study terminated on D14 with CO = 2 in 1/3; 1 animals fully reversed by D7; CO = 0 on D2 in 1/3	X
598	Sodium hydrogenated tallow L-glutamine	Unknown	N/A	Unknown	N	NICEATM	Unknown	Unknown	1 of 1	SCNM<br>(Cat 1)	CO mean ≥ 1	3/3	CO pers D7; Conj pers D7; IR pers D7	1/3; 1/3; 1/3			Study terminated on D14 with CO = 2, IR = 1, CR = 1 and CC = 1 in 1/3	X
599	Alcohol SDA 39C (95%)	64-17-5 (Ethanol)<br>84-66-6 (Diethylphthalate)	Ethanol	L	Y	NICEATM	Fisher Scientific;<br>PHARMCO-AAPER	Unknown	1 of 1	SCNM<br>(Cat 2A or higher)	CO mean ≥ 1; Conj mean ≥ 2	3/3; 3/3	CO pers D7; Conj pers D7	1/3; 1/3			Study terminated on D10 with CR = 2 and CC = 1 in 1/3; 2 animals fully reversed by D7<br><br>SDA = special denatured alcohol (99.01% (v/v) Ethanol + 0.99% (v/v) Diethylphthalate)	X
600	alpha-Hexyl cinnamic aldehyde (5% in Alcohol SDA 39C)	101-86-0	Alpha,beta unsaturated aldehyde| Benzyl	L	Y	NICEATM	Sigma-Aldrich (for alpha-Hexyl cinnamic aldehyde); Fisher Scientific (for Alcohol SDA 39C)	≥95%	1 of 1	SCNM<br>(Cat 2A or higher)	Conj mean ≥ 2; CO mean ≥ 1	3/3; 2/3	CO pers D7; Conj pers D7	2/3; 2/3			Study terminated on D10 with CO = 1 in 2/3, CR = 2 in 2/3, CC = 2 in 1/3 and CC = 1 in 1/3; 1 animal fully reversed by D4<br><br>SDA = special denatured alcohol (99.01% (v/v) Ethanol + 0.99% (v/v) Diethylphthalate)	X
601	Emulphogene BC-610 (Chemical name: Polyoxyethylene (12) Tridecyl Ether)	78330-21-9	Alcohol| Alkoxy| Ether	L	Y	NICEATM	Sigma-Aldrich	Unknown	1 of 1	SCNM<br>(Cat 2A or higher)	CO mean ≥ 1; Conj mean ≥ 2; IR mean ≥ 1	6/6; 6/6; 5/6	CO pers D7; Conj pers D7; IR pers D7	6/6; 6/6; 5/6			Study terminated on D7	X
602	Ethanol (neat) (4 of 4)	64-17-5	Alcohol	L	Y	NICEATM	Sigma-Aldrich	>99.8%	4 of 4	SCNM<br>(Cat 2A or higher)	CO mean ≥ 1	2/3	CO pers D7; Conj pers D7	2/3; 1/3			Study terminated on D14 with CO = 1 in 2/3; CO = 0 on D2 in 1/3; 1 animal fully reversed by D5	X
603	Furfuryl alcohol	98-00-0	Alcohol| Allyl| Aryl| Furane	L	Y	ECETOC	Sigma-Aldrich	≥98	1 of 1	SCNM<br>(Cat 2A or higher)	CO mean ≥ 1; IR mean ≥ 1	1/1; 1/1	CO pers D7; Conj pers D7	1/1; 1/1			One single animal fully reversed by D14; Observations indicating irritation	X
604	Igepal CA-720 (Chemical name: Octyl phenol ethoxylate, branched)	9036-19-5 or 9002-93-1 (9016-45-9 reported in NICEATM ALTTOX, which is wrong)	Alcohol| Alkane branched with quaternary carbon| Aryl| Ether| tert-Butyl	L	Y	NICEATM	Sigma-Aldrich	Unknown	1 of 1	SCNM<br>(Cat 2A or higher)	CO mean ≥ 1; Conj mean ≥ 2; IR mean ≥ 1	6/6; 6/6; 6/6	CO pers D7; Conj pers D7; IR pers D7	6/6; 6/6; 4/6			Study terminated on D7	X
605	Igepal CO-720 (Chemical name: Nonyl phenol ethoxylate, branched)	68412-54-4 or 9016-45-9 or 26027-38-3 (9016-45-9 reported in NICEATM ALTOX)	Alcohol| Benzyl| Ether	L	Y	NICEATM	Sigma-Aldrich	Unknown	1 of 1	SCNM<br>(Cat 2A or higher)	CO mean ≥ 1; Conj mean ≥ 2	6/6; 6/6	CO pers D7; Conj pers D7; IR pers D7	6/6; 6/6; 5/6			Study terminated on D7	X
606	Isazofos (Cheminal name: O-(5-Chlor-1-isopropyl-1,2,4-triazol-3-yl)-O,O-diethylthiophosphate)	42509-80-8	Alkoxy| Aryl halide| Isopropyl| Thiophosphate	L	Y	NICEATM	SCBT	>98%	1 of 1	SCNM<br>(Cat 2A or higher)	CO mean ≥ 1; Conj mean ≥ 2; IR mean ≥ 1	3/3; 2/3; 2/3	CO pers D7	3/3			Study terminaterd on D14 with CO = 1 in 1/3; 2 animals fully reversed by D14	X
607	Polysolvan (Chemical name: n-Butyl glycolate)	7397-62-8	Alcohol| Carboxylic acid ester	L	Y	ZEBET	Sigma-Aldrich	≥90%	1 of 1	SCNM<br>(Cat 2A or higher)	CO mean ≥ 1; Conj mean ≥ 2; IR mean ≥ 1	3/3; 3/3; 2/3	CO pers D7; Conj pers D7; IR pers D7	1/3; 1/3; 1/3			Delayed effects; Study terminated on D7 with CO = 3, IR = 1, CR = 2 and CC = 1 in 1/3; 2 animals fully reversed by D7	X
608	Silan 253 (Chemical name: 3-(Triethoxysilyl)-N,N-bis-3-(triethoxysilyl)-propyl-propane-1-amine)	18784-74-2	Aliphatic Amine, tertiary| Alkoxy| AlkoxySilane	L	Y	ZEBET	Fluorochem	100%	1 of 1	SCNM<br>(Cat 2A or higher)	CO mean ≥ 1; Conj mean ≥ 2	3/3; 3/3	CO pers D7; Conj pers D7	2/3; 2/3			At least 2/3 for CO, CR and CC persistence on D7; Study terminated on D4 in 1/3 and on D7 in 2/3	X
609	Sodium lauryl ether sulphate (unknown concentration)	3088-31-1	Alkoxy| Ether| Sulfate	L	N	ZEBET	Unknown	Unknown	1 of 1	SCNM<br>(Cat 2A or higher)	Conj mean ≥ 2	5/6	Conj pers D7	5/6			Study terminated on D7	X
610	Cetylpyridinium chloride (1%)	6004-24-6	Ammonium salt| Aryl| Pyridine	L (tested in solvent, available as S)	Y	NICEATM	Sigma-Aldrich	>99%	1 of 1	SCNM<br>(Cat 2A or higher)	Conj mean ≥ 2; CO mean ≥ 1	3/3; 2/3	Conj pers D7; CO Pers D7	3/3; 2/3			Study terminated on D14 with CO = 1 in 1/3; CO = 0 on D2 in 1/3; CO = 0 on D8 in 1/3; 1 animal fully reversed by D9; 1 animal fully reversed by D10	X
611	Aluminium sulphate hydrate	17927-65-0 (10043-01-3 reported in NICEATM ALTTOX, which corresponds to Aluminium sulphate)	N/A	S	Y	NICEATM	Sigma-Aldrich	98%	1 of 1	SCNM<br>(Cat 2A or higher)	CO mean ≥ 1; Conj mean ≥ 2	at least 5/6; 5/6	CO pers D7; Conj pers D7	6/6; 6/6			Study terminated on D7; 10 mg dose	X
612	Ethoxyphenylenediamine	1197-37-1	Alkoxy| Ether| Precursors quinoid compounds	S	N	NICEATM	Unknown	Unknown	1 of 1	SCNM<br>(Cat 2A or higher)	Conj mean ≥ 2; IR mean ≥ 1	3/3; 2/3	Conj pers D7; CO pers D7	2/3; 1/3			Study terminated on D14 with CR = 1 and CC = 2 in 1/3, with CO fully reversing on D14 and IR fully reversing on D7; CR fully reversed in the other two animals on day 7 and day 14; CC fully reversed in the other two animals on day 7 in both animals; CO fully reversed in the other two animals on day 2 and day 7; IR fully reversed in the other two animals on day 3 and day 7	X
613	n-Butyl carbamate	592-35-8	Carbamate	S	Y	ZEBET	Sigma-Aldrich	98%	1 of 1	SCNM<br>(Cat 2A or higher)	CO mean ≥ 1; Conj mean ≥ 2; IR mean ≥ 1	3/3; 3/3; 3/3	CO pers D7; Conj pers D7	3/3; 3/3			Study terminated on D7	X
614	o-Phenylenediamine (1 of 2)	95-54-5	Precursors quinoid compounds	S	Y	ZEBET	Sigma-Aldrich	≥99%	1 of 2	SCNM<br>(Cat 2A or higher)	CO mean ≥ 1; Conj mean ≥ 2; IR mean ≥ 1	3/3; 3/3; 3/3	Conj pers D7; CO pers D7; IR pers D7	3/3; 1/3; 1/3			Study terminated on D7	X
615	o-Phenylenediamine (2 of 2)	95-54-5	Precursors quinoid compounds	S	Y	NICEATM	Sigma-Aldrich	≥99%	2 of 2	SCNM<br>(Cat 2A or higher)	CO mean ≥ 1	2/3	CO pers D7; Conj pers D7; IR pers D7	2/3; 2/3; 1/3			Study terminaterd on D14 with CO = 1 in 1/3, CR = 1 in 2/3 and CC = 2 in 1/3; 1 animal fully reversed by D7	X
616	Sodium lauryl sulphate (neat)	151-21-3	Alkoxy| Sulfate	S	Y	NICEATM	Sigma-Aldrich	≥99%	1 of 1	SCNM<br>(Cat 2A or higher)	CO mean ≥ 1	2/3	Conj pers D7	1/3			Study terminated on D14 with CR = 1 in 1/3; 1 animal fully reversed by D3; 1 animal fully reversed by D6	X
617	Sodium pyrosulphite (Sodium disulphite)	7681-57-4	N/A	S	Y	ZEBET	Sigma-Aldrich	98-100.5%	1 of 1	SCNM<br>(Cat 2A or higher)	CO mean ≥ 1; Conj mean ≥ 2; IR mean ≥ 1	3/3; 3/3; 3/3	CO pers D7; Conj pers D7; IR pers D7	3/3; 3/3; 3/3			Study terminated on D7; Delayed effects	X
618	Sucrose fatty acid ester (neat)	Unknown	N/A	S	N	NICEATM	Unknown	Unknown	1 of 1	SCNM<br>(Cat 2A or higher)	CO mean ≥ 1	2/3	CO pers D7; Conj pers D7; IR pers D7	2/3; 1/3; 1/3			Study terminated on D14 with CO = 1 in 1/3; CO = 0 in the whole study in 1/3; CO = 0 on D8 in 1/3; 1 animal fully reversed by D2; 1 animal fully reversed by D8	X
619	1-Piperidinecarboxaldehyde	2591-86-8	Formylamino| Piperidine| Saturated heterocyclic amine| Saturated heterocyclic fragment	L	Y	NICEATM	Sigma-Aldrich	99%	1 of 1	SCNM<br>(Cat 2 or Cat 1)	CO mean ≥ 1; Conj mean ≥ 2	6/6; 6/6					Study terminated on D3	X
620	2,2,3-Trimethyl-3-cyclo-pentene-1-acetaldehyde	4501-58-0	Allyl| Cycloalkene| Ketone	L	Y	ZEBET	Parchem	85-100%	1 of 1	SCNM<br>(Cat 2 or Cat 1)	CO mean ≥ 1	2/3					Study terminated on D4 or D7 (unclear); May be Cat 2A based on CO and/or CR persistence on D7 in 2/3 (unclear if data reports do D7 or D4)	X
621	2,3-Dibromopropanol	96-13-9	Alcohol| Alkyl halide	L	Y	NICEATM	Sigma-Aldrich	98%	1 of 1	SCNM<br>(Cat 2 or Cat 1)	CO mean ≥ 1; Conj mean ≥ 2	6/6; 5/6					Study terminated on D3	X
622	3-Cyclohexene-1-methanol	1679-51-2	Alcohol| Alkane, branched with tertiary carbon| Allyl| Cycloalkene	L	Y	ZEBET	Sigma-Aldrich	98%	1 of 1	SCNM<br>(Cat 2 or Cat 1)	CO mean ≥ 1; Conj mean ≥ 2	3/3; 3/3					Study terminated on D3	X
623	Diethylenetriamine-pentaacetic acid pentasodium salt (unknown concentration; assumed to be ~40%, aqueous)	140-01-2	Aliphatic Amine, tertiary| Carboxylic acid	L	Y	ZEBET	Sigma-Aldrich	purum; ~40%, aqueous	1 of 1	SCNM<br>(Cat 2 or Cat 1)	CO mean ≥ 1; Conj mean ≥ 2	2/3; 2/3					Study terminated on D3	X
624	Methyl trifluoroacetate	431-47-0	Alkyl halide| Carboxylic acid ester	L	Y	NICEATM	Sigma-Aldrich	99%	1 of 1	SCNM<br>(Cat 2 or Cat 1)	CO mean ≥ 1; IR mean ≥ 1	5/6; 5/6					Study terminated on D3	X
625	Parafluoroaniline	371-40-4	Aniline| Aryl halide	L	Y	ECETOC	Sigma-Aldrich	99%	1 of 1	SCNM<br>(Cat 2 or Cat 1)	CO mean ≥ 1; Conj mean ≥ 2	6/6; 6/6					Study terminated on D3	X
626	iso-Cyanic acid polymethylene polyphenylene ester	9016-87-9	Benzyl| Isocyanate	L	Y	NICEATM	Sigma-Aldrich	Unknown	1 of 1	SCNM<br>(Cat 2 or Cat 1)	Conj mean ≥ 2	4/6					Study terminated on D3	X
627	Toluene (2 of 2)	108-88-3	Aryl	L	Y	NICEATM	Sigma-Aldrich	≥99.9%	2 of 2	SCNM<br>(Cat 2 or Cat 1)	Conj mean ≥ 2	4/6					Study terminated on D3	X
628	Toluene diisocyanate	26471-62-5	Aryl| Isocyanate	L	Y	NICEATM	Merck	Unknown	1 of 1	SCNM<br>(Cat 2 or Cat 1)	Conj mean ≥ 2	6/6					Study terminated on D3	X
629	1,2-Dodecanediol	1119-87-5	Dihydroxyl group	S	Y	ZEBET	Sigma-Aldrich	90%	1 of 1	SCNM<br>(Cat 2 or Cat 1)	CO mean ≥ 1; IR mean ≥ 1	3/3; 3/3					Study terminated on D3	X
630	3-Mercapto-1,2,4-triazole	3179-31-5	Aryl| Thiourea derivatives| Triazole	S	Y	ZEBET	Sigma-Aldrich	97%	1 of 1	SCNM<br>(Cat 2 or Cat 1)	CO mean ≥ 1; Conj mean ≥ 2	3/3; 3/3					Study terminated on D3	X
631	6-Methyl Purine	2004-03-7	Purine derivatives	S	Y	NICEATM	Sigma-Aldrich	≥99%	1 of 1	SCNM<br>(Cat 2 or Cat 1)	CO mean ≥ 1	6/6					Study terminated on D3	X
632	Hyton (Chemical name: 2-Amino-5-phenyl-1,3-oxazol-4(5H)-one)	2152-34-3	Amidine| Aryl| Lactams| Oxazolidine derivatives| Saturated heterocyclic fragment	S	Y	ZEBET	Sigma-Aldrich	≥98%	1 of 1	SCNM<br>(Cat 2 or Cat 1)	CO mean ≥ 1; Conj mean ≥ 2	3/3; 3/3					Study terminated on D3;<br>Available in Sigma under product number P0048	X
633	Iminodiacetic acid	142-73-4	Sarcosine	S	Y	ZEBET	Sigma-Aldrich	≥98%	1 of 1	SCNM<br>(Cat 2 or Cat 1)	CO mean ≥ 1; Conj mean ≥ 2; IR mean ≥ 1	3/3; 3/3; 2/3					Study terminated on D3	X
634	L-Aspartic acid	56-84-8	Alpha amino acid| Carboxylic acid	S	Y	ECETOC / ZEBET	Sigma-Aldrich	≥99%	1 of 1	SCNM<br>(Cat 2 or Cat 1)	CO mean ≥ 1; IR mean ≥ 1	2/3; 2/3					Study terminated on D3; Two studies are reported in ECETOC and ZEBET with the exact same animal scores and are considered duplicate entries	X
635	L-Glutamic acid HCL	138-15-8	Alpha amino acid| Carboxylic acid	S	Y	ZEBET	Sigma-Aldrich	>99%	1 of 1	SCNM<br>(Cat 2 or Cat 1)	CO mean ≥ 1; Conj mean ≥ 2; IR mean ≥ 1	2/3; 2/3; 2/3					Study terminated on D3	X
636	Nicotinamide	98-92-0	Aryl| Carboxamide| Pyridine	S	Y	ZEBET	Sigma-Aldrich	≥99.5%	1 of 1	SCNM<br>(Cat 2 or Cat 1)	CO mean ≥ 1; Conj mean ≥ 2; IR mean ≥ 1	3/3; 3/3; 2/3					Study terminated on D3	X
637	Potassium hexacyanoferrate II	14459-95-1	N/A	S	Y	ZEBET	Sigma-Aldrich	≥99.5%	1 of 1	SCNM<br>(Cat 2 or Cat 1)	CO mean ≥ 1	2/3					Study terminated on D3	X
638	Potassium hexacyanoferrate III	13746-66-2	N/A	S	Y	ZEBET	Sigma-Aldrich	≥99%	1 of 1	SCNM<br>(Cat 2 or Cat 1)	CO mean ≥ 1	3/3					Study terminated on D3	X
639	TPBP C4 Amide (Chemical name: Phosphonium, triphenyl(phenyl-methyl)-, salt with 1,1,2,2,3,3,4,4,4 -nonafluoro-N-methyl-1 butanesulfonamide (1:1))	332350-93-3	N/A	S	Y	NICEATM	Unknown	Unknown	1 of 1	SCNM<br>(Cat 2 or Cat 1)	CO mean ≥ 1; Conj mean ≥ 2; IR mean ≥ 1	3/3; 3/3; 3/3					Study terminated on D3 with CO = 2 in 3/3	X

Study Number	Test Chemical Name	CAS RN	Organic Functional Groups	Physical Form as tested	Physical Form Confirmation e.g., by MSDS (Y / N)	Data Source	Commercial Source	Available Purity	Number of Studies	UN GHS Classification	Drivers of Classification						Comments	Should Not Be Used
											Severity		Persistence		Specific Observations			
											Cut-off values	Number of animals	Cut-off time	Number of animals	CO = 4 or other observations	Number of animals		
640	4,4-Diphenylmethane diisocyanate	101-68-8	Benzyl| Isocyanate	S	Y	NICEATM	Sigma-Aldrich	98%	1 of 1	SCNM<br>(Cat 2 or Cat 1)	Conj mean ≥ 2; IR mean ≥ 1	5/6; at least 4/6					Study terminated on D3	X
641	Potassium cyanate	590-28-3	N/A	S	Y	ECETOC / ZEBET	Sigma-Aldrich	96%	1 of 1	SCNM<br>(Cat 2 or Cat 1)	Conj mean ≥ 2	3/3					Study terminated on D3; Two studies are reported in ECETOC and ZEBET with the exact same animal scores and are considered duplicate entries	X
642	Sodium silicate (2.4 ratio hydrous)	1344-09-8	N/A	S	Y	NICEATM	PQ Corporation;<br>Samarth Chemical Products	Unknown	1 of 1	SCNM<br>(Cat 2 or Cat 1)	Conj mean ≥ 2	5/6					Study terminated on D3; 10 mg dose	X
643	Thiazolium, 3-ethyl-2-[3-(3-ethyl-2-thizolidinylidene)-1-propenyl]-4,5-dihydro-iodide	3065-71-2	Ammonium salt| Conjugated system| Saturated heterocyclic fragment| Sulfide| Thiazolidine| Thiazoline| Unsaturated heterocyclic fragment	S	Y	NICEATM	Unknown	Unknown	1 of 1	SCNM<br>(Cat 2 or Cat 1)	Conj mean ≥ 2	3/3					Study terminated on D2 due to extreme chemosis in all animals (score 4); For all other endpoints only D1 scores available	X
644	Diacetone alcohol	123-42-2	Alcohol| Ketone	L	Y	LNS	Sigma-Aldrich	99%	1 of 1	SCNM<br>(Cat 2A)	CO mean ≥ 1; Conj mean ≥ 2	3/3; 2/3	CO pers D7	1/3			Original study indicates a CO = 1 in 1/3 on D14; However, study comment states full reversibility on D14; Most likely, it was meant as D21	X
645	Poly(ethylene glycol) butyl ether	9004-77-7	Alcohol| Alkoxy| Ether	L	Y	ZEBET	Sigma-Aldrich	Unknown	1 of 1	SCNM<br>(Cat 2A)	CO mean ≥ 1; Conj mean ≥ 2; IR mean ≥ 1	3/3; 3/3; 2/3	Conj pers D7; CO Pers D7	2/3; 1/3			Study terminated on D7	X
646	p-Ethyltoluene	622-96-8	Aryl	L	Y	NICEATM	Sigma-Aldrich	≥95%	1 of 1	SCNM<br>(Cat 2A)	Conj mean ≥ 2	4/6	Conj pers D7	4/6			Study terminaterd on D7 with CR = 3 in 1/6, CR = 2 in 2/6, CR = 1 in 1/6 and CC = 1 in 2/6; 1 animal fully reversed by D4; 1 animal fully reversed by D7	X
647	Triton X-100 (5%) (2 of 2)	9002-93-1	Alcohol| Alkane branched with quaternary carbon| Aryl| Ether| tert-Butyl	L	Y	ECETOC	Sigma-Aldrich	laboratory grade	2 of 2	SCNM<br>(Cat 2A)	CO mean ≥ 1; Conj mean ≥ 2	5/6; 4/6	Conj pers D7	4/6			Study terminated on D7 with CC = 1 in 1/6; 1 animal fully reversed by D3, 1 animal fully reversed by D7 and 3 animals fully reversed by D10	
648	p-Anisidine	104-94-9	Aniline| Ether	S	Y	ZEBET	Sigma-Aldrich	≥99%	1 of 1	SCNM<br>(Cat 2A)	CO mean ≥ 1; Conj mean ≥ 2; IR mean ≥ 1	3/3; 3/3; 3/3	Conj pers D7; CO Pers D7	3/3; 2/3			Study terminated on D7	X
649	Maneb	12427-38-2	Thiocarbamate	S	Y	ECETOC	Sigma-Aldrich	analytical standard	1 of 1	SCNM<br>(Cat 2A)	Conj mean ≥ 2	6/6	Conj pers D7	2/6			Study terminated on D7 with CR = 1 in 1/6 and with CR and CC = 1 in 1/6; 4 animals fully reversed by D7	X
650	1,2,3-Trimercaptopropane	4756-13-2	Thioalcohol	L	Y	ECETOC	Unknown	Unknown	1 of 1	SCNM<br>(Cat 2)	Conj mean ≥ 2	2/3					Study terminated on D3 with CC = 1 in 1/6 and with CR and CC = 1 in 1/6; 1 animal fully reversed by D2	X
651	Chlorhexidine HCL	3697-42-5	Aryl halide| Guanidine	S	Y	ZEBET	Sigma-Aldrich	≥98%	1 of 1	SCNM<br>(Cat 2)	Conj mean ≥ 2	2/3					Study terminated on D3	X
652	Dodecanal (1% in Alcohol SDA 39C)	112-54-9	Aldehyde	L	Y	NICEATM	Sigma-Aldrich (for Dodecanal);<br>Fisher Scientific (for Alcohol SDA 39C)	≥95%	1 of 1	SCNM<br>(Cat 2B)	Conj mean ≥ 2	2/2					2 animals only, both fully reversed by D7<br><br>SDA = special denatured alcohol (99.01% (v/v) Ethanol + 0.99% (v/v) Diethylphthalate)	X
653	1,2-Expoxydodecane	2855-19-8	Epoxide| Saturated heterocyclic fragment	L	Y	ZEBET	Alfa Aesar	95%	1 of 1	SCNM<br>(No Cat)	CO > 0 **	** CO 1/3					Study terminated on D3	X
654	1-Methoxy-2-propanol	107-98-2	Alcohol| Ether	L	Y	NICEATM	Sigma-Aldrich	≥99.5%	1 of 1	SCNM<br>(No Cat)	CO > 0 **	** CO 2/6; Conj 2/6					Study terminated on D8 with CO = 1 in 2/6, CR = 2 in 1/6 and CR = 1 in 4/6	X
655	Butyl alcohol	78-92-2	Alcohol	L	Y	NICEATM	Sigma-Aldrich	≥99.5%	1 of 1	SCNM<br>(No Cat)	CO > 0 **	** CO 2/6; Conj 2/6					Animal 6 killed on D7 without explanation with CO = 3, IR = 2 and CR = 2; 3 animals fully reversed by D7; 2 animals fully reversed by D14	X
656	Carsonon N-6 (5%) (Chemical name: Nonyl phenol ethoxylate, branched)	9016-45-9 (127087-87-0 reported in NICEATM ALTTOX, which corresponds to Tergitol NP nonionic surfactants)	Alcohol| Benzyl| Ether	L	Y	NICEATM	Unknown	Unknown	1 of 1	SCNM<br>(No Cat)	CO > 0 **	** CO 1/6; IR 1/6					Study terminated on D7 with CO = 2 and CR = 1 in 1/6; 3 animals fully reversed by D3; 2 animals fully reversed by D4	X
657	Diepoxide 126 (Chemical name: 3,4-Epoxycyclohexylmethyl 3,4-epoxycyclohexane carboxylate)	2386-87-0	Alkane, branched with tertiary carbon| Carboxylic acid ester| Cycloalkane| Fused saturated heterocycles| Saturated heterocyclic fragment	L	Y	ZEBET	Sigma-Aldrich	Unknown	1 of 1	SCNM<br>(No Cat)	CO > 0 **	** CO 1/3					Study terminated on D3	X
658	n-Butyl glycidyl ether	2426-08-6	Alkoxy| Epoxide| Ether| Saturated heterocyclic fragment	L	Y	NICEATM	Sigma-Aldrich	97%	1 of 1	SCNM<br>(No Cat)	CO > 0 **	** CO 2/6; Conj 2/6; IR 1/6					Study terminated on D3	X
659	Silan 103 (Chemical name: Trimethoxypropylsilane)	1067-25-0	AlkoxySilane	L	Y	ZEBET	Sigma-Aldrich	97%	1 of 1	SCNM<br>(No Cat)	CO > 0 **	** CO 1/3; Conj 1/3					Study terminated on D3	X
660	Ammonium persulphate	7727-54-0	N/A	S	Y	ZEBET	Sigma-Aldrich	≥98%	1 of 1	SCNM<br>(No Cat)	CO > 0 **	** CO 1/3					Study terminated on D3	X
661	Fomesafen, acid form	72128-02-0	Alkyl halide| Aryl| Aryl halide| Benzamide| Ether| Sulfonamide	S	Y	ECETOC	Unknown	Unknown	1 of 1	SCNM<br>(No Cat)	CO > 0 **	** CO 1/6					Study terminated on D7 with CR = 1 in 1/6 and with CC = 1 in 1/6; 1 animal fully reversed by D2; 3 animals fully reversed by D7;<br>Fomesafen (CAS # 72178-02-0) available from Sigma	X
662	1,5-Dimethyl cyclo-octadiene	3760-14-3	Allyl| Cycloalkene	L	Y	ECETOC	Unknown	Unknown	1 of 1	SCNM<br>(No Cat)	CO > 0						Study terminated on D7 with CR = 1 in 1/6; 4 animals fully reversed by D2; 1 animal fully reversed by D7	X
663	alpha-Lactid	4511-42-6	Dioxane| Lactone| Saturated heterocyclic fragment	S	Y	ZEBET	Sigma-Aldrich	98%	1 of 1	SCNM<br>(No Cat)	CO > 0						Study terminated on D4	X
664	Dibromoneopentyl glycol	3296-90-0	Alcohol| Alkane branched with quaternary carbon| Alkyl halide	S	Y	NICEATM	Sigma-Aldrich	98%	1 of 1	SCNM<br>(No Cat)	CO > 0						Study terminated on D8 with CR = 1 in 2/6	X
665	L-Lysine monohydrate	39665-12-8	Aliphatic Amine, primary| Alpha amino acid	S	Y	ZEBET	Sigma-Aldrich	≥98%	1 of 1	SCNM<br>(No Cat)	CO > 0						Study terminated on D3	X
666	Sodium cyanate	917-61-3	N/A	S	Y	ZEBET	Sigma-Aldrich	96%	1 of 1	SCNM<br>(No Cat)	CO > 0						Study terminated on D3	X
667	2,4-Difluoronitrobenzene	446-35-5	Aryl halide| Nitrobenzene	L	Y	ECETOC	Sigma-Aldrich	99%	1 of 1	SCNM<br>(No Cat)	CO = 0 **	** Conj 2/6					Study terminated on D7 with CR = 1 in 1/6; 5 animals fully reversed by D7	X
668	Ethylbutanal	97-96-1	Aldehyde	L	Y	ZEBET	VWR	98%	1 of 1	SCNM<br>(No Cat)	CO = 0 **	** Conj 1/3; IR 1/3					Study terminated on D7	X
669	Tripropylene glycol monomethyl ether	25498-49-1	Alcohol| Ether	L	Y	NICEATM	Sigma-Aldrich	≥97.5%	1 of 1	SCNM<br>(No Cat)	CO = 0 **	** IR 2/6; Conj 1/6					Study terminated on D3	X
670	C12-16 blend alpha olefin	Unknown	N/A	L	N	NICEATM	Unknown	Unknown	1 of 1	SCNM<br>(No Cat)	CO = 0						Study terminated on D3 with CR = 1 in 4/6;<br>RTECS # AZ7987000	X
671	C6 alpha-Olefin	68855-57-2	N/A	L	N	NICEATM	Unknown	Unknown	1 of 1	SCNM<br>(No Cat)	CO = 0						Study terminated on D3 with CR = 1 in 2/6	X
672	Ethoxyquin	91-53-2	Alkoxy| Allyl| Dihydroquinoline/ Dihydroisoquinoline| Ether	L	Y	EURL ECVAM	Sigma-Aldrich	analytical standard	1 of 1	SCNM<br>(No Cat)	CO = 0						Study terminated on D3 with CR = 1 in 1/6	X
673	iso-Myristyl alcohol	5333-48-2	Alcohol| Alkane, branched with tertiary carbon	L	Y	ECETOC	Sigma-Aldrich	Unknown	1 of 1	SCNM<br>(No Cat)	CO = 0						Study terminated on D3 with CR = 1 in 1/3; 1 animal fully reversed by D1; 1 animal fully reversed by D2	X
674	Polymethylenepolyphenylenepolyamine	25214-70-4	N/A	L	Y	NICEATM	Unknown	Unknown	1 of 1	SCNM<br>(No Cat)	CO = 0						Study terminated on D3 with CR = 1 in 2/6	X
675	Tergitol nonionic surfactant min-foam 2x (Alcohols,C11-15-secondary, ethoxylated propoxylated)	68551-14-4	N/A	L	Y	NICEATM	Dow	100%	1 of 1	SCNM<br>(No Cat)	CO = 0						Study terminated on D3 with CR = 1 in 3/4;<br>Tergitol® min foam 1x (CAS # 68551-14-4) available from Sigma	X
676	Sodium sulphite	7757-83-7	N/A	S	Y	ZEBET	Sigma-Aldrich	≥98%	1 of 1	SCNM<br>(No Cat)	CO = 0						Study terminated on D3	X
677	Tetrabromobisphenol A (2 of 2)	79-94-7	Aryl halide| Phenol	S	Y	NICEATM	Sigma-Aldrich	97%	2 of 2	SCNM<br>(No Cat)	CO = 0						Study terminated on D3 with CR = 1 in 3/6	
678	Silan 167 (Chemical name: Trimethoxy-[3-(3-trimethoxysilylpropyldisulfanyldisulfanyl)propyl]silane)	41453-78-5	AlkoxySilane| Sulfide, poly	Unknown	N	ZEBET	Unknown	Unknown	1 of 1	SCNM<br>(No Cat)	CO = 0						Study terminated on D3; CR = 1 on D3 in 1/3	X
679	Diethyl phthalate	84-66-2	Aryl| Carboxylic acid ester	L	Y	NICEATM	Sigma-Aldrich	99.5%	1 of 1	SCNM<br>(No Cat)	NA						Data only for D1 with CR = 1 in 2/6	X
680	Anisole	100-66-3	Aryl| Ether	L	Y	ZEBET	Sigma-Aldrich	≥99%	1 of 1	SCNM	Conj mean ≥ 2	1/1	CO pers D7; Conj pers D7	1/1; 1/1			1 single animal; Study terminated on D7; CO, CR and CC = 2 on D7	X
681	Dow Corning Siloxanes and Silicones, Di-Me (35% aq dispersion) (Chemical name: Polydimethylsiloxane)	63148-62-9	Silane	L	Y	NICEATM	SCBT	100%	1 of 1	SCNM	IR mean ≥ 1	1/2					2 animals only, 1 fully reversed by D3 and the other fully reversed by D4; 1 animal with IR > 1	X
